# Pd-Catalyzed C(sp^2^)–H/C(sp^2^)–H Coupling of Limonene

**DOI:** 10.1021/acs.joc.4c00501

**Published:** 2024-07-18

**Authors:** Marco Di Matteo, Anna Gagliardi, Alexandre Pradal, Luis F. Veiros, Fabrice Gallou, Giovanni Poli

**Affiliations:** † Institut Parisien de Chimie Moléculaire (IPCM), Faculté des Sciences et Ingénierie, CNRS, Sorbonne Université, 4 Place Jussieu, 75005 Paris, France; ‡ Centro de Química Estrutural, Institute of Molecular Sciences, Departamento de Engenharia Química, Instituto Superior Técnico, Universidade de Lisboa, Av. Rovisco Pais, 1049 001 Lisboa, Portugal; § Novartis Pharma AG, CH-4057 Basel, Switzerland

## Abstract

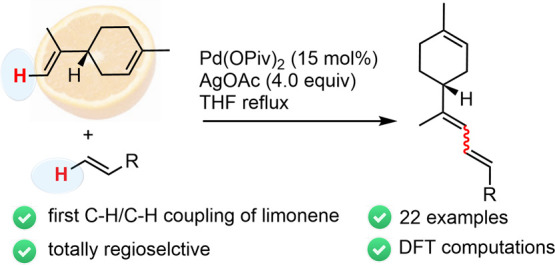

Limonene undergoes a regioselective Pd(II)-catalyzed C(sp^2^)–H/C(sp^2^)–H coupling with acrylic acid esters and amides, α,β-unsaturated ketones, styrenes, and allyl acetate, affording novel 1,3-dienes. DFT computations gave results in accord with the experimental results and allowed for the formulation of a plausible mechanism. The postfunctionalization of one of the coupled products was achieved via a large-scale Sonogashira reaction conducted under micellar catalysis.

## Introduction

It is widely agreed that the achievement of sustainability with the stop of global warming in our society must pass through the steady decrease of the use of fossil feedstock.^1^ On this basis, the gradual replacement of nonrenewable feedstock with biomass appears to be one main measure to take toward this goal.^2^ While this transition is already in full swing for the industrial production of alternative fuels, it is not systematically applied in fine chemistry.^3^ Accordingly, a major role of today’s synthetic chemist consists in valorizing molecules made in bulk amounts by nature, or easily available from waste material, and establishing robust protocols to transform them into drop-in^4^ or dedicated^5^ biobased molecules^6^ of interest in fields such as material and crop sciences, flavor, fragrance, or medicinal chemistry. On the other hand, the catalytic functionalization of an intrinsically unactivated C–H bond in a molecule is a key topic in green chemistry, as it complies with atom- as well as step-economy, adding the C–H bond to the catalogue of the ordinary functional groups, such as halides, alcohols, or carbonyls.^7^

**Scheme 1:**
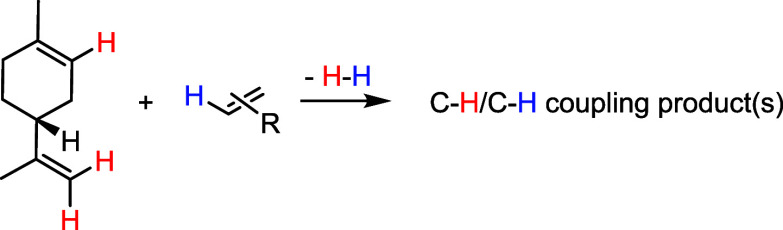
General Project: Discovery of Catalytic C–H/C–H Cross-Coupling Reactions Starting from Monoterpenes

Terpenes are natural hydrocarbons featuring vast structural diversity. Although this class of molecules is considerably less abundant than other primary feedstocks such as lignin, starch, cellulose, proteins, chitin, or triglycerides, they possess a multitude of chemically different C–H bonds.^8^ Therefore, their use as starting material for catalytic C–H activation/functionalization processes appears to be an ideal, yet challenging, combination toward sustainability in chemical synthesis. As part of a new, long-term project dedicated to the catalytic C–H/C–H functionalization of terpenes, we decided to first focus our attention on the palladium-catalyzed C–H/C–H cross-coupling of (+)-limonene (Scheme 1).

**Scheme 2:**
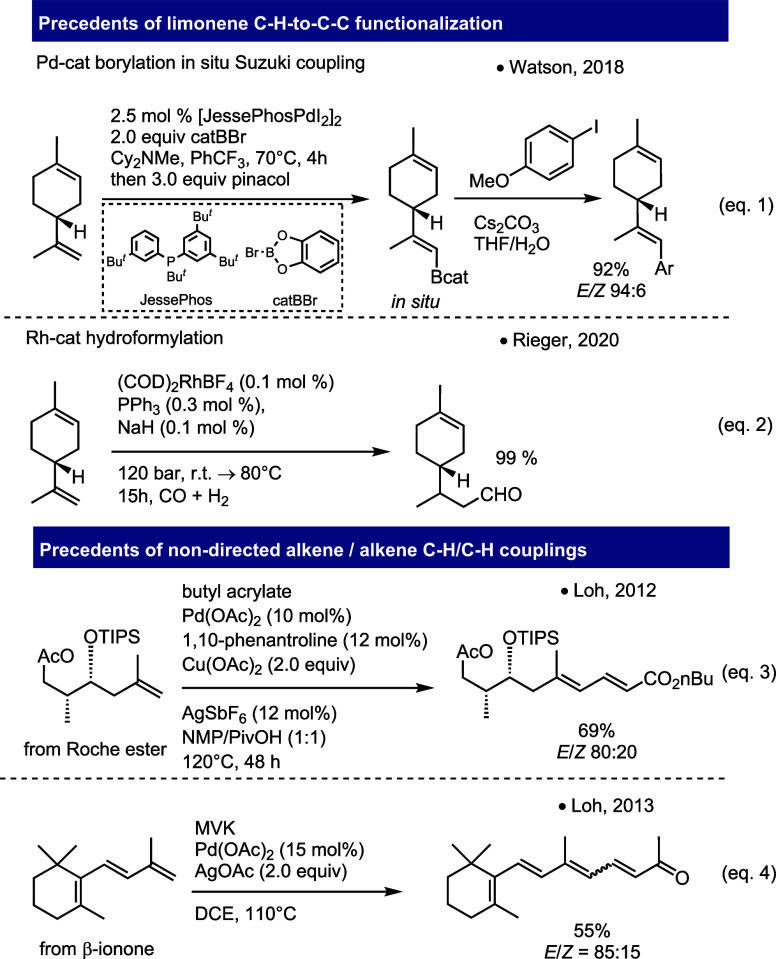
(Top) Precedents of Limonene C–H Functionalization and (Bottom) Precedents of Nondirected Alkene/Alkene CDCs

Available directly as a byproduct of the citrus juice industry or produced biotechnologically by yeasts, limonene is the cheapest monoterpene. With two unsaturations, five allylic C–H bond types (11 C–H bonds in total), and two vinylic C–H bond types (3 C–H bonds in total), it appears a perfect substrate for the development of new C–H activation/functionalization protocols. In this article, we show our results on the Pd-catalyzed regioselective direct C(sp^2^)–H/C(sp^2^)–H (dehydrogenative) cross-coupling^9−13^ between (+)-limonene and alkene partners. To the best of our knowledge, no such cross-coupling has been reported so far, and only two previous C–H functionalizations involving limonene have been described to date. Watson’s group^14^ reported the Pd-catalyzed C–H borylation of limonene to give the corresponding vinylborane, which could be engaged *in situ* in a Suzuki-Miyaura reaction (Scheme 2, eq 1). A second precedent deals with the Rh-catalyzed hydroformylation of limonene, pioneered by Kollár^15^ in 1990, and improved first by Gusevskaya,^16^ then by Rieger^17^ in 2020 (Scheme 2, eq 2).^18^ In a different context, some years ago the group of Loh pioneered nondirected cross-dehydrogenative couplings (CDCs) of intrinsically unactivated 1,1-disubstituted alkenes. In these highly challenging oxidative Pd(II)-catalyzed couplings, alkene reaction partners that are not intrinsically biased for a directed intramolecular metalation come together through C–H bond breaking (Scheme 2, eqs 3 and 4). Thus, building on Loh’s results, we undertook the study of undirected Pd-catalyzed C–H/C–H couplings of terpenes, starting with limonene.

## Results and Discussion

### Choice of the Model Reaction and Optimization

We started our study by reacting (*R*)-(+)-limonene **1a** and ethyl acrylate **2a**(19) in a 1-to-2 ratio, applying the same reaction conditions as those reported by Loh in 2013. Gratifyingly, under these reaction conditions, a regioselective C9–H/C3–H (limonene numbering) dehydrogenative coupling between the exocyclic alkene of limonene and the β-position of the acrylate took place, generating the corresponding sorbate derivative **3aa** in a promising 28% spectroscopic (^1^H NMR) yield (Table 1, entry 1). The reaction was not stereoselective, generating an almost inseparable *E/Z* mixture in a 69:31 ratio. Optimization of the reaction conditions was then undertaken. Among the ethereal solvents (1,4-dioxane, THF, diglyme, and 2-methyltetrahydrofuran), THF turned out to be the best compromise in terms of conversion, yield, and diastereomeric ratio (Table 1, entries 2–5).

**Table 1:**
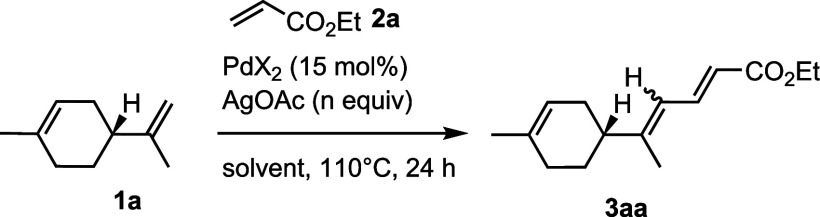
Optimization of the Model C–H-to-C–C Coupling of Limonene^a^

entry	X	*n*	solvent	yield^b^	*E/Z*
1	OAc	2.0	DCE	28	69:31
2	OAc	2.0	dioxane	37	67:33
3	OAc	2.0	THF	36	68:32
4	OAc	2.0	diglyme	c	
5	OAc	2.0	2-Me-THF	34	66:34
6	OAc	2.0	DMSO	<5	50:50
7	OAc	2.0	AcOH	c	
8	OAc	2.0	HFIP	<5	82:18
9	OAc	2.0	DMF	12	51:49
10	OAc	2.0	NMP	9	66:34
11	OAc	2.0	DMSO/PivOH 1:1	5	57:43
12	OCOCF_3_	2.0	THF	6	64:36
13	PdCl_2_(ACN)_2_	2.0	THF	33	65:35
14	PEPPSI-IPr^d^	2.0	THF	19	62:38
15	OPiv	2.0	THF	41	62:38
16	OPiv	4.0	THF	85	75:25

a Reaction conditions: (+)-limonene (0.5 mmol), ethyl acrylate (2.0 equiv), PdX_2_ (15 mol %), solvent (0.2 M), and 110 °C in a sealed vial.

b Measured by quantitative ^1^H NMR, using 1,4-dinitrobenzene as internal standard.

c No conversion was observed.

d [1,3-Bis(2,6-Diisopropylphenyl)imidazol-2-ylidene](3-chloropyridyl)palladium(II) dichloride.

Switch to other dipolar aprotic or protic solvents such as DMSO, AcOH, HFIP, DMF, NMP, or a 1:1 v/v mixture of DMSO/PivOH resulted in either low yields or no conversion (Table 1, entries 6–11). The use of Pd(OCOCF_3_)_2_, PdCl_2_(CH_3_CN)_2_, PEPPSI-IPr, and Pd(OPiv)_2_ as palladium sources (Table 1, entries 12–15) in THF gave the final product in 6, 33, 19, and 41% yield, respectively. In summary, Pd(OAc)_2_ and Pd(OPiv)_2_ (Table 1, entries 3 and 15) in THF turned out to be the best and almost equally performing catalysts. However, due to the variable purity of commercial Pd(OAc)_2_,^20^ we decided to stick to Pd(OPiv)_2_ for the completion of the study. Finally, keeping the reaction conditions of entry 15 and increasing the amount of AgOAc^21^ to 4.0 equiv generated the conjugated dienoate **3aa** in 85% yield and 75:25 4*E*/4*Z* ratio (Table 1, entry 16). Unfortunately, it was not possible to reduce the catalytic loading of the palladium catalyst^22^ nor to switch to greener solvents.^23^

### Study of the Selectivity

**Figure 1:**
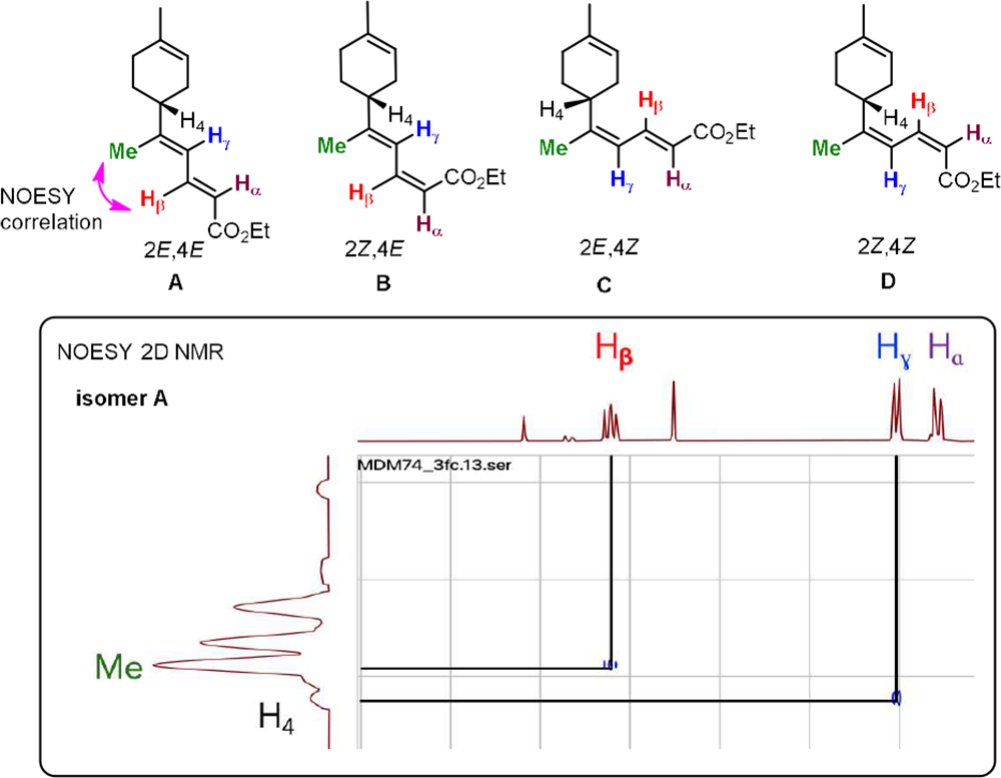
Top: the four possible geometrical isomers of the coupling reaction between **1** and **2**. Bottom: selected region of the NOESY 2D NMR spectrum of major isomer **A**.

The coupling generates only two of the four possible geometrical isomers: **A** (2*E,*4*E*), **B** (2*Z,*4*E*), **C** (2*E,*4*Z*), and **D** (2*Z,*4*Z*) (Figure 1, top). Since the vicinal coupling constant *J*_(Hα/Hβ)_ in the ^1^H NMR spectrum of the two observed isomers is 11.5 Hz, we can rule out isomers **B** and **D**. Furthermore, a NOESY 2D NMR spectrum of an analytically pure sample of the major isomer showed a correlation between the methyl group on C8 (limonene numbering) and H_β_ (Figure 1, bottom). This allowed to assign the configuration 2*E,*4*E* to the major isomer **A** and the 2*E,*4*Z* to the minor isomer **C**.

### Scope of the C–H/C–H Coupling

**Scheme 3:**
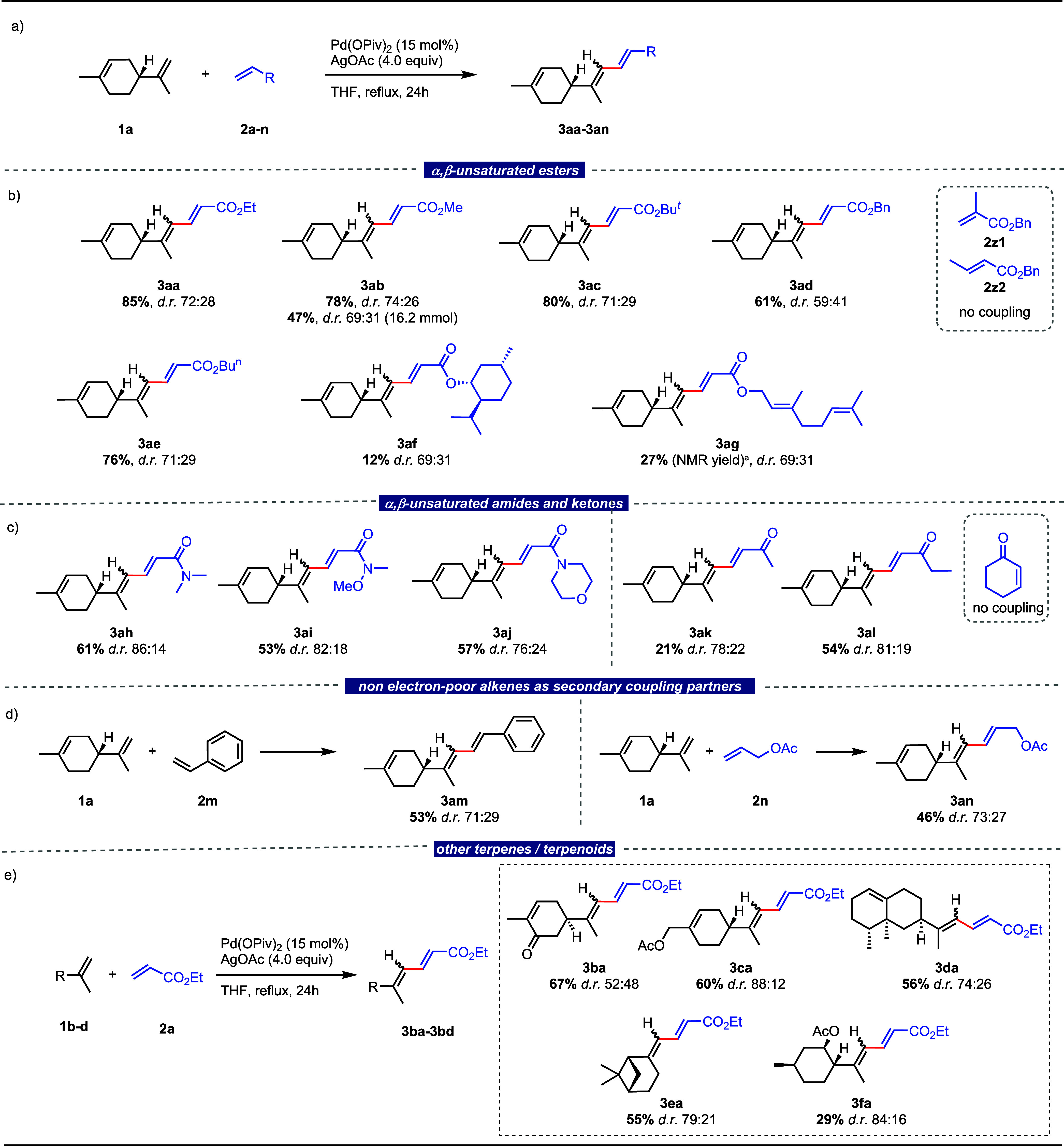
Substrate Scope All reactions were performed on 0.5 mmol scale. Unless otherwise stated, percentage represent isolated yields. Reaction conditions: (+)-limonene (0.5 mmol), electron-poor alkene (2.0 equiv), Pd(OPiv)_2_ (15 mol %), AgOAc (4.0 equiv), THF (0.2 M), and 110 °C (reflux). Yield measured by quantitative ^1^H NMR using 1,4-dinitrobenzene as internal standard.

After the model reaction was optimized, the scope of this dehydrogenative coupling was evaluated by testing other alkenes as reaction partners for limonene (Scheme 3). Reacting limonene with ethyl, methyl, *t*-butyl, benzyl, and *n*-butyl acrylates under the previously optimized reaction conditions gave the expected corresponding dienoates **3aa–3ae** in isolated yields ranging from 61 to 85%.^24^ We then considered the influence of substitution of the partner alkene. Unfortunately, no conversion was observed with benzyl methacrylate **2z1**, while only degradation products were generated with benzyl crotonate **2z2**. Thus, the coupling appears to be incompatible with substitutions at the α- and β-positions of the electron-poor alkenes. More complex acrylate esters such as menthyl and geranyl acrylates gave the corresponding coupling products **3af** and **3ag**, although in low yield (Scheme 3, part b).^25^

Tertiary α,β-unsaturated amides were also good coupling partners, giving the expected coupling products **3ah**, **3ai**, and **3aj** in 53–61% isolated yield. These coupling partners appear to be somewhat less efficient, yet, slightly more stereoselective than acrylates.^26^ Methyl vinyl ketone and ethyl vinyl ketone reacted too, giving the corresponding conjugated dienes in 21%^27^ and 54% isolated yields, respectively. Cyclohex-2-enone gave no conversion, confirming the incompatibility of this protocol with the β-substitution of the α,β-unsaturated alkene (Scheme 3, part c).^28^ A couple of non-electron-poor alkenes were also tested as secondary coupling partners. Accordingly, styrene reacted regioselectively at the less substituted alkene position, affording a 71:29 4*E*/4*Z* mixture of the coupled product **3am** in 53% yield, while allyl acetate gave a 73:27 4*E*/4*Z* mixture of the dienyl allylic acetate **3an** in 46% yield (Scheme 3, part d). Keeping ethyl acrylate as the electron-poor coupling partner, the coupling was also tested with other terpenes or terpenoids carrying an isopropenyl moiety besides an endocyclic alkene functional group. Accordingly, carvone, perillyl acetate, valencene, β-pinene, and isopulegol acetate all reacted exclusively at the exocyclic alkene, providing the corresponding coupling products in 67, 60, 56, 55, and 29% yield (Scheme 3, part e).

### Check for Potential Racemization/Epimerization

**Scheme 4:**
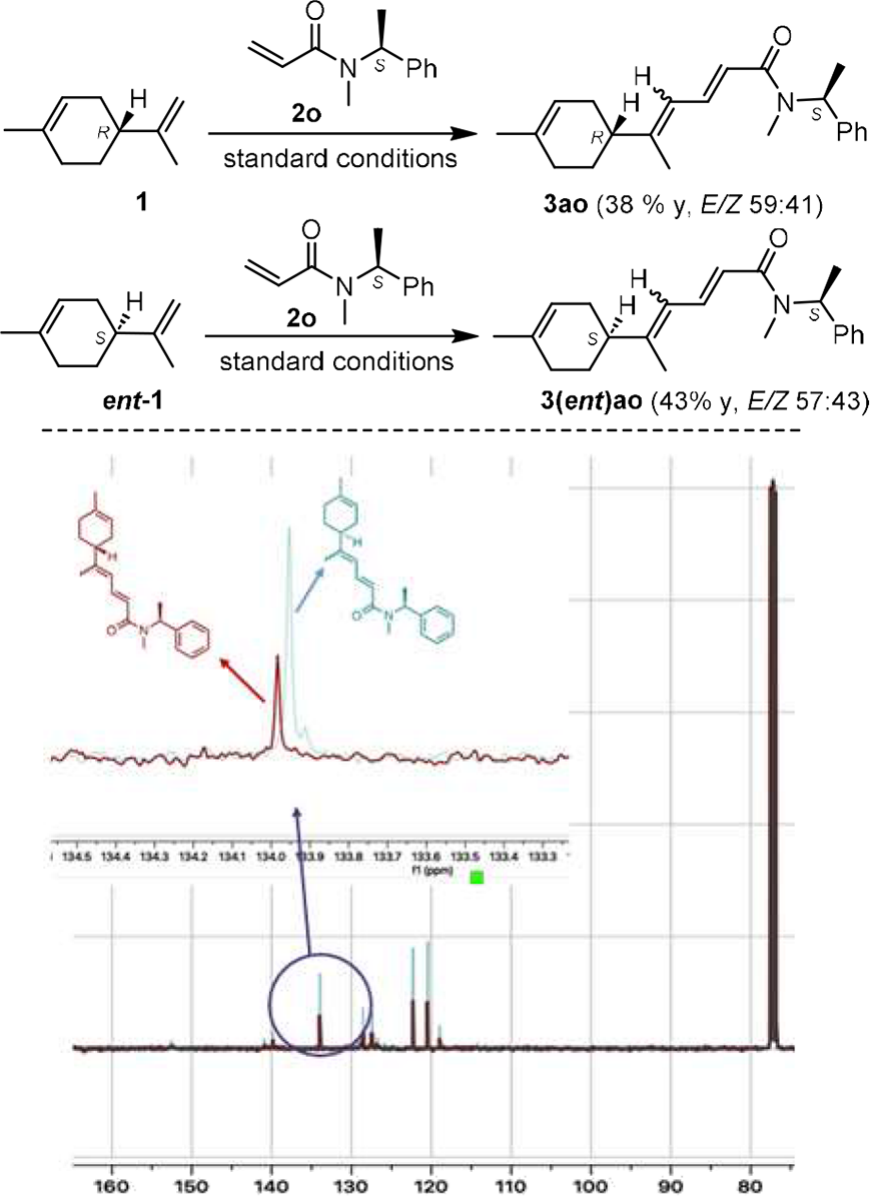
Top: Coupling between (*R*)- or (*S*)-Limonene, and (*S*)-*N*-Methyl-*N*-(1-phenylethyl)acrylamide Conditions: Pd(OPiv)_2_ (15 mol %), AgOAc (4.0 equiv), THF, reflux, 24 h. Bottom: Comparison of the ^13^C-NMR spectra of the products from the coupling between **1** and **2o**, and between *ent***-1** and **2o.**

To confirm that the reaction conditions are not affecting the stereogenic allylic center of limonene, we tested and compared the couplings of (*R*)- and (*S*)-limonene with that of (*S*)-*N*-methyl-*N*-(1-phenylethyl)acrylamide **2o**. We reasoned that in the absence of racemization each coupling should afford a couple of enantiopure *EE*/*EZ* products **3ao** and **3(***ent***)ao** that are different (diastereoisomeric relation) from each other. Contrarily, the four diastereoisomers (as *E/Z* couples) should be produced from each experiment, should the racemization of limonene (or the epimerization of the coupled product) take place. The two couplings were successful, affording products **3ao** and **3(***ent***)ao** in 59:41 and 57:43 *E/Z* ratio, and 38 and 43% yield, respectively (Scheme 4). A careful comparison of the ^13^C spectra of the products of the two coupling reactions (each as an *E/Z* mixture) showed that they are not fully superimposable. Hence, we confirmed that this coupling does not affect the stereochemical integrity of the allylic stereocenter of limonene.

### Study of the Reaction Mechanism

**Scheme 5:**
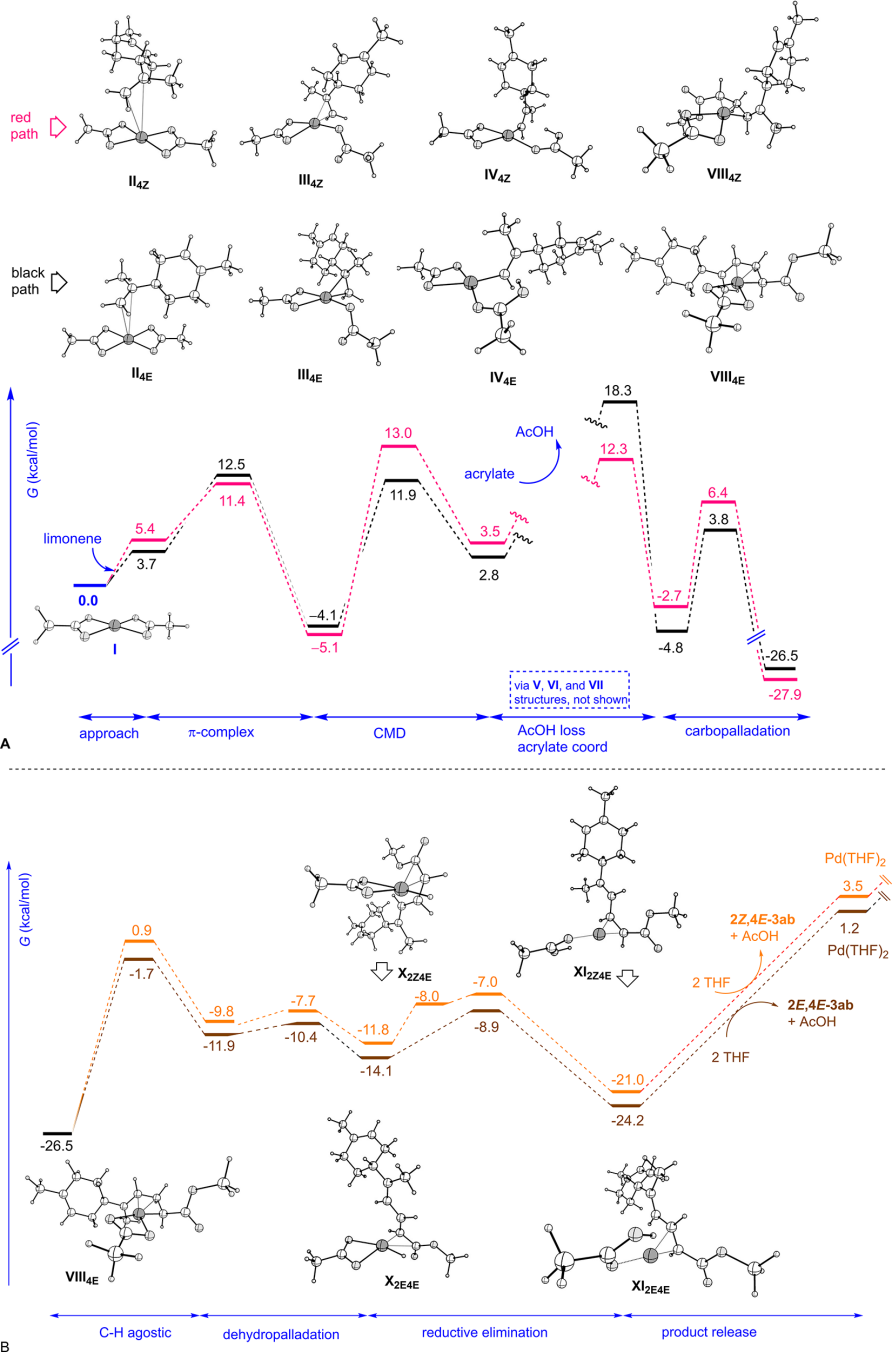
Part A: First Part of the Free Energy Profile of the Pd(OAc)_2_-Catalyzed/AgOAc-Mediated Coupling between Limonene and Methyl Acrylate; From the Starting Complex to the Carbopalladation Step Part B: second part of the energy profile of the Pd(OAc)_2_ catalyzed/AgOAc-mediated coupling between limonene and methyl acrylate; from the carbopalladation complex **VIII**_4E_ to product **2***E***,4***E***-3ab.**

The coupling mechanism between limonene and methyl acrylate to give the sorbate derivative **3ab** was studied via DFT calculations.^29^ As four geometrical isomers can in principle be generated, two of which are experimentally observed, we computationally studied all four pathways (the complete profiles are shown in the Supporting Information). The monomeric complex [Pd(II)(bis-κ^2^(OAc)_2_] **I** was considered as the starting active catalyst (Δ*G* = 0 kcal/mol as a reference) (Scheme 5, part A). Formation of the aggregate between [Pd(II)(bis-κ^2^(OAc)_2_] and the exocyclic alkene of limonene is the first step of the catalytic cycle.^30^ This endergonic step already biases the geometry of the trisubstituted alkene in the final product. Indeed, the two located complexes **II**_4E_ (3.7 kcal/mol, black path) and **II**_4Z_ (5.4 kcal/mol, red path) are already en route toward the *E* and *Z* trisubstituted alkenes, respectively. As to the first path, following full π coordination of the exocyclic alkene of limonene to palladium generates intermediate **III**_4E_ (Δ*G* = −4.1 kcal/mol), passing an energetic barrier of 8.8 kcal/mol. In this step, one acetate ligand passes from κ^2^ to κ^1^-coordination, to allow the π coordination by the alkene. The next step is concerted metalation deprotonation (CMD) wherein the κ^1^-bound acetate ligand intramolecularly deprotonates the terminal H atom of exocyclic alkene *trans* to the C_6_ ring. The step is endergonic, passing an energetic barrier of 16.0 kcal/mol and generating the σ-vinylpalladium complex **IV**_4E_ (2.8 kcal/mol) with the newly generated acetic acid molecule still coordinated. Worthy of note is the metal assistance (d_Pd–H_ = 2.30 Å) in the CMD transition state **TS**_III4E→IV4E_ (see Figure S1 in the Supporting Information). The step is stereoretentive, in that the palladium atom replaces the position of the deprotonated vinylic H atom. Follows a two-step dissociative, globally exergonic ligand exchange between the exiting acetic acid and the entering acrylate, which generates complex **VII**_4E_ (−4.8 kcal/mol, not shown) through a barrier of 15.5 kcal/mol. The subsequent carbopalladation is very exergonic, generating complex **VIII**_4E_ (−26.5 kcal/mol), the resting state of the catalytic cycle, via a barrier of 8.6 kcal/mol.

The higher energy aggregate **II**_4Z_ follows an analogous [π-complex formation/CMD/AcOH-acrylate ligand exchange/carbopalladation] sequence of steps as the one starting from **II**_4E_, leading to intermediate **VIII**_4Z_ (−27.9 kcal/mol, red path). The following step for the two paths (black and red) is a dehydropalladation (β-H elimination) (Scheme 5, part B). This globally endergonic two-step transformation lies at the second bifurcation of the mechanism and determines which of the two diastereotopic allylic H atoms at C3 is involved in the agostic interaction, which is in turn associated with the geometry of the 1,2-disubstituted alkene in the product. In one of these two paths (brown path), intermediate **VIII**_4E_ generates the hydride complex **X**_2E4E_ (−14.1 kcal/mol) stepping over a barrier of 24.8 kcal/mol and passing through the “agostic” intermediate **IX**_2E4E_ (−11.9 kcal/mol). The subsequent reductive elimination is exergonic, affording the Pd(0) complex π-coordinated with the final 2*E*,4*E* product and σ-coordinated with acetic acid **XI**_2E4E_ (−24.2 kcal/mol), passing a barrier of 5.2 kcal/mol. Then, the final products **2***E***,4***E***-3ab** and acetic acid are released from the complex through the entry into the coordination sphere of two solvent molecules that stabilize the Pd atom. This step is endergonic, reaching +1.2 kcal/mol. The alternative path from intermediate **VIII**_4E_ (orange path) follows an analogous [dehydropalladation/reductive elimination/product release] sequence, affording the hydride complex **X**_2Z4E_ (−11.8 kcal/mol), passing through the “agostic” intermediate **IX**_2Z4E_ (−9.8 kcal/mol), while the subsequent reductive elimination gives **XI**_2Z4E_ (−21.0 kcal/mol), and the product release step generates **2***Z***,4***E***-3ab**, Pd(THF)_2_, and AcOH, rising the energy to +3.5 kcal/mol. Inspection of this second part of the mechanism reveals that the brown path lies consistently at much lower energies than the orange path.

It is important to notice that the barrier for going backward from complexes **XI**_2E4E_ and **XI**_2Z4E_ to common resting state **VIII**_4E_ is lower than the one for going forward toward product release (Scheme 5, part B), and this is also true for the corresponding sequence of steps starting from minor resting state **VIII**_4Z_ (see the Supporting Information).

**Figure 2:**
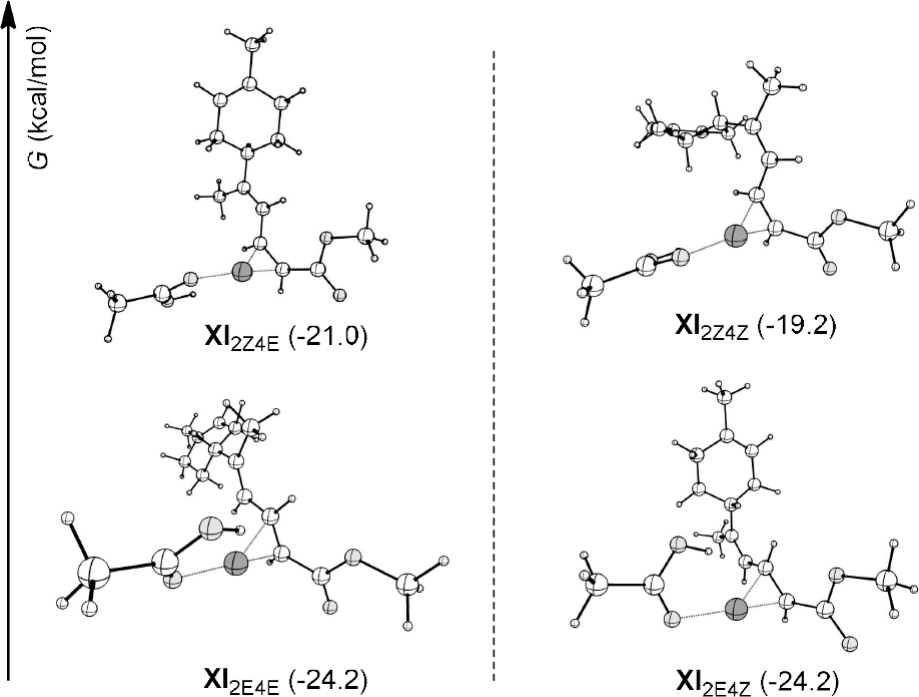
Free energy values of the Pd(0) complexes π-coordinated with the final products and σ-coordinated with acetic acid. Left side: the 4*E* couple (Δ*G*_4E_ = 3.2 kcal/mol); right side: the 4*Z* couple (Δ*G*_4Z_ = 5.0 kcal/mol).

This scenario implies that equilibration between **XI**_2E4E_ and **XI**_2Z4E_ takes place before product release. Accordingly, the 2*E*/2*Z* ratios in the products are expected to depend on the energy differences between **XI**_2E4E_ and **XI**_2Z4E_ for the major 4*E* path, and on that between **XI**_2E4Z_ and **XI**_2Z4Z_ for the minor 4*Z* path. The computed Δ*G*s highly favor the 2*E* stereochemistry in both cases (Δ*G*_4E_ = 3.2 kcal/mol; Δ*G*_4Z_ = 5.0 kcal/mol), which agrees with the fact that only the 2*E* geometrical isomers are experimentally observed (Figure 2).

**Scheme 6:**
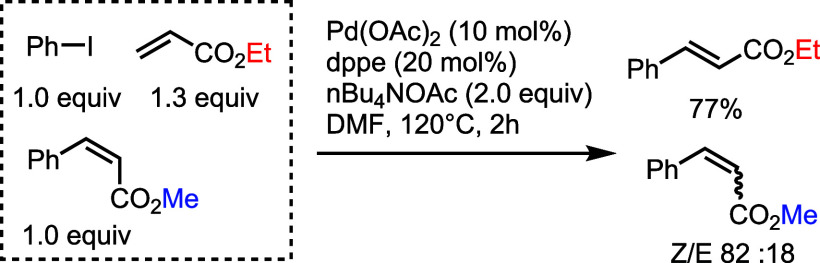
Corollary Experiment to Prove the Reversibility of the Dehydropalladation/Reductive Elimination Sequence

According to the above results, the catalytic cycle is experimentally viable only for the two paths that at the second branching lead to the 2*E* configurated products. To further support the above-mentioned equilibration between the intermediates **XI**_2E_ and **XI**_2Z_, a classical Mizoroki-Heck coupling between ethyl acrylate and phenyl iodide was carried out [Pd(OAc)_2_ (10 mol %), dppe (20 mol %), *n*Bu_4_NOAc (2.0 equiv), DMF, 120 °C, 2 h] in the presence of *Z*-methyl cinnamate (Scheme 6). Besides the formation of the expected *E*-ethyl cinnamate, this corollary experiment restituted an 82:18 *Z*/*E* mixture of methyl cinnamate. Visibly, the Pd(0)/AcOH system was responsible for the isomerization of *Z*-cinnamate (see Scheme S3 in the Supporting Information for the mechanistic detail), which is in accordance with the reversibility of the dehydropalladation/reductive elimination sequence.^31^

### On the Palladium Reoxidation Step

**Scheme 7:**
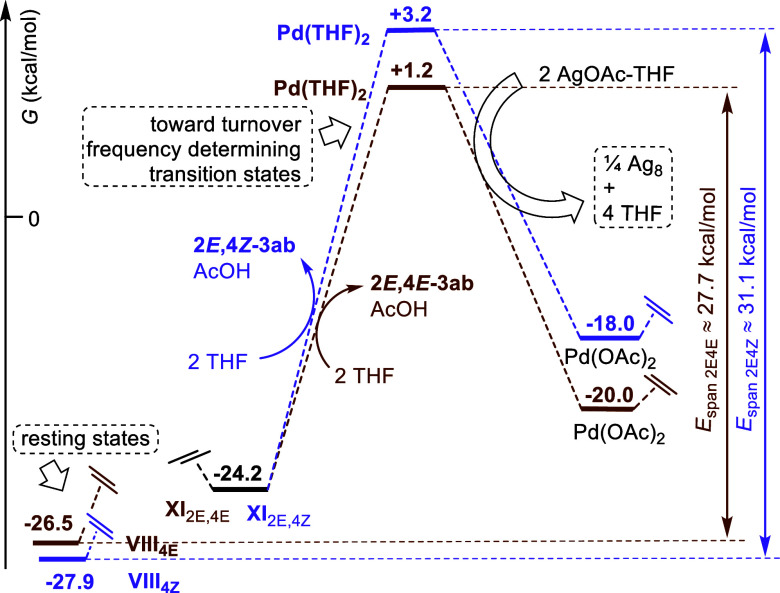
Final Part of the Free Energy Profile of the Pd(OAc)_2_-Catalyzed/AgOAc-Mediated Coupling between Limonene and Methyl Acrylate: From Intermediates **XI**_**2E4E**_ and **XI**_**2E4Z**_ to Pd(OAc)_2_ Regeneration

Coming back to the computational study, the last step of the catalytic cycle is the silver-mediated oxidation of the released Pd(THF)_2_ (Scheme 7).^32,33^ This state is generated after an endergonic product release that reaches 1.2 and 3.2 kcal/mol through a 25.4 and 27.4 kcal/mol rise from the isoenergetic intermediates **XI**_2E4E_ and **XI**_2E4Z_, respectively. In this redox step, Ag(I) is reduced to Ag(0), whose actual state is undetermined. Consideration of the simplest aggregate Ag_2_,^34^ according to the equation [Pd(0)THF_2_ + 2 AgOAc-THF → Pd(OAc)_2_ + Ag_2_ + 4 THF] turns out to be endergonic (Δ_r_*G*° = +2.5 kcal/mol). However, the thermodynamics becomes slightly favorable (Δ_r_*G*° = −11.1 kcal/mol) when the Ag_4_ (*D*_2h_) aggregate is considered as the silver reduced product, according to the equation [2 Pd(0)-THF_2_ + 4 AgOAc-THF → 2 Pd(OAc)_2_ + Ag_4_ + 8 THF], and clearly favorable (Δ_r_*G*° = −84.6 kcal/mol) when the Ag_8_ (*D*_2h_, dodecahedron)^34d,35^ aggregate is considered as the silver reduced product, according to the equation [4 Pd(0)-THF_2_ + 8 AgOAc-THF → 4 Pd(OAc)_2_ + Ag_8_ + 16 THF]. Thus, using the latter stoichiometry, the reaction free energy changes (Δ_r_*G*°) of the catalytic cycles relative to the formation of **2***E***,4***E***-3ab** as well as **2***E***,4***Z***-3ab** become −20.0 and −18.0 kcal/mol, respectively. Therefore, assuming that the states relative to Pd(THF)_2_ are very close in energy and geometry to the corresponding transition states leading to and departing from it, we can predict energetic spans^36^ close to 27.7 kcal/mol [+1.2 – (−26.5)] for the 2*E*,4*E* isomer and to 31.1 kcal/mol [(+3.2 – (−27.9)] for the 2*E*,4*Z* isomer. Here again, the computed energy values are qualitatively in accord with the observed 2*E*,4*E* (major)/2*E*,4*Z* (minor) isomer ratio of the coupled products and also with the experimental conditions (24 h of THF reflux).

**Scheme 8:**
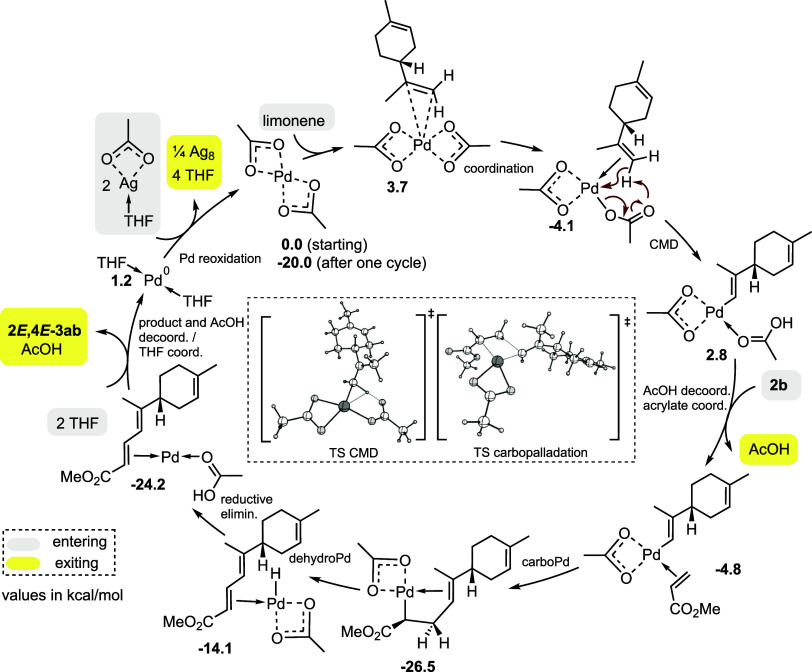
Proposed Mechanism for the Pd-Catalyzed Coupling between Limonene and Methyl Acrylate to Give the Major Product **2***E***,4***E***-3ab**

However, the aggregation state of metallic silver is not the only factor affecting the thermodynamics of the palladium reoxidation step. Indeed, the fact that four equivalents of AcOAg are necessary to obtain optimal yields of couplings suggests that on a macroscopic ground, the excess of oxidant^37^ has the role of pushing the equilibrium toward the product side, thus securing the regeneration of adequate amounts of Pd(OAc)_2_. Therefore, while the *E/Z* isomeric ratio at the trisubstituted C4=C5 double bond is kinetic in origin, resulting from a different energy span, that at C2=C3 results (for each isomer at C4=C5) from a local thermodynamic control due to product equilibration before its release from the corresponding Pd(0) complex precursor. According to the above DFT computations, the mechanism of the full catalytic cycle relative to the coupling between limonene and methyl acrylate to give the major isomer **2***E***,4***E***-3ab** is proposed in Scheme 8.

### Coupling with Allyl Acetate

**Scheme 9:**
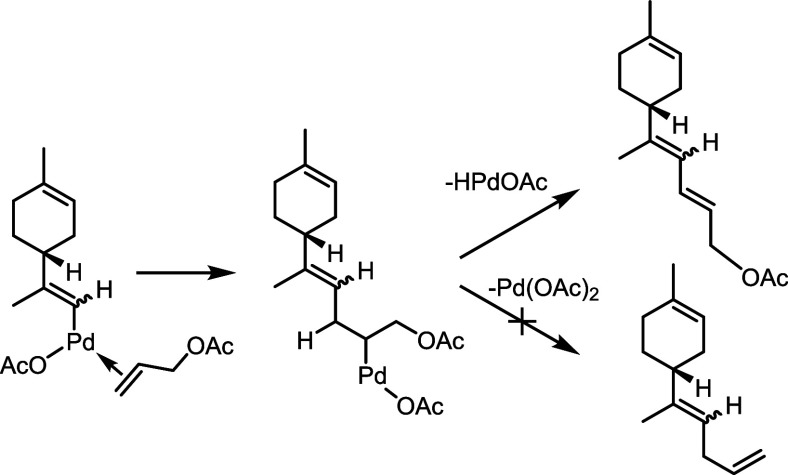
Observed (Top Right) and Nonobserved (Bottom Right) Pathways after the Carbopalladation Step in the Coupling between Limonene and Allyl Acetate

The mechanism of the coupling between limonene and allyl acetate to give **3an** deserves particular comment.^38^ Indeed, the intermediate after the carbopalladation step undergoes dehydropalladation, although deacetoxypalladation could have been an alternative plausible path.^39^ We speculate that the allylic nature of the hydrogen atom involved in the dehydropalladation, which in turn generates a conjugated 1,3-diene, is probably the reason for this behavior (Scheme 9).

**Scheme 10:**
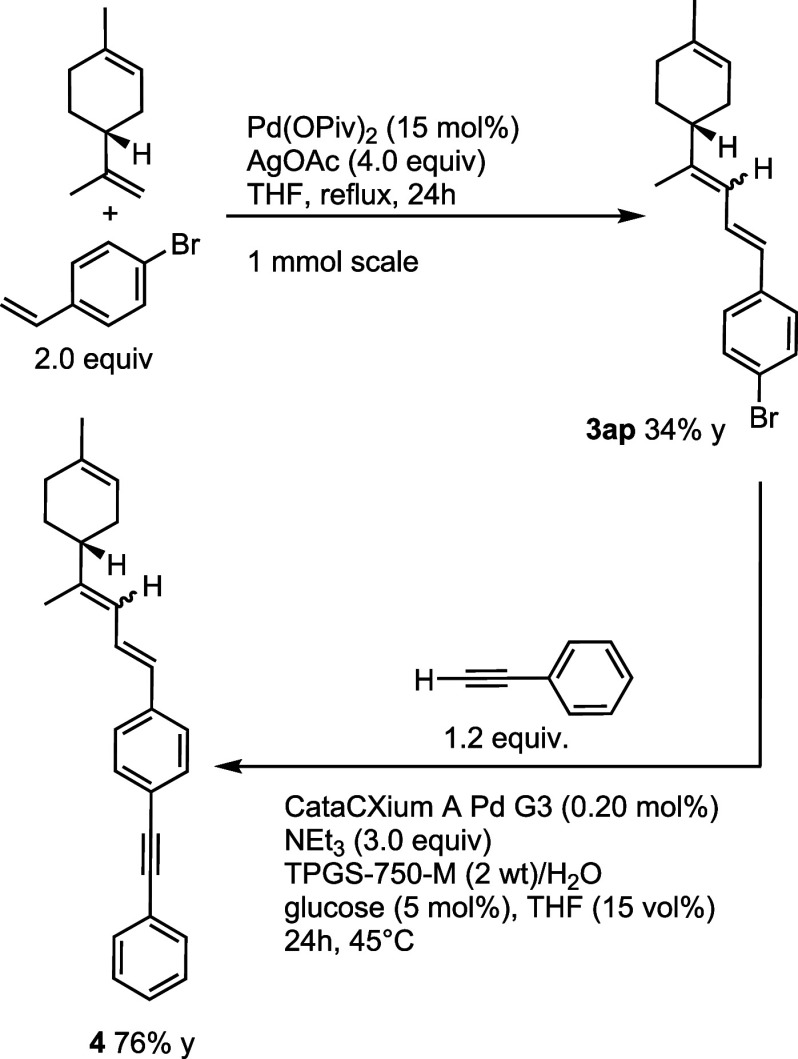
Sonogashira Coupling under Micellar Catalysis from a C–H/C–H Coupling Product, as Postfunctionalization Example

In view of the nature of our mixed academic/industrial collaborative project, and to further demonstrate the usefulness of this C–H-activation-based, catalytic decoration of limonene, we decided to test a postfunctionalization protocol based on micellar catalysis.^40^ This greener type of catalysis may become possible thanks to the presence of surfactants, polymersomes, dendrimers, or nanogels that self-assemble in supramolecular architectures that behave as nanoreactors. In this context, we have recently studied Pd(0) nanoparticles embedded in core–shell nanogels as recoverable catalysts for the Mizoroki–Heck reaction,^41^ and we and others have reported a series of articles demonstrating the power of surfactant-promoted, transition-metal-catalyzed chemistry in water at room temperature with possible catalyst recovery.^42^ Accordingly, we decided to perform the coupling between limonene and *p*-bromostyrene, and, if successful, to test the resulting product in a Sonogashira coupling^43^ using micellar conditions. The planned C(sp^2^)–H/C(sp^2^)–H coupling, using the optimized conditions in THF as the solvent, worked as expected, giving bromodiene **3ap** in 34% yield. The Sonogashira coupling was next tackled. After some experimentation (see Supporting Information for the optimization), we found that treatment of **3ao** (1.0 mmol scale) with ethynylbenzene in the presence of the catalytic system [CataCXium-A-Pd-G3 (0.20 mol %), NEt_3_ (3.0 equiv), TPGS-750-M (2 wt)/H_2_O, glucose (5 mol %)], and THF (15%) as the cosolvent of choice for 24 h at 45 °C gave the desired coupling product **4** in 76% isolated yield (Scheme 10). Notably, these conditions are compared favorably with the classical Sonogashira protocol.^44^ The demonstrated sequence allows for a rapid further elaboration of a valorized limonene platform.

## Conclusions

In conclusion, we have developed the first vinyl/vinyl coupling of limonene with several unsaturated partners such as acrylic esters and amides, α,β-unsaturated ketones, styrenes, and allyl acetate. The reaction turned out to be totally regioselective for both reaction partners, involving exclusively the exocyclic unsaturation of limonene without affecting the integrity of the absolute stereochemistry of the C4 stereogenic center of limonene. As to the stereoselectivity, the reaction constantly produced a mixture of the two geometrical isomers 2*E*,4*E* and 2*E*,4*Z*, out of the four possible, with the former always prevailing. On the other hand, the 2*Z* isomers were never detected. In addition, terpenes other than limonene, possessing an isopropenyl function in addition to an endocyclic alkene, such as carvone, perillyl acetate, and valencene, could also be satisfactorily coupled. DFT computation gave results in qualitative accord with the observed selectivity, allowing the proposal of a plausible mechanism for the coupling.

To further valorize the method developed, a large-scale Sonogashira reaction using a C–H/C–H coupled product as a starting substrate was successfully performed under micellar catalysis conditions, allowing its further selective functionalization. Future work will focus on the development of this C(sp^2^)–H/C(sp^2^)–H coupling in water, on other selective postfunctionalizations, and on new selective catalytic modifications of terpenes.

## Data Availability

The data underlying this study are available in the published article, in its Supporting Information, and openly available in Zenodo (10.5281/zenodo.10462156).

## Supporting Information Available

Detailed optimization data, experimental procedures, characterization data, copies of NMR spectra of all new compounds, and DFT computations with coordinates (PDF)
