# Supplementary material for: Pd-Catalyzed C(sp2)–H/C(sp2)–H Coupling of Limonene
Source: J Org Chem. 2024 Jul 18;89(15):10451–61. doi: 10.1021/acs.joc.4c00501 (PMC11301683; doi:10.1021/acs.joc.4c00501)
Supplement: Supplementary file 1 — jo4c00501_si_001.pdf [file jo4c00501_si_001.pdf]

Supporting Information

Pd-catalyzed $C(sp^2)$–H / $C(sp^2)$–H coupling of Limonene

Marco Di Matteo,[a] Anna Gagliardi,[a†] Alexandre Pradal,[a] Luis F. Veiros,[b] Fabrice Gallou,[c] and Giovanni Poli[a*]*

[a] Institut Parisien de Chimie Moléculaire (IPCM), Sorbonne Université, Faculté des Sciences et Ingénierie, CNRS, 4 Place Jussieu, 75005 Paris, France.

[b] Centro de Química Estrutural, Institute of Molecular Sciences, Departamento de Engenharia Química, Instituto Superior Técnico, Universidade de Lisboa, Av. Rovisco Pais, 1049 001 Lisboa, Portugal.

[c] Novartis Pharma AG, CH-4057 Basel, Switzerland.

alexandre.pradal@sorbonne-universite.fr

giovanni.poli@sorbonne-universite.fr

Table of Contents

1. General Remarks S3
2. Experimental Procedures and Characterization Data S3
3. Synthesis of acrylates. S6
4. Synthesis of acrylamides....S6
5. Synthesis of other terpene derivatives....S7
6. Pd(II)-catalyzed cross dehydrogenative coupling S7
7. Mechanistic hypotheses for the first palladation....S16
8. Mechanistic study....S17
9. Post-functionalization: Cu-free Sonogashira cross-coupling....S18
10. ^{1}H and ^{13}C NMR spectra for unknown compounds S22
11. Computational details S45
12. Coordinates....S57
13. Author Contributions....S108
14. References S108

1. General Remarks

Reactions: All reactions necessitating inert atmosphere were carried out under an argon, by standard syringe and septa techniques. Glassware was flame-dried under vacuum or taken directly from the oven (100 °C) and let cool under vacuum prior to use. Purifications by flash column chromatography were performed using silica-gel Merck Geduran® SI 60 (40-63 µm). Yields refer to chromatographically and spectroscopically pure compounds, unless otherwise mentioned.
Reagents and solvents: Reagents and solvents, including *R*- and *S*-limonene, carvone, perillyl alcohol, valencene, (*S*)-*N*-methyl-1-phenylethan-1-amine, ethyl acrylate, methyl acrylate, and acryloyl chloride, were purchased from commercial sources and generally used as received. Commercially available reagents were purchased at Alfa Aesar, Acros Organics, Sigma Aldrich, TCI Chemicals and Fluorochem suppliers. Solvents were purchased at Carlo Erba. Dichloromethane and THF were dried on a Mbraun purification system MB SPS-800. DMSO and 1,4-dioxane were purchased anhydrous, dried appropriately and kept under inert atmosphere.
TLC: Reactions were magnetically stirred and monitored by thin layer chromatography using Merck- Kieselgel $60F_{254}$ plates and analyzed with either an ultra-violet lamp (λ = 254 nm) and using potassium permanganate or p-anisaldehyde as a stain.
NMR: NMR spectra (^{1}H, ^{13}C and ^{19}F) were recorded on a Bruker AM 300 MHz or on a Bruker AVANCE 400 MHz spectrophotometer. NMR experiments were carried out at room temperature in $CDCl_3$. Chemical shifts are given in parts per million (ppm) using the $CDCl_3$ residual non-deuterated signals as reference (δ ^{1}H = 7.26 ppm; δ ^{13}C = 77.16 ppm). The terms m, s, d, t and q correspond to multiplet, singulet, doublet, triplet and quartet, respectively. The term br. and app. are respectively used when the peak is broad or apparent, and in the latter case when the correct real multiplicity cannot be surely assigned. Coupling constants (J) are given in Hertz (Hz). When mixtures of diastereoisomers were found, the assignable signals are indicated for one of the diastereoisomers with the terms "major" and "minor", respectively. For known compounds, a reference where the characterization can be found was indicated. In addition, 1H NMR data for these products was shown.
IR: IR spectra were recorded with a Tensor 27 (ATR Diamond) Bruker spectrophotometer and Spectrum Two (ATR diamond) PerkinElmer spectrometer. IR spectra were reported as characteristic bands (cm^{-1}).
HRMS: High resolution mass spectra (HRMS) were obtained using a mass spectrometer MicroTOF from Bruker with an electron spray source (ESI) or an atmospheric pressure chemical ionization (APCI) source and a TOF detector at Institut Parisien de Chimie Moléculaire (FR 2769).

2. Experimental Procedures and Characterization Data

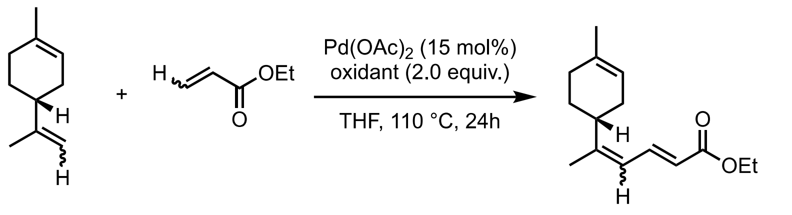

1a **2a** **3aa**

<u>*Variation of the oxidant.*</u>
We investigated the influence of the oxidant, while keeping THF as the solvent and $Pd(OPiv)_2$ as the catalyst, to find out the most efficient Pd(0)-Pd(II) reoxidation agent for the catalytic cycle (Table S1). The best results were obtained with AgOAc, $AgNO_3$ and $AgCO_3$ generating the desired compound **3aa** with 76%, 72% and 69% yield, respectively (Table S1, entries 1-3) while Ag_2O (Table S1, entry 4) led essentially to degradation products. Changing the oxidant to other metal salts such as $Cu(OAc)_2$ and $CuCl_2$ (Table S1, entries 5 and 6) led to the formation of only trace amounts of product. Other oxidants such as 1,4-benzoquinone and derivatives (Table S1, entry 7 and 8), diacetoxyiodobenzene (PIDA), Oxone®, H_2O_2, *tert*-butylhydroperoxide, potassium

persulfate or Selectfluor® led only to degradation being observed (Table S1, entries 9-14). So, AgOAc revealed to be the best oxidant.

Table S1. Screening of the terminal oxidants.

Entry[a]	Oxidant	3aa (%)[b]	Conv. (%)[b]	*d.r.*
**1**	AgOAc	41	76	62:38
**2**	$AgNO_3$	24	72	64:36
**3**	$AgCO_3$	32	69	71:29
**4**	Ag_2O	-[c]	>99	-
**5**	$Cu(OAc)_2$	traces	>99	65:35
**6**	$CuCl_2$	traces	>99	62:38
**7**	BQ	-[c]	>99	-
**8**	DMBQ	-[c]	>99	-
**9**	PIDA	-[c]	>99	-
**10**	Oxone®	-[c]	>99	-
**11**	H_2O_2	-[c]	>99	-
**12**	*t*-BuOOH	-[c]	>99	-
**13**	$K_2S_2O_8$	-[c]	>99	-
**14**	Selectfluor	-[c]	>99	-

[a] typical reaction conditions: (+)-limonene **1a** (0.5 mmol), ethyl acrylate **2a** (2.0 equiv.), $Pd(OPiv)_2$ (15 mol%), oxidant (2.0 equiv.), THF (0.2 M), 110 °C in a sealed vial; [b] measured by quantitative ^{1}H-NMR using 1,4-dinitrobenzene as an internal standard; [c] degradation; [d] formation of an isomeric mixture of oxidized byproducts

Introduction of a ligand.

Since very moderate yields were obtained, we decided to check whether the introduction of a ligand could avoid, or at least moderate, the loss of catalytic activity, and prevent the aggregation of Pd black from the newly generated Pd(0) before its reoxidation. The bidentate White sulfoxide ligand (±)-1,2-bis(phenylsulfinyl)ethane (Table S2, entry 2) or the monodentate phenylvinylsulfoxide ligand[1] (Table S2, entry 3) did not give better yields compared to the previously reported ligandless conditions. Pyridine-type ligands such as 1,10-phenanthroline or 2,2'-bipyridine proved to be much less efficient (Table S2, entries 4 and 5). The Pd/S,O-ligands are known to exhibit great activity and exert a strong influence on site selectivity when compared to the well-established Pd/pyridine-based catalytic system. Unfortunately, in our hands, the use of 3-methyl-2-(phenylthio)butanoic acid did not improve the reaction efficiency (Table S2, entry 6). *N*-protected amino acids are known to favor C–H activation processes via a Concerted Metalation Deprotonation (CMD) mechanism. However, the use of *N*-acetyl-*L*-leucine (Ac-Leu-OH) and *N*-acetyl-*L*-valine (Ac-Val-OH) did not provide better yields than that the reaction carried out under ligandless conditions (Table S2, entries 7-8). Phosphine ligands were also ineffective (Table S2, entry 9-11). In light of the above experiments, we decided to stick to the original ligandless conditions for the rest of the study.

Table S2. Screening of the ligand.

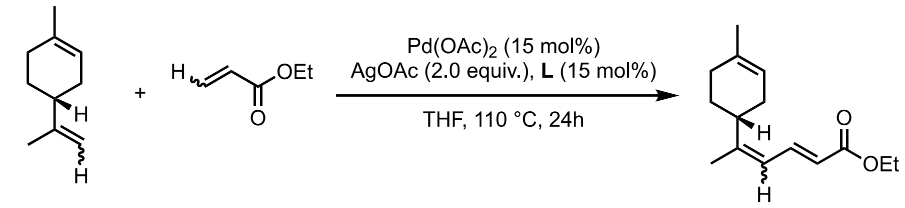

1a **2a** **3aa**

Entry[a]	Ligand	3aa (%)[b]	Conv. (%)[b]	*d.r.*
**1**	No ligand	41	76	62:38
**2**	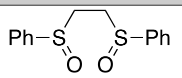	18	82	67:33
**3**	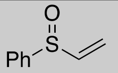	21	86	72:28
**4**	1,10-phenanthroline	traces	>99	-[c]
**5**	2,2'-bipyridine	traces	>99	-[c]
**6**	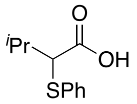	traces	>99	-[c]
**7**	Ac-Leu-OH	23	>99	62:38
**8**	Ac-Val-OH	28	>99	73:27
**9**	*rac*-BINAP	-[d]	>99	-
**10**	XantPhos	-[d]	>99	-
**11**	Dppe	-[d]	>99	-

[a] typical reaction conditions: (+)-limonene **1a** (0.5 mmol), ethyl acrylate **2a** (2.0 equiv.), $Pd(OPiv)_2$ (15 mol%), AgOAc (2.0 equiv.), ligand (15 mol%), THF (0.2 M), 110 °C in a sealed vial; [b] measured by quantitative ^{1}H-NMR, using 1,4-dinitrobenzene as an internal standard; [c] not determined because of overlapping NMR signals; [d] degradation.

Amount of the oxidant.

The amount of AgOAc was considered next (Table S3, entry 1-4). An increase of the amount of the oxidant increased the yield. Specifically, the use of four equivalents of AgOAc turned out to be the best compromise, affording **3aa** in 85% yield (Table S3, entry 3).

Table S3. Influence of the amount of oxidant

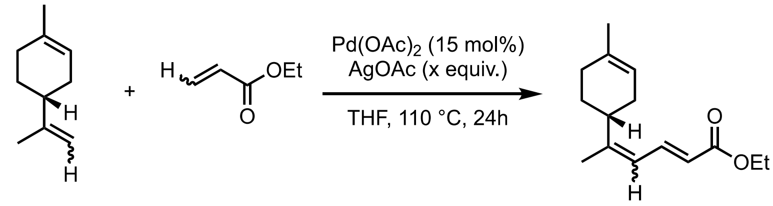

1a **2a** **3aa**

Entry[a]	AgOAc (equiv.)	5ab (%)[b]	Conv. (%)[b]	*d.r.*
**1**	2	41	76	62:38
**2**	3	69	89	64:36
**3**	4	85	>95	74:26
**4**	5	85	>95	73:27

[a] typical reaction conditions: (+)-limonene **1a** (0.5 mmol), ethyl acrylate **2a** (2.0 equiv.), $Pd(OPiv)_2$ (15 mol%), AgOAc (x equiv.), THF (0.2 M), 110 °C in a sealed vial; [b] measured by quantitative ^{1}H-NMR using 1,4-dinitrobenzene as an internal standard.

3. Synthesis of acrylates.

Acrylates **2f** and **2g** were synthesized according to literature procedures.[2]

General procedure A: Synthesis of acrylates

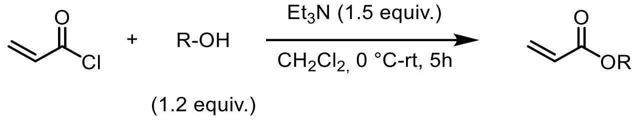

Scheme S1. Synthesis of acrylates

A round bottom flask purged with argon was charged with a solution of the corresponding alcohol (1.2 equiv.) in dichloromethane (0.5 M). Then, triethylamine (1.5 equiv.) was added dropwise at 0 °C. After stirring at this temperature for 15 min, acryloyl chloride (1.0 equiv.) was slowly added. The solution was allowed to warm to rt and stirred for another 5h. The solution was washed with an aqueous 1M solution of HCl and the resulting organic layer was dried with anhydrous magnesium sulfate and filtered. The solvent was removed under reduced pressure and the residue was purified by silica gel column chromatography (pentane/ethyl acetate) to give the corresponding compound.

(1*R*,2*S*,5*R*)-2-isopropyl-5-methylcyclohexyl acrylate (**2f**)

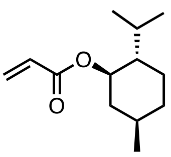

2f

Prepared via the general procedure **A** with 937.6 mg (6.0 mmol, 1.2 equiv.) of (-)-menthol, 1.0 mL (7.5 mmol, 1.5 equiv.) and 0.4 mL (5.0 mmol, 1.0 equiv.) of acryloyl chloride in 10 mL of dry dichloromethane. After work-up, the acrylate **2f** was obtained without further purification as an orange oil (736.2 mg, 70%). The characterization data are in accordance with the ones reported in the literature.[3] **^{1}H NMR** (400 MHz, $CDCl_3$): δ (ppm) 6.35 (dd, *J* = 17.3, 1.6 Hz, 1H), 6.07 (dd, *J* = 17.3, 10.4 Hz, 1H), 5.76 (dd, *J* = 10.4, 1.6 Hz, 1H), 4.73 (td, *J* = 10.9, 4.4 Hz, 1H), 2.02-1.97 (m, 1H), 1.87-1.81 (m, 1H), 1.67-1.62 (m, 2H), 1.52-1.44 (m, 1H), 1.42-1.35 (m, 1H), 1.07-0.93 (m, 3H), 0.88 (d, *J* = 5.6 Hz, 3H), 0.86 (d, *J* = 6.2 Hz, 3H), 0.74 (d, *J* = 7.0 Hz, 3H).

(*E*)-3,7-dimethylocta-2,6-dien-1-yl acrylate (**2g**)

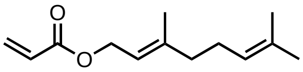

2g

Prepared according to general procedure **A** using 925.5 mg (6.0 mmol, 1.2 equiv.) of geraniol, triethylamine (1.0 mL, 7.5 mmol, 1.5 equiv.) and 0.4 mL (5.0 mmol, 1.0 equiv.) of acryloyl chloride in 10 mL of dry dichloromethane. After work-up, the acrylate **2g** was obtained without further purification as an orange oil (941.8 mg, 90%).The characterization data are in accordance with the ones reported in the literature.[4] **^{1}H NMR** (300 MHz, $CDCl_3$): δ (ppm) 6.37–6.43 (dd, 1H), 6.08–6.17 (m, 1H), 5.79–5.82 (dd, 1H), 5.35–5.39 (m, 1H), 5.05–5.10 (m, 1H), 4.67–4.69 (d, 2H), 2.01–2.12 (m, 4H), 1.72 (s, 3H), 1.68 (s, 3H), 1.60 (s, 3H).

4. Synthesis of acrylamides.

Most of the acrylamides used for the study were commercially available and ready to use, while two of them (**2i** and **2p**) had to be prepared according to reported procedures.

N-methoxy-*N*-methylacrylamide (**2i**)

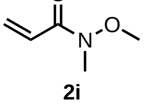

2i

In a round bottom flask was introduced *N*,*O*-dimethylhydroxylamine (1.0 g, 10.3 mmol, 1.1 equiv.). The flask was placed under vacuum before being backfilled with argon. The vacuum/argon cycles were repeated twice and 20 mL of chloroform were added. The solution was cooled down to 0°C (ice/water bath) and acryloyl chloride (0,75 mL, 9.3 mmol, 1.0 equiv.) was added dropwise followed by anhydrous pyridine (1.65 mL, 20.6 mmol, 2.2 equiv.). After stirring 1.5 h at room temperature, the solvent was evaporated. The residue was dissolved in 1M HCl and the aqueous layer was extracted with dichloromethane (3x15 mL). The combined organic layers

were washed with a saturated solution of sodium bicarbonate and brine, dried over anhydrous magnesium sulfate and filtered. The filtrate was evaporated under vacuum to afford the desired acrylonitrile **2i** as a colorless oil (1.16 g, 54% yield). The characterization data are in accordance with the ones reported in the literature.[5] **^{1}H NMR** (400 MHz; $CDCl_3$): δ (ppm) 6.74 (dd, *J* = 17.3 Hz, 10.4 Hz, 1H), 6.44 (dd, *J* = 17.1 Hz, 2 Hz, 1H), 5.76 (dd, *J* = 10.3 Hz,= 2 Hz, 1H), 3.27 (s, 3H), 3.72 (s, 3H).

<u>(*S*)-*N*-methyl-*N*-(1-phenylethyl)acrylamide (**2o**)</u>

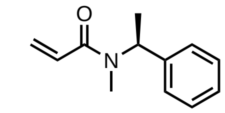

2o

(*S*)-*N*-methyl-1-phenylethan-1-amine (0,7 mL, 5 mmol, 1.0 equiv.) and dry dichloromethane (20 mL) were placed in a flame-dried flask previously purged with argon and triethylamine (1.0 mL, 7.5 mmol, 1.5 equiv.) was added to the solution. The reaction mixture was then cooled to 0 °C. Acryloyl chloride (0.5 mL, 6 mmol, 1.2 equiv.) was added and then the mixture was stirred at rt overnight under inert atmosphere. The reaction was quenched through the addition of aqueous ammonium chloride; the mixture was extracted with dichloromethane (2 × 10 mL) and the organic layers combined, dried over anhydrous magnesium sulfate and concentrated. The residue was purified by flash column chromatography (pentane/ethyl acetate 70:30) to afford the acrylonitrile **2o** as a pale-yellow oil (842.1 mg, 89%). The characterization data are in accordance with the ones reported in the literature.[6] **^{1}H-NMR** (300 MHz, $CDCl_3$): δ (both rotamers) (ppm) 7.37-7.16 (m, 5H), 6.71–6.52 (m, 1H), 6.39–6.28 (m, 1H), 6.09 (q, *J* = 6.9 Hz, 0.6H), 5.69 (app d, *J* = 8.7 Hz, 1H), 5.23 (q, *J* = 6.0 Hz, 0.4H), 2.69 (s, 3H), 1.58 (d, *J* = 6.0 Hz, 1H), 1.48 (d, *J* = 6.9 Hz, 2H).

5. Synthesis of other terpene derivatives.

(-)-Perillyl alcohol was protected to the corresponding acetate **1c** according to a literature protocol.[7]

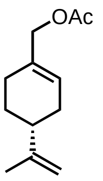

1c

In a round bottom flask was placed 4-DMAP (0.93 mg, 0.15 equiv.). The flask was placed under vacuum before being backfilled with argon. The vacuum/argon cycles were repeated twice and (-)-perillyl alcohol (245.8 mg, 1.55 mmol, 1.0 equiv.) was introduced together with acetic anhydride (172.4 mg, 1.83 mmol, 1.2 equiv.) and triethylamine (0.4 mL, 2.75 mmol, 1.8 equiv.) in THF (0.5 M) at 0°C. The reaction mixture was maintained at 0 °C for 1h and then it was allowed to stir at rt overnight. The solvent was removed under reduced pressure and the resulting material was diluted with ethyl acetate and washed with water and brine. The organic layer was then dried over anhydrous magnesium sulfate. After work-up, the crude product was purified by flash chromatography on silica gel (pentane/ethyl acetate 7:3) to give perillyl acetate **1c** as a colorless oil (127.1 mg, 42%). The characterization data are in accordance the ones reported in the literature.[8] **^{1}H-NMR** ($CDCl_3$, 400 MHz): δ (ppm) 5.73 (s, 1H), 4.73-4.70 (m, 2H). 4.44 (s, 2H), 2.19-2.02 (m, 4H), 2.06 (s, 3H), 1.99-1.92 (m, 1H), 1.87-1.81 (m, 1H), 1.72 (s, 3H), 1.53-1.45 (m, 1H).

6. Pd(II)-catalyzed cross dehydrogenative coupling

General Procedure B for the Pd(II)-catalyzed dehydrogenative coupling

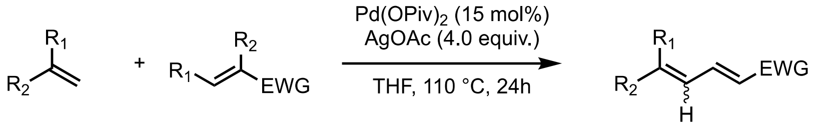

***ent*-1a** or **1a-1f** — **2a-2p** — **3aa-3ap** and ***ent*-3ao/3ab-3fb**

A 10 mL ace pressure tube was charged with 23.2 mg (0.075 mmol, 15 mol%) of palladium (II) pivalate, 333.8 mg (2.0 mmol, 4.0 equiv.) of silver(I) acetate. The tube was placed under vacuum and backfilled with argon. The vacuum/argon cycles were repeated twice and 3.0 mL of freshly distilled THF was added. To this solution

was added the terpene/terpenoid ***ent*-1a** or **1a-1d** (0.5 mmol, 1.0 equiv.) and the electron-poor alkene **2a-2p** (1.0 mmol, 2.0 equiv.). The solution was stirred under inert atmosphere at reflux (heating bath at 110°C) for 24 h. After cooling to rt, the reaction was filtered over a plug of Celite® using 20 mL of dichloromethane. The filtrate was concentrated under reduced pressure and the residue was purified by flash column chromatography on silica gel affording the desired product.

Ethyl (2*E*)-5-((*R*)-4-methylcyclohex-3-en-1-yl)hexa-2,4-dienoate (**3aa**)

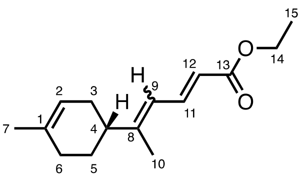

3aa

Prepared according to the general procedure **B** from (+)-limonene **1a** (81.0 µL, 0.5 mmol, 1.0 equiv.) and ethyl acrylate **2a** (106.5 µL, 1.0 mmol, 2.0 equiv.). The crude product was purified by flash column chromatography over silica gel (pentane/ethyl acetate 99:1) to give *ethyl (2E)-5-((R)-4-methylcyclohex-3-en-1-yl)hexa-2,4-dienoate* **3aa** as a yellow oil (100.0 mg, 85%). **^{1}H NMR** (400 MHz, $CDCl_3$): δ (ppm) 7.60 (dd, *J* = 15.1, 11.6 Hz, 1H, H-11), 6.01 (d, *J* = 11.7 Hz, 1H, H-9 major isomer), 5.95 (d, *J* = 12.0 Hz, 1H, H-9 minor isomer), 5.78 (d, *J* = 15.1 Hz, 1H, H-12 major isomer), 5.76 (d, *J* = 15.1 Hz, 1H, H-12 minor isomer), 5.39 (br. s, 1H, H-2), 4.19 (q, *J* = 7.1 Hz, 2H, H-14), 2.17-1.18 (m, 16H, H-3, H-4, H-5, H-6, H-7, H-10, H-15).**^{13}C{^{1}H} NMR** (101 MHz, $CDCl_3$): δ (ppm) 167.86 (C-13 minor isomer), 167.76 (C-13 major isomer), 154.0 (C-1 both isomers), 141.2 (C-11 major isomer), 140.0 (C-11 minor isomer), 134.0 (C-8 minor isomer), 133.9 (C-8 major isomer), 123.9 (C-9 minor isomer), 121.9 (C-9 major isomer), 120.4 (C-2 minor isomer), 120.3 (C-2 major isomer), 119.3 (C-12 major isomer), 119.2 (C-12 minor isomer), 60.5 (C-14 minor isomer), 60.2 (C-14 major isomer), 43.7 (C-4 major isomer), 36.4 (C-4 minor isomer), 30.5 (2C, C-3 and C-6 major isomers), 30.4 (C-3 or C-6 minor isomer), 30.0 (C-3 or C-6 minor isomer), 27.6 (C-5 major isomer), 27.4 (C-5 minor isomer), 23.6 (C-7 minor isomer), 23.5 (C-7 major isomer), 20.3 (C-10 minor isomer), 15.7 (C-10 major isomer), 14.4 (C-15 major isomer), 14.3 (C-15 minor isomer). **IR (ATR)** ν (cm^{-1}): 2913, 1714, 1633, 1444, 1367, 1271, 1150, 1134, 1042, 979, 887, 801, 757. **HRMS (ESI)** m/z: $[M+H]^+$ calcd for $C_{15}H_{22}O_2H$ 235.1693, found 235.1694.

Methyl (*R*)-5-(4-methylcyclohex-3-en-1-yl)hexa-2,4-dienoate (**3ab**)

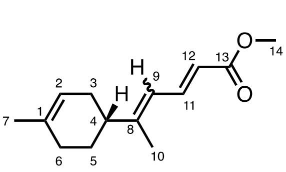

3ab

Prepared according to the general procedure **B** from (+)-limonene **1a** (81.0 µL, 0.5 mmol, 1.0 equiv.) and methyl acrylate **2b** (90.6 µL, 1.0 mmol, 2.0 equiv.). The crude product was purified by flash column chromatography over silica gel (pentane/ethyl acetate 99:1) to give *methyl (R)-5-(4-methylcyclohex-3-en-1-yl)hexa-2,4-dienoate* **3ab** as a brown oil (85.9 mg, 78%).

Scale-up assay:

Prepared according to the general procedure **B** from (+)-limonene **1a** (2.62 mL, 16.2 mmol, 1.0 equiv.) and methyl acrylate **2b** (2.94 mL, 1.0 mmol, 2.0 equiv.). The crude product was purified by flash column chromatography over silica gel (pentane/ethyl acetate 100:0 then 99:1) to give *methyl (R)-5-(4-methylcyclohex-3-en-1-yl)hexa-2,4-dienoate* **3ab** as a brown oil (1.68 g, 47%).

^{1}H NMR (400 MHz, $CDCl_3$): δ (ppm) 7.62 (dd, *J* = 15.2, 11.6 Hz, 1H, H-11), 6.02 (d, *J* = 11.6 Hz, 1H, H-9 major isomer), 5.97 (d, *J* = 11.8 Hz, 1H, H-9 minor isomer), 5.79 (d, *J* = 15.1 Hz, 1H, H-12 major isomer), 5.78 (d, *J* = 15.2 Hz, 1H, H-12 minor isomer), 5.40 (br. s, 1H, H-2), 3.74 (s, 3H, H-14), 2.14-1.20 (m, 13H, H-3, H-4, H-5, H-6, H-7, H-10). **^{13}C{^{1}H} NMR** (101 MHz, $CDCl_3$): δ (ppm) 168.3 (C-13 minor isomer), 168.2 (C-13 major isomer), 154.4 (C-1 major isomer), 148.1 (C-1 minor isomer), 141.5 (C-11 major isomer), 140.3 (C-11 minor isomer), 134.1 (C-8 minor isomer), 134.0 (C-8 major isomer), 123.8 (C-9 minor isomer), 121.9 (C-9 major isomer), 120.4 (C-2 minor isomer), 120.3 (C-2 major isomer), 118.79 (C-12 major isomer), 118.75 (c-12 minor isomer), 51.5 (C-14 both isomers), 43.7 (C-4 major isomer), 36.5 (C-4 minor isomer), 30.5 (2C, C-3 and C-6 both isomers), 30.4 (C-3 or C-6 minor isomer), 30.1 (C-3 or C-6 minor isomer), 27.7 (C-5 major isomer), 27.4 (C-5 minor isomer), 23.65 (C-7 minor isomer), 23.57 (C-7 major isomer), 20.4 (C-10 minor isomer), 15.7 (C-10 major isomer). **IR (ATR)** ν (cm^{-1}): 2914, 1710, 1633, 1434, 1271, 1151, 1137, 979, 887, 793. **HRMS (ESI)** m/z: $[M+H]^+$ calcd for $C_{14}H_{20}O_2H$ 221.1536, found 221.1538.

Tert-butyl (2E)-5-((R)-4-methylcyclohex-3-en-1-yl)hexa-2,4-dienoate (**3ac**)

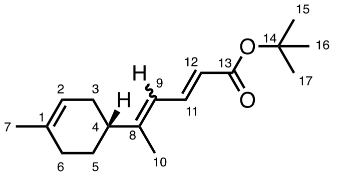

3ac

Prepared according to the general procedure **B** from (+)-limonene **1a** (81.0 µL, 0.5 mmol, 1.0 equiv.) and *tert*-butyl acrylate **2c** (145.2 µL, 1.0 mmol, 2.0 equiv.). The crude product was purified by flash column chromatography over silica gel (pentane/ethyl acetate 99:1) to give *tert*-butyl (2*E*)-5-((*R*)-4-methylcyclohex-3-en-1-yl)hexa-2,4-dienoate **3ac** as a yellow oil (104.6 mg, 80%). **^{1}H NMR** (400 MHz, $CDCl_3$): δ (ppm) 7.52 (dd, *J* = 15.1, 11.6 Hz, 1H, H-11), 5.99 (d, *J* = 11.6 Hz, 1H, H-9 major isomer), 5.93 (d, *J* = 11.5 Hz, 1H, H-9 minor isomer), 5.72 (d, *J* = 15.1 Hz, 1H, H-12 major isomer), 5.71 (d, *J* = 15.1 Hz, 1H, H-12 minor isomer), 5.39 (br. s, 1H, H-2), 2.02-1.61 (m, 13H, H-3, H-4, H-5, H-6, H-7, H-10), 1.48 (s, 9H, H-15, H-16, H-17).**^{13}C{^{1}H} NMR** (75 MHz, $CDCl_3$): δ (ppm) 167.4 (C-13 minor isomer), 167.2 (C-13 major isomer), 153.4 (C-1 minor isomer), 153.2 (C-1 major isomer), 140.2 (C-11 major isomer), 139.2 (C-11 minor isomer), 133.9 (C-8 both isomers), 123.8 (C-9 minor isomer), 121.9 (C-9 major isomer), 121.2 (C-12 major isomer), 121.0 (C-12 minor isomer), 120.5 (C-2 minor isomer), 120.4 (C-2 major isomer), 80.1 (C-14 minor isomer), 80.0 (C-14 major isomer), 43.6 (C-4 major isomer), 36.4 (C-4 minor isomer), 30.5 (2C, C-3 and C-6 major isomers), 30.4 (C-3 or C-6 minor isomer), 30.0 (C-3 or C-6 minor isomer), 28.3 (3C, C-15, C-16, C-17, all 6 isomers), 27.7 (C-5 major isomer) 27.5 (C-5 minor isomer), 23.62 (C-7 minor isomer), 23.56 (C-7 major isomer), 20.3 (C-10 minor isomer), 15.6 (C-10 major isomer). **IR (ATR)** ν (cm^{-1}): 2927, 1708, 1663, 1367, 1284, 1145, 979, 886, 853. **HRMS (ESI)** m/z: $[M+Na]^+$ calcd for $C_{17}H_{26}O_2Na$ 285.1825, found 285.1825.

Benzyl (2E)-5-((R)-4-methylcyclohex-3-en-1-yl)hexa-2,4-dienoate (**3ad**)

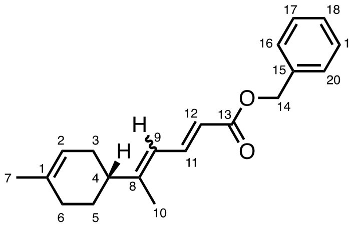

3ad

Prepared according to the general procedure **B** from (+)-limonene **1a** (81.0 µL, 0.5 mmol, 1.0 equiv.) and benzyl acrylate **2d** (153.0 µL, 1.0 mmol, 2.0 equiv.). The crude product was purified by flash column chromatography over silica gel (pentane/ethyl acetate 99:1) to give *benzyl (2E)-5-((R)-4-methylcyclohex-3-en-1-yl)hexa-2,4-dienoate* **3ad** as a yellow oil (91.0 mg, 61%). **^{1}H NMR** (300 MHz, $CDCl_3$): δ (ppm) 7.57 (dd, *J* = 15.2, 11.6 Hz, 1H, H-11), 7.33-7.19 (m, 5H, H-16, H-17, H-18, H-19, H-20), 5.93 (d, *J* = 11.6 Hz, 1H. H-9 major isomer), 5.75 (d, *J* = 15.1 Hz, 1H, H-12), 5.30 (br. s, 1H), 5.10 (s, 2H), 2.17–1.08 (m, 13H). **^{13}C{^{1}H} NMR** (101 MHz, $CDCl_3$): δ (ppm)) 167.7 (C-13 minor isomer), 167.6 (C-13 major isomer), 154.6 (C-1 both isomers), 141.8 (C-11 major isomer), 140.6 (C-11 minor isomer), 136.5 (C-15 major isomer), 136.5 (C-15 minor isomer), 133.99 (C-8 minor isomer), 133.92 (C-8 major isomer), 128.7 (2C, C-16, C-20 both isomers), 128.3 (2C, C-17, C-19 both isomers), 128.2 (C-18 both isomers), 123.8 (C-9 minor isomer), 121.9 (C-9 major isomer), 120.38 (C-2 minor isomer), 120.27 (C-2 major isomer), 118.8 (C-12 major isomer), 118.7 (C-12 minor isomer), 66.1 (C-14 major isomer), 66.0 (C-14 minor isomer), 43.7 (C-4 major isomer), 36.4 (C-4 minor isomer), 30.4 (2C, C-3 or C-6 both isomers), 30.3 (C-3 or C-6 minor isomer), 30.0 (C-3 or C-6 minor isomer), 27.6 (C-5 major isomer), 27.4 (C-5 minor isomer), 23.63 (C-7 minor isomer), 23.55 (C-7 major isomer), 20.4 (C-10 minor isomer), 15.8 (C-10 major isomer). **IR (ATR)** ν (cm^{-1}) 2963, 1715, 1633, 1270, 1150, 1132, 979, 754, 697. **HRMS (ESI)** m/z: $[M+H]^+$ calcd for $C_{20}H_{24}O_2H$ 297.1849, found 297.1849.

Butyl (2E)-5-((R)-4-methylcyclohex-3-en-1-yl)hexa-2,4-dienoate (**3ae**)

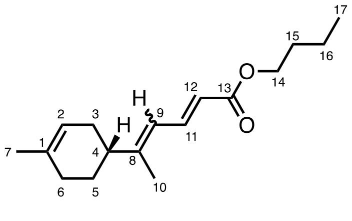

3ae

Prepared according to the general procedure **B** from (+)-limonene **1a** (81.0 µL, 0.5 mmol, 1.0 equiv.) and *n*-butyl acrylate **2e** (142.4 µL, 1.0 mmol, 2.0 equiv.). The crude product was purified by flash column chromatography over silica gel (pentane/ethyl acetate 99:1) to give *butyl (2E)-5-((R)-4-methylcyclohex-3-en-1-yl)hexa-2,4-dienoate* **3ae** as a yellow oil (101.3 mg, 76%). **^{1}H NMR** (400 MHz, $CDCl_3$): δ (ppm) 7.60 (dd, *J* = 15.0, 11.6 Hz, 1H, H-11), 6.01 (d, *J* = 11.6 Hz, 1H, H-9 major isomer), 5.96 (d, *J* = 11.9 Hz, 1H, H-9 minor isomer), 5.79 (d, *J* = 15.2 Hz, 1H, H-12 major isomer), 5.77 (d, *J* = 15.1 Hz, 1H, H-12 minor isomer), 5.39 (br. s, 1H, H-2), 4.14 (t, *J* = 6.7 Hz, 2H, H-14), 2.08-1.21 (m, 17H, H-3, H-4, H-5, H-6, H-7, H-10, H-15, H-16), 0.93 (t, *J* = 7.4 Hz, 3H, H-17). **^{13}C{^{1}H} NMR** (101 MHz, $CDCl_3$): δ (ppm) 168.0 (C-

3 minor isomer), 167.9 (C-3 major isomer), 154.02 (C-1 minor isomer), 154.07 (C-1 major isomer), 141.2 (C-11 major isomer), 140.0 (C-11 minor isomer), 134.1 (C-8 minor isomer), 134.0 (C-8 major isomer), 123.9 (C-9 minor isomer), 121.9 (C-9 major isomer), 120.4 (C-2 minor isomer), 120.3 (C-2 major isomer), 119.3 (C-12 major isomer), 119.2 (C-12 minor isomer), 64.2 (C-14 minor isomer), 64.1 (C-14 major isomer), 43.7 (C-4 major isomer), 35.4 (C-4 minor isomer), 30.9 (C-3 or C-6 major isomer), 30.5 (C-3 or C-6 major isomer), 30.4 (C-3 or C-6 minor isomer), 30.1 (C-3 or C-6 minor isomer), 27.5 (C-5 minor isomer), 27.6 (C-5 major isomer), 23.7 (C-7 minor isomer), 23.6 (C-7 major isomer), 20.4 (C-15 both isomers), 19.3 (C-16 both isomers), 15.7 (C-10 both isomers), 13.9 (C-17 both isomers). **IR (ATR)** ν (cm^{-1}): 2928, 1714, 1633, 1438, 1379, 1273, 1150, 1134, 1066, 979, 887, 794. **HRMS (ESI)** m/z: [M+H]$^+$ calcd for $C_{17}H_{26}O_2H$ 263.2006, found 263.2006.

(1*R*,2*S*,5*R*)-2-isopropyl-5-methylcyclohexyl (2*E*)-5-((*R*)-4-methylcyclohex-3-en-1-yl)hexa-2,4-dienoate (**3af**)

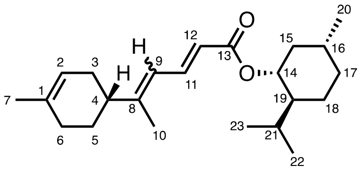

3af

Prepared according to the general procedure **B** from (+)-limonene **1a** (81.0 µL, 0.5 mmol, 1 equiv.) and menthyl acrylate **2f** (223.7 µL, 1.0 mmol, 2.0 equiv.). The crude product was purified by flash column chromatography over silica gel (pentane/ethyl acetate 99:1) to give *(1R,2S,5R)-2-isopropyl-5-methylcyclohexyl (2E)-5-((R)-4-methylcyclohex-3-en-1-yl)hexa-2,4-dienoate* **3af** as a colorless oil (20.6 mg, 12%). **^{1}H NMR** (400 MHz, $CDCl_3$): δ (ppm) 7.60 (dd, *J* = 15.1, 11.6 Hz, 1H, H-11), 6.02 (d, *J* = 11.6 Hz, 1H, H-9), 5.79 (d, *J* = 15.1 Hz, 1H, H-12), 5.40 (br. s, 1H, H-2), 4.76 (td, *J* = 10.9, 4.5 Hz, 1H, H-14), 1.94–0.85 (m, 28H, H-3, H-4, H-5, H-6, H-7, H-10, H-15, H-16, H-17, H-18, H-19, H-21, H-22, H-23), 0.77 (d, *J* = 6.9 Hz, 3H, H-20).**^{13}C{^{1}H} NMR** (101 MHz, $CDCl_3$): δ (ppm) 167.4 (C-3), 153.9 (C-1), 141.1 (C-11), 134.0 (C-8), 122.0 (C-9), 120.4 (C-2), 119.4 (C-12), 73.9 (C-14), 47.4 (C-19), 43.7 (C-4), 41.2 (C-15), 34.5 (C-17 or C-18), 31.6 (C-16), 30.53 (C-3 or C-6), 30.51 (C-3 or C-6), 27.7 (C-5), 26.5 (C-21), 23.8 (C-17 or C-18), 23.6 (C-7), 22.2 (C-22 or C-23), 20.9 (C-22 or C-23), 16.7 (C-20), 15.7 (C-10).**IR (ATR)** ν (cm^{-1}): 2955, 2925, 2360, 1710, 1633, 1455, 1306, 1271, 1193, 1149, 1135, 1014, 981. **HRMS (ESI)** m/z: [M+H]$^+$ calcd for $C_{23}H_{36}O_2H$ 345.2788, found 345.2787.

(*E*)-3,7-dimethylocta-2,6-dien-1-yl (2*E*)-5-((*R*)-4-methylcyclohex-3-en-1-yl)hexa-2,4-dienoate (**3ag**)

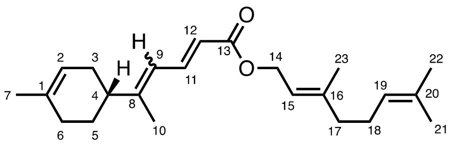

3ag

Prepared according to the general procedure **B** from (+)-limonene **1a** (81.0 µL, 0.5 mmol, 1 equiv.) and geranyl acrylate **2g** (228.9 µL, 1.0 mmol, 2.0 equiv.). The crude product was purified by flash column chromatography over silica gel (pentane/ethyl acetate 99:1) to give *(E)-3,7-dimethylocta-2,6-dien-1-yl (2E)-5-((R)-4-methylcyclohex-3-en-1-yl)hexa-2,4-dienoate* **3ag** as a colorless oil (46.2 mg, 27%). **^{1}H NMR** (400 MHz, $CDCl_3$): δ (ppm) 7.62 (d, *J* = 15.1, 11.5 Hz, 1H, H-11), 6.02 (d, *J* = 11.5 Hz, 1H, H-9), 5.80 (d, *J* = 15.1 Hz, 1H, H-12), 5.55-5.30 (m, 2H, H-2 and H-15), 5.21-5.02 (m, 1H, H-19), 4.67 (d, *J* = 6.9 Hz, 2H, H-14), 1.94–0.85 (m, 28H, H-3, H-4, H-5, H-6, , H-10, H-17, H-18, H-21, H-22), 0.77 (d, *J* = 6.9 Hz, 3H, H-20). **^{13}C{^{1}H} NMR** (101 MHz, $CDCl_3$): δ (ppm) 167.9 (C-3 both isomers), 154.1 (C-1 both isomers), 142.1 (C-16 both isomers), 141.3 (C-11 both isomers), 134.0 (C-8 both isomers), 131.9 (C-20 both isomers), 124.0, (C-19 both isomers) 122.0 (C-9 both isomers), 120.3 (C-2 both isomers), 119.3 (C-12 both isomers), 118.8 (C-15 both isomers), 61.3 (C-14 both isomers), 43.7 (C-4 both isomers), 39.7 (C-17 both isomers), 30.5 (C-3 or C-6 both isomers), 29.9 (C-3 or C-6 both isomers), 27.7 (C-5 both isomers), 26.5 (C-18 both isomers), 25.8 (C-23 both isomers), 23.6 (C-7 both isomers), 17.8 (C-22 or C-21 both isomers), 16.7 (C-22 or C-21 both isomers), 15.7 (C-10 both isomers). **IR (ATR)** ν (cm^{-1}): 2925, 1717, 1637, 1455, 1377, 1271, 1100, 756. **HRMS (ESI)** m/z: [M+Na]$^+$ calcd for $C_{23}H_{34}O_2Na$ 365.2451, found 365.2451.

(2*E*)-*N*,*N*-dimethyl-5-((*R*)-4-methylcyclohex-3-en-1-yl)hexa-2,4-dienamide (**3ah**)

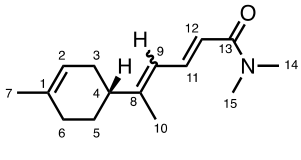

3ah

Prepared according to the general procedure **B** from (+)-limonene **1a** (81.0 µL, 0.5 mmol, 1.0 equiv.) and *N*,*N*-dimethylacrylate **2h** (99.5 µL, 1.0 mmol, 2.0 equiv.). The crude product was purified by flash column chromatography over silica gel (pentane/ethyl acetate 3:7) to give *(2E)-N,N-dimethyl-5-((R)-4-methylcyclohex-3-en-1-yl)hexa-2,4-dienamide* **3ah** as a colorless oil (71.4 mg, 61%). **^{1}H NMR** (400 MHz, $CDCl_3$): δ (ppm) 7.60 (dd, *J* = 14.6, 11.5 Hz, 1H, H-11), 6.24 (d, *J* = 14.6 Hz, 1H, H-12), 6.03 (d, *J* = 11.6 Hz, 1H, H-9), 5.39 (br. s, 1H, H-2),

3.07 (s, 3H, H-14 or H-15), 3.01 (s, 3H, H-14 or H-15), 2.23-1.16 (m, 13H, H-3, H-4, H-5, H-6, H-7, H-10). **^{13}C{^{1}H} NMR** (101 MHz, $CDCl_3$): δ (ppm) 167.6 (C-13 both isomers), 152.2 (C-1 both isomers), 139.2 (C-11 both isomers), 134.0 (C-8 both isomers), 122.2 (C-9 both isomers), 120.5 (C-2 both isomers), 118.5 (C-12), 43.6 (C-4 both isomers), 37.4 (C-14 or C-15 both isomers), 35.9 (C-14 or C-15 both isomers), 30.56 (C-3 both isomers), 30.54 (C-6 both isomers), 27.7 (C-5 both isomers), 23.6 (C-7 both isomers), 15.6 (C-10 both isomers). **IR (ATR)** ν (cm^{-1}): 2925, 1648, 1618, 1589, 1494, 1454, 1394, 1267, 1149, 1123, 979, 887. **HRMS (ESI)** m/z: $[M+H]^+$ calcd for $C_{15}H_{23}NOH$ 234.1852, found 234.1852.

<u>(2*E*)-*N*-methoxy-*N*-methyl-5-((*R*)-4-methylcyclohex-3-en-1-yl)hexa-2,4-dienamide (**3ai**)</u>

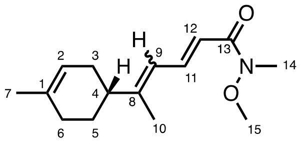

3ai

Prepared according to the general procedure **B** from (+)-limonene **1a** (81.0 µL, 0.5 mmol, 1.0 eq) and *N*-methoxy-*N*-methyl acrylamide **2i** (223.7 µL, 1.0 mmol, 2.0 equiv.). The crude product was purified by flash column chromatography over silica gel (pentane/ethyl acetate 1:1) to give *(2E)-N-methoxy-N-methyl-5-((R)-4-methylcyclohex-3-en-1-yl)hexa-2,4-dienamide* **3ai** as a colorless oil (65.9 mg, 53%). **^{1}H NMR** (300 MHz, $CDCl_3$): δ (ppm) 7.64 (dd, *J* = 14.9, 11.6 Hz, 1H, H-11), 6.37 (d, *J* = 14.9 Hz, 1H, H-12 major isomer), 6.35 (d, *J* = 14.9 Hz, 1H, H-12 minor isomer), 6.07 (d, *J* = 11.5 Hz, 1H, H-9 major isomer), 6.01 (d, *J* = 11.7 Hz, 1J, H-9 minor isomer), 5.38 (br. s, 1H, H-2), 3.69 (s, 3H, H-15), 3.23 (s, 3H, H-14), 2.22-1.48 (m, 13H, H-3, H-4, H-5, H-6, H-7, H-10). **^{13}C{^{1}H} NMR** (75 MHz, $CDCl_3$): δ (ppm) 167.6 (C-13 both isomers), 152.2 (C-1 both isomers), 139.2 (C-11 major isomer), 139.0 (C-11 minor isomer), 133.96 (C-8 minor isomer), 133.91 (C-8 major isomer), 124.4 (C-9 minor isomer), 122.2 (C-9 major isomer), 120.43 (C-2 minor isomer), 120.39 (C-2 major isomer), 117.0 (C-12 major isomer), 116.9 (C-12 minor isomer), 61.8 (C-15 major isomer), 60.5 (C-15 minor isomer), 43.7 (C-4 major isomer), 36.3 (C-4 minor isomer), 32.5 (C-14 both isomers), 30.5 (2C, C-3 and C-6 major isomers), 30.4 (C-3 or C-6 minor isomer), 30.0 (C-3 or C-6 minor isomer), 27.7 (C-5 major isomer), 27.4 (C-5 minor isomer), 23.59 (C-7 minor isomer), 23.54 (C-7 major isomer), 15.6 (C-10 both isomers). **IR (ATR)** ν (cm^{-1}): 2917, 1739, 1655, 1621, 1600, 1412, 1376, 1241, 1175, 1091, 1047, 1004, 886, 801, 704, 618. **HRMS (ESI)** m/z: $[M+H]^+$ calcd for $C_{15}H_{23}NO_2H$ 250.1802, found 250.1802.

<u>(2*E*)-5-((*R*)-4-methylcyclohex-3-en-1-yl)-1-morpholinohexa-2,4-dien-1-one (**3aj**)</u>

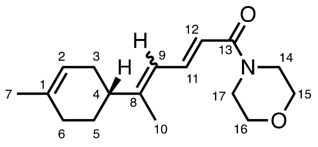

3aj

Prepared according to the general procedure **B** from (+)-limonene **1a** (81.0 µL, 0.5 mmol, 1.0 equiv.) and morpholine acrylamide **2j** (125.8 µL, 1.0 mmol, 2.0 equiv.). The crude product was purified by flash column chromatography over silica gel (pentane/ethyl acetate 1:1) to give *(2E)-5-((R)-4-methylcyclohex-3-en-1-yl)-1-morpholinohexa-2,4-dien-1-one* **3aj** as a colorless oil (78.9 mg, 57%). **^{1}H NMR** (300 MHz, $CDCl_3$): δ (ppm) 7.62 (dd, *J* = 14.7, 11.6 Hz, 1H, H-11), 6.19 (d, *J* = 14.5 Hz, 1H, H-12 major isomer), 6.16 (d, *J* = 14.5 Hz, 1H, H-12 minor isomer), 6.01 (d, *J* = 11.4 Hz, 1H, H-9 major isomer), 5.96 (d, *J* = 10.9 Hz, 1H, H-9 minor isomer), 5.37 (br. s, 1H, H-2), 3.66 (br. s, 8H, H-14, H-15, H-16, H-17), 2.18–1.18 (m, 13H, H-3, H-4, H-5, H-6, H-7, H-10). **^{13}C{^{1}H} NMR** (75 MHz, $CDCl_3$): δ (ppm) 166.8 (C-13 minor isomer), 166.3 (C-13 major isomer), 152.9 (C-1 minor isomer), 152.8 (C-1 major isomer), 140.0 (C-11 major isomer), 138.9 (C-11 minor isomer), 133.91 (C-8 major isomer), 133.87 (C-8 minor isomer), 124.1 (C-9 minor isomer), 122.1 (C-9 major isomer), 120.37 (C-2 minor isomer), 120.34 (C-2 major isomer), 117.5 (C-12 major isomer), 117.3 (minor isomer), 66.9 (2C, C-15 and C-16), 43.6 (C-4 major isomer), 36.3 (C-4 minor isomer), 30.48 (2C, C-3 and C-6 both isomers), 30.46 (2C, C-14 and C-17 both isomers), 27.6 (C-5 major isomer), 27.5 (C-5 minor isomer), 23.57 (C-7 minor isomer), 23.53 (C-7 major isomer), 20.2 (C-10 minor isomer), 15.7 (C-10 major isomer). **IR (ATR)** ν (cm^{-1}): 2919, 1737, 1646, 1594, 1430, 1271, 1231, 1115, 1042, 976, 854. **HRMS (ESI)** m/z: $[M+H]^+$ calcd for $C_{17}H_{25}NO_2H$ 276.1958, found 276.1958.

(3*E*)-6-((*R*)-4-methylcyclohex-3-en-1-yl)hepta-3,5-dien-2-one (**3ak**)

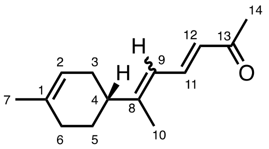

3ak

Prepared according to the general procedure **B** from (+)-limonene **1a** (81.0 µL, 0.5 mmol, 1.0 equiv.) and methylvinylketone **2k** (83.4 µL, 1.0 mmol, 2.0 equiv.). The crude product was purified by flash column chromatography over silica gel (pentane/ethyl acetate 96:4) to give *(3E)-6-((R)-4-methylcyclohex-3-en-1-yl)hepta-3,5-dien-2-one* **3ak** as a colorless oil (21.6 mg, 21%). **^{1}H NMR** (400 MHz, $CDCl_3$): δ (ppm) 7.47 (dd, *J* = 15.3, 11.4 Hz, 1H, H-11), 6.09 (d, *J* = 15.3 Hz, 1H, H-12 major isomer), 6.08 (d, *J* = 15.2 Hz, 1H, H-12 minor isomer), 6.04 (d, *J* = 11.3 Hz, 1H, H-9 major isomer), 5.98 (d, *J* = 12.2 Hz, 1H, H-9 minor isomer), 5.40 (br. s, 1H, H-2), 2.27 (s, 3H, H-14), 2.10-1.18 (m, 13H, H-3, H-4, H-5, H-6, H-7, H-10). **^{13}C{^{1}H} NMR** (101 MHz, $CDCl_3$): δ (ppm) 198.9 (C-13 both isomers), 155.6 (C-1 minor isomer), 155.5 (C-1 major isomer), 139.9 (C-11 major isomer), 138.6 (C-11 minor isomer), 134.2 (C-8 minor isomer), 134.0 (C-8 major isomer), 128.8 (C-12 major isomer), 128.7 (C-12 minor isomer), 124.4 (C-9 minor isomer), 122.4 (C-9 major isomer), 120.4 (C-2 minor isomer), 120.3 (C-2 major isomer), 43.9 (C-4 , major isomer), 36.77 (C-4 minor isomer), 30.5 (2C, C-3 and C-6 major isomers), 30.4 (C-3 or C-6 minor isomer), 30.2 (C-3 or C-6 minor isomer), 27.9 (C-5 or C-14 minor isomer), 27.66 (C-5 or C-14 major isomer), 27.65 (C-5 or C-14 major isomer), 27.5 (C-5 or C-14 minor isomer), 23.7 (C-7 minor isomer), 23.6 (C-7 major isomer), 20.6 (C-10 minor isomer), 15.9 (C-10 major isomer). **IR (ATR)** ν (cm^{-1}) 2923, 1666, 1625, 1587, 1437, 1358, 1255, 1155, 974, 885, 802. **HRMS (ESI)** m/z: $[M+Na]^+$ calcd for $C_{14}H_{20}ONa$ 227.1406, found 227.1407

(4*E*)-7-((*R*)-4-methylcyclohex-3-en-1-yl)octa-4,6-dien-3-one (**3al**)

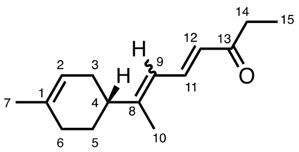

3al

Prepared according to the general procedure **B** from (+)-limonene **1a** (81.0 µL, 0.5 mmol, 1.0 equiv.) and ethylvinylketone **2l** (99.6 µL, 1.0 mmol, 2.0 equiv.). The crude product was purified by flash column chromatography over silica gel (pentane/ethyl acetate 99:1) to give *(4E)-7-((R)-4-methylcyclohex-3-en-1-yl)octa-4,6-dien-3-one* **3al** as a colorless oil (58.9 mg, 54%). **^{1}H NMR** (400 MHz, $CDCl_3$): δ (ppm) 7.51 (dd, *J* = 15.2, 11.4 Hz, 1H, H-11), 6.11 (d, *J* = 15.2 Hz, 1H, H-12 major isomer), 6.10 (d, *J* = 15.1 Hz, 1H, H-12 minor isomer), 6.03 (d, *J* = 11.4 Hz, 1H, H-9 major isomer), 5.97 (d, *J* = 12.1 Hz, 1H, H-9 minor isomer), 5.40 (br. s, 1H), 2.74-2.44 (m, 2H, H-14), 2.11-1.22 (m, 13H, H-3, H-4, H-5, H-6, H-7, H-10). 1.11 (td, *J* = 7.4, 2.8 Hz, 3H, H-15). **^{13}C{^{1}H} NMR** (101 MHz, $CDCl_3$): δ (ppm) 201.4 (C-13 both isomers), 155.3 (C-1 minor isomer), 155.2 (C-1 major isomer), 138.6 (C-11 major isomer) 137.5 (C-11 minor isomer), 134.1 (C-8 minor isomer), 134.0 (C-8 major isomer), 127.7 (C-12 major isomer), 127.5 (C-12 minor isomer), 124.4 (C-9 minor isomer), 122.3 (C-9 major isomer), 120.4 (C-2 minor isomer), 120.2 (C-2 major isomer), 43.7 (C-4 mjor isomer), 36.6 (C-4 minor isomer), 34.3 (C-14 minor isomer), 34.0 (C-14 major isomer), 30.49 (2C, C-3 and C-6 major isomer), 30.38 (C-3 or C-6 minor isomer), 30.1 (C-3 or C-6 minor isomer), 27.7 (C-5 major isomer), 27.5 (C-5 minor isomer), 23.4 (C-7 major isomer), 23.1 (C-7 minor isomer), 15.8 (C-10 major isomer), 14.2 (C-10 minor isomer), 8.53 (C-15 major isomer), 8.49 (C-15 minor isomer). **IR (ATR)** ν (cm^{-1}): 2921, 1665, 1625, 1591, 1202, 1115, 1033, 976, 887, 756. **HRMS (API)** m/z: $[M+H]^+$ calcd for $C_{15}H_{22}OH$ 219.1743, found 219.1744.

((1*E*)-4-((*R*)-4-methylcyclohex-3-en-1-yl)penta-1,3-dien-1-yl)benzene (**3am**)

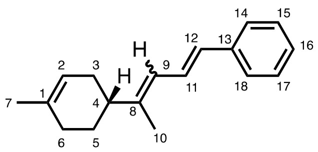

3am

Prepared according to the general procedure **B** from (+)-limonene **1a** (81.0 µL, 0.5 mmol, 1.0 equiv.) and styrene **2m** (115 µL, 1.0 mmol, 2.0 equiv.). The crude product was purified by flash column chromatography over silica gel (pentane 100%) to give *((1E)-4-((R)-4-methylcyclohex-3-en-1-yl)penta-1,3-dien-1-yl)benzene* **3am** as a colorless oil (63.2 mg, 53%). **^{1}H NMR** (400 MHz, $CDCl_3$): δ (ppm) 7.40 (m, 2H, H-14 and H-18), 7.31 (m, 2H, H-15 and H-17), 7.19 (m, 1H, H-16), 7.05 (dd, *J* = 15.5, 10.9 Hz, 1H, H-11), 6.48 (d, *J* = 15.5 Hz, 1H, H-12 major isomer), 6.45 (d, *J* = 15.4 Hz, 1H, H-12 minor isomer), 6.06 (d, *J* = 10.9 Hz, 1H, H-9 major isomer), 6.01 (d, *J* = 10.5 Hz, 1H, H-9 minor isomer), 5.43 (br. s, 1H, H-2), 2.24-1.48 (m, 13H, H-3, H-4, H-5, H-6, H-7, H-10). **^{13}C{^{1}H} NMR** (ppm) (101 MHz, $CDCl_3$): δ 144.6 (C-1 major isomer), 144.4 (C-1 minor isomer), 138.27 (C-13 major isomer), 138.21 (C-13 minor isomer), 134.1 (C-8 minor isomer), 133.9 (C-8 major isomer), 130.4 (C-16 major isomer), 130.3 (C-16 minor isomer), 128.7 (2C, C-14 and C-18 both isomers), 127.08 (C-11 minor isomer), 127.05 (C-11 major isomer), 126.27 (2C, C-15 and C-17 minor isomers), 126.23 (2C, C-15 and C-17 isomers),

125.9 (C-12 major isomer), 125.8 (C-12 minor isomer), 124.9 (C-2 minor isomer), 123.7 (C-2 major isomer), 121.0 (C-9 minor isomer), 120.8 (C-9 major isomer), 43.5 (C-4), 30.76 (2C, C-3 and C-6), 30.74 (2C, C-3 and C-6), 28.0 (C-5), 23.6 (C-7), 15.3 (C-15). **IR (ATR)** ν (cm^{-1}) 3031, 2913, 1596, 1495, 1447, 1072, 959, 914, 801, 747, 691. **GC-MS** (EI, 70 eV): *m/z* 238 (M^+, 12%).[9]

<u>(2*E*)-5-((*R*)-4-methylcyclohex-3-en-1-yl)hexa-2,4-dien-1-yl acetate (**3an**)</u>

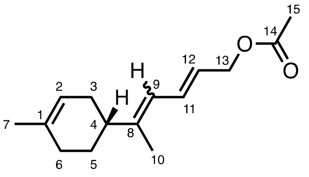

3an

Prepared according to the general procedure **B** from (+)-limonene **1a** (81.0 µL, 0.5 mmol, 1.0 equiv.) and allyl acetate **2n** (107.9 µL, 1.0 mmol, 2.0 equiv.). The crude product was purified by flash column chromatography over silica gel (pentane/ethyl acetate 99:1) to give *(2E)-5-((R)-4-methylcyclohex-3-en-1-yl)hexa-2,4-dien-1-yl acetate* **3an** as a colorless oil (53.5 mg, 46%). **^{1}H NMR** (400 MHz, $CDCl_3$): δ (ppm) 6.56 (dd, *J* = 15.0, 10.9 Hz, 1H, H-11), 5.88 (d, *J* = 10.8 Hz, 1H, H-9 major isomer), 5.82 (d, *J* = 11.5 Hz, 1H, H-9 minor isomer), 5.66 (dt, *J* = 14.1, 6.8 Hz, 1H, H-12), 5.40 (br. s, 1H, H-2), 4.60 (d, *J* = 6.8 Hz, 2H, H-13), 2.06 (s, 3H, H-15), 2.03-1.16 (m, 13H, H-3, H-4, H-5, H-6, H-7, H-10). **^{13}C{^{1}H} NMR** (101 MHz, $CDCl_3$): δ (ppm) 171.0 (C-14 both isomers), 145.5 (C-1 major isomer), 145.4 (C-1 minor isomer), 134.1 (C-8 minor isomer), 133.9 (C-8 major isomer), 131.8 (H-11 major isomer), 130.8 (C-11 minor isomer), 124.2 (C-12 major isomer), 124.1 (C-12 minor isomer), 122.2 (C-9 both isomers), 120.8 (C-2 minor isomer), 120.7 (C-2 major isomer), 66.3 (C-13 minor isomer), 65.5 (C-13 major isomer), 43.3 (C-4 major isomer), 37.2 (C-4 minor isomer), 30.69 (C-3 or C-6 major isomer), 30.62 (C-3 or C-6 major isomer), 30.58 (C-3 or C-6 minor isomer), 29.8 (C-3 and C-6 minor isomer), 27.8 (C-5), 23.8 (C-7 minor isomer), 23.6 (C-7 major isomer), 21.2 (C-10 major isomer), 21.1 (C-10 minor isomer), 19.8 (C-15 minor isomer), 15.1 (C-15 major isomer). **IR (ATR)** ν (cm^{-1}): 2919, 2360, 1741, 1438, 1376, 1227, 1023, 964, 798. **HRMS (ESI)** m/z: $[M+Na]^+$ calcd for $C_{15}H_{22}O_2Na$ 257.1512, found 257.1513.

<u>(2*E*)-*N*-methyl-5-((*R*)-4-methylcyclohex-3-en-1-yl)-*N*-((*S*)-1-phenylethyl)hexa-2,4-dienamide (**3ao**)</u>

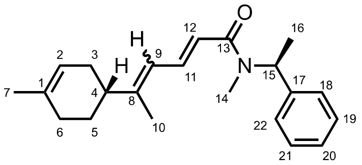

3ao

Prepared according to the general procedure **B** from (+)-limonene **1a** (81.0 µL, 0.5 mmol, 1.0 equiv.) and (*S*)-*N*-methyl-*N*-(1-phenylethyl)acrylamide (189.3 mg, 1.0 mmol, 2.0 equiv.) **2o**. The crude product was purified by flash column chromatography over silica gel (pentane/ethyl acetate 3:7) to give *(2E,4E)-N-methyl-5-((R)-4-methylcyclohex-3-en-1-yl)-N-((S)-1-phenylethyl)hexa-2,4-dienamide* **3ao** as a colorless oil (61.5 mg, 38%). **^{1}H NMR** (400 MHz, $CDCl_3$): 7.72 (m, 1H, H-11), 7.37-7.26 (m, 5H, H-18, H-19, H-20, H-21, H-22), 6.31-5.99 (m, 2H, H-9 and H-12), 5.40 (br. s, 1H, H-2), 2.73 (s, 3H, H-14), 2.35-1.16 (m, 17H, H-3, H-4, H-5, H-6, H-7, H-10, H-15, H-16).**^{13}C{^{1}H} NMR** (101 MHz, $CDCl_3$): δ (ppm) 167.4 (C-13 both isomers), 152.6 (C-1 both isomers), 141.0 (C-11 both isomers), 139.9 (C-17 both isomers), 134.0 (C-8 both isomers), 128.6 (C-9 both isomers), 127.5 (C-2 both isomers), 126.8 (C-12 both isomers), 122.3 (2C, C-18 and C-22 both isomers), 120.5 (2C, C-19 and C-21 both isomers), 119.0 (C-20 both isomers), 50.6 (C-15 both isomers), 43.7 (C-4 both isomers), 30.6 (2C, C-3 and C-6 both isomers), 29.8 (C-14 both isomers), 27.7 (C-5 both isomers), 26.9 (C-16 both isomers), 23.6 (C-7 both isomers), 15.7 (C-10 both isomers). **IR (ATR)** ν (cm^{-1}): 3012, 2933, 1632, 1591, 1497, 1113, 977, 845. **HRMS (ESI)** m/z: $[M+H]^+$ calcd for $C_{22}H_{29}NOH$ 324.2321, found 324.2320.

<u>1-bromo-4-((1*E*)-4-((*R*)-4-methylcyclohex-3-en-1-yl)penta-1,3-dien-1-yl)benzene (**3ap**)</u>

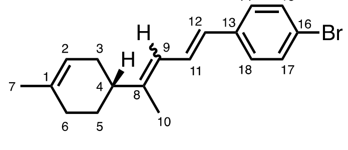

3ap

Prepared according to the general procedure **B** from (+)-limonene **1a** (81.0 µL, 0.5 mmol, 1.0 equiv.) and 4-bromostyrene **2p** (130.8 µL, 1.0 mmol, 2.0 equiv.). The crude product was purified by flash column chromatography over silica gel (cyclohexane 100%) to give *1-bromo-4-((1E)-4-((R)-4-methylcyclohex-3-en-1-yl)penta-1,3-dien-1-yl)benzene* **3ap** as a colorless oil (53.9 mg, 34%). **^{1}H NMR** (400 MHz, $CDCl_3$); δ (ppm) 7.42 (m, 2H, H-15 and H-17), 7.28 (m, 2H, H-14 and H-18), 7.05 (dd, *J* = 15.4, 10.8 Hz, 1H, H-11), 6.41 (d, *J* = 15.5 Hz, 1H, H-12 major isomer), 6.38 (d, *J* = 15.5 Hz, 1H, H-12 minor isomer), 6.05 (d, *J* = 10.9 Hz, 1H, H-9 major isomer), 6.00 (d, *J* = 10.7 Hz, 1H, H-9 minor isomer), 5.44 (br. s, 1H, H-2), 2.32-1.24 (m, 13H, H-3, H-4, H-5, H-6, H-7, H-10). **^{13}C{^{1}H} NMR** (101 MHz, $CDCl_3$): δ (ppm) 145.5 (C-1 major isomer), 145.3 (C-1 minor isomer), 137.2 (C-13 major isomer), 137.1 (C-13 minor isomer), 134.1 (C-8 minor isomer), 134.0 (C-8 minor

isomer), 132.0 (C-15 or C-17 minor isomer), 131.8 (2C, C-15 and C-17 major isomer), 131.7 (C-15 or C-17 minor isomer), 129.1 (C-12 minor isomer), 129.0 (C-12 major isomer), 128.0 (C-14 or C-18 minor isomer), 127.8 (C-14 or C-18 minor isomer), 127.7 (2C, C-14 and C-18 major isomers), 126.7 (C-11 minor isomer), 126.6 (C-11 major isomer), 125.5 (C-9 minor isomer), 123.5 (C-9 major isomer), 121.6 (C-2 or C-9 minor isomer), 120.9 (C-2 or C-16 minor isomer), 120.68 (C-2 or C-16 major isomer), 120.62 (C-16 minor isomer), 43.5 (C-4), 30.7 (2C, C-3 and C-6), 27.9 (C-5), 23.6 (C-7), 15.4 (C-10). **IR (ATR)** ν (cm^{-1}): 2922, 1635, 1487, 1437, 1072, 1008, 961, 850, 802, 758. **GC-MS** (EI, 70 eV): *m/z* 318 (M^+, 24%), 316 (M^+, 24%).[9]

Ethyl (2*E*)-5-((*R*)-4-methyl-5-oxocyclohex-3-en-1-yl)hexa-2,4-dienoate (**3ba**)

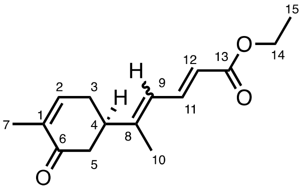

3ba

Prepared according to the general procedure **B** from (-)-carvone **1b** (78.2 µL, 0.5 mmol, 1.0 equiv.) and ethyl acrylate **2a** (106.5 µL, 1.0 mmol, 2.0 equiv.). The crude product was purified by flash column chromatography over silica gel (pentane/ethyl acetate 9:1) to give *ethyl (2E)-5-((R)-4-methyl-5-oxocyclohex-3-en-1-yl)hexa-2,4-dienoate* **3ba** as a colorless oil (83.8 mg, 67%). **^{1}H NMR** (400 MHz, $CDCl_3$): δ (ppm) 7.55 (dd, *J* = 15.2, 11.4 Hz, 1H, H-11), 6.73 (ddd, *J* = 5.7, 2.8, 1.5 Hz, 1H, H-2), 6.01 (d, *J* = 11.4 Hz, 1H, H-9 major isomer), 6.01 (d, *J* = 11.4 Hz, 1H, H-9 minor isomer), 5.82 (d, *J* = 15.1 Hz, 1H, H-12 major isomer), 5.80 (d, *J* = 15.1 Hz, 1H, H-12 minor isomer), 4.18 (q, *J* = 7.1 Hz, 2H, H-14), 2.83-2.75 (m, 1H, H-4), 2.65-2.30 (m, 4H, H-3 and H-5), 1.89 (s, 3H, H-7 or H-10), 1.80-1.76 (m, 3H, H-7 or H-11), 1.27 (t, *J* = 7.1 Hz, 3H, H-15). **^{13}C{^{1}H} NMR** (101 MHz, $CDCl_3$): δ (ppm)) 199.1 (C-6 major isomer), 198.8 (C-6 minor isomer), 167.41 (C-13 minor isomer), 167.37 (C-13 major isomer), 149.2 (C-1 major isomer), 148.6 (C-1 minor isomer), 144.4 (C-2 minor isomer), 144.3 (C-2 major isomer), 140.3 (C-11 major isomer), 138.6 (C-11 minor isomer), 135.8 (C-8 minor isomer), 135.7 (C-8 major isomer), 125.5 (C-9 minor isomer), 123.3 (C-9 major isomer), 121.0 (C-12 major isomer), 120.9 (C-12 minor isomer), 60.42 (C-14 minor isomer), 60.36 (C-14 major isomer), 44.9 (C-4 major isomer), 42.8 (C-5 major isomer), 42.2 (C-4 minor isomer), 37.6 (C-5 minor isomer), 31.0 (C-3 major isomer), 30.6 (C-3 minor isomer), 20.4 (C-7 minor isomer), 15.9 (C-7 major isomer), 15.6 (C-10 both isomers), 14.5 (C-15 both isomers). **IR (ATR)** ν (cm^{-1}): 2982, 2923, 2361, 1710, 1674, 1634, 1366, 1274, 1213, 1196, 1160, 1100, 1041, 979, 900, 859. **HRMS (ESI)** m/z: $[M+H]^+$ calcd for $C_{15}H_{20}O_3H$ 249.1485, found 249.1485.

Ethyl (2*E*)-5-((*R*)-4-(acetoxymethyl)cyclohex-3-en-1-yl)hexa-2,4-dienoate (**3ca**)

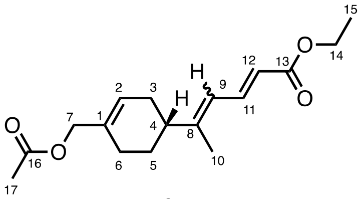

3ca

Prepared according to the general procedure **B** from (-)-perillyl alcohol acetate **1c** (98.9 µL, 0.5 mmol, 1.0 equiv.) and ethyl acrylate **2a** (106.5 µL, 1.0 mmol, 2.0 equiv.). The crude product was purified by flash column chromatography over silica gel (pentane/ethyl acetate 95:5) to give *ethyl (2E)-5-((R)-4-(acetoxymethyl)cyclohex-3-en-1-yl)hexa-2,4-dienoate* **3ca** as a colorless oil (87.7 mg, 60%). **^{1}H NMR** (300 MHz, $CDCl_3$): δ (ppm) 7.59 (dd, *J* = 15.2, 11.5 Hz, 1H, H-11), 6.00 (d, *J* = 12.8 Hz, 1H, H-9), 5.81-5.75 (m, 2H, H-12 and H-2), 4.45 (br. s, 2H, H-7), 4.18 (q, *J* = 7.1 Hz, 2H, H-14), 2.30-1.79 (m, 13H, H-3, H-4, H-5, H-6, H-10, H-17), 1.27 (t, *J* = 7.1 Hz, 3H, H-15). **^{13}C{^{1}H} NMR** (75 MHz, $CDCl_3$): δ (ppm) 171.12 (C-16 minor isomer), 171.09 (C-16 major isomer), 167.8 (C-13 minor isomer), 167.7 (C-13 major isomer), 153.12 (C-1 major isomer), 153.05 (C-1 minor isomer), 141.0 (C-11 major isomer), 139.7 (C-11 minor isomer) 133.0 (C-8 minor isomer), 132.9 (C-8 major isomer), 125.7 (C-9 minor isomer), 125.4 (C-9 major isomer), 124.2 (C-2 minor isomer), 122.2 (C-2 major isomer), 119.68 (C-12 major isomer), 119.61 (C-12 minor isomer), 68.6 (C-7 minor isomer), 68.4 (C-7 major isomer), 60.32 (C-14 minor isomer), 60.26 (C-14 major isomer), 43.4 (C-4 major isomer), 36.2 (C-4 minor isomer), 30.2 (C-6 major isomer), 29.8 (C-3 or C-6 minor isomer), 29.7 (C-3 or C-6 minor isomer), 27.1 (C-5 major isomer), 26.9 (C-5 minor isomer), 26.4 (C-3 major isomer), 21.1 (C-17 major isomer), 20.3 (C-17 minor isomer), 15.6 (C-10 both isomers), 14.5 (C-15 both isomers). **IR (ATR)** ν (cm^{-1}): 2926, 1739, 1712, 1633, 1228, 1135, 1026. **HRMS (ESI)** m/z: $[M+H]^+$ calcd for $C_{17}H_{24}O_4H$ 293.1747, found 293.1748.

Ethyl (2*E*)-5-((2*R*,8*R*,8a*S*)-8,8a-dimethyl-1,2,3,4,6,7,8,8a-octahydronaphthalen-2-yl)hexa-2,4-dienoate (**3da**)

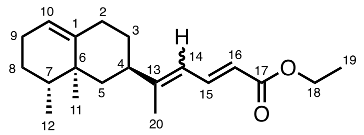

3da

Prepared according to the general procedure **B** from (+)-valencene **1d** (111.1 µL, 0.5 mmol, 1.0 equiv.) and ethyl acrylate **2a** (106.5 µL, 1.0 mmol, 2.0 equiv.). The crude product was purified by flash column chromatography over silica gel (pentane/ethyl acetate 99:1) to give *ethyl (2E)-5-((2R,8R,8aS)-8,8a-dimethyl-1,2,3,4,6,7,8,8a-octahydronaphthalen-2-yl)hexa-2,4-dienoate* **3da** as a colorless oil (84.6 mg, 56%). **^{1}H NMR** (400 MHz, $CDCl_3$): δ (ppm) 7.54 (dd, *J* = 15.1, 11.6 Hz, 1H, H-15), 6.01 (d, *J* = 12.8 Hz, 1H, H-14), 5.78 (d, *J* = 15.2 Hz, 1H, H-16), 4.83 (td, *J* = 11.0, 4.5 Hz, 1H, H-10), 4.19 (q, *J* = 7.1 Hz, 2H, H-18), 2.18-0.92 (m, 24H, H-2, H-3, H-4, H-5, H-7, H-8, H-9, H-11, H-12, H-19, H-20). **^{13}C{^{1}H} NMR** (101 MHz, $CDCl_3$): δ (ppm) 167.8 (C-17 both isomers), 154.4 (C-1 both isomers), 142.6 (C-13 both isomers), 141.3 (C-15 both isomers), 121.9 (C-14 both isomers), 120.7 (C-10 both isomers), 119.2 (C-16 both isomers), 60.2 (C-18 both isomers), 44.5 (C-5 both isomers), 43.6 (C-4 or C-7 both isomers), 41.1 (C-4 or C-7 both isomers), 38.0 (C-6 both isomers), 32.7 (C-2 or C-3 both isomers), 32.6 (C-2 or C-3 both isomers), 27.2 (C-8 or C-9 both isomers), 26.0 (C-8 or C-9 both isomers), 18.5 (C-20 both isomers), 15.8 (C-11 or C-12 both isomers), 15.8 (C-11 or C-12 both isomers), 14.5 (C-19 both isomers). **IR (ATR)** ν (cm^{-1}): 2966, 2923, 1714, 1632, 1369, 1306, 1268, 1214, 1148, 1134, 1096, 1045, 979, 887, 844, 809, 732. **HRMS (ESI)** m/z: $[M+H]^+$ calcd for $C_{20}H_{30}O_2H$ 303.2319, found 303.2320.

Ethyl 4-((1*R*,5*S*)-6,6-dimethylbicyclo[3.1.1]heptan-2-ylidene)but-2-enoate (**3ea**)

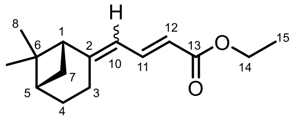

3ea

Prepared according to the general procedure **B** from (-)-β-pinene **1e** (78.3 µL, 0.5 mmol, 1.0 equiv.) and ethyl acrylate **2a** (106.5 µL, 1.0 mmol, 2.0 equiv.). The crude product was purified by flash column chromatography over silica gel (pentane/ethyl acetate 99:1) to give *ethyl 4-((1R,5S)-6,6-dimethylbicyclo[3.1.1]heptan-2-ylidene)but-2-enoate* **3ea** as a colorless oil (64.4 mg, 55%). **^{1}H NMR** (400 MHz, $CDCl_3$): δ (ppm) 7.56 (dd, *J* = 15.2, 11.8 Hz, 1H, H-11), 5.80 (d, *J* = 12.1 Hz, 1H, H-10), 5.69 (d, *J* = 15.2 Hz, 1H, H-12), 4.22-4.10 (m, 2H, H-14), 2.76-1.87 (m, 6H, H-1, H-3, H-4, H-5), 1.44-1.33 (m, 2H, H-7), 1.27 (m, 9H, H-8, H-9, H-15). **^{13}C{^{1}H} NMR** (101 MHz, $CDCl_3$): δ (ppm) 168.1 (C-13 minor isomer), 167.9 (C-13 major isomer), 158.2 (C-2 minor isomer), 158.0 (C-2 major isomer), 140.5 (C-11 major isomer), 140.3 (C-11 minor isomer), 121. 1 (C-10 minor isomer), 120.6 (C-10 major isomer), 117.8 (C-12 major isomer), 117.6 (C-12 minor isomer), 60.13 (C-14 major isomer), 60.09 (C-14 minor isomer), 53.64 (C-1 minor isomer), 53.55 (C-1 minor isomer), 41.8 (C-6 minor isomer), 41.6 (C-6 major isomer), 40.8 (C-5 minor isomer), 40.6 (C-5 major isomer), 27.4 (C-7 major isomer), 26.33 (C-7 minor isomer), 26.27 (C-3 or C-4 minor isomer), 26.22 (C-8 or C-9 minor isomer), 26.15 (C-8 or C-9 major isomer), 23.9 (C-3 or C-4 minor isomer), 23.6 (C-3 or C-4 major isomer), 22.1 (C-8 or C-9 major isomer), 22.0 (C-8 or C-9 minor isomer), 20.7 (C-3 or C-4 major isomer), 14.5 (C-15 major isomer), 14.3 (C-15 minor isomer). **IR (ATR)** ν (cm^{-1}): 2924, 1712, 1630, 1369, 1309, 1269, 1211, 1202, 1157, 1143, 1127, 1097 1042, 981, 913. **HRMS (ESI)** m/z: $[M+H]^+$ calcd for $C_{15}H_{22}O_2H$ 235.1693, found 235.1694.

Ethyl (2*E*)-5-((1*S*,2*R*,4*R*)-2-acetoxy-4-methylcyclohexyl)hexa-2,4-dienoate (**3fa**)

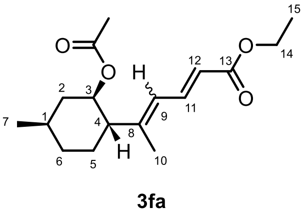

3fa

Prepared according to the general procedure **B** from (-)-isopulegol acetate **1f** (104.5 µL, 0.5 mmol, 1.0 equiv.) and ethyl acrylate **2a** (106.5 µL, 1.0 mmol, 2.0 equiv.). The crude product was purified by flash column chromatography over silica gel (pentane/ethyl acetate 95:5) to give *ethyl (2E)-5-((1S,2R,4R)-2-acetoxy-4-methylcyclohexyl)hexa-2,4-dienoate* **3fa** as a colorless oil (46.5 mg, 32%). **^{1}H NMR** (400 MHz, $CDCl_3$): δ (ppm) 7.55 (dd, *J* = 15.2, 11.5 Hz, 1H, H-11), 6.01 (d, *J* = 11.5 Hz, 1H, H-9), 5.78 (d, *J* = 15.1 Hz, 1H, H-12), 4.84 (td, 1H, H-3), 4.25 (m, 2H, H-14), 2.16 (m, 1H, H-4), 1.92 (s, 3H, H-10 or H-17), 1.81 (s, 3H, H-10 or H-17), 1,77-1.35 (m, 7H, H-1, H-2, H-5, H-6), 1.28 (t, *J* = 7.2 Hz, 3H, H-15), 0.93 (d, J = 6.4 Hz, 3H, H-7). **^{13}C{^{1}H} NMR** (101 MHz, $CDCl_3$): δ (ppm) 170.5 (C-16 both isomers), 167.7 (C-17 both isomers), 149.8 (C-8 both isomers), 140.6 (C-11 both isomers), 124.9 (C-9 both isomers), 119.9 (C-12 both isomers), 73.2 (C-3 both isomers), 60.3 (C-14 both isomers), 53.2 (C-4 both isomers), 40.5 (C-2 both isomers), 34.0 (C-6 both isomers), 31.5 (C-1 both isomers), 29.9 (C-5 both isomers), 22.1 (C-7 both isomers), 21.2 (C-17 both isomers), 14.6 (C-10 both isomers), 14.5 (C-15 both

isomers). **IR (ATR)** ν (cm^{-1}): 2928, 2360, 2341, 1736, 1714, 1633, 1368, 1243, 1160, 1928. **HRMS (ESI)** m/z: [M+H]$^+$ calcd for $C_{17}H_{26}O_4H$ 295.1904, found 295.1905.

<u>(2*E*)-*N*-methyl-5-((*S*)-4-methylcyclohex-3-en-1-yl)-*N*-((*S*)-1-phenylethyl)hexa-2,4-dienamide (**3*ent*-ao**)</u>

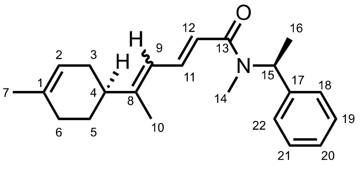

3*ent*-ao

Prepared according to the general procedure **B** from (-)-limonene ***ent*-1a** (81.0 µL, 0.5 mmol, 1.0 equiv.) and (*S*)-*N*-methyl-*N*-(1-phenylethyl)acrylamide **2o** (189.3 mg, 1.0 mmol, 2.0 equiv.). The crude product was purified by flash column chromatography over silica gel (pentane/ethyl acetate 3:7) to give *(2E)-N-methyl-5-((S)-4-methylcyclohex-3-en-1-yl)-N-((S)-1-phenylethyl)hexa-2,4-dienamide* **3ent-ao** as a colorless oil (69.5 mg, 43%). **^{1}H NMR** (400 MHz, $CDCl_3$): **^{1}H NMR** (400 MHz, $CDCl_3$): 7.72 (m, 1H, H-11), 7.40-7.19 (m, 5H, H-18, H-19, H-20, H-21, H-22), 6.27-5.99 (m, 2H, H-9 and H-12), 5.40 (br. s, 1H, H-2), 2.72 (s, 3H, H-14), 2.34-1.19 (m, 17H, H-3, H-4, H-5, H-6, H-7, H-10, H-15, H-16).**^{13}C{^{1}H} NMR** (101 MHz, $CDCl_3$): δ (ppm) 167.4 (C-13 both isomers), 152.5 (C-1 both isomers), 140.9 (C-11 both isomers), 139.8 (C-17 both isomers), 134.0 (C-8 both isomers), 128.5 (C-9 both isomers), 127.4 (C-2 both isomers), 122.3 (2C, C-18 and C-22 both isomers), 120.5 (C-12 both isomers), 120.4 (2C, C-19 and C-21 both isomers), 119.0 (C-20 both isomers), 50.6 (C-15 both isomers), 43.6 (C-4 both isomers), 30.5 (2C, C-3 and C-6 both isomers), 29.7 (C-14 both isomers), 27.8 (C-5 both isomers), 23.6 (C-7 both isomers), 22.5 (C-16 both isomers), 15.7 (C-10 both isomers). **IR (ATR)** ν (cm^{-1}): 3012, 2933, 1632, 1591, 1497, 1113, 977, 845. **HRMS (ESI)** m/z: [M+H]$^+$ calcd for $C_{22}H_{29}NOH$ 324.2321, found 324.2320.

7. Mechanistic hypotheses for the first palladation

Before performing the DFT studies, three mechanistic hypotheses relative to the first palladation event were initially considered. The first one involved a concerted metalation deprotonation (CMD) mechanism on a vinylic H atom. A second one implicates an acetoxypalladation of the double bond. Finally, a third hypothesis entailed a CMD on an allylic H atom of the isopropenyl substituent (Scheme S2). The DFT calculation validated the first path.

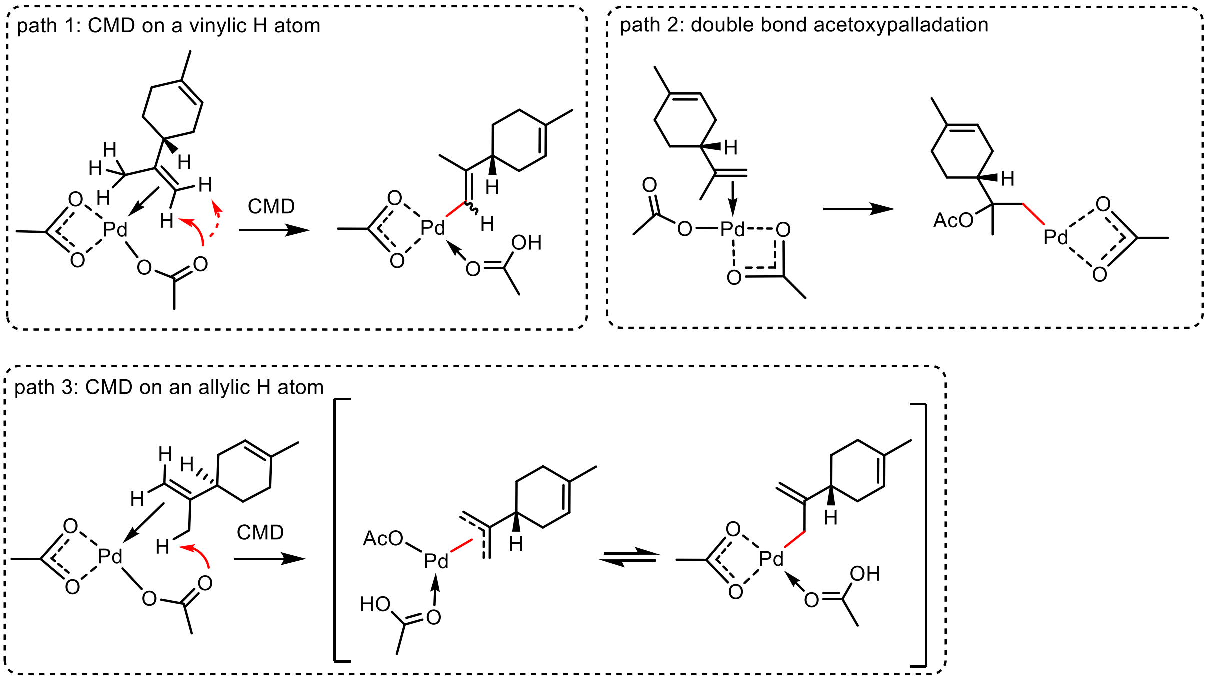

Scheme S2. Mechanistic hypotheses relative to the fist palladation event.

8. *Mechanistic study*

Synthesis of (*Z*)-methyl cinnamate

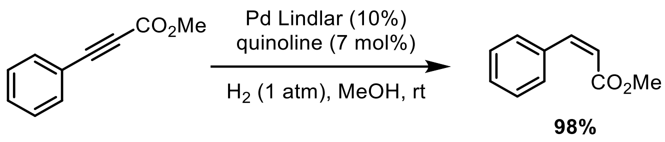

(*Z*)-methyl cinnamate was prepared according to a procedure reported in the literature.[10]
A round bottom flask was charged with Pd Lindlar (660 mg, 6.24 mmol, 1.0 equiv.), quinoline (52 µL, 0.44 mmol, 7 mol%) and methanol (20 mL, 0.3M) was added. To this solution was introduced methyl phenylpropiolate (0.92 mL, 6.24 mmol, 1.0 equiv.) and hydrogen gas was bubbled inside the solution for 10 minutes. The reaction was then stirred overnight at rt under a hydrogen atmosphere. The mixture was then diluted with 100 mL of dichloromethane and filtered over a pad of Celite®. The filtrate was concentrated under reduced pressure. The crude residue was then purified by flash column chromatography (cyclohexane/ethyl acetate 20:1) to afford (*Z*)-methyl cinnamate (985 mg, 98%) as a colorless oil.
^{1}H NMR (400 MHz, $CDCl_3$): δ (ppm) 7.72-7.47 (m, 2H), 7.47-7.28 (m, 3H), 6.96 (d, *J* = 12.6 Hz, 1H), 5.96 (d, *J* = 12.7 Hz, 1H), 3.72 (s, 3H).

Partial isomerization of *Z* methyl cinnamate to E methyl cinnamate in the presence of Mizoroki-Heck reaction conditions.

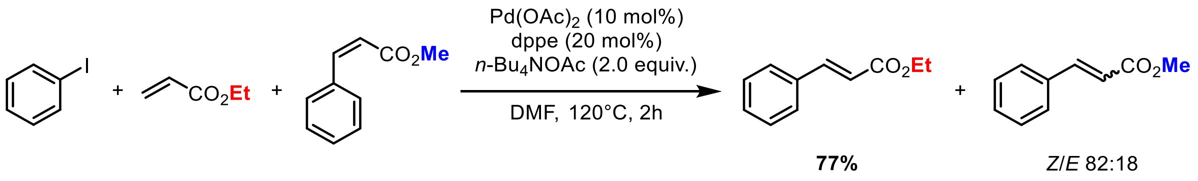

The reaction conditions were taken from a procedure reported in the literature.[11]
An ace pressure tube was charged with palladium(II) acetate (11.2 mg, 0.05 mmol, 10 mol%), dppe (39.8 mg, 0.1 mmol, 20 mol%), (Z)-methyl cinnamate (81.1 mg, 0.5 mmol, 1.0 equiv.) and tetra-*n*-butylammonium acetate (301.5 mg, 1.0 mmol, 2.0 equiv.). The tube was placed under vacuum before being backfilled with argon. The vacuum/argon cycles were repeated twice and anhydrous DMF (6 mL, 0.08M) was added. To this solution was introduced iodobenzene (72 µL, 0.5 mmol, 1.0 equiv.) and ethyl acrylate (53 µL, 0.65 mmol, 1.3 equiv.). The tube was sealed and the mixture was stirred at 120°C until complete conversion as checked by TLC (2h). The mixture was diluted with 25 mL of ethyl acetate and filtered over a pad of silica gel. The filtrate was concentrated under reduced pressure and the crude mixture was analyzed by quantitative ^{1}H-NMR using 1,4-dinitrobenzene (8.4 mg, 0.05 mmol, integration I = 4.0) as internal standard. The results are summarized in Table S4.

Table S4. Data for the *Z-to-E* isomerization of methyl cinnamate

molecule	Integration	n (mmol)	ratio
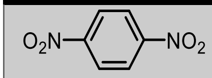 internal standard	4.00 (4H)	0.05	-
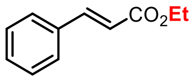	15.41 (2H)	0.385	50%
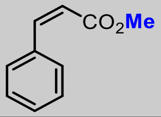	18.72 (3H)	0.312	41%

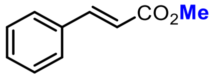	4.21 (3H)	0.070	9%

The *Z*-to-*E* isomerization of methyl cinnamate, although partial, is ostensibly due to the Pd(0)/AcOH system, in accordance with the reversibility of the dehydropalladation / reductive elimination sequence.

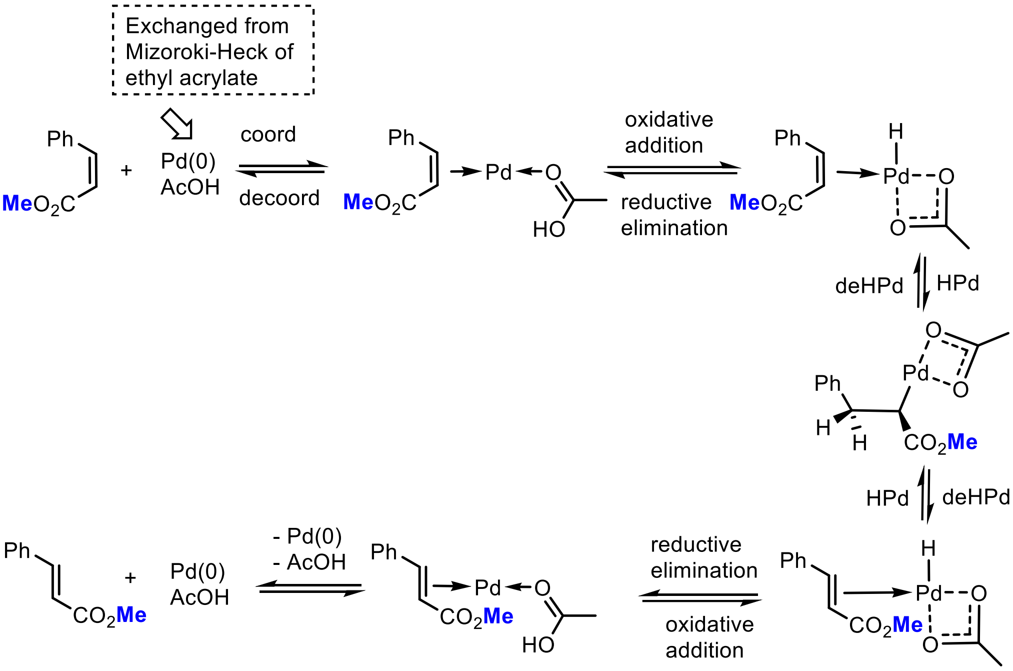

Scheme S3. Proposed mechanism for the Z/E equilibration of Z methyl cinnamate during the Mizoroki-Heck coupling.

9. *Post-functionalization: Cu-free Sonogashira cross-coupling*

The Sonogashira coupling between **3ap** and phenyl acetylene under micellar catalysis was studied. To find out the optimum reaction conditions for the desired coupled product, we carried out a comparative study using three different reported Sonogashira coupling (Scheme S4). A first one based on the catalytic system [Pd(MeCN)$_2$Cl$_2$ (1.0 mol%), XPhos (2.5 mol%), Et$_3$N (2.0 equiv.), TPGS-750-M (3 wt%)] (protocol A); a second one based on the catalytic system [CataCXium A Pd G3 (0.30 mol%), Et$_3$N (3.0 equiv.), TPGS-750-M (2 wt%), H_2O, glucose (5 mol%), THF (15 vol%)] (protocol B), and a third one based on [Pd(MeCN)$_2$Cl$_2$ (1.0 mol%), XPhos (1.3 mol%), Et$_3$N (2.0 equiv.), HPMC 40-60 cPs[a] (2 wt%)] (protocol C). In the event, system A gave, after a 24h reaction time, the desired product **4** in a 24% yield, while system B gave 54%, again after a 24h reaction. Incorporation of THF (15 vol%), to enhance reaction homogeneity, and the addition of glucose (5 mol%), to sustain catalytic efficiency, were crucial for the better result of this protocol with respect to the first one.[12]
When applying conditions C, the desired coupled product **4** was obtained with a notable yield of 48% after an overnight reaction. It is worth noting that these reactions typically exhibit remarkable rapidity, frequently concluding within a timeframe ranging from 2 minutes to 2 hours. Consequently, the observed plateau in reaction progress after an overnight duration can be attributed to the inherently rapid kinetics characterizing these transformations.
Considering the cost-effectiveness of the polymer relative to the surfactant and the demonstrated efficacy of condition B, we have chosen the latter as the starting reaction conditions for the beginning of our optimization studies.

---

[a] "cP" stands for "centipoise", which is a unit to define the viscosity of a fluid.

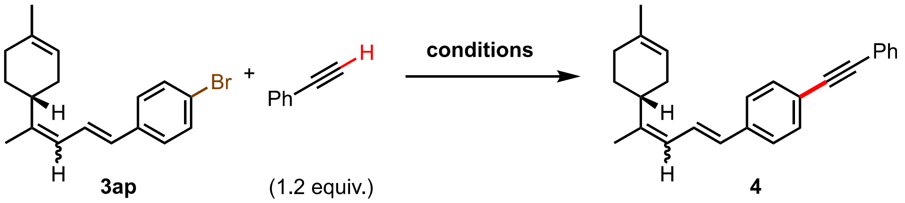

condition A 24%
condition B 54%
condition C 48%

conditions A: $Pd(MeCN)_2Cl_2$ (1.0 mol%), XPhos (2.5 mol%), Et_3N (2.0 equiv.), TPGS-750-M (3 wt%), H_2O, rt, 24h
conditions B: CataCXium A Pd G_3 (0.30 mol%), Et_3N (3.0 equiv.), TPGS-750-M (2 wt%), H_2O, glucose (5 mol%), THF (15 vol%), 45°C, 24h
conditions C: $Pd(MeCN)_2Cl_2$ (1.0 mol%), XPhos (1.3 mol%), Et_3N (2.0 equiv.), HPMC 40-60 cps (2 wt%), rt, overnight

Scheme S4. Sonogashira cross-coupling between the limonene-derived aryl bromide **3ap** and phenyl acetylene: three protocols compared.

Our initial investigations involved a systematic examination of the catalytic loadings (Table S5). Although 0.3 mol% (Table S5, entry 1) of the palladium catalyst emerged as the most favorable compromise among all loadings because compound **4** was obtained with 34% and 62% yield respectively with 0.1 and 0.5 mol% (Table S5, entries 2-3), we have selected a catalytic loading of 0.1 mol% for further optimizing the reaction conditions. The decision to retain a catalytic loading of 0.1 mol% is motivated by cost considerations. This choice provides an economically viable starting point for our optimization efforts. As we refine the reaction conditions, we may explore the possibility of increasing the catalytic loading, especially during the scale-up of the reaction, to assess its impact on performance.

Table S5. Screening of the catalytic loading of the palladium catalyst.

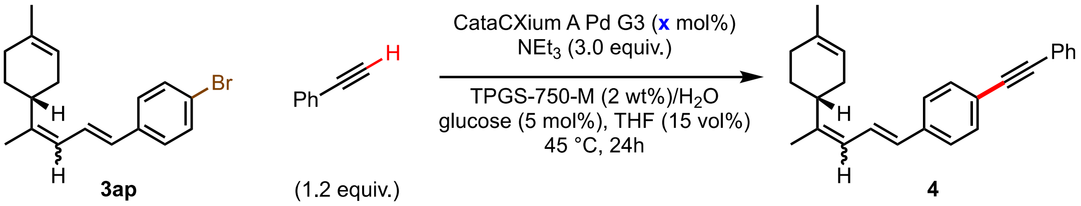

Entry[a]	CataCXium A Pd G3 (x mol%)	4 (%)[b]
**1**	0.30	54
**2**	0.10	34
**3**	0.50	62

[a] typical reaction conditions: **3ap** (0.1 mmol), phenyl acetylene (1.2 equiv.), CataCXium A Pd G3 (**x** mol%), NEt_3 (3.0 equiv.), TPGS-750-M (2 wt%)/H_2O, glucose (5 mol%), THF (15 vol%) at 45 °C in a sealed vial; [b] measured by quantitative ^{1}H-NMR using 1,4-dinitrobenzene as an internal standard.

After the determination of the optimal catalytic loading, our efforts focused on the optimization of reaction conditions (Table S6). Attempts to substitute THF with alternative, more polar co-solvents such as acetone or *tert*-amyl alcohol (Table S6, entry 2 and 3) did not yield any noticeable improvements. Similarly, the exploration of alternative inorganic bases (Table S6, entries 4-5), such as sodium carbonate or cesium carbonate, or the extension of lipophilicity (Table S6, entry 6) within the alkyl chain through the use of *n*-Oct_3N, did not result in enhanced yields.
Interestingly, a substantial increase in yield from 34% to 54% (Table S6, entry 7) was observed upon doubling the concentration of the reaction, prompting our decision to maintain this concentration level for further optimization efforts. Subsequently, we turned our attention to investigating the influence of the temperature

on the reaction outcome. However, neither room temperature (rt) nor a higher temperature of 60°C appeared to significantly enhance reaction performance (Table S6, entries 8-9).

Table S6. Optimization of the reaction conditions.

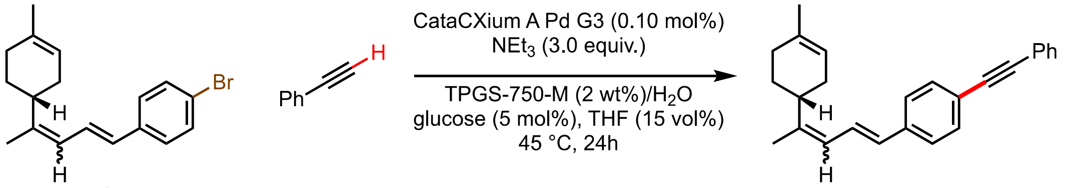

Entry[a]	Deviation from standard conditions	4 (%)[b]
1	None	34
2	acetone instead of THF	12
3	*tert*-amyl alcohol instead of THF	8
4	Na_2CO_3 instead of Et_3N	-[c]
5	Cs_2CO_3 instead of Et_3N	-[c]
6	(*n*-oct)$_3$N instead of Et_3N	12
7	0.4M instead of 0.2M	53
8	rt instead of 45°C	23
9	60°C instead of 45°C	52

[a] typical reaction conditions: **3ap** (0.1 mmol), phenyl acetylene (1.2 equiv.), CataCXium A Pd G3 (0.10 mol%), base (3.0 equiv.), TPGS-750-M (2 wt%)/H_2O, glucose (5 mol%), co-solvent (15 vol%) at T in a sealed vial; [b] measure by quantitative ^{1}H-NMR, using 1,4-dinitrobenzene as an internal standard; [c] not determined.

The desired compound **4** was prepared according to a procedure reported in the literature.[13]

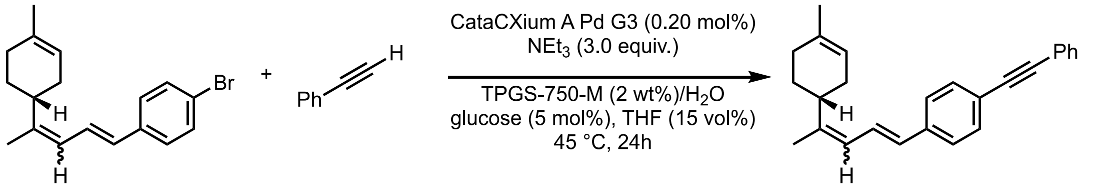

A round bottom flask was charged with 1-bromo-4-((1*E*)-4-((*R*)-4-methylcyclohex-3-en-1-yl)penta-1,3-dien-1-yl)benzene **3ap** (317.3 mg, 1.0 mmol, 1.0 equiv.) and the flask was placed under vacuum before being backfilled with argon. The vacuum/argon cycles were repeated twice and a 2 wt% aqueous solution of TPGS-750-M (3.0 mL) was added. To this solution was introduced a 0.2 mol% of CataCXium A Pd G3 (145 mg) stock solution in THF (15 vol%), 5 mol% glucose (9.9 mg, 0.05 equiv.), 150 µL of THF and ethynylbenzene (131 µL, 1.2 mmol, 1.2 equiv.). The mixture was stirred for 5 min at rt and triethylamine (418 µL, 3.0 mmol, 3.0 equiv.) was added. The reaction mixture was heated up to 45°C and the reaction was stirred for 24 h. The reaction mixture was cooled to rt and transferred into a 5 mL syringe filled with 3 g of silica. The silica was washed subsequently with heptane (2 x 2 mL) and ethyl acetate (2 x 2 mL). The combined organic layers were washed with brine (2 x 1 mL), dried over Na_2SO_4 and the solvents were removed *in vacuo*. Crude materials were purified by liquid-liquid extraction (Heptane/AcOEt 7:1) to afford the desired compound *1-((1E)-4-((R)-4-methylcyclohex-3-en-1-yl)penta-1,3-dien-1-yl)-4-(phenylethynyl)benzene* **4** as a colourless oil (257.3 mg, 76%).

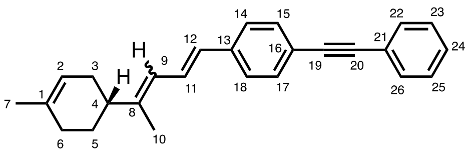

4

^{1}H NMR (400 MHz, $CDCl_3$): δ (ppm) 7.54 (m, 2H, H-22 and H-26), 7.44-7.30 (m, 5H, H-14, H-15, H-17, H-18, H-24), 7.28-7.22 (m, 2H, H-23 and H-25), 7.12-6.89 (m, 1H, H-11), 6.38 (dd, *J* = 15.4, 1H, H-12), 6.14–5.94 (m, 1H, H-9), 5.41 (br. s, 1H, H-2), 2.30-0.81 (m, 13H, H-3, H-4, H-5, H-6, H-7, H-10). **^{13}C{^{1}H} NMR** (75 MHz, $CDCl_3$): δ (ppm) 141.1 (C-1 both isomers), 133.9 (C-8 both isomers), 132.7 (2C, C-15 and C-17 both isomers), 131.8 (2C, C-14 and C-18 both isomers), 129.4 (2C, C-22 and C-26 both isomers), 129.0 (C-21 both isomers), 128.6 (2C, C-23 and C-25 both isomers), 127.7 (C-13 both isomers), 126.6 (C-24 both isomers), 123.5 (C-9 both isomers), 122.0 (C-2 both isomers), 120.9 (C-16 both isomers), 120.7 (C-11 both isomers), 119.5 (C-12 both isomers), 81.7 (C-19 both isomers), 74.1 (C-20 both isomers), 43.8 (C-4 major isomer), 43.5 (C-4 minor isomer), 30.9 (C-3 or C-6 major isomer), 30.87 (C-3 or C-6 major isomer), 30.7 (C-3 and C-6 minor isomers), 28.1 (C-5 major isomer), 27.9 (C-5 minor isomer), 23.8 (C-7 minor isomer), 23.7 (C-7 major isomer), 15.4 (C-10 minor isomer), 14.7 (C-10 major isomer). **IR (ATR)** ν (cm^{-1}): 2978, 1535, 1587, 1427, 1104, 1082, 942, 812, 834, 794. **HRMS (ESI)** m/z: $[M+H]^+$ calcd for $C_{26}H_{26}H$ 339.5020, found 339.5020.

10. ^{1}H and ^{13}C NMR spectra for unknown compounds

^{1}H NMR Spectrum **3aa** (400 MHz, $CDCl_3$)

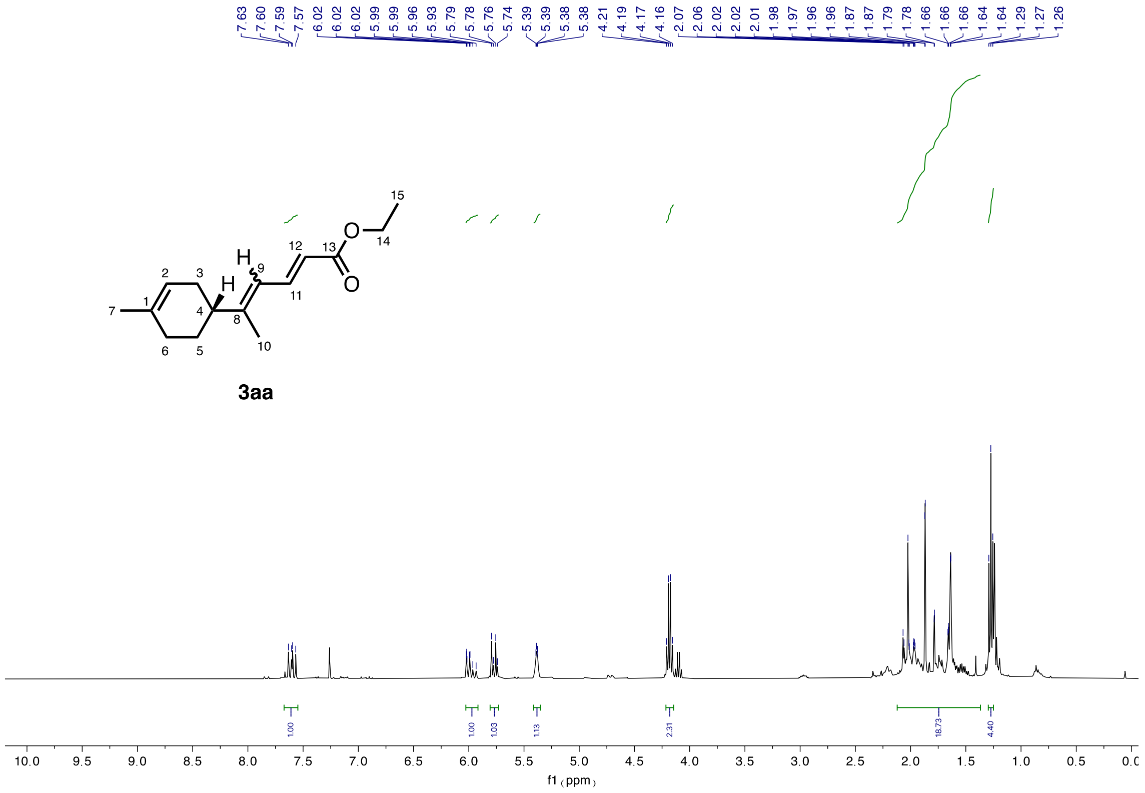

$^{13}C\{^{1}H\}$ NMR Spectrum **3aa** (101 MHz, $CDCl_3$)

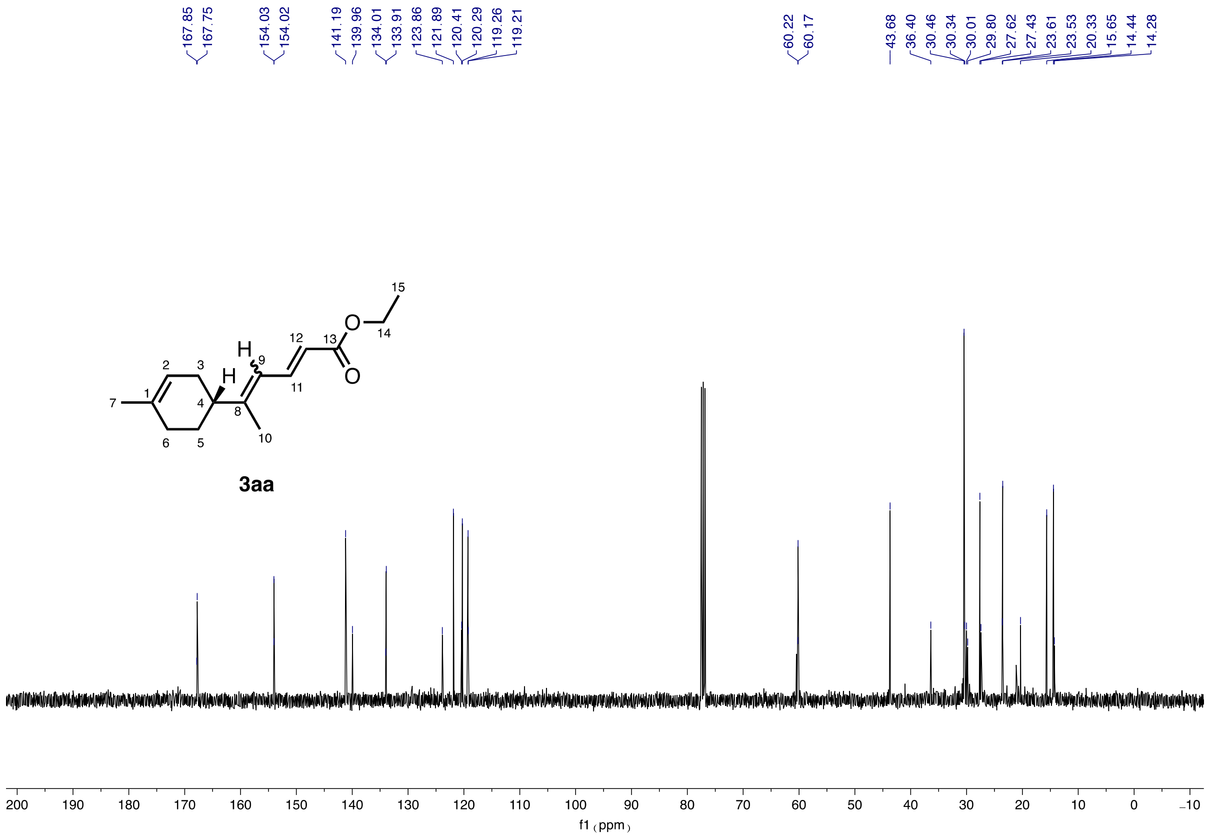

^{1}H NMR Spectrum **3ab** (400 MHz, $CDCl_3$)

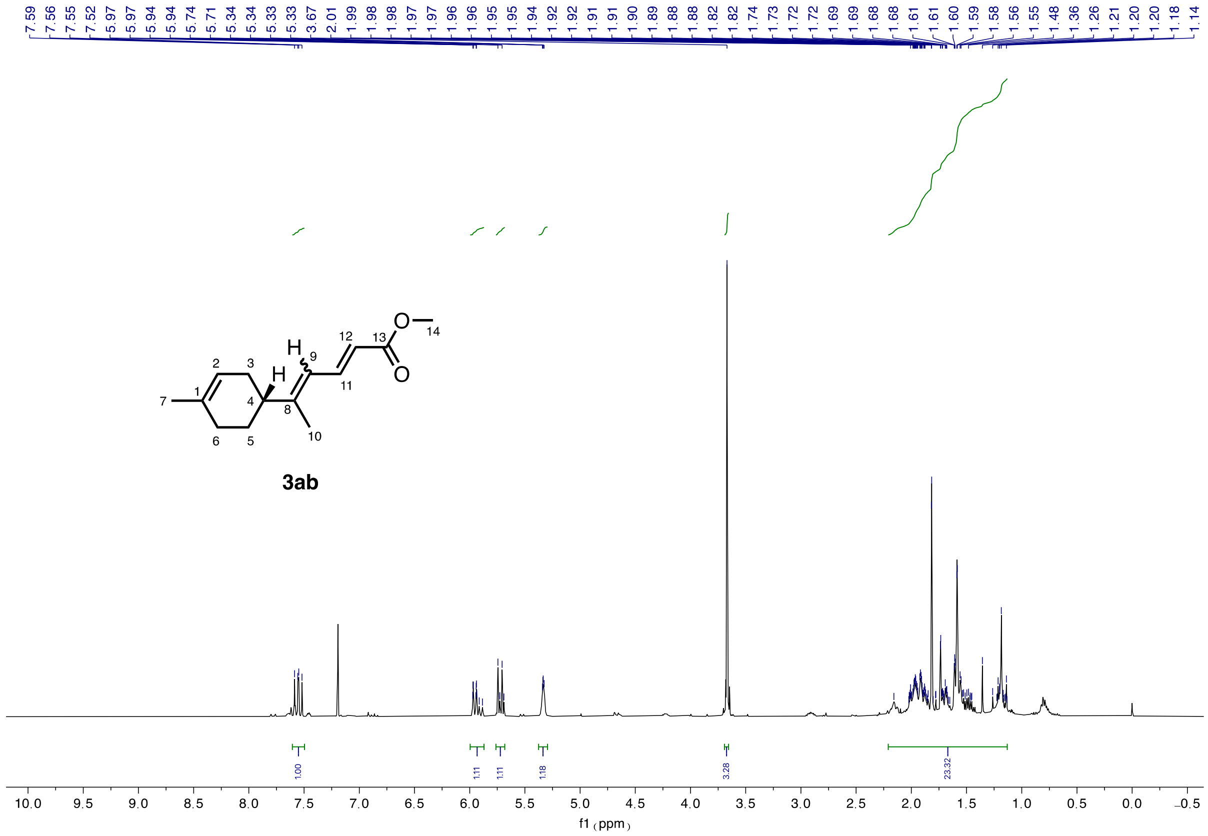

^{13}C{^{1}H} NMR Spectrum **3ab** (101 MHz, $CDCl_3$)

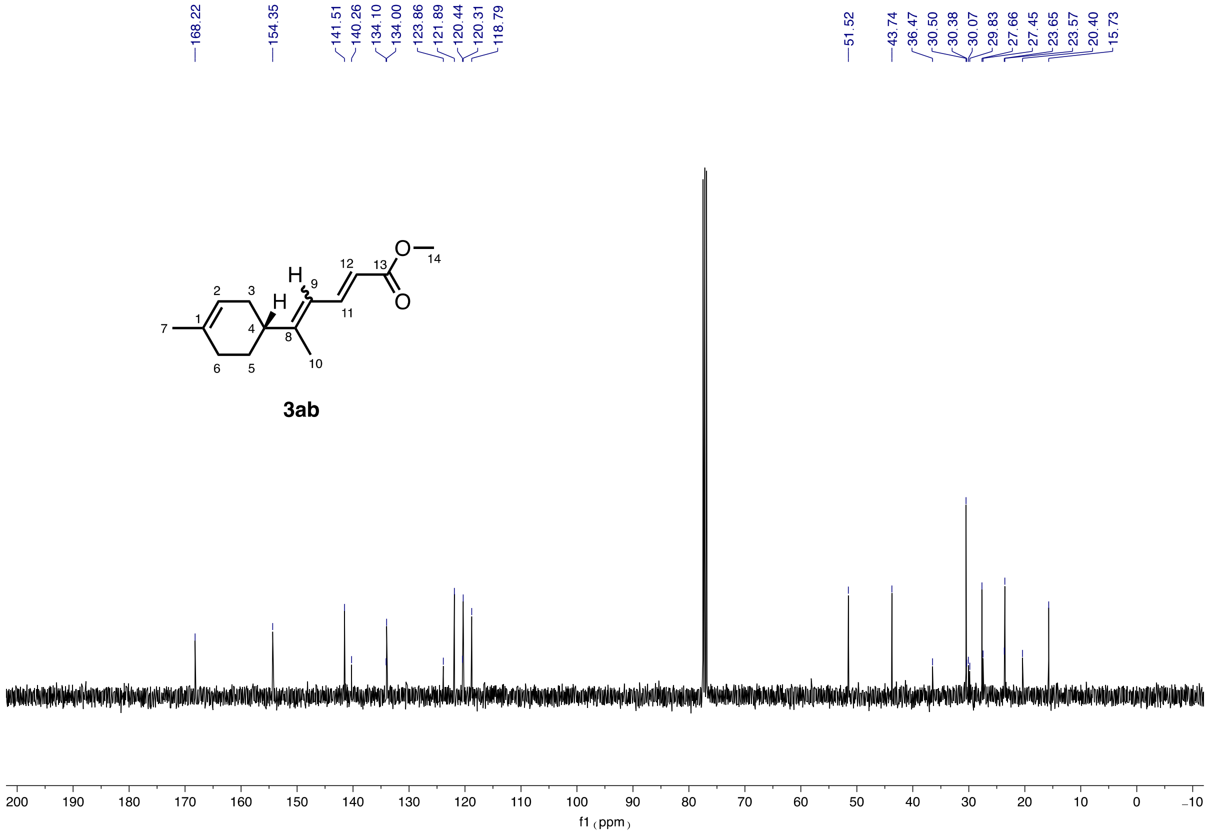

^{1}H NMR Spectrum **3ac** (400 MHz, $CDCl_3$)

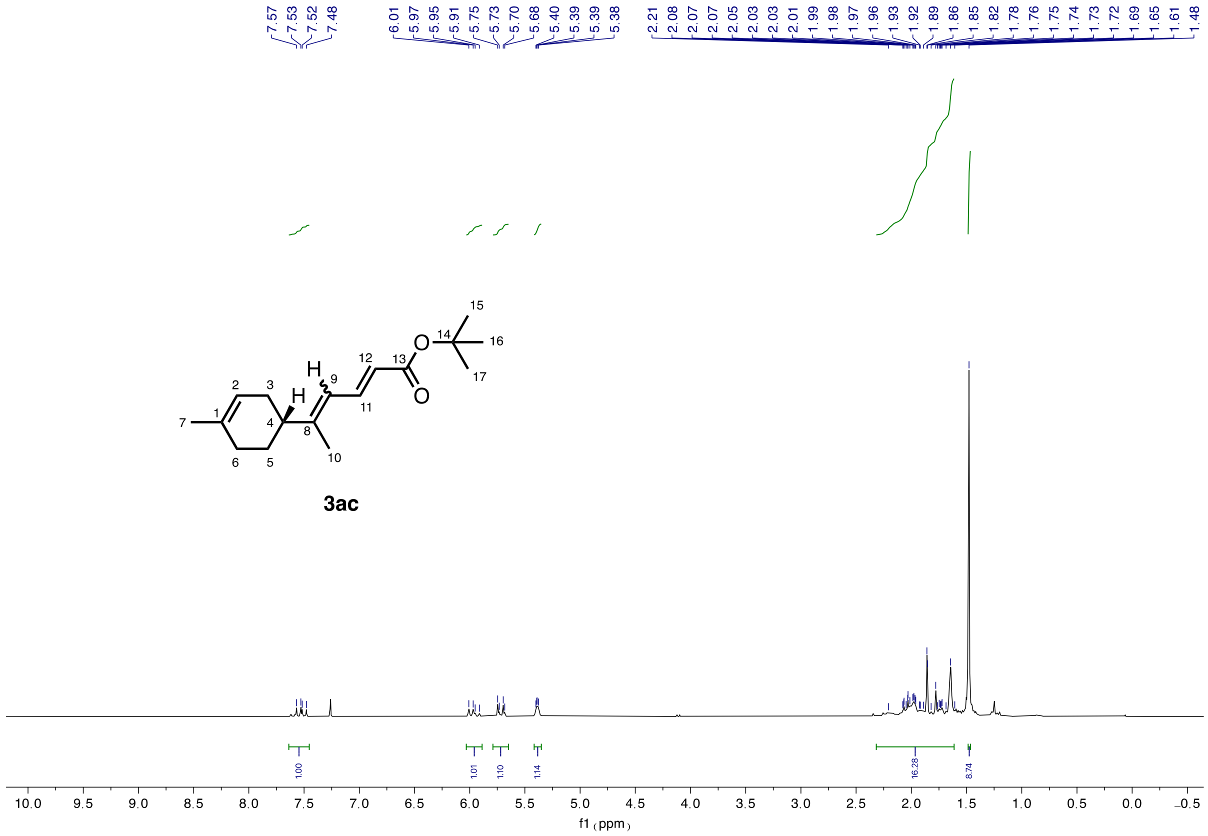

^{13}C{^{1}H} NMR Spectrum **3ac** (75 MHz, $CDCl_3$)

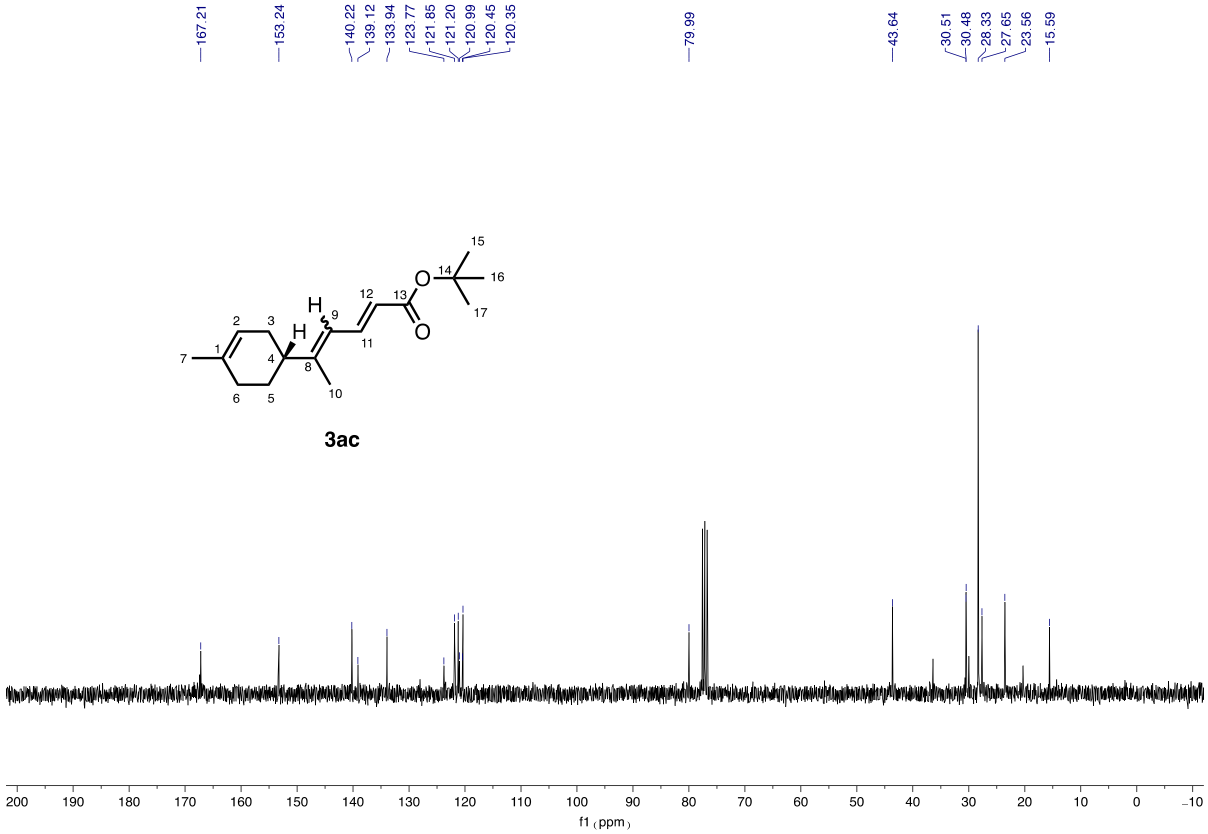

^{1}H NMR Spectrum **3ad** (300 MHz, $CDCl_3$)

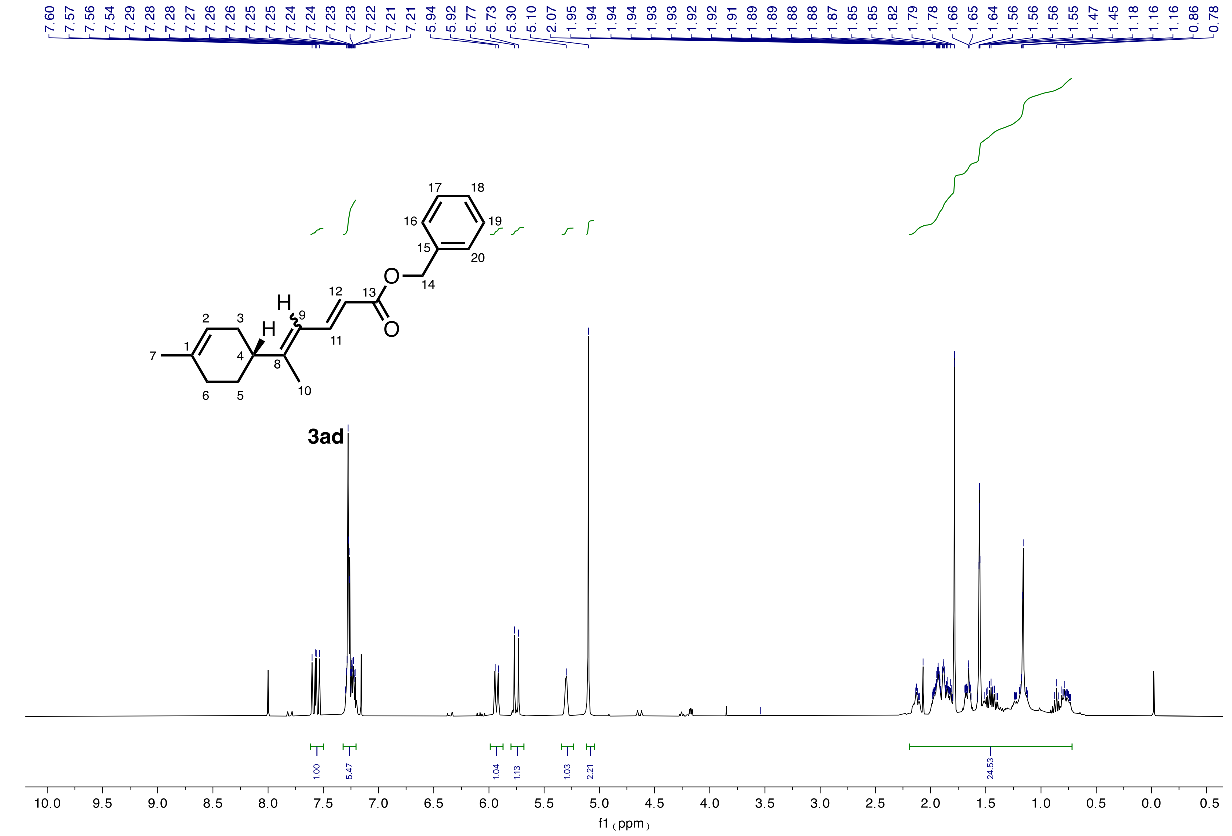

$^{13}C\{^{1}H\}$ NMR Spectrum **3ad** (101 MHz, $CDCl_3$)

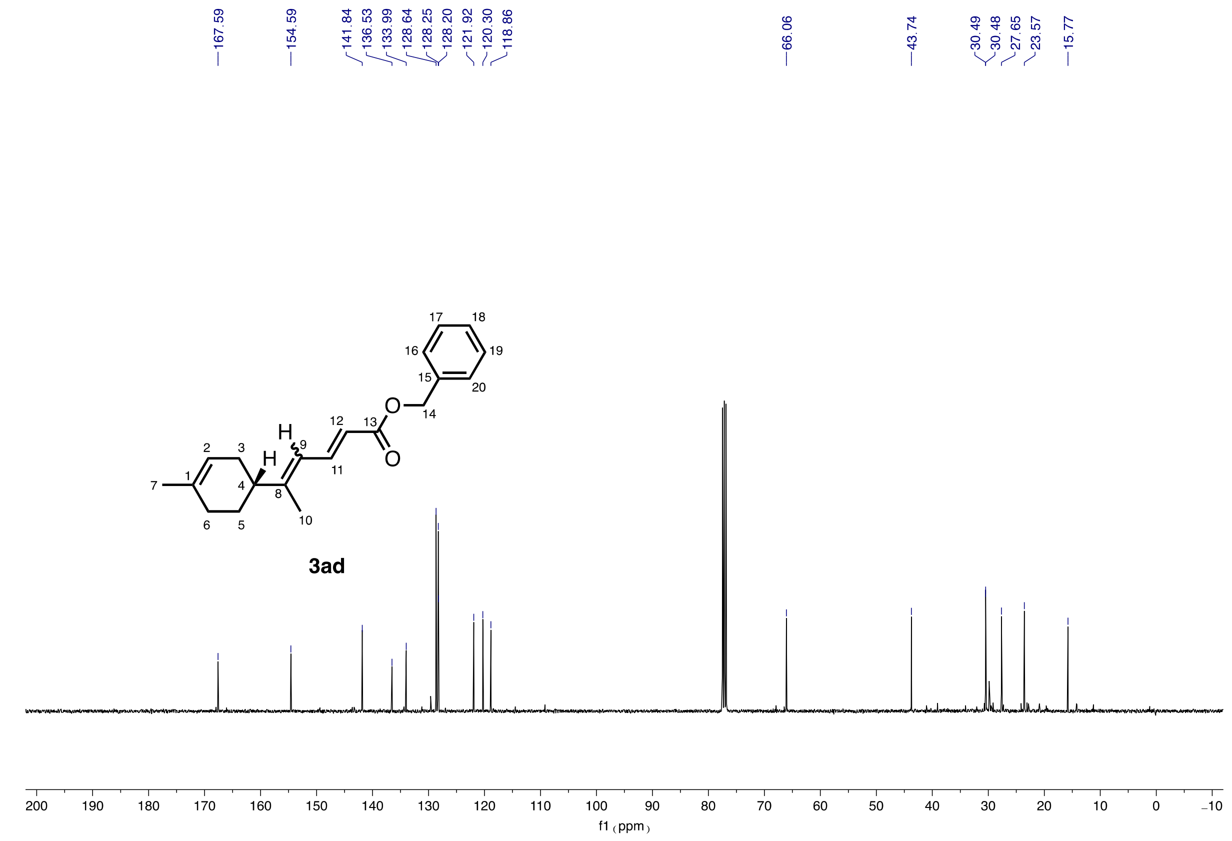

^{1}H NMR Spectrum **3ae** (400 MHz, $CDCl_3$)

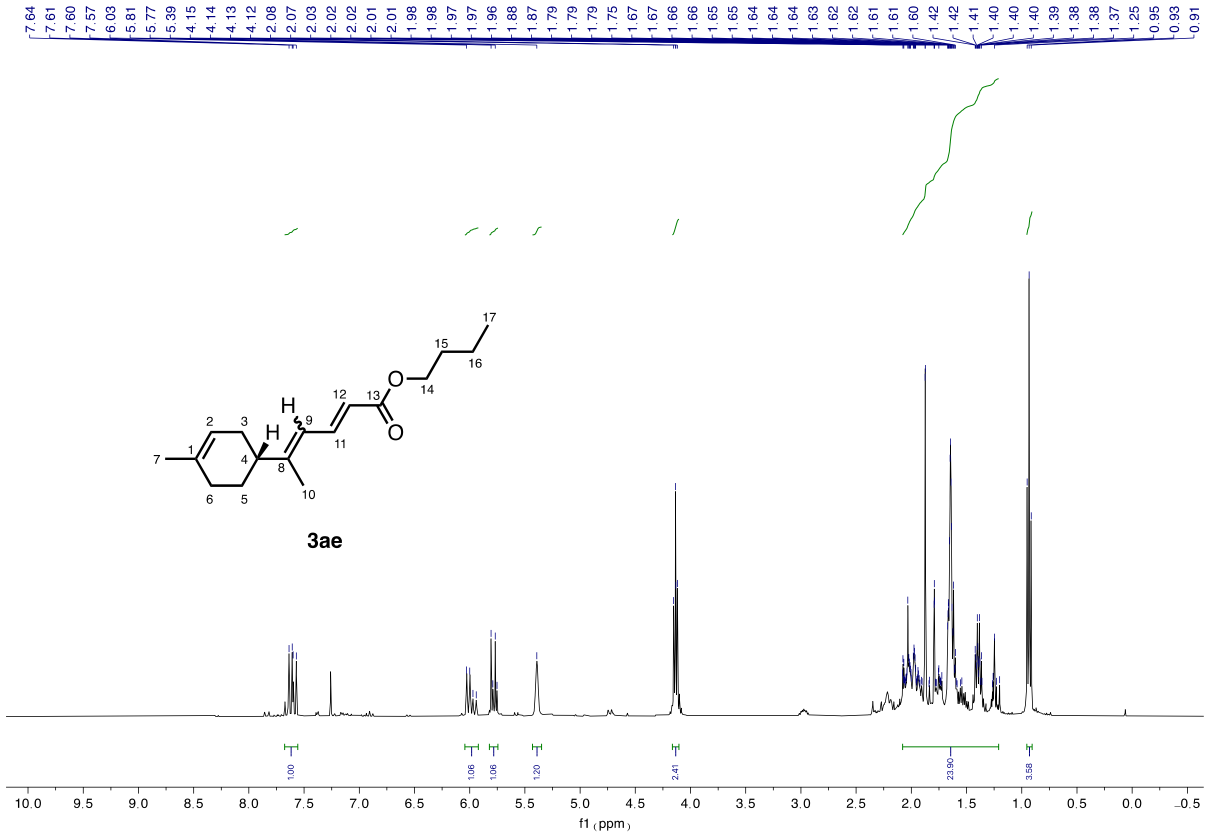

^{13}C{^{1}H} NMR Spectrum **3ae** (101 MHz, $CDCl_3$)

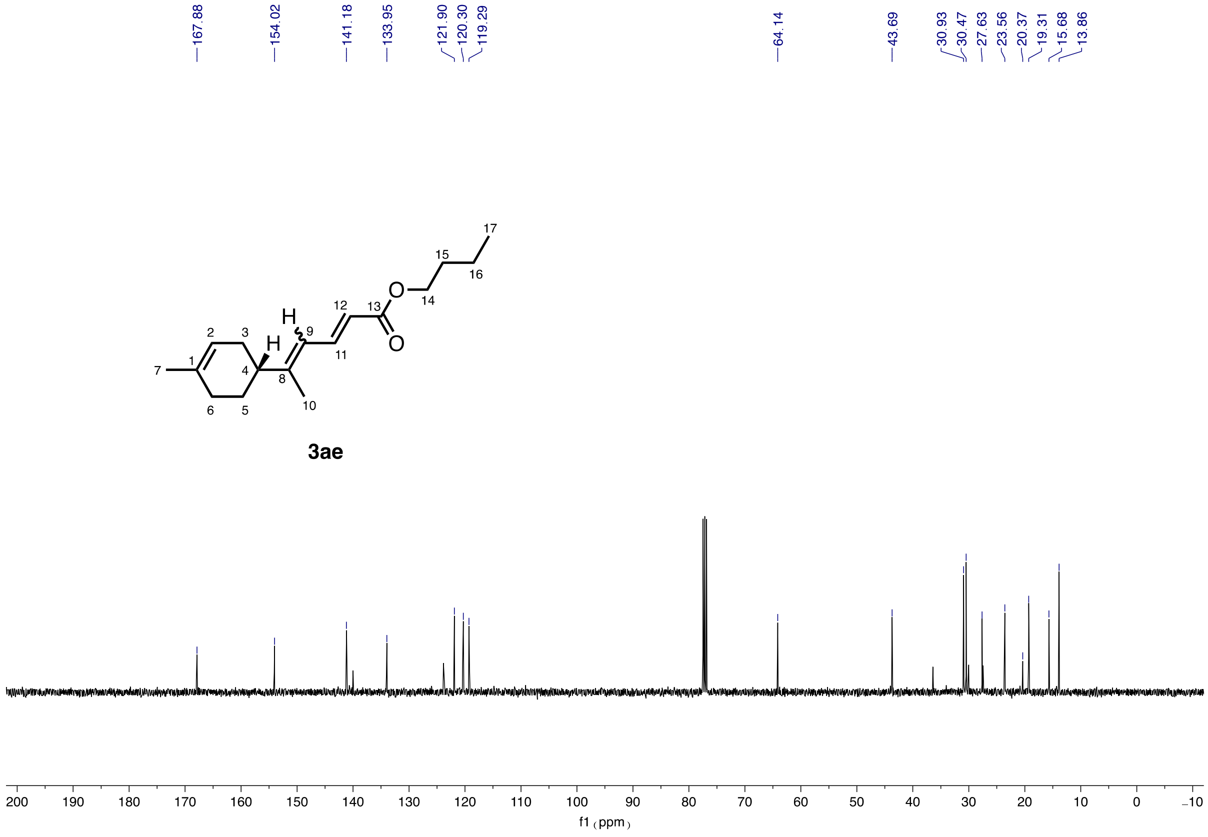

^{1}H NMR Spectrum **3af** (400 MHz, $CDCl_3$)

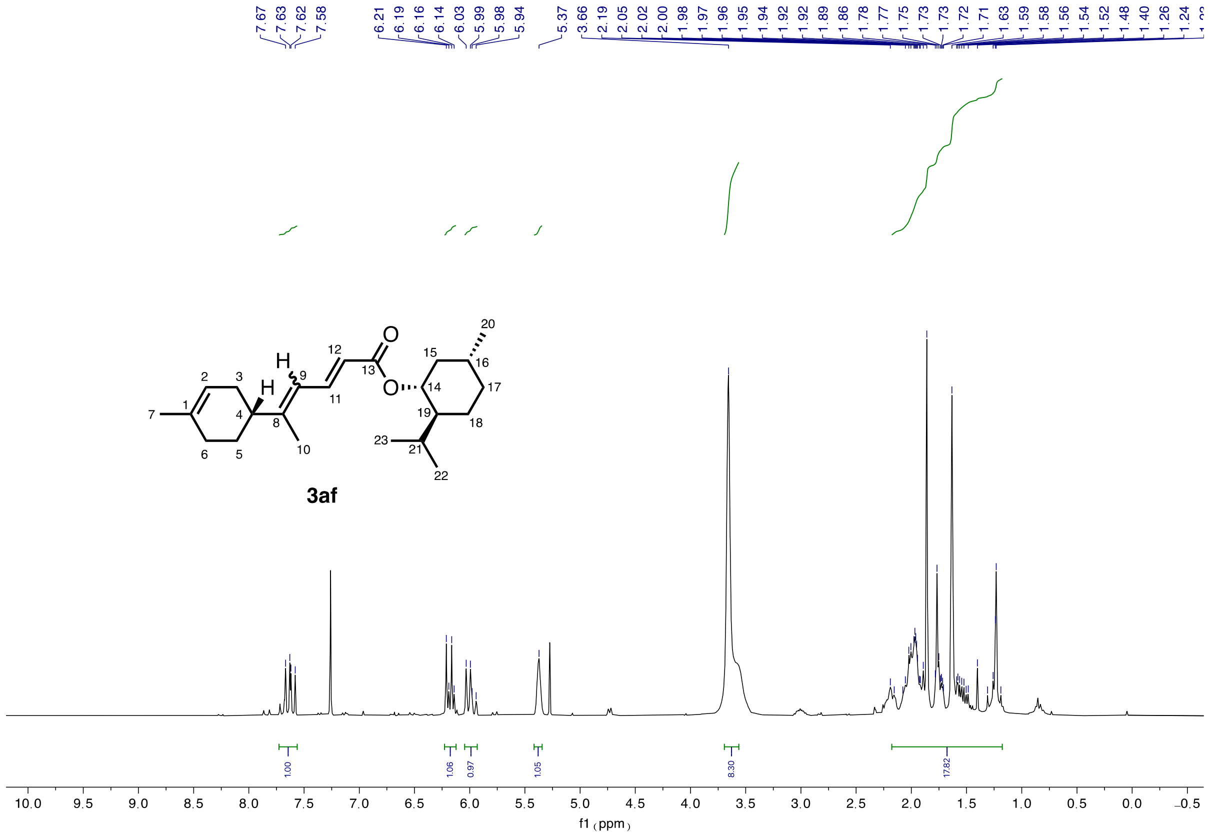

$^{13}C\{^{1}H\}$ NMR Spectrum **3af** (101 MHz, $CDCl_3$)

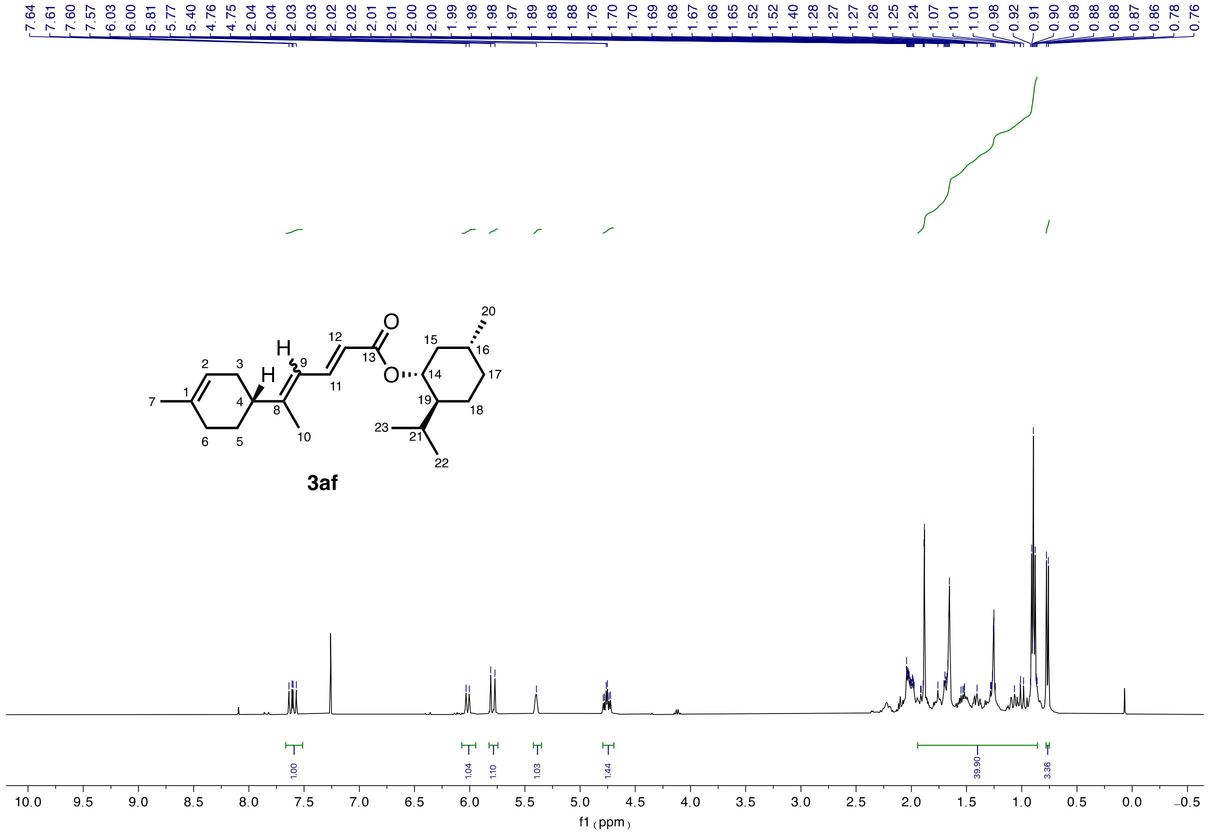

^{1}H NMR Spectrum **3ag** (400 MHz, $CDCl_3$)

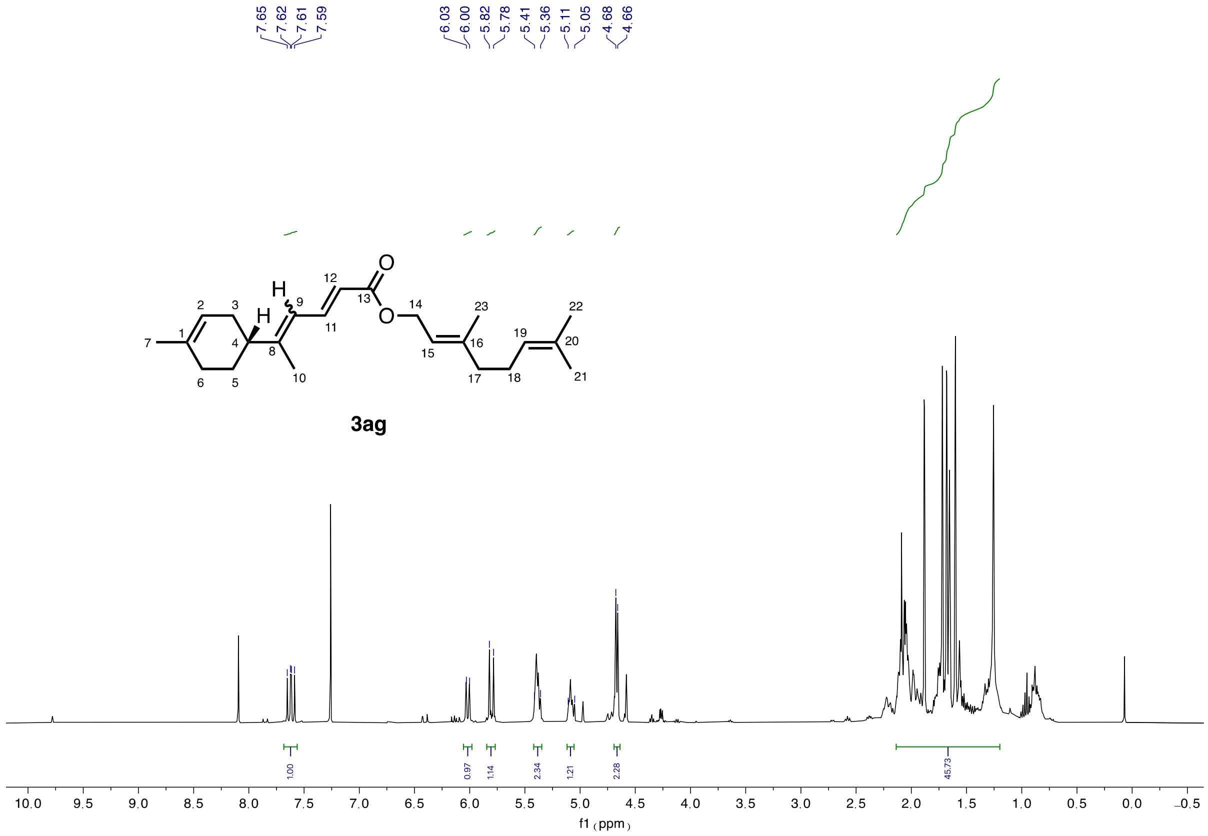

$^{13}C\{^{1}H\}$ NMR Spectrum **3ag** (101 MHz, $CDCl_3$)

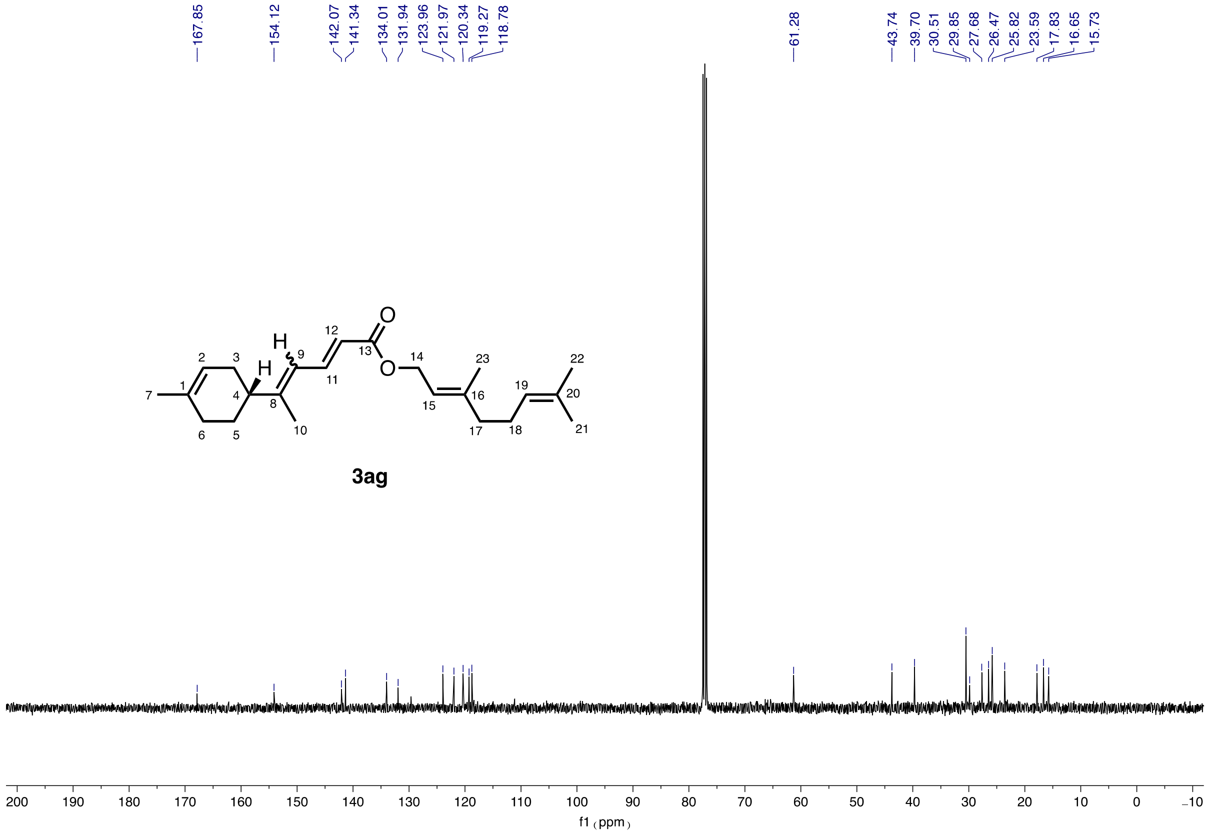

^{1}H NMR Spectrum **3ah** (400 MHz, $CDCl_3$)

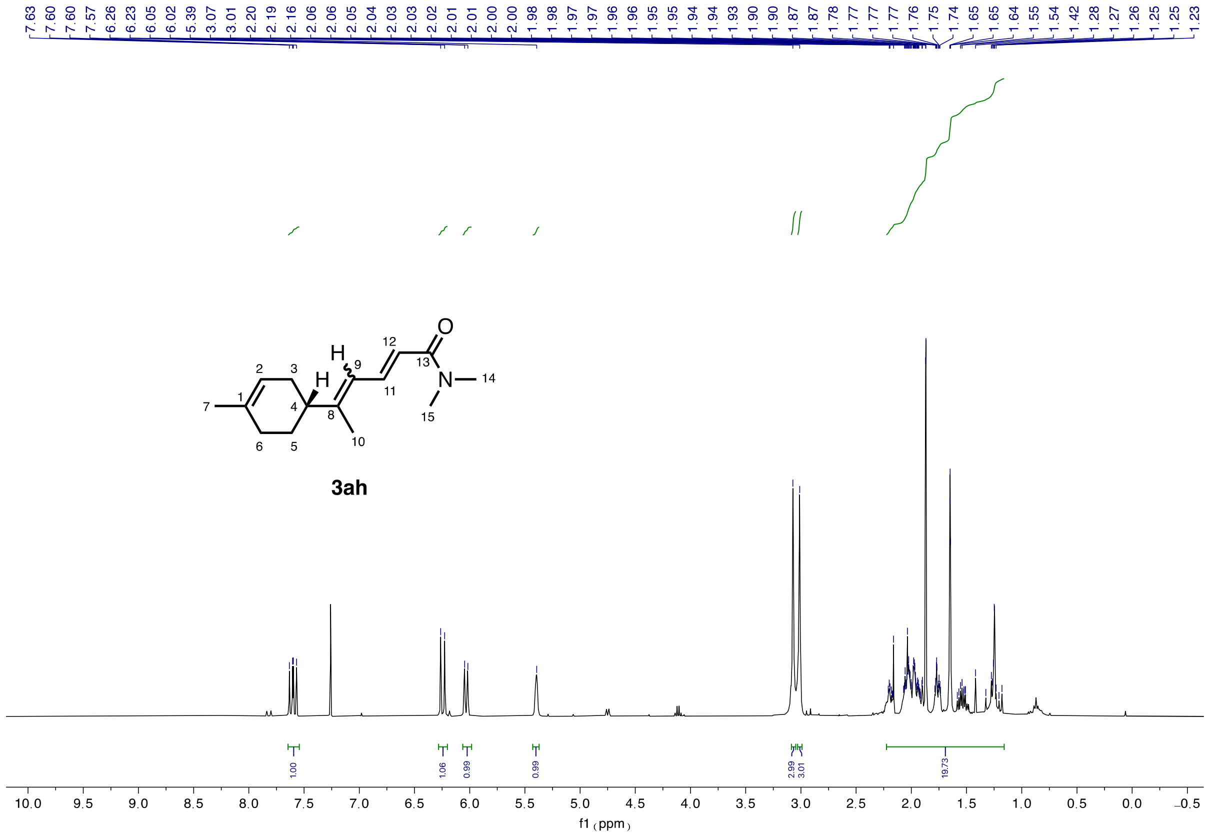

$^{13}C\{^{1}H\}$ NMR Spectrum **3ah** (101 MHz, $CDCl_3$)

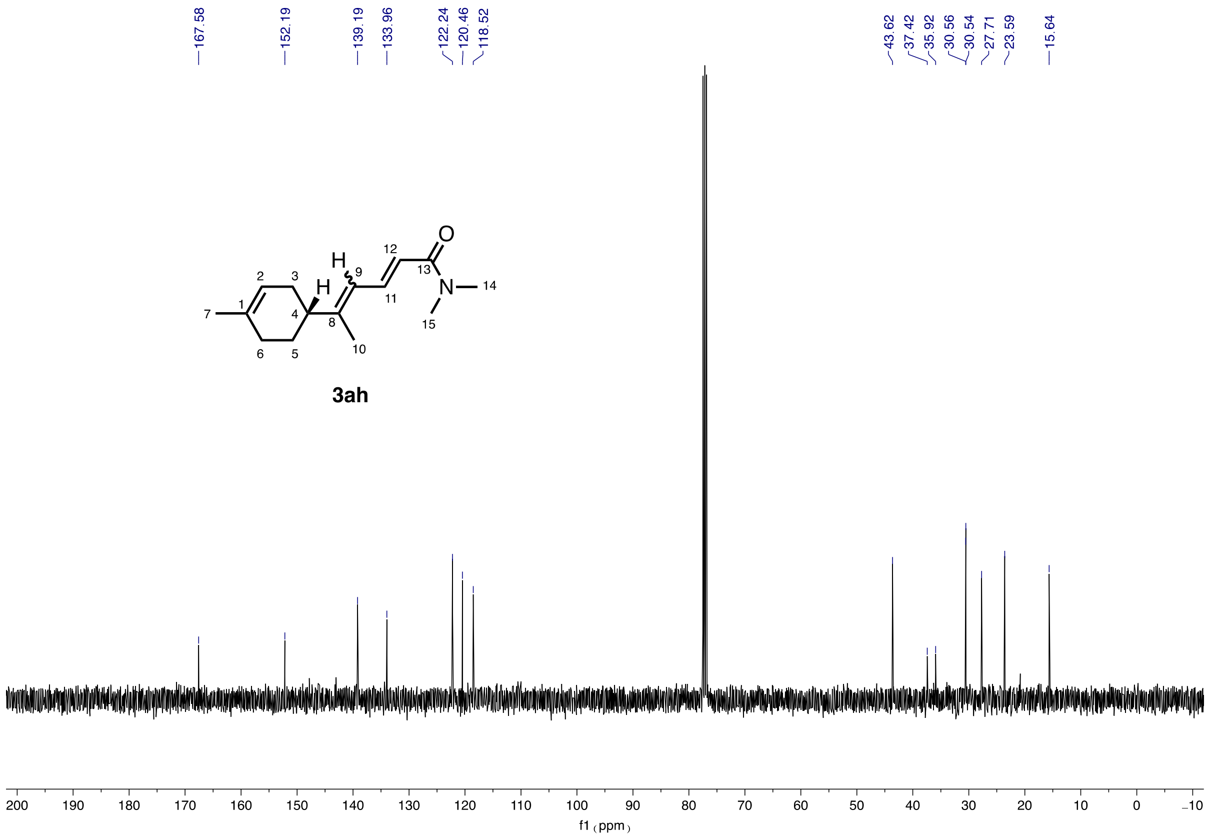

^{1}H NMR Spectrum **3ai** (300 MHz, $CDCl_3$)

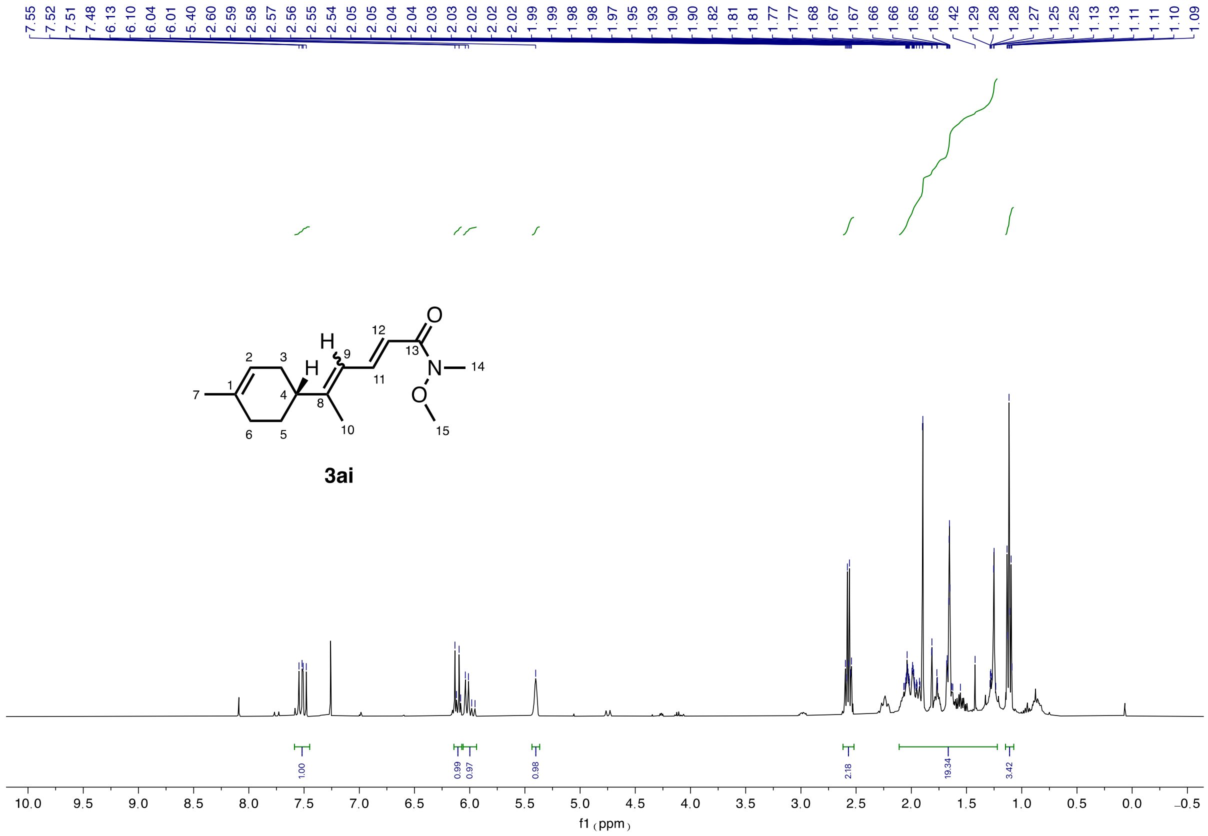

$^{13}C\{^{1}H\}$ NMR Spectrum **3ai** (75 MHz, $CDCl_3$)

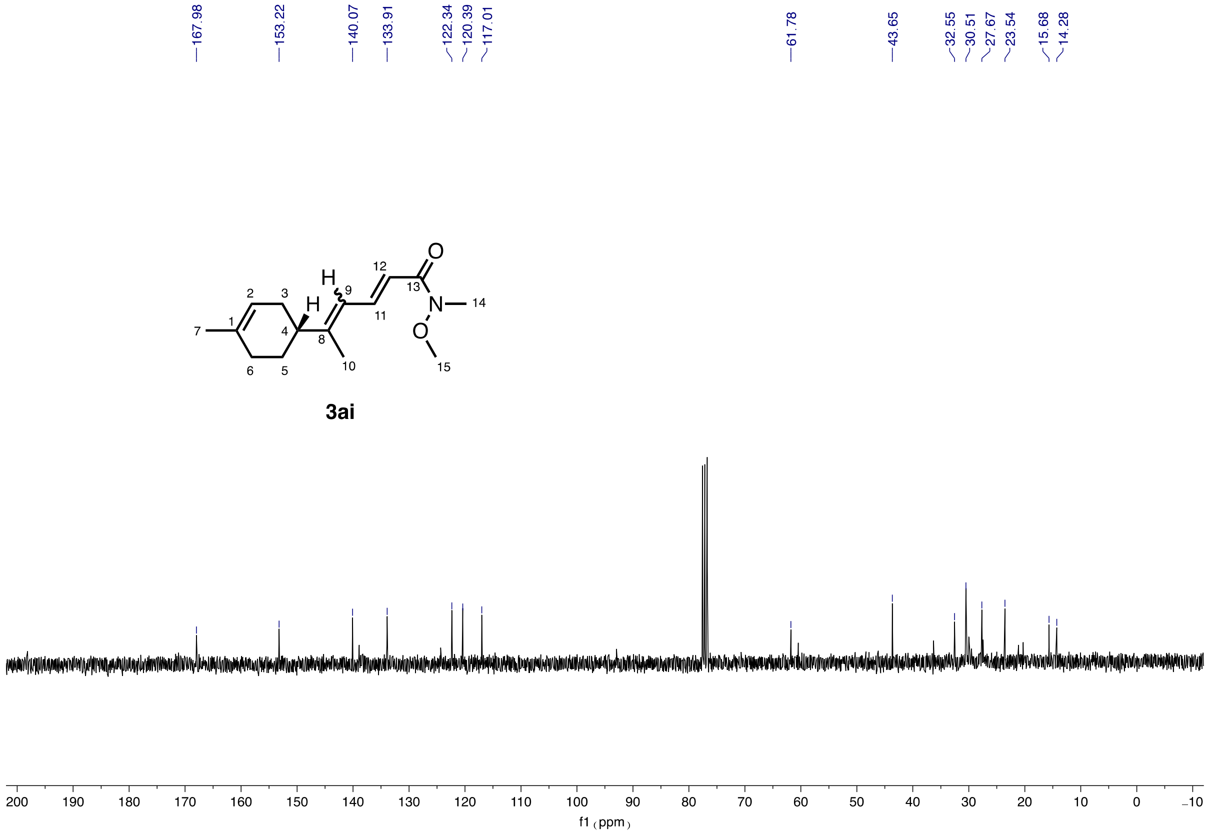

^{1}H NMR Spectrum **3aj** (300 MHz, $CDCl_3$)

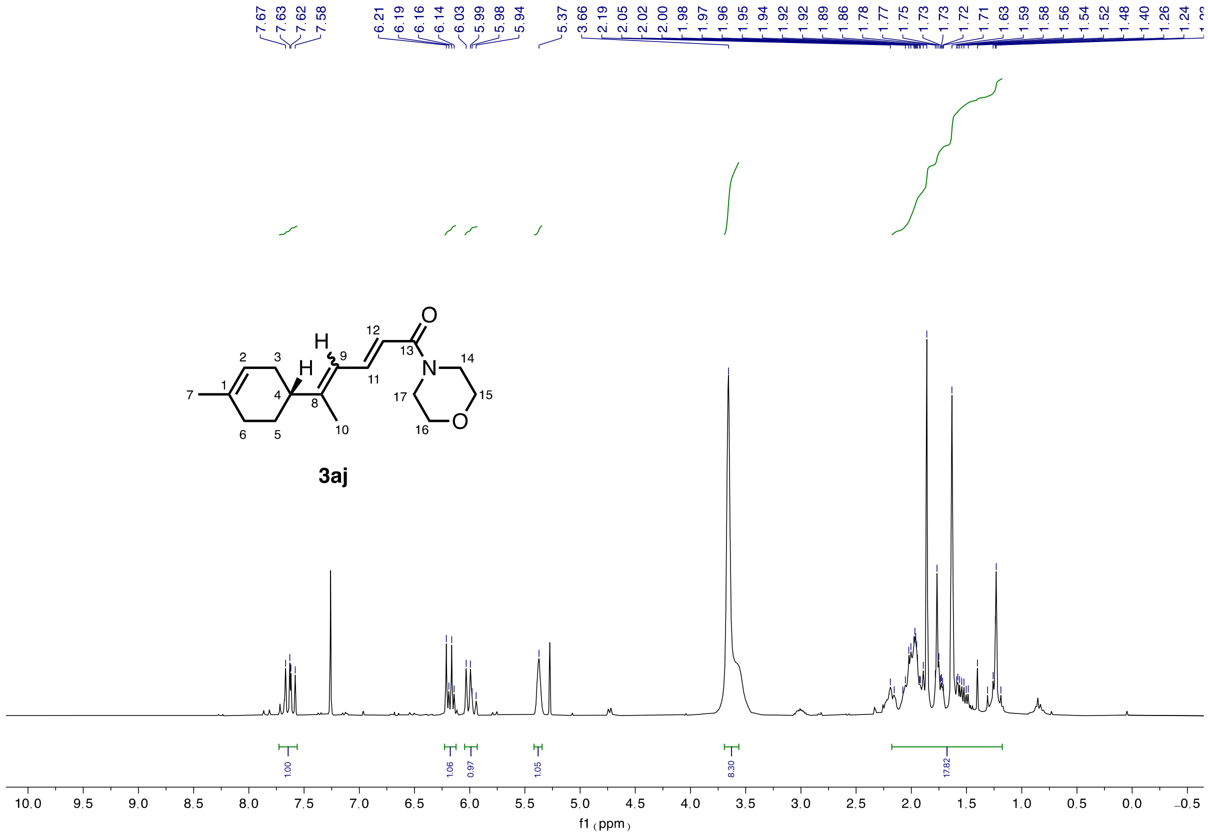

^{13}C{^{1}H} NMR Spectrum **3aj** (75 MHz, $CDCl_3$)

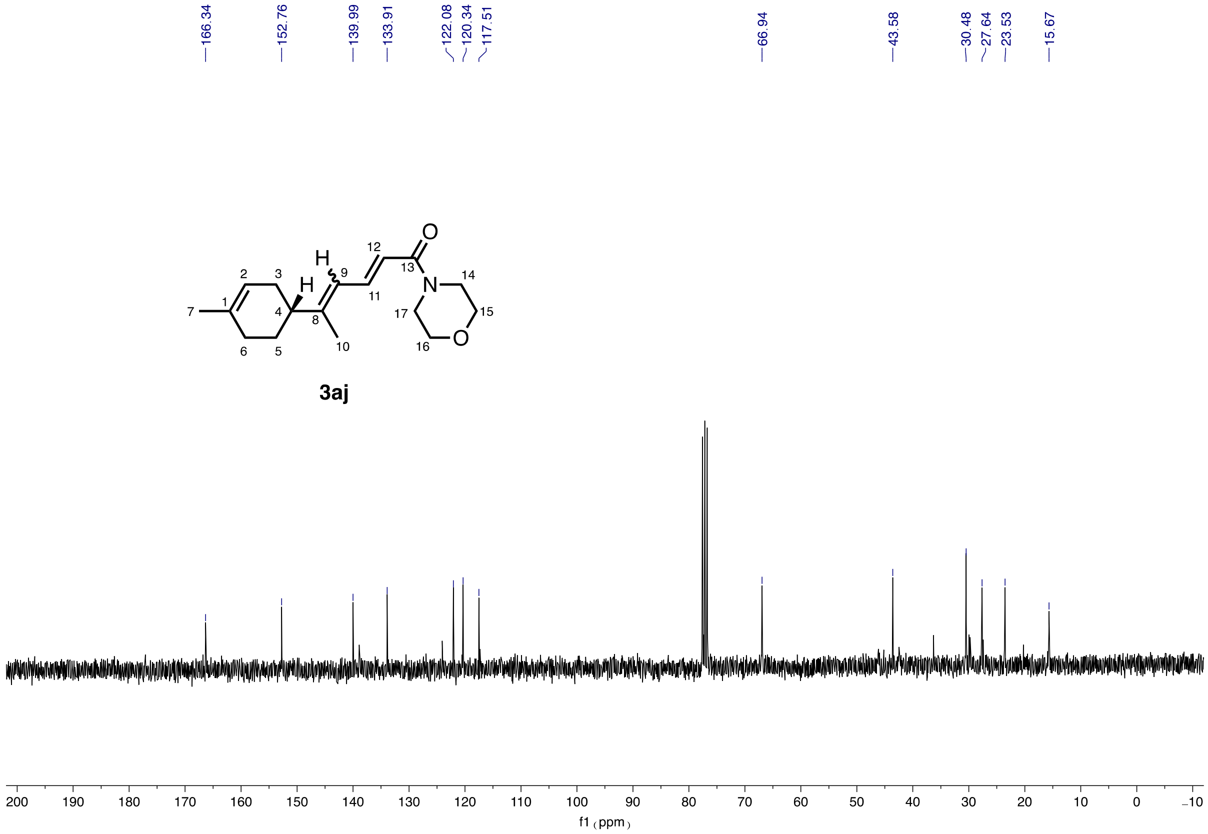

^{1}H NMR Spectrum **3ak** (400 MHz, $CDCl_3$)

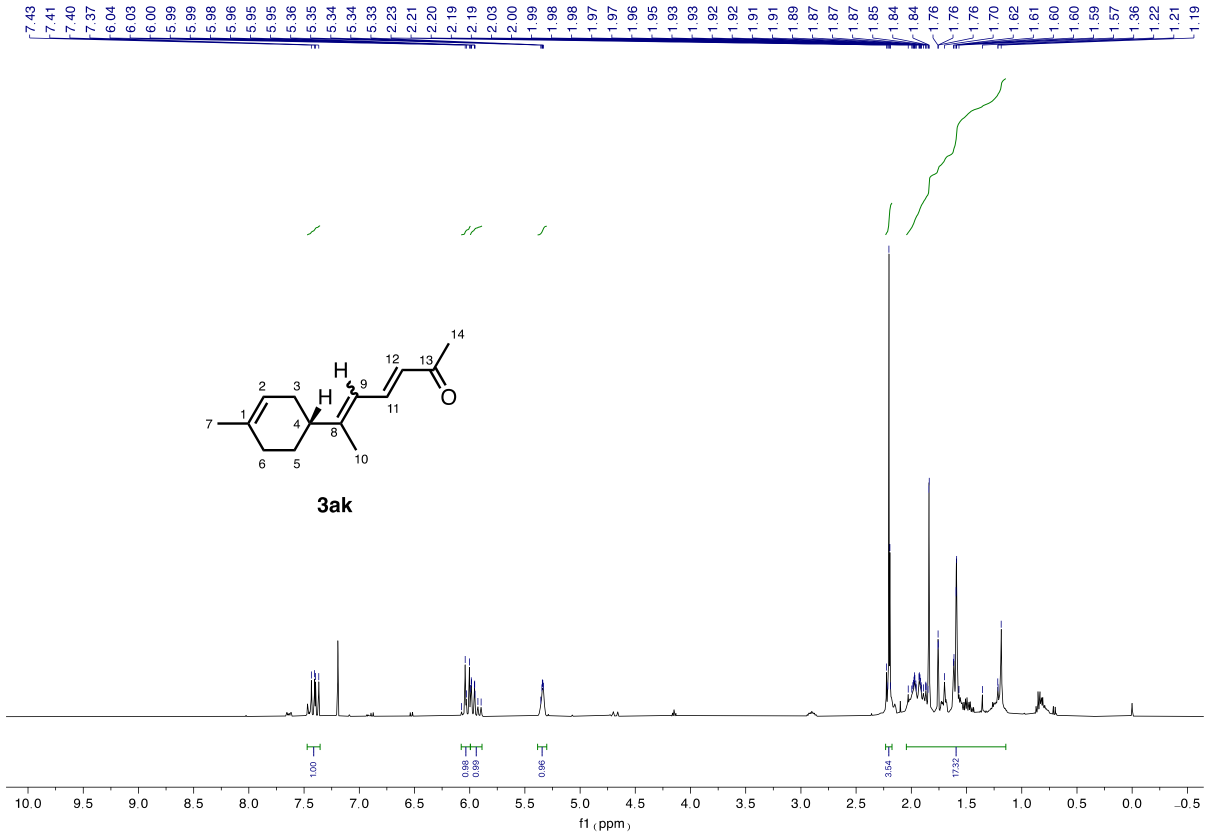

$^{13}C\{^{1}H\}$ NMR Spectrum **3ak** (101 MHz, $CDCl_3$)

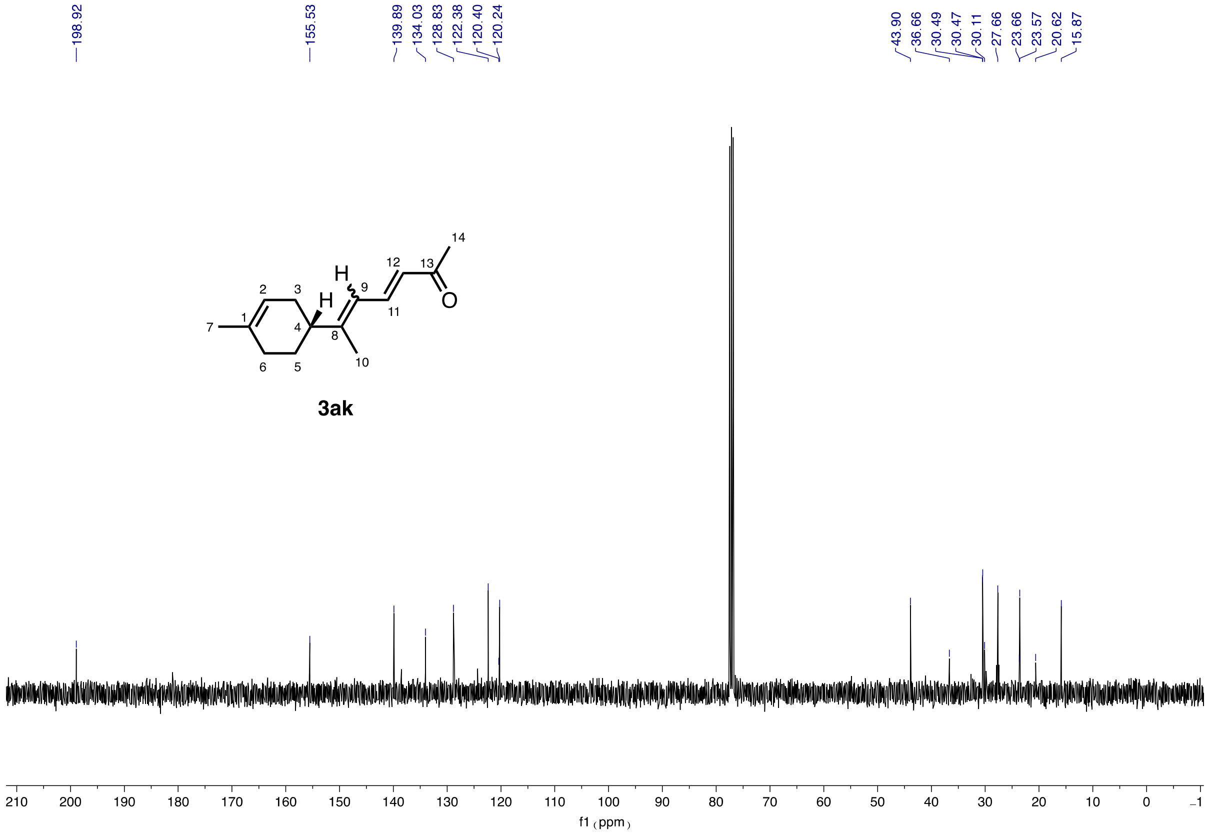

^{1}H NMR Spectrum **3al** (400 MHz, $CDCl_3$)

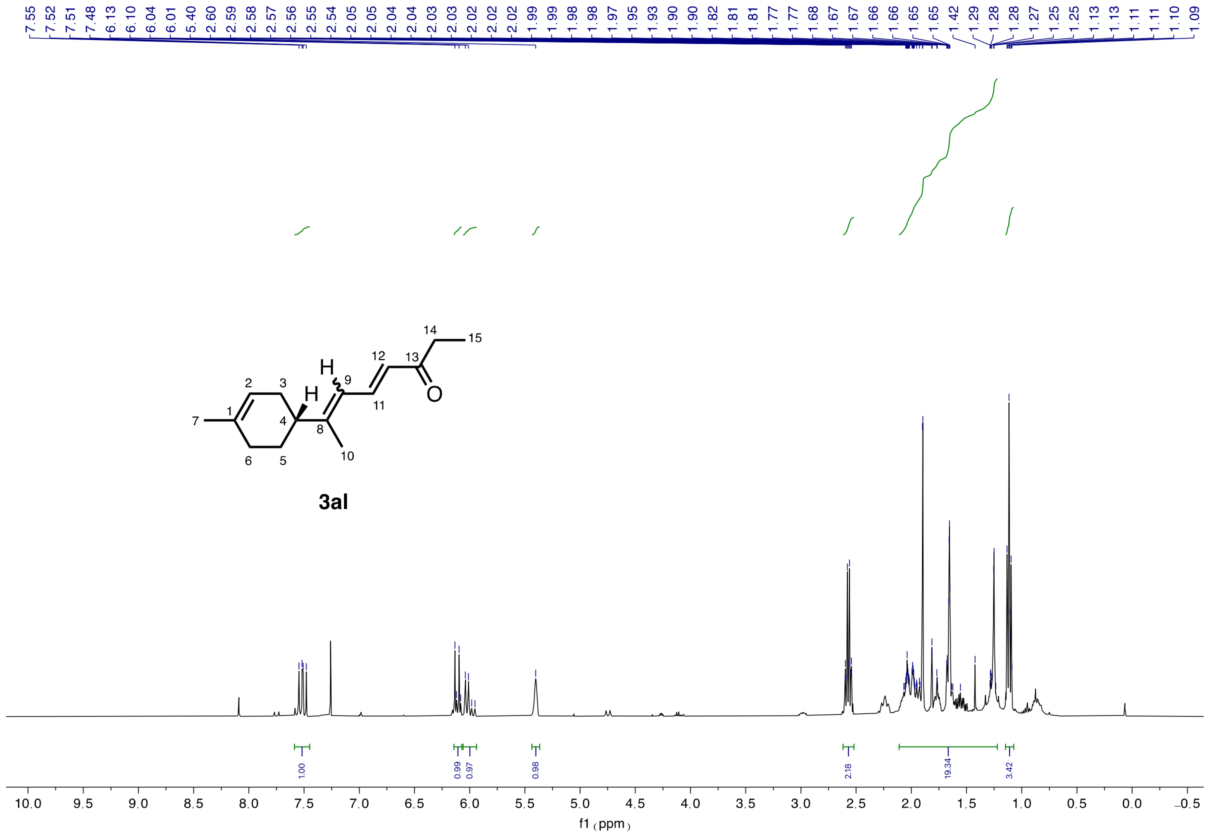

^{13}C{^{1}H} NMR Spectrum **3al** (101 MHz, $CDCl_3$)

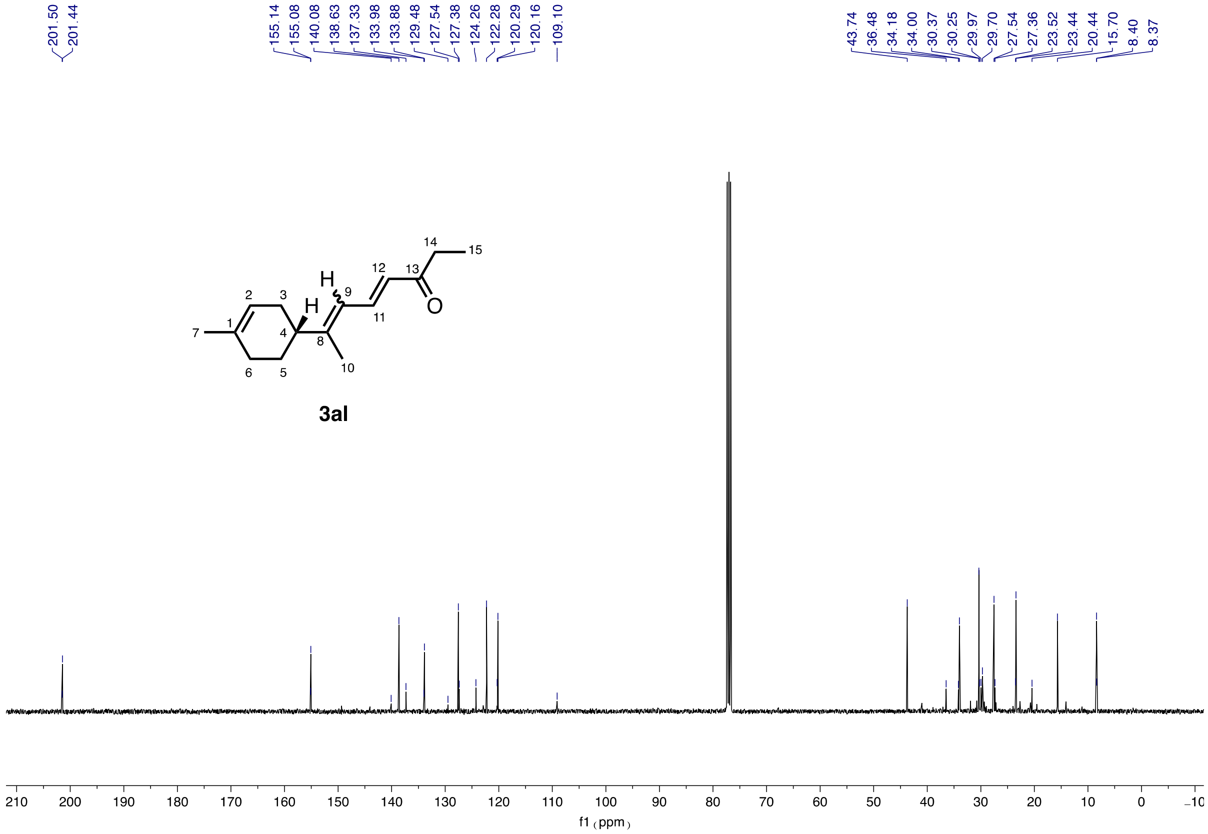

^{1}H NMR Spectrum **3am** (400 MHz, $CDCl_3$)

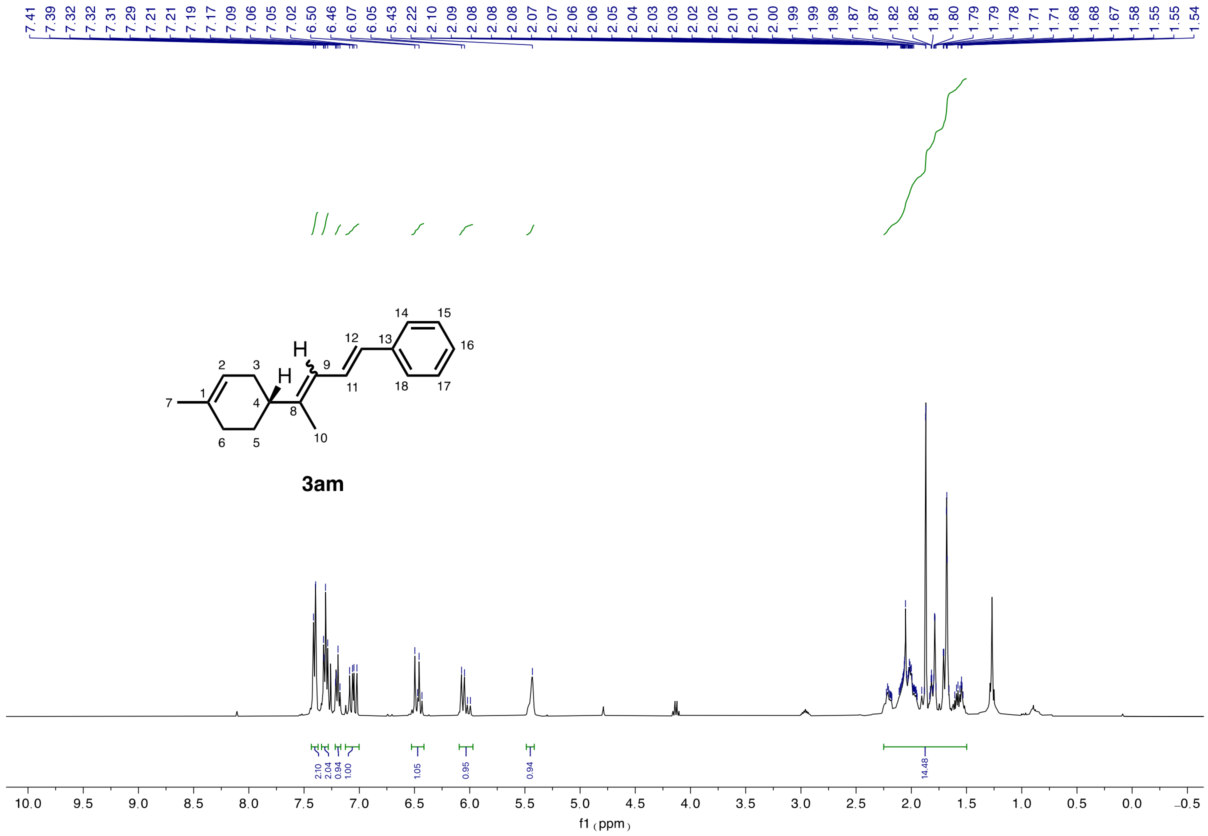

^{13}C{^{1}H} NMR Spectrum **3am** (101 MHz, $CDCl_3$)

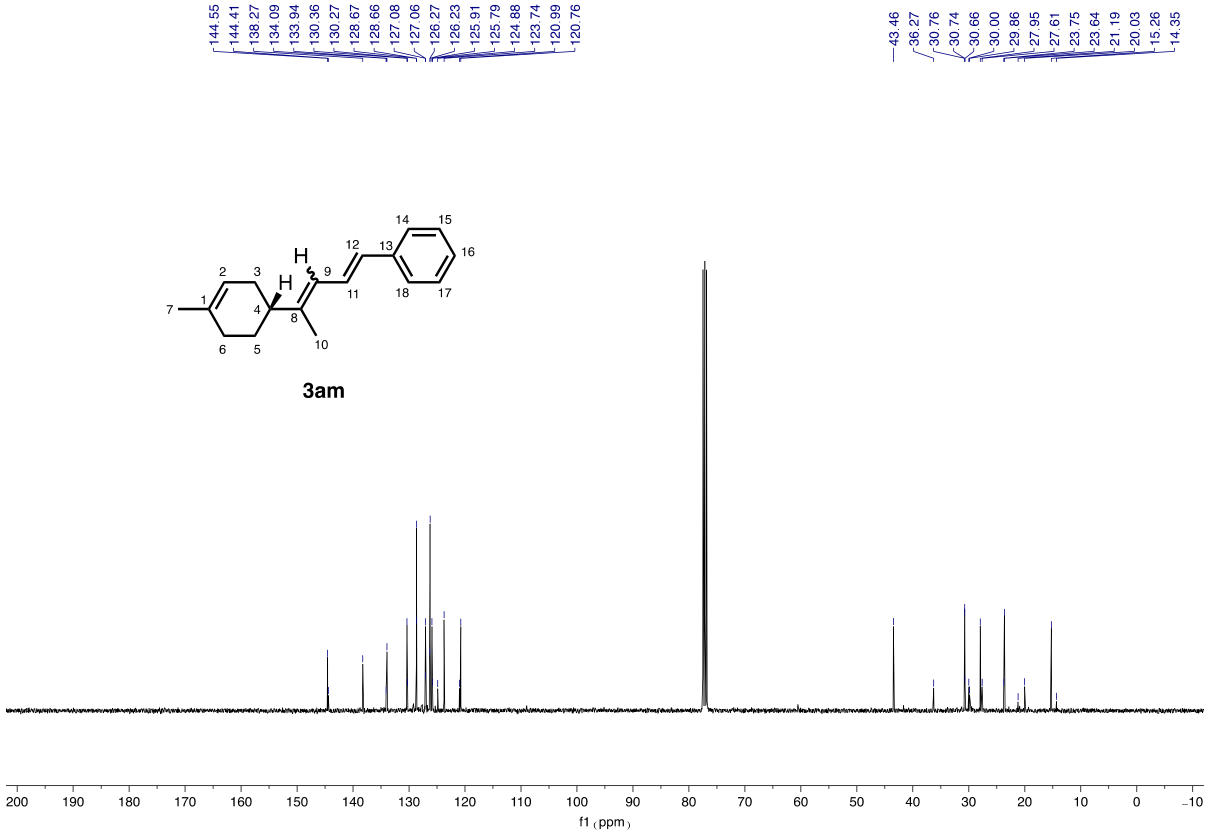

^{1}H NMR Spectrum **3an** (400 MHz, $CDCl_3$)

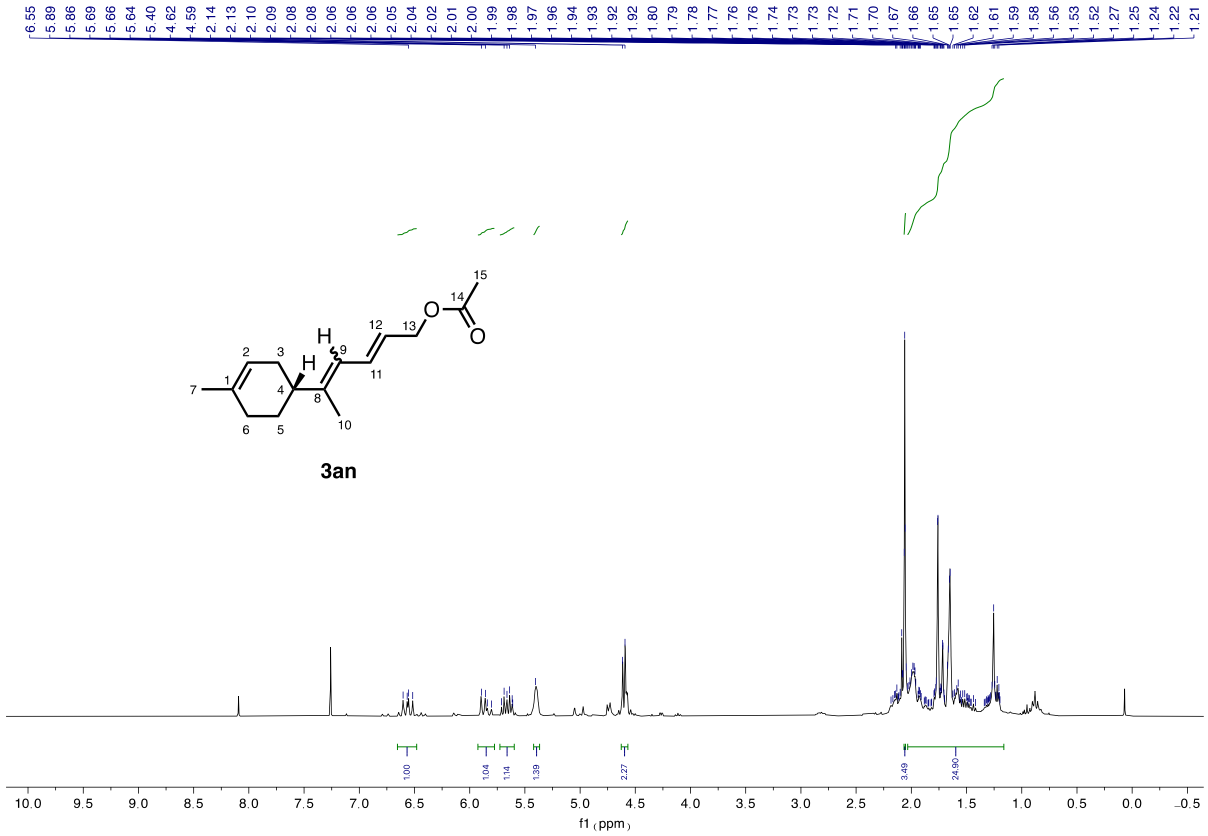

^{13}C{^{1}H} NMR Spectrum **3an** (101 MHz, $CDCl_3$)

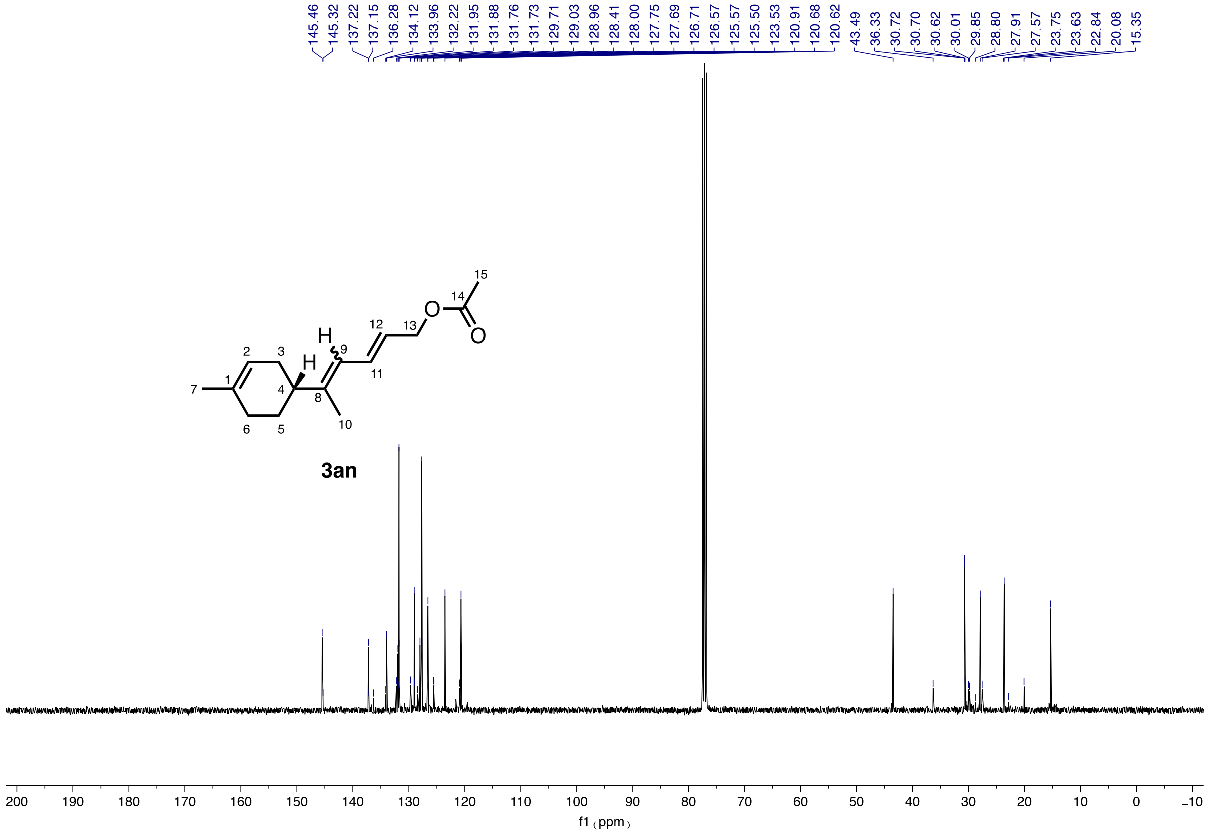

^{1}H NMR Spectrum **3ao** (400 MHz, $CDCl_3$)

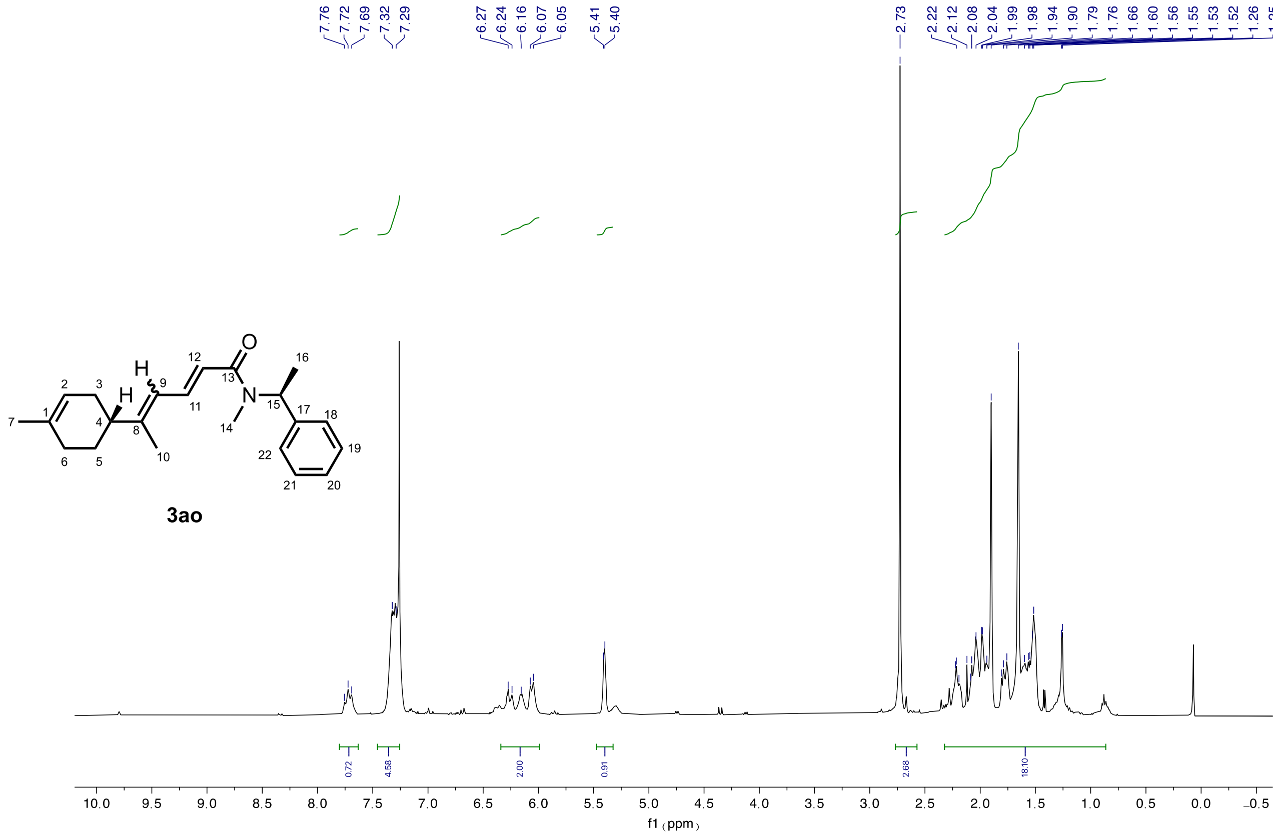

^{13}C{^{1}H} NMR Spectrum **3ao** (101 MHz, $CDCl_3$)

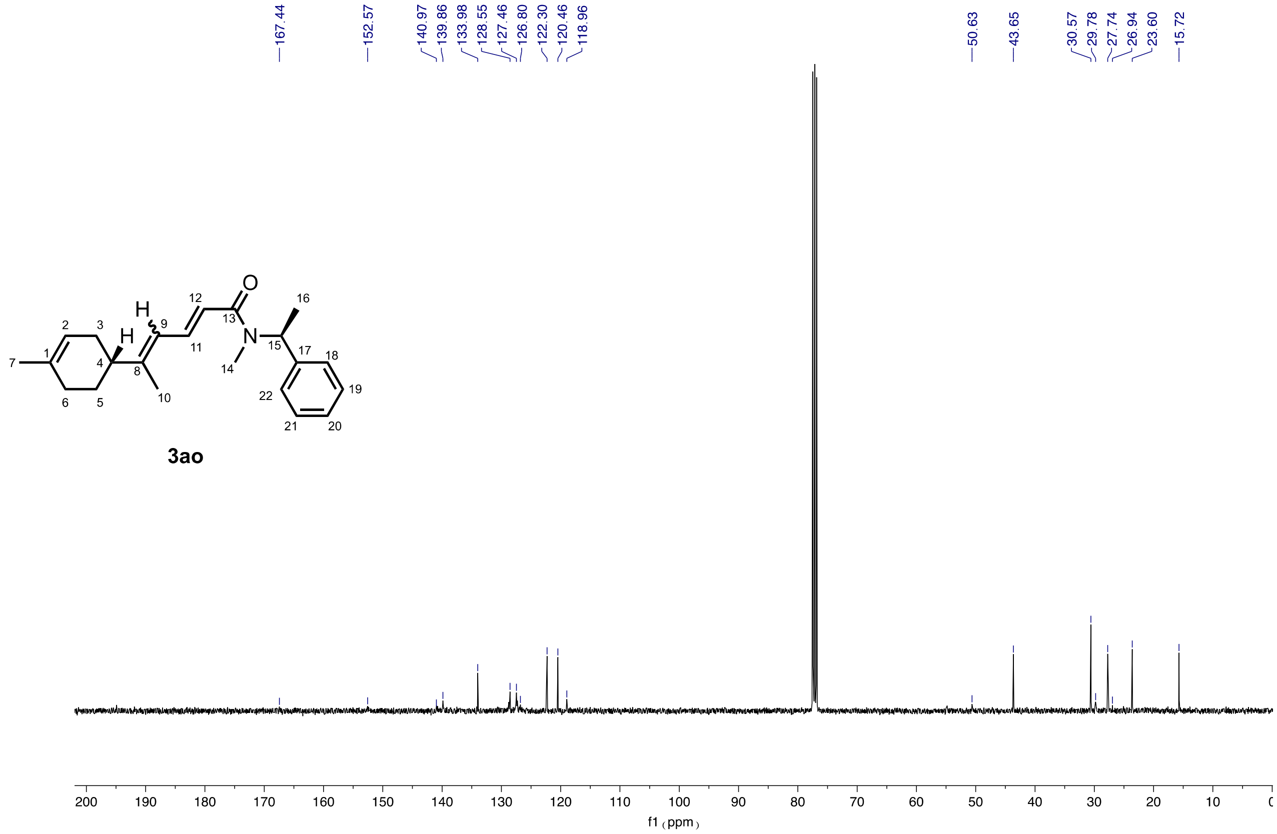

^{1}H NMR Spectrum **3ap** (400 MHz, $CDCl_3$)

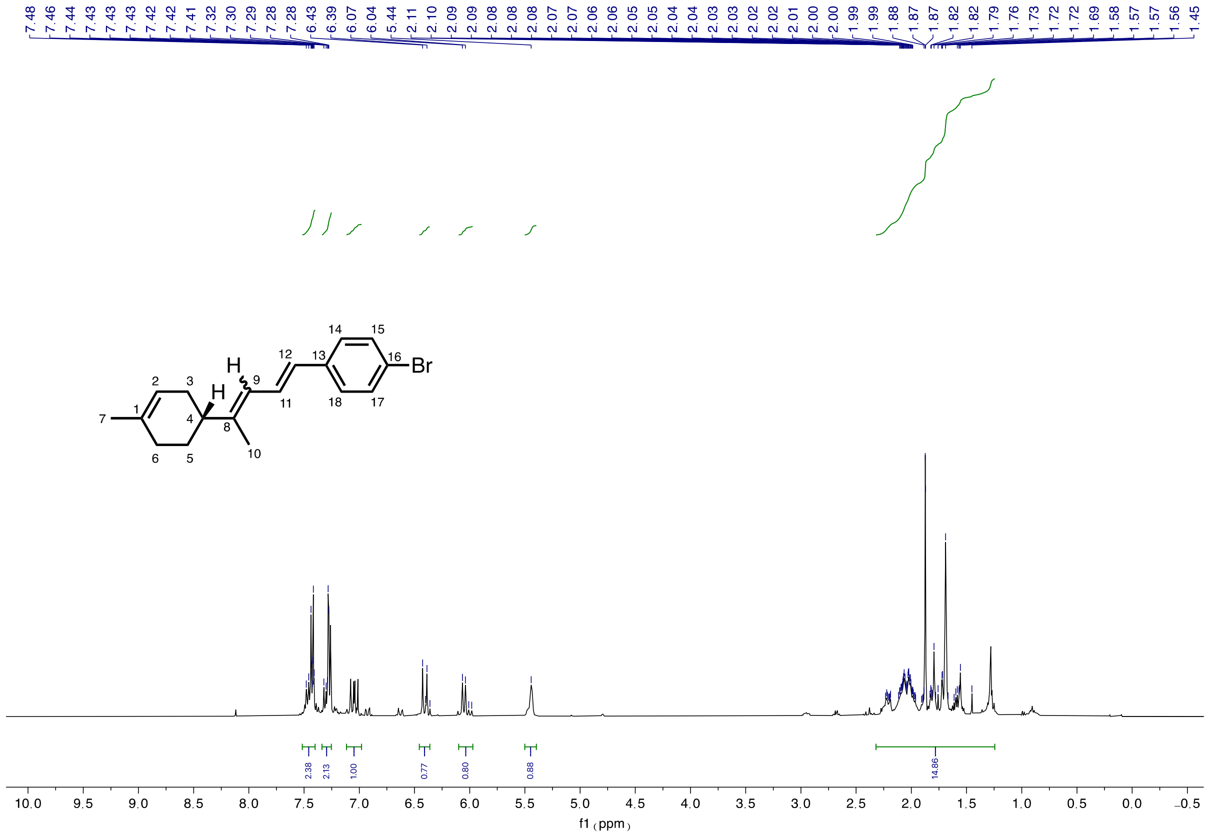

$^{13}C\{^{1}H\}$ NMR Spectrum **3ap** (101 MHz, $CDCl_3$)

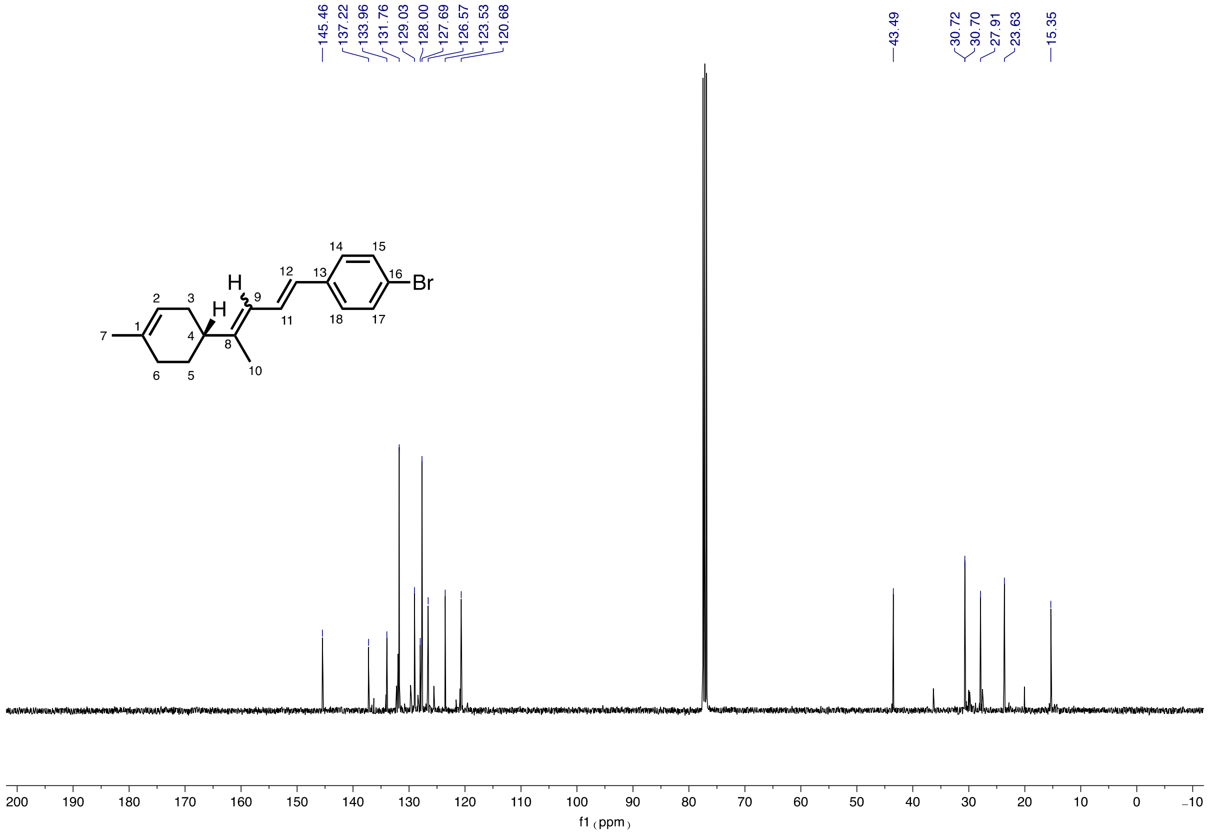

^{1}H NMR Spectrum **3ba** (400 MHz, $CDCl_3$)

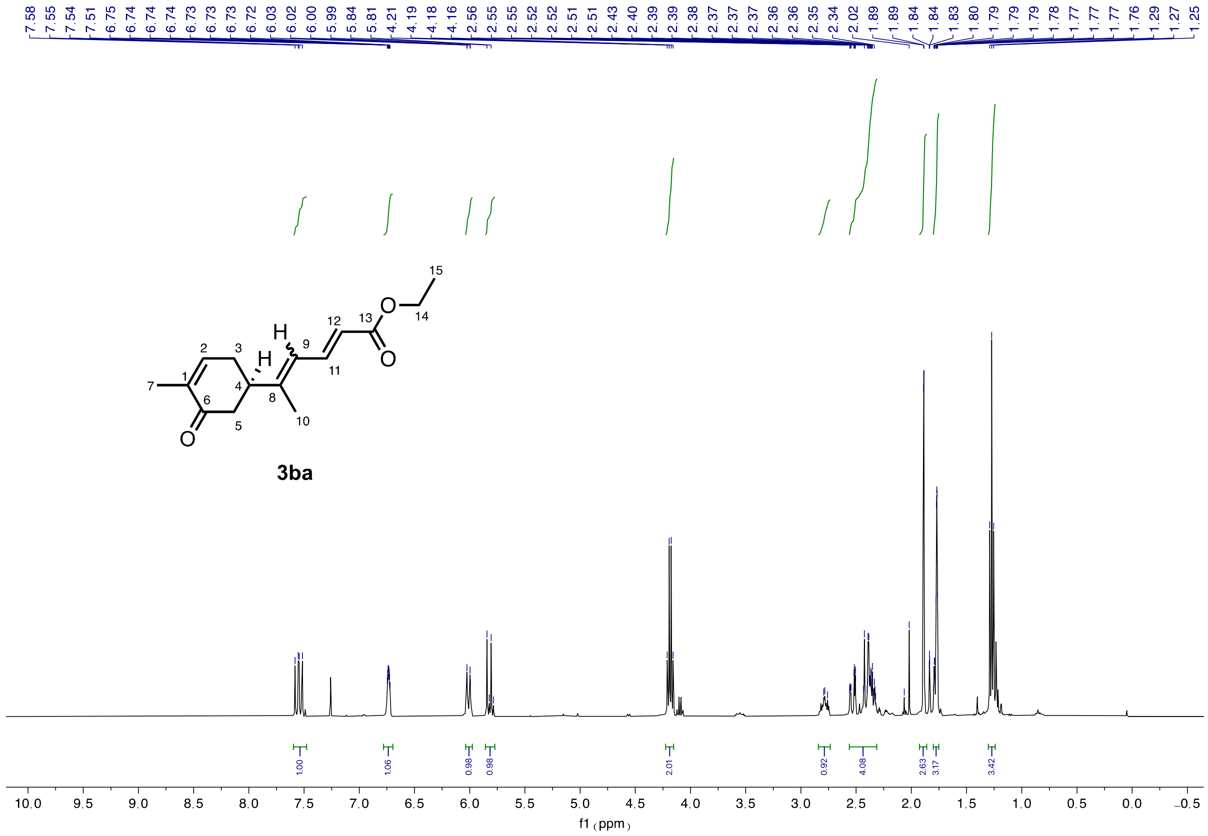

$^{13}C\{^{1}H\}$ NMR Spectrum **3ba** (101 MHz, $CDCl_3$)

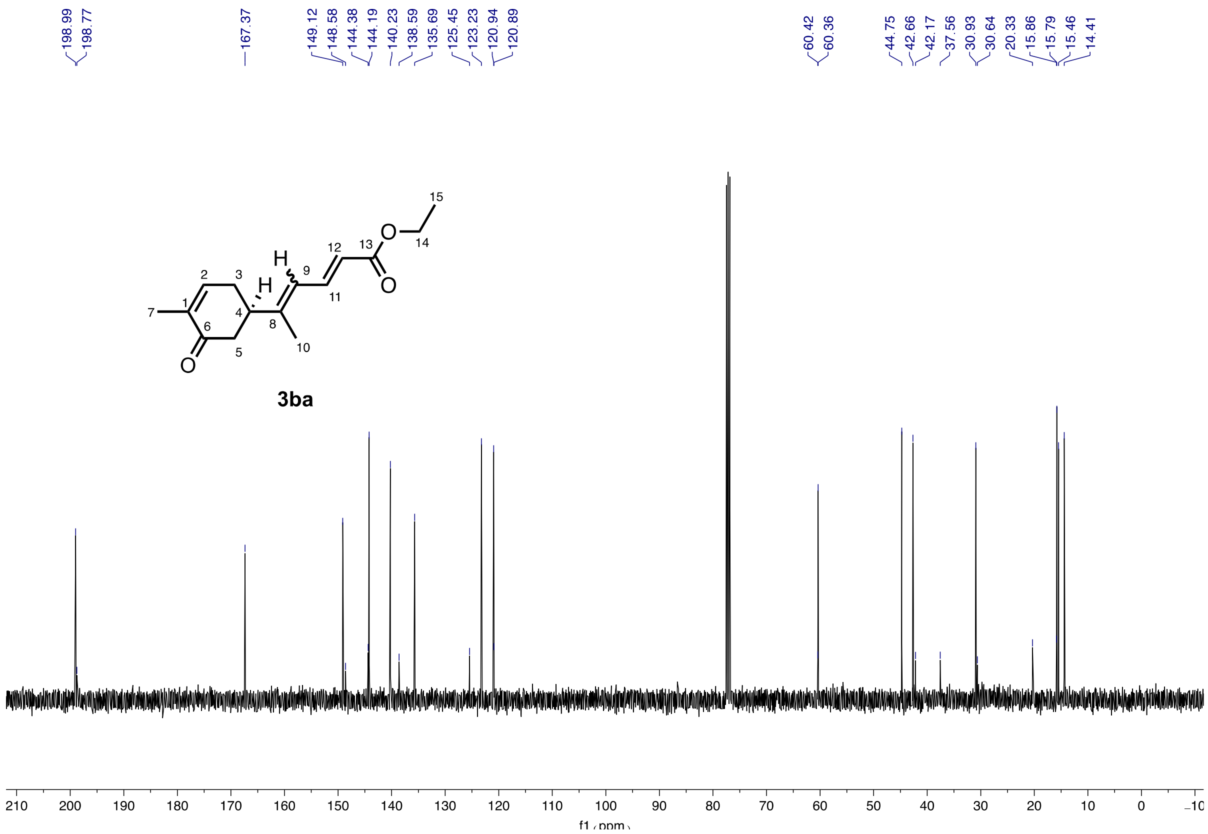

^{1}H NMR Spectrum **3ca** (300 MHz, $CDCl_3$)

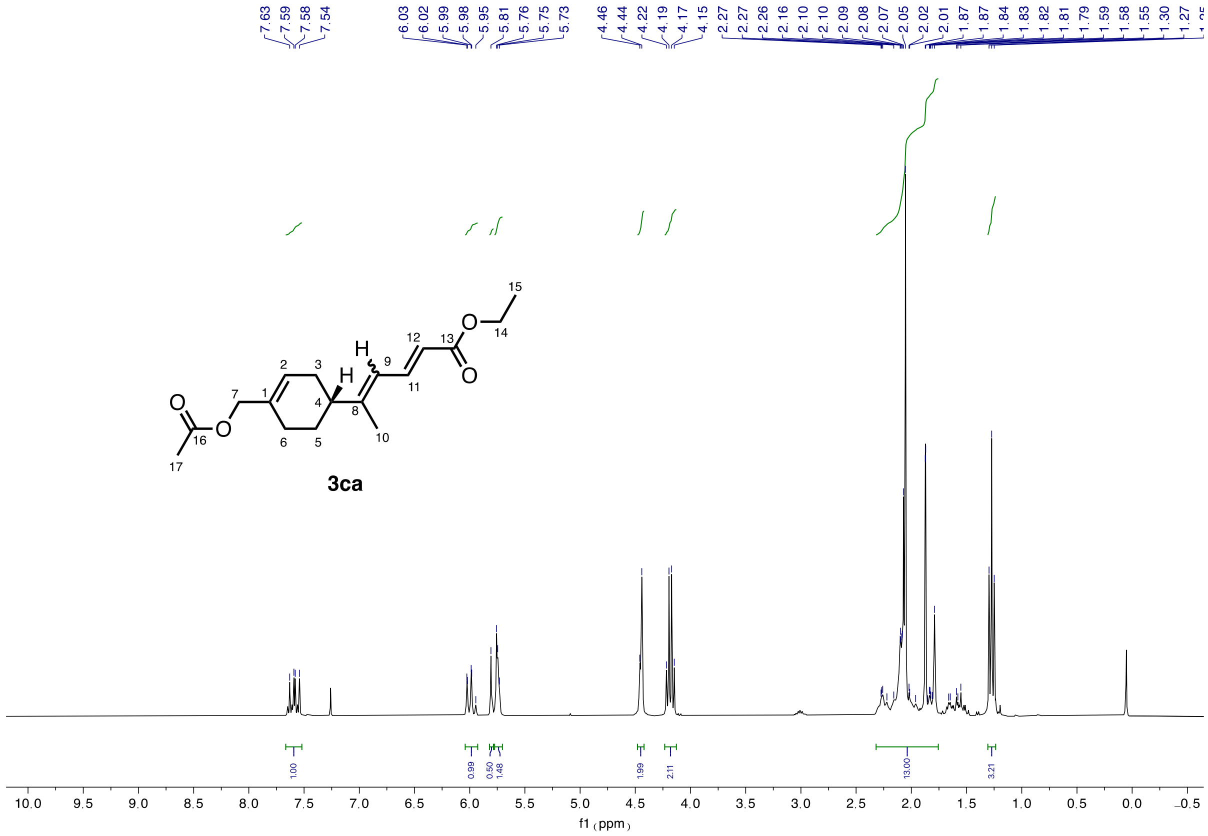

^{13}C{^{1}H} NMR Spectrum **3ca** (75 MHz, $CDCl_3$)

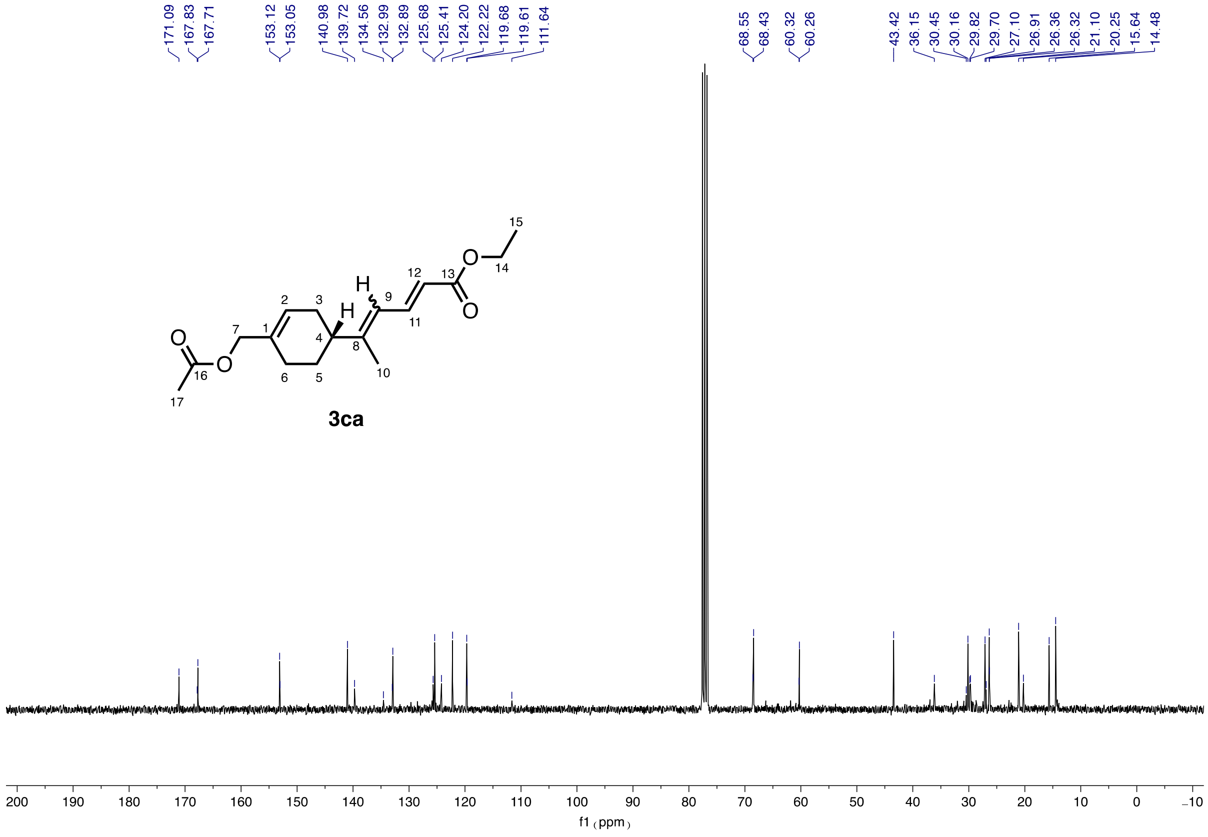

^{1}H NMR Spectrum **3da** (400 MHz, $CDCl_3$)

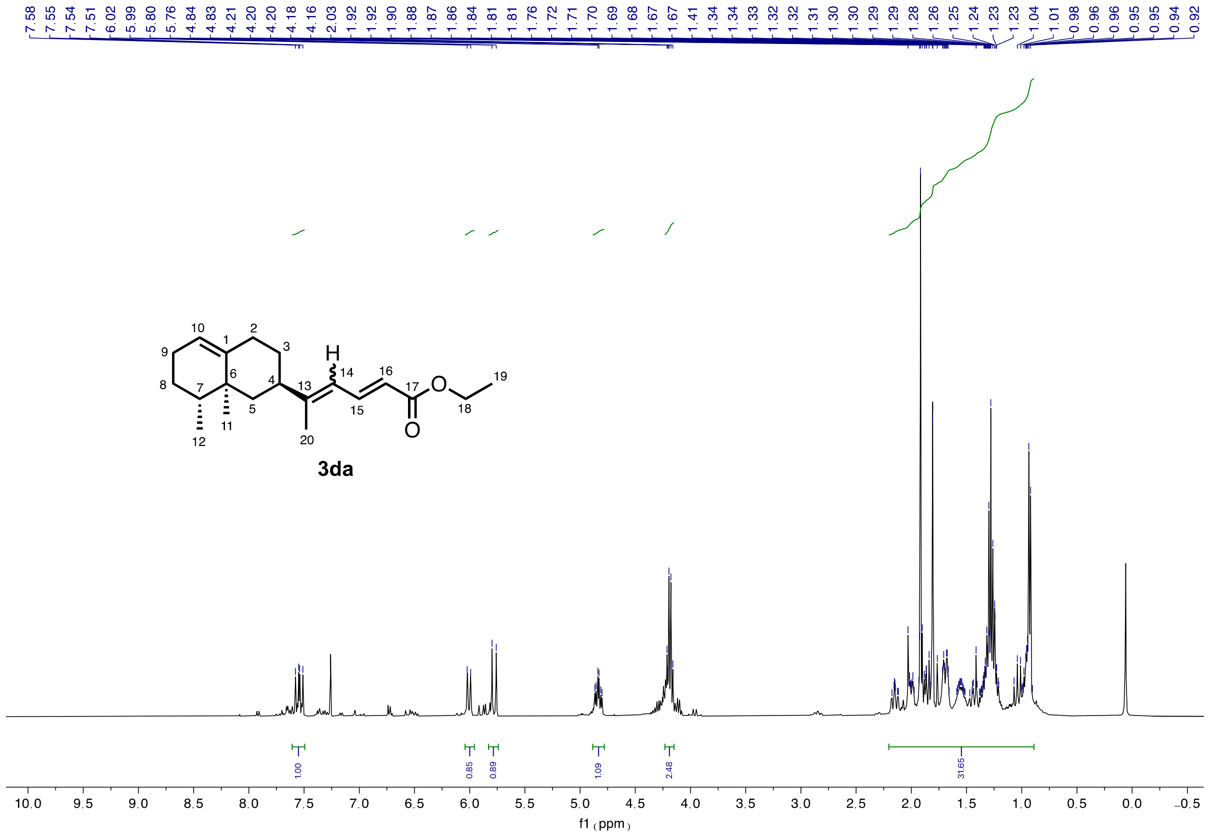

^{13}C{^{1}H} NMR Spectrum **3da** (101 MHz, $CDCl_3$)

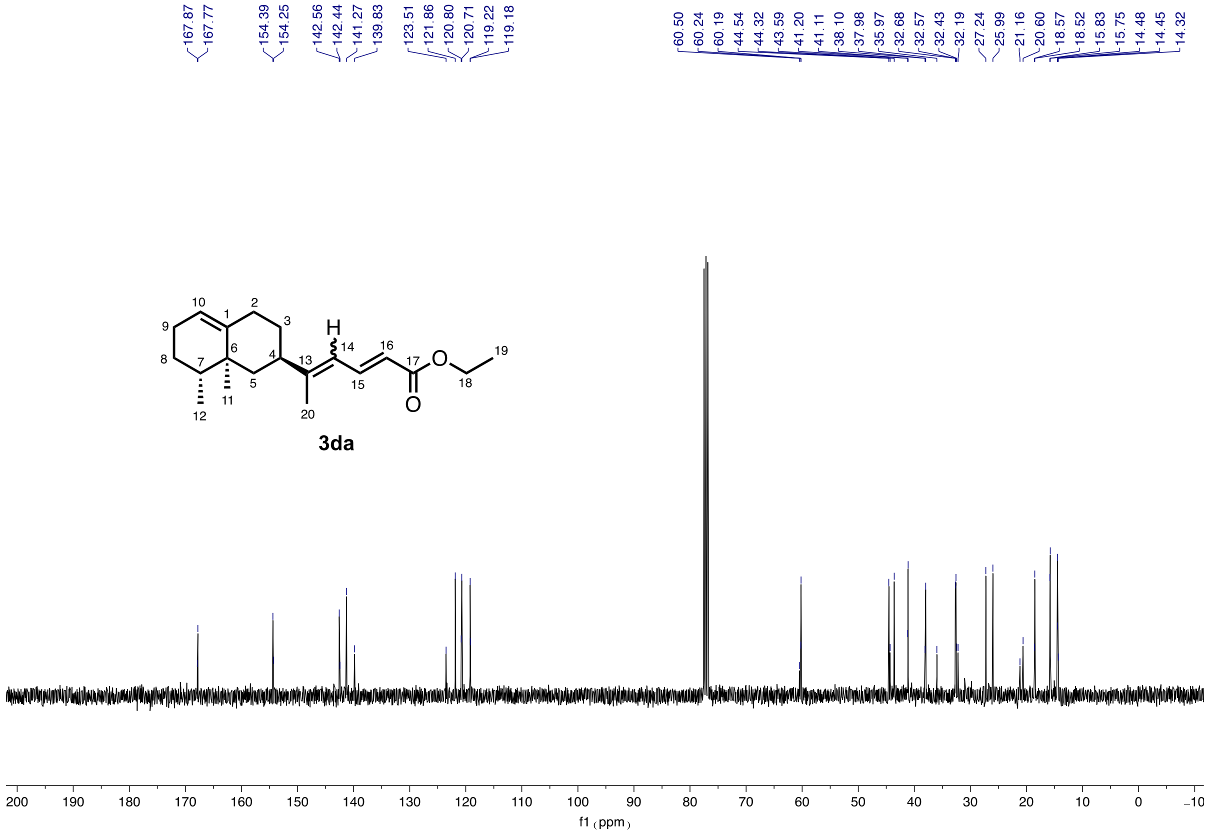

^{1}H NMR Spectrum **3ea** (400 MHz, $CDCl_3$)

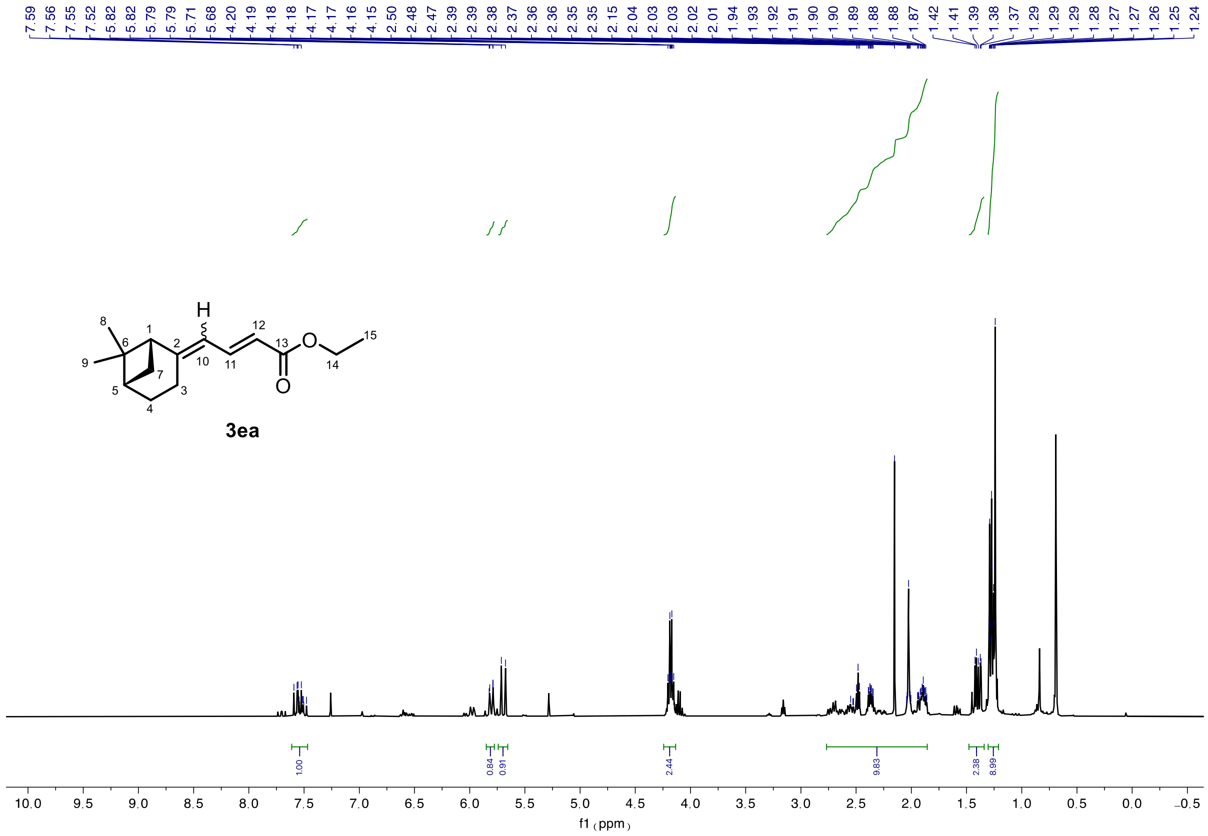

$^{13}C\{^{1}H\}$ NMR Spectrum **3ea** (101 MHz, $CDCl_3$)

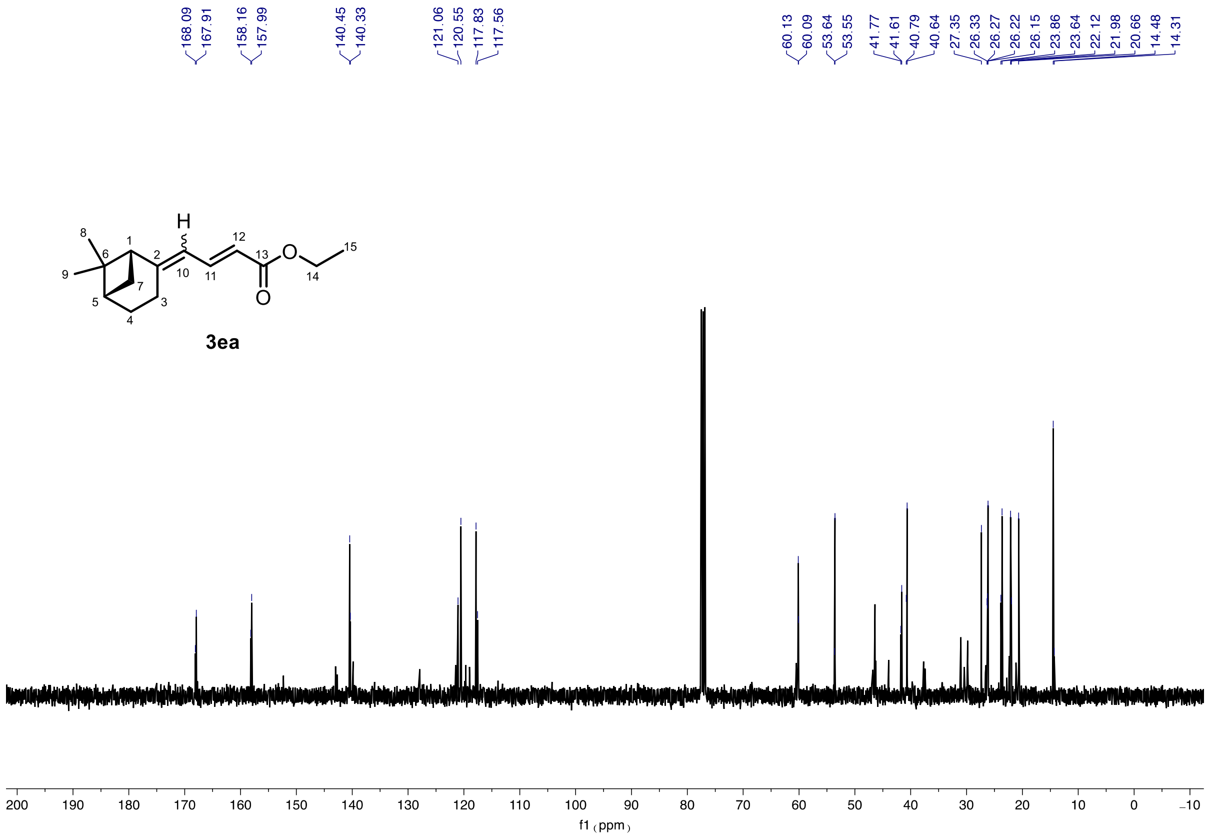

^{1}H NMR Spectrum **3fa** (400 MHz, $CDCl_3$)

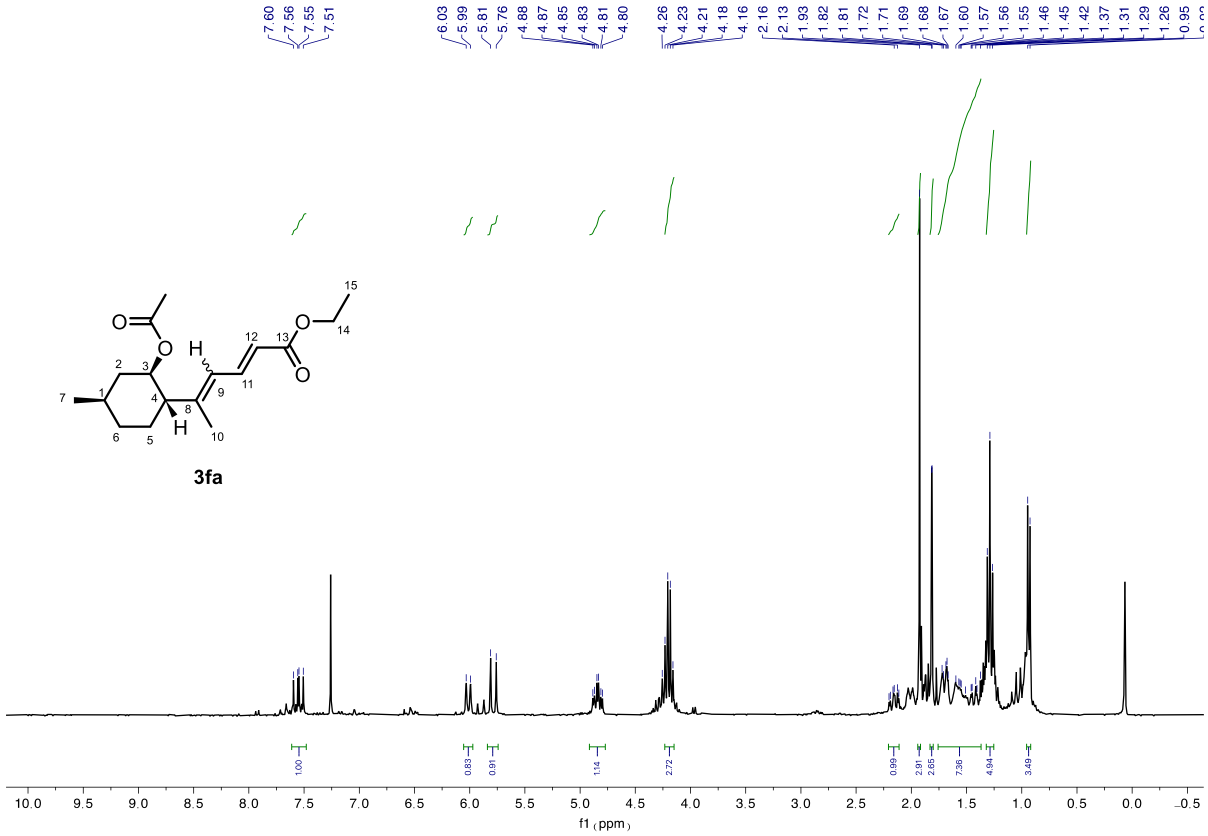

^{13}C{^{1}H} NMR Spectrum **3fa** (101 MHz, $CDCl_3$)

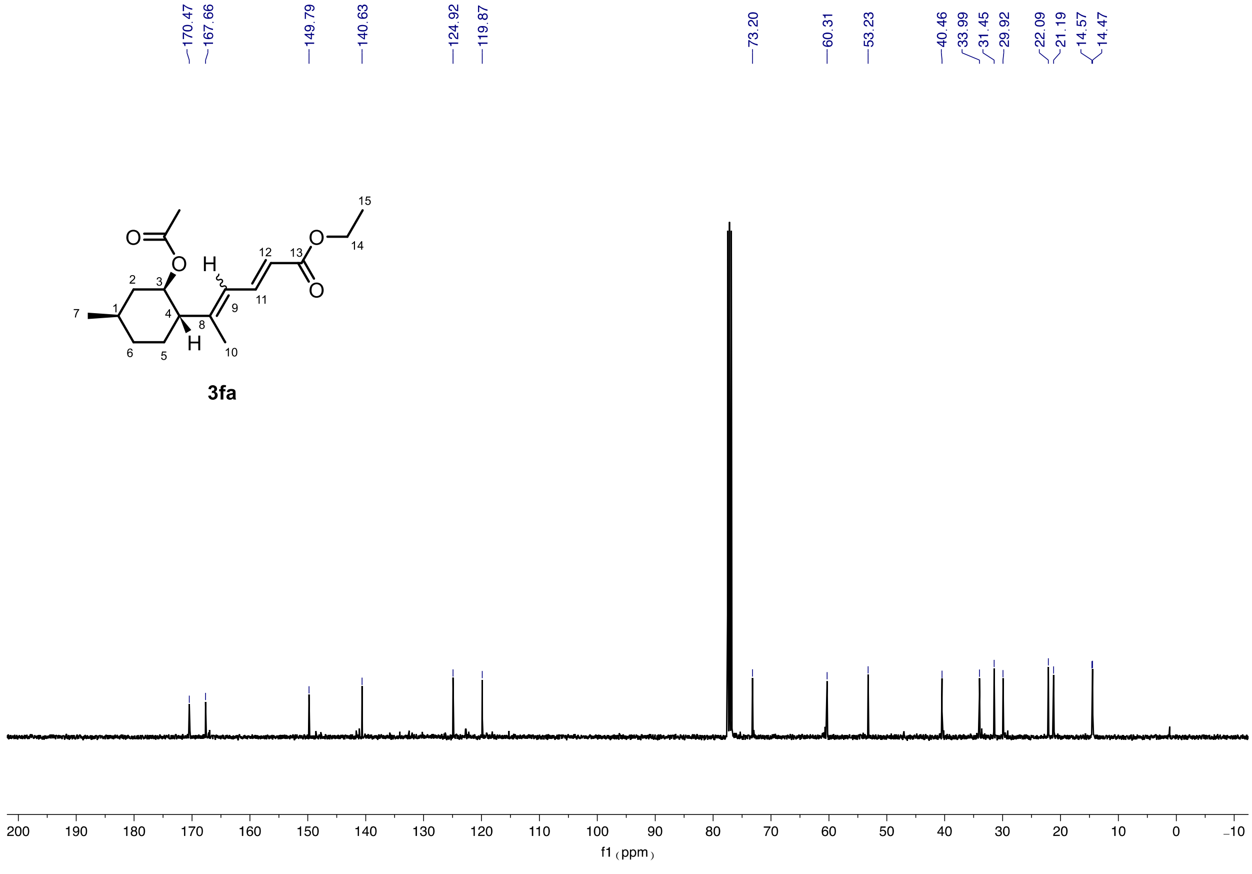

^{1}H NMR Spectrum **3*ent*-ao** (400 MHz, $CDCl_3$)

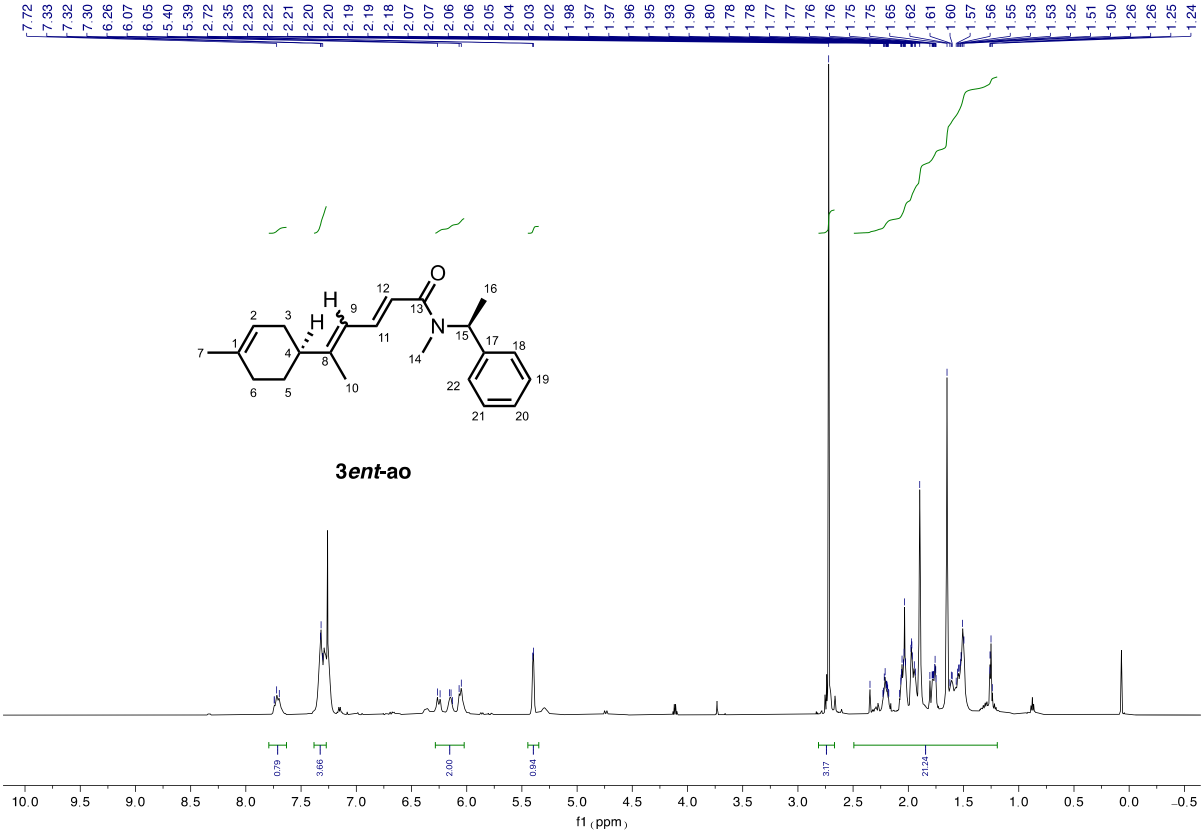

^{13}C{^{1}H} NMR Spectrum **3*ent*-ao** (101 MHz, $CDCl_3$)

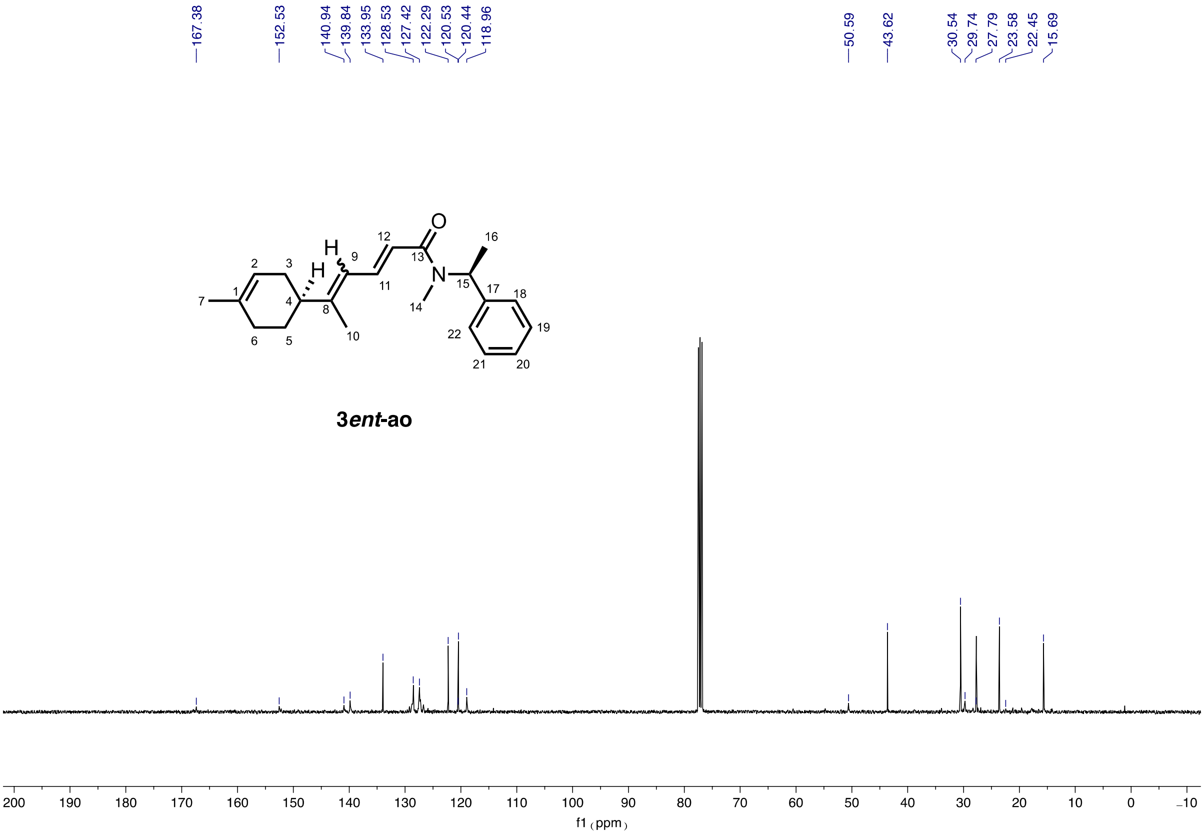

^{1}H NMR Spectrum **4** (400 MHz, $CDCl_3$)

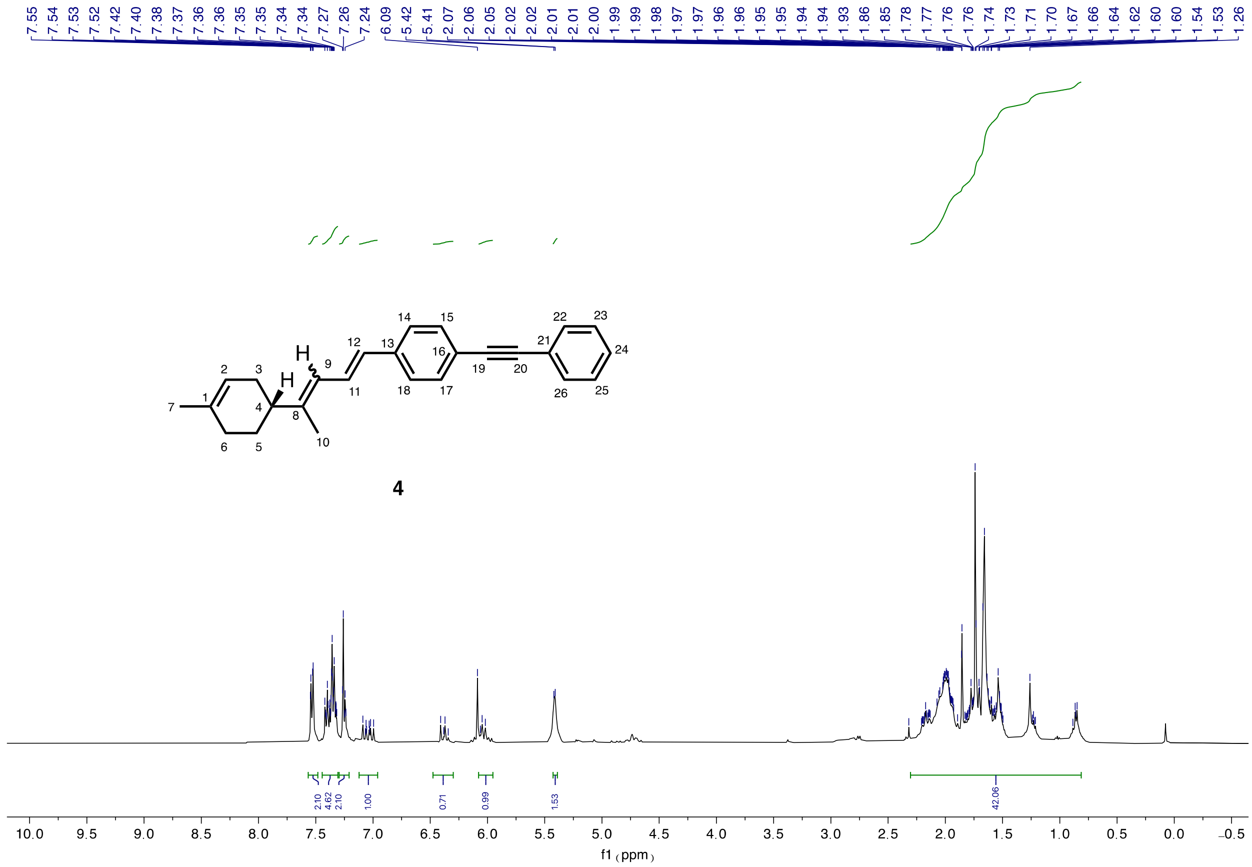

$^{13}C\{^{1}H\}$ NMR Spectrum **4** (101 MHz, $CDCl_3$)

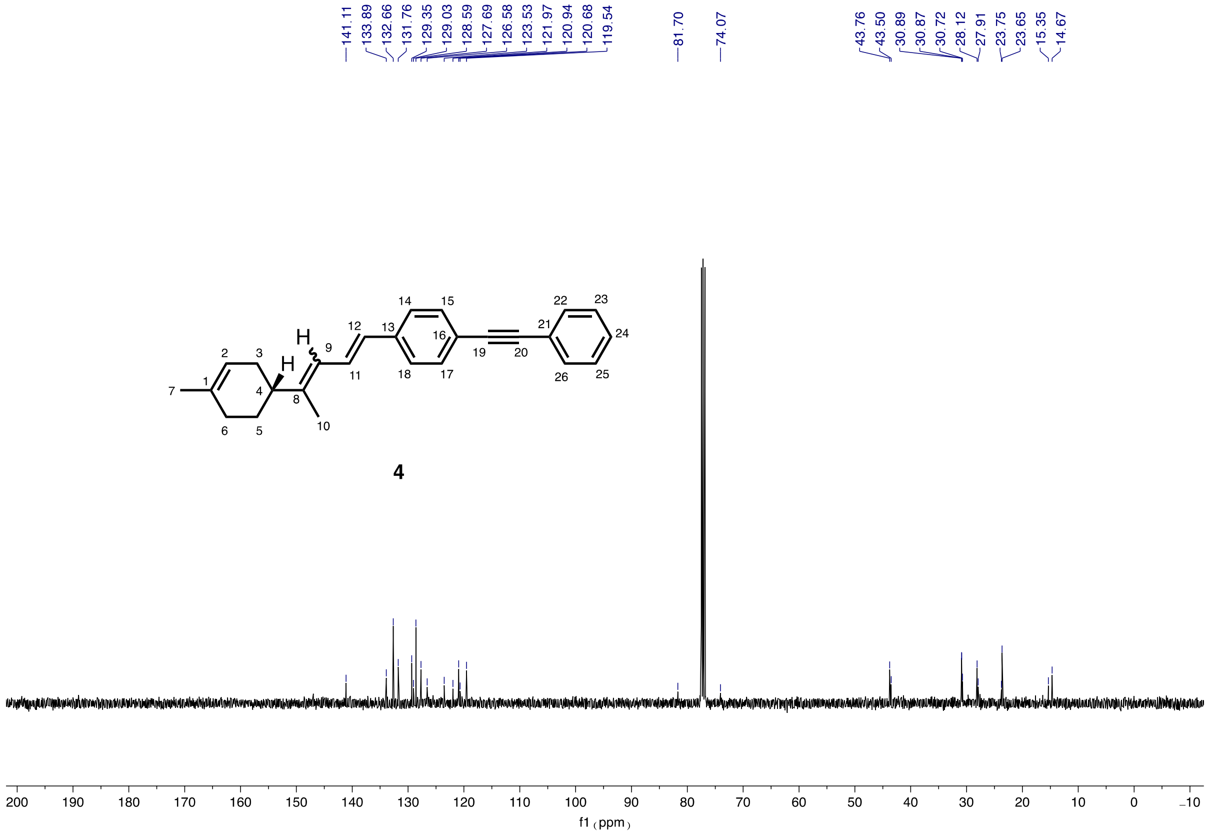

11. Computational details

Calculations were performed using the GAUSSIAN 09 software package[14] and the PBE0 functional, without symmetry constraints. That functional uses a hybrid generalized gradient approximation (GGA), including 25 % mixture of Hartree-Fock[15] exchange with DFT[16] exchange-correlation, given by Perdew, Burke and Ernzerhof functional (PBE).[17] The optimized geometries were obtained with the Stuttgart Effective Core Potentials and associated basis set (SDD)[18] for Pd and Ag with an added *f*-polarization function,[19] and a standard 6-31G(d,p)[20] for the remaining elements (basis b1). Transition state optimizations were performed with the Synchronous Transit-Guided Quasi-Newton Method (STQN) developed by Schlegel *et al*,[21] following extensive searches of the Potential Energy Surface. Frequency calculations were performed to confirm the nature of the stationary points, yielding one imaginary frequency for the transition states and none for the minima. Each transition state was further confirmed by following its vibrational mode downhill on both sides and obtaining the minima presented on the energy profile. The electronic energies (E_{b1}) obtained at the PBE0/b1 level of theory were converted to free energy at 298.15 K and 1 atm (G_{b1}) by using zero-point energy and thermal energy corrections based on structural and vibration frequency data calculated at the same level.

Single point energy calculations were performed on the geometries obtained at the PBE0/b1 level using the same functional, the same basis set for the metal atoms and a 6-311++G(d,p) basis set[22] for the remaining elements (basis b2). Solvent effects (THF) were accounted for in all calculations (including geometry optimizations) by means of the Polarisable Continuum Model (PCM) initially devised by Tomasi and coworkers[23] with radii and non-electrostatic terms of the SMD solvation model, developed by Truhler *et al*.[24] The free energy values presented (G_{b2}-D3) were corrected for dispersion by means of Grimme DFT-D3 method[25] with Becke and Johnson short distance damping,[26] being derived from the electronic energy values obtained at the PBE0-D3/b2//PBE0/b1 level (E_{b2}-D3) according to the following expression:

$$(G_{b2}\text{-D3}) = (E_{b2}\text{-D3}) + G_{b1} - E_{b1}$$

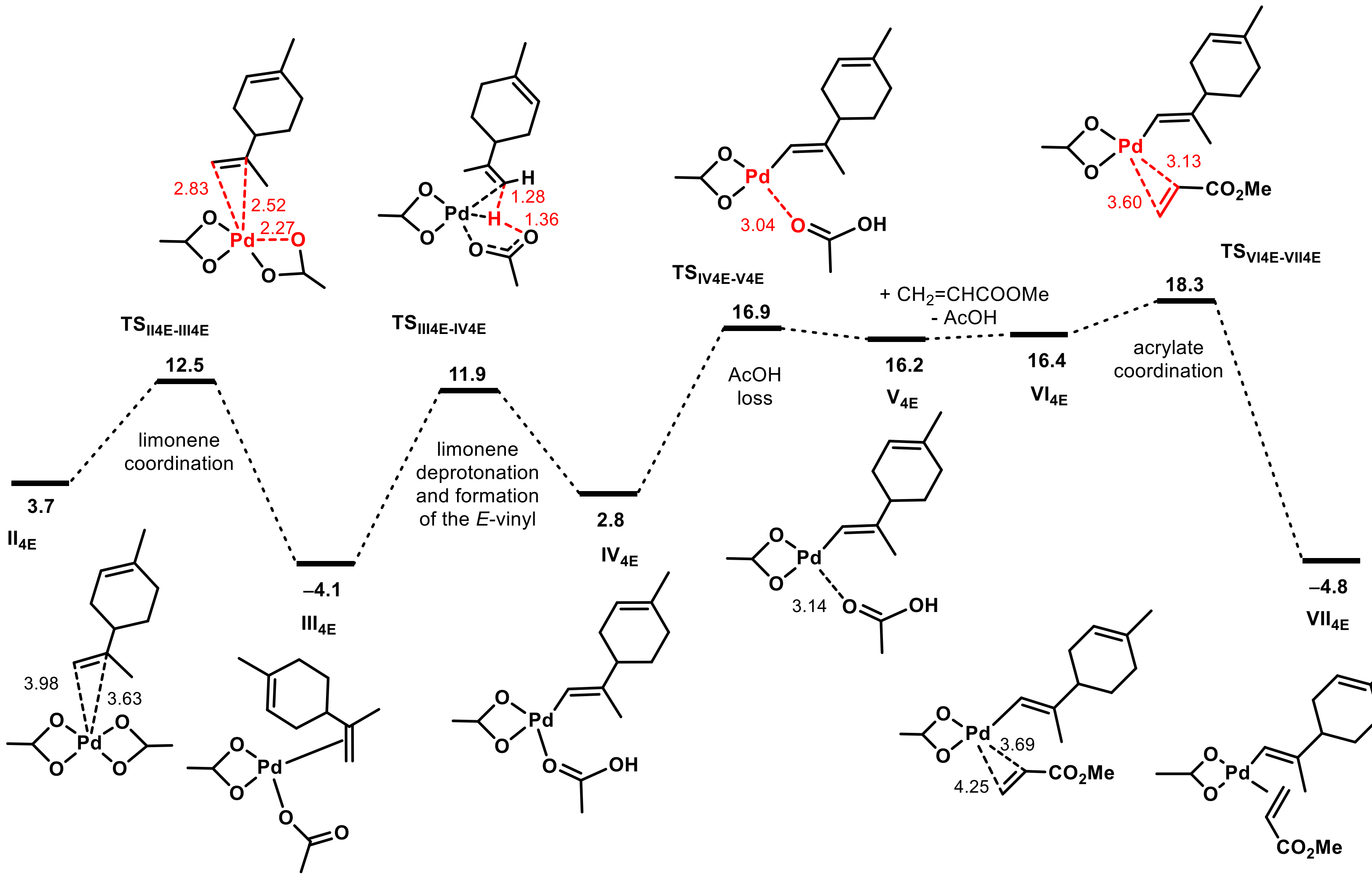

Figure S1. Free energy profile (kcal/mol) for the formation of vinyl intermediate **VII$_{4E}$**. Free energy values referred to [Pd(AcO)$_2$] (**I**) and distances in Å.

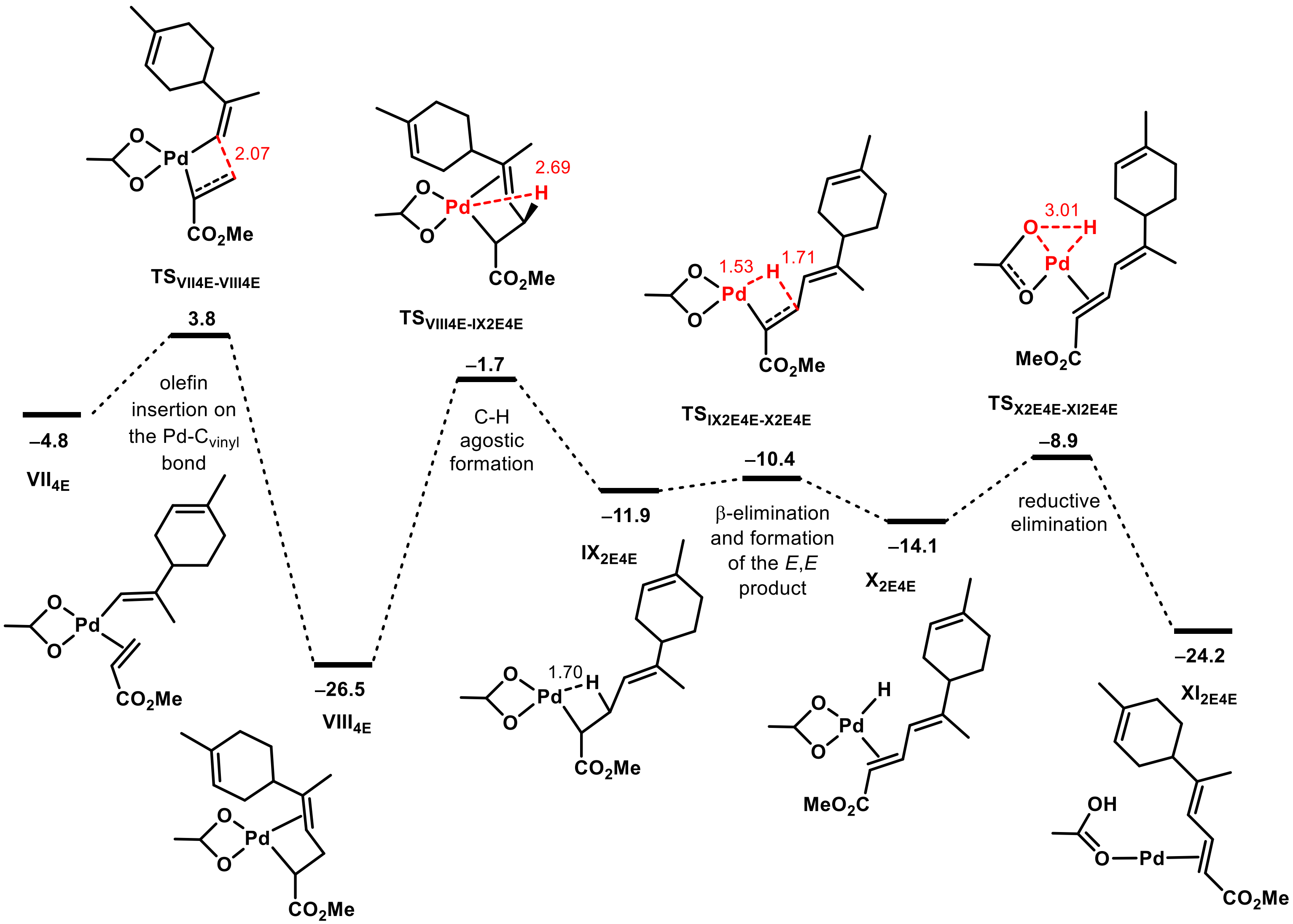

Figure S2. Free energy profile (kcal/mol) for the formation of product **2E,4E-3ab**. Free energy values referred to [Pd(AcO)$_2$] (**I**) and distances in Å.

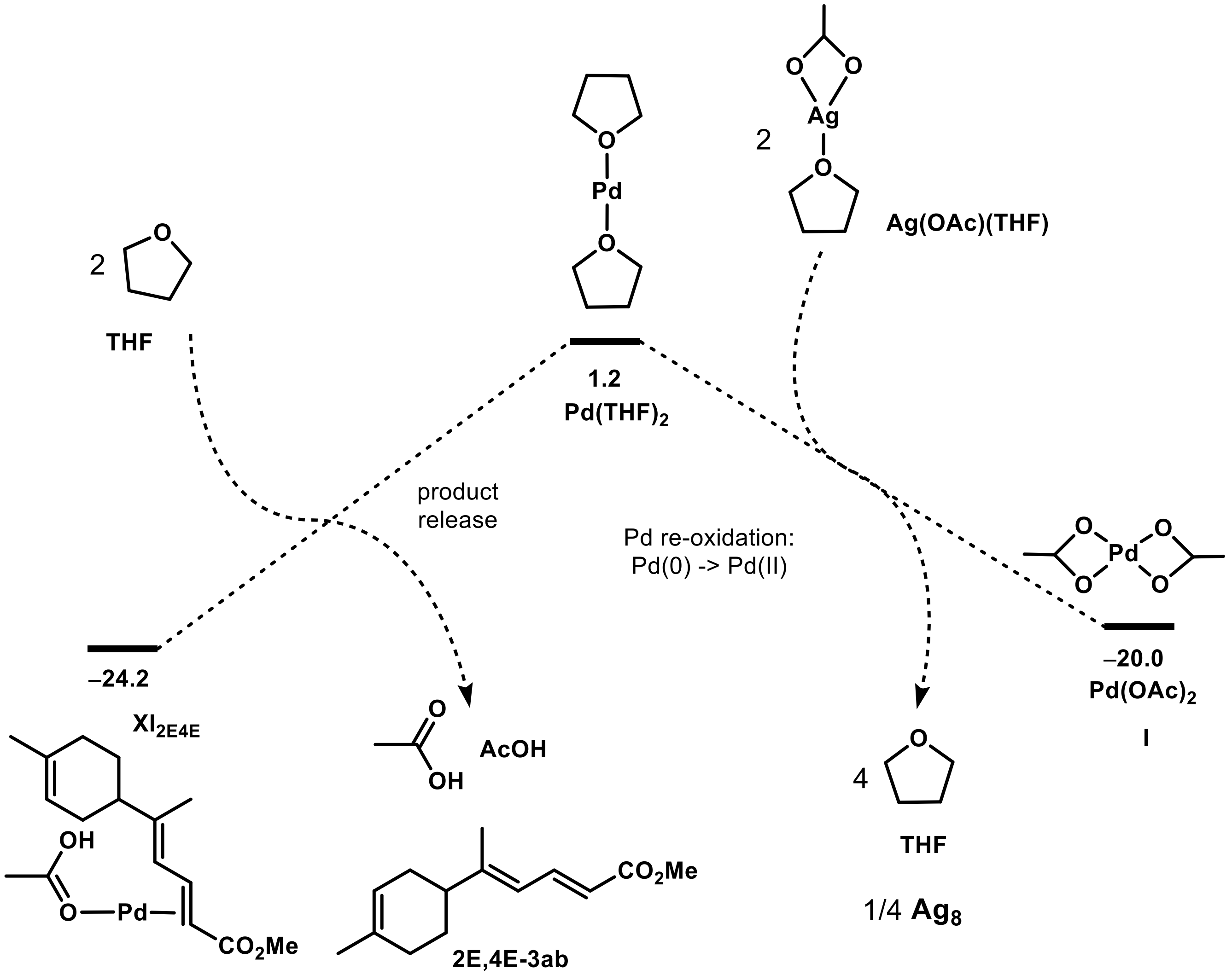

Figure S3. Cycle closure with the formation of product **2E,4E-3ab**. Free energy values (kcal/mol) referred to [Pd(AcO)$_2$] (**I**).

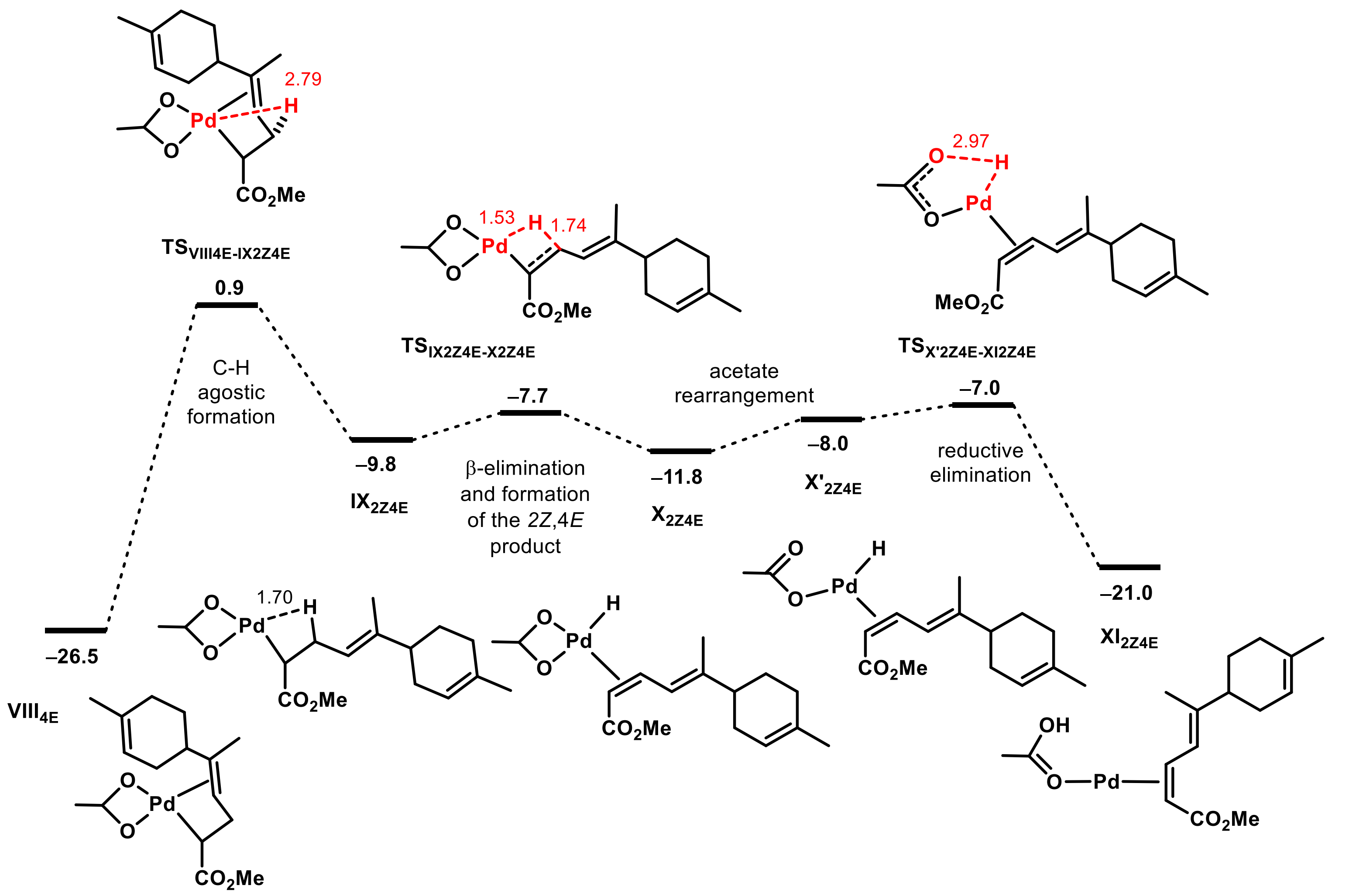

Figure S4. Free energy profile (kcal/mol) for the formation of product **2Z,4E-3ab** from intermediate **VIII$_{4E}$**. Free energy values referred to [Pd(AcO)$_2$] (**I**) and distances in Å.

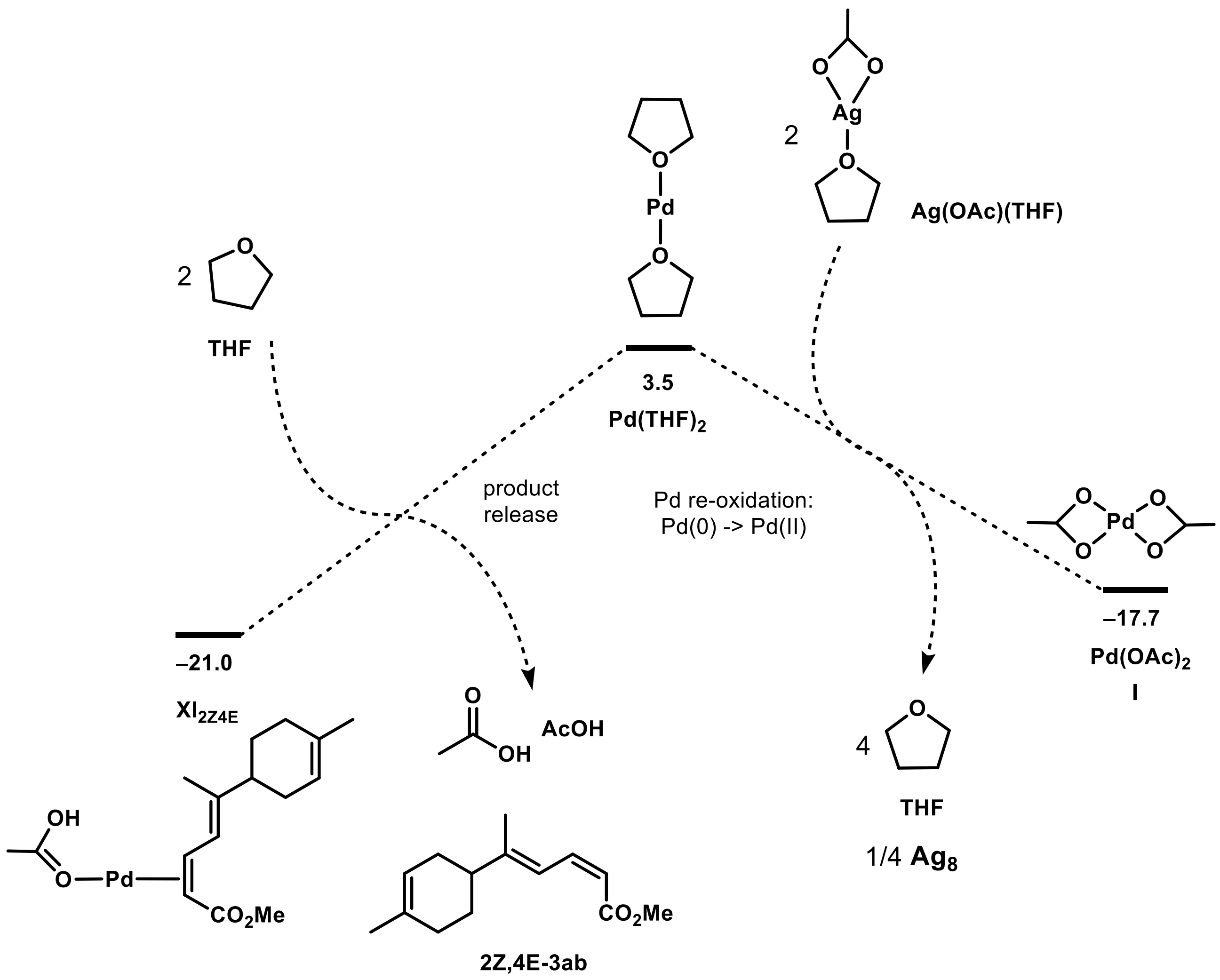

Figure S5. Cycle closure with the formation of product **2Z,4E-3ab**. Free energy values (kcal/mol) referred to [Pd(AcO)$_2$] (**I**).

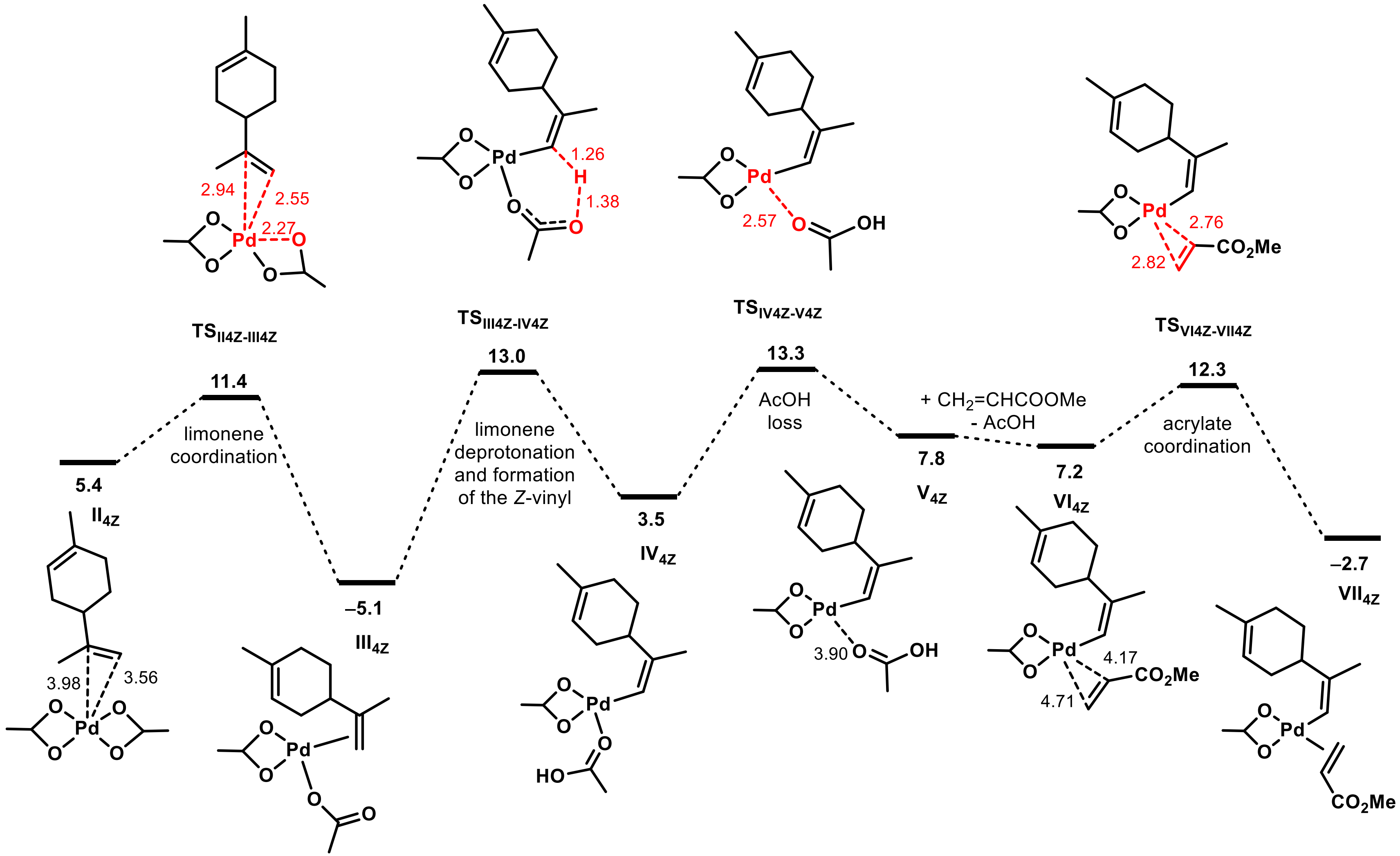

Figure S6. Free energy profile (kcal/mol) for the formation of vinyl intermediate **VII$_{4Z}$**. Free energy values referred to [Pd(AcO)$_2$] (**I**) and distances in Å.

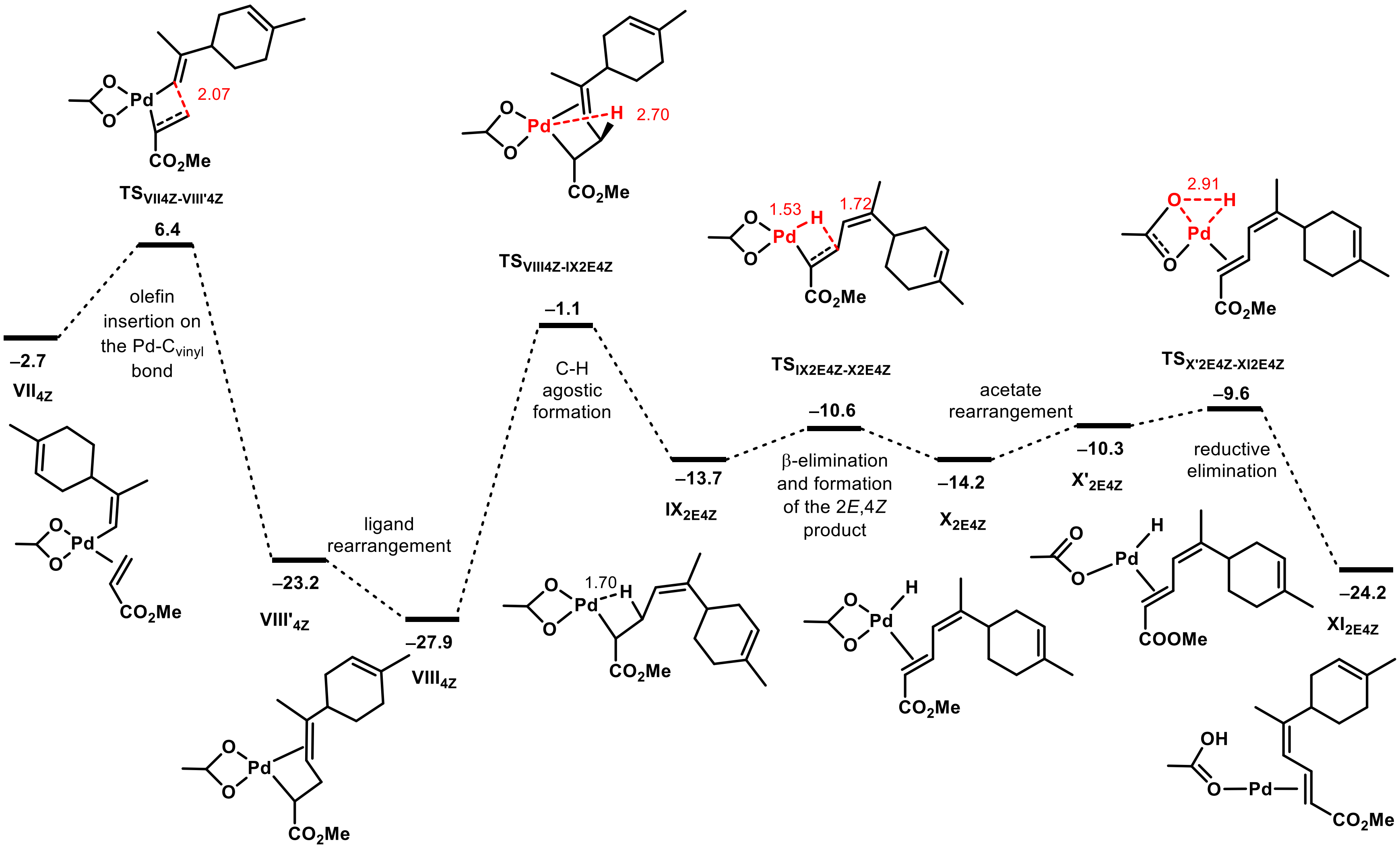

Figure S7. Free energy profile (kcal/mol) for the formation of product **2E,4Z-3ab**. Free energy values referred to [Pd(AcO)$_2$] (**I**) and distances in Å.

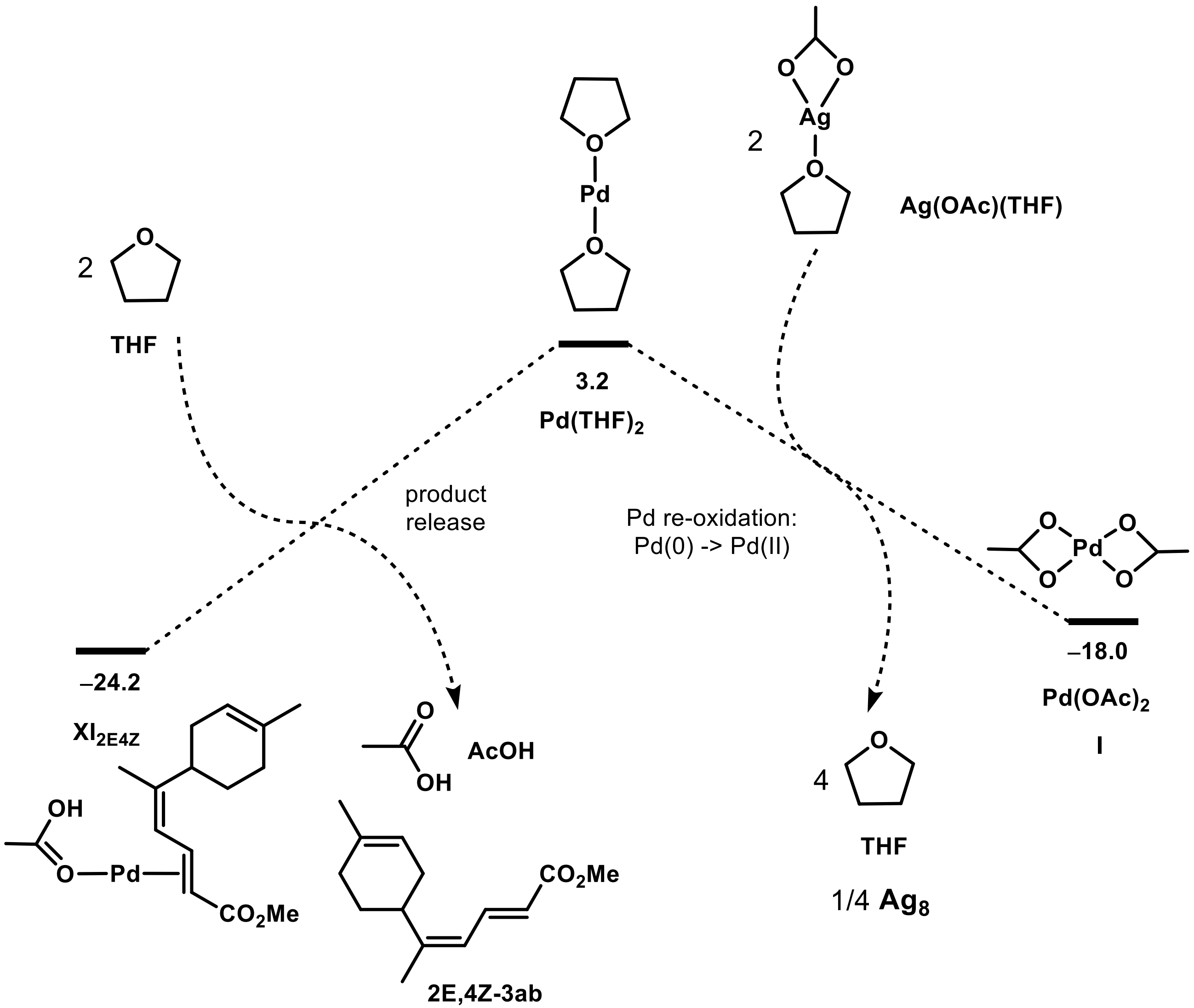

Figure S8. Cycle closure for the formation of product **2E,4Z-3ab**. Free energy values (kcal/mol) referred to [Pd(AcO)$_2$] (**I**).

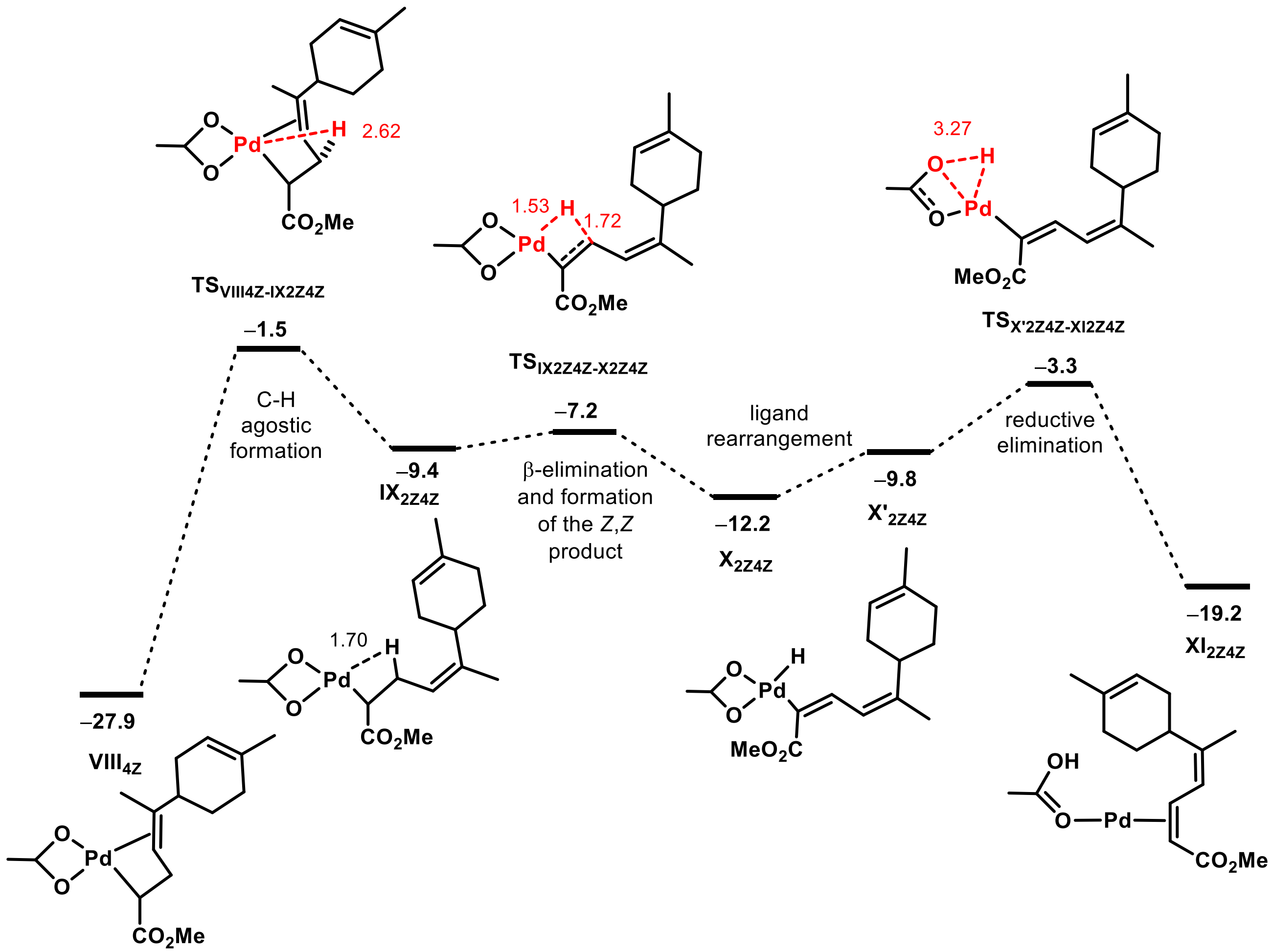

Figure S9. Free energy profile (kcal/mol) for the formation of product **2Z,4Z-3ab** from intermediate **VIII$_{4Z}$**. Free energy values referred to [Pd(AcO)$_2$] (**I**) and distances in Å.

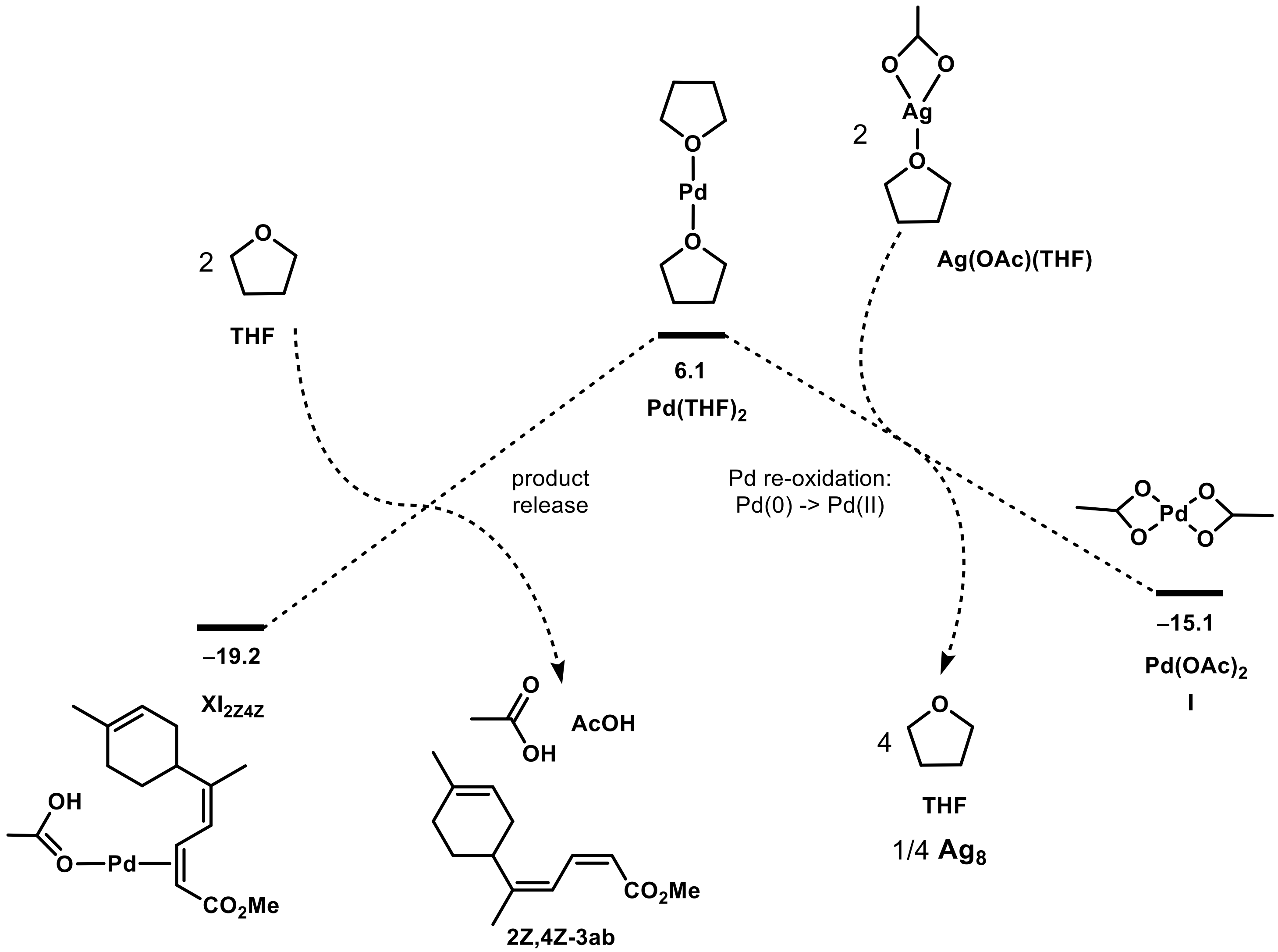

Figure S10. Cycle closure for the formation of product **2Z,4Z-3ab**. Free energy values (kcal/mol) referred to [Pd(AcO)$_2$] (**I**).

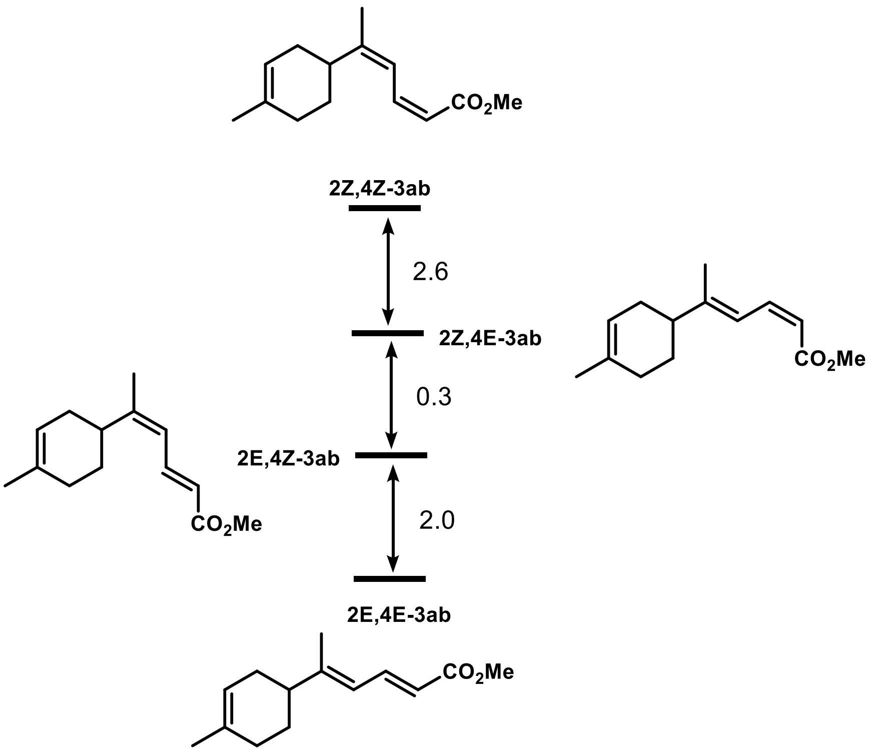

Figure S11. Relative stability (free energy, kcal/mol) of the product isomers.

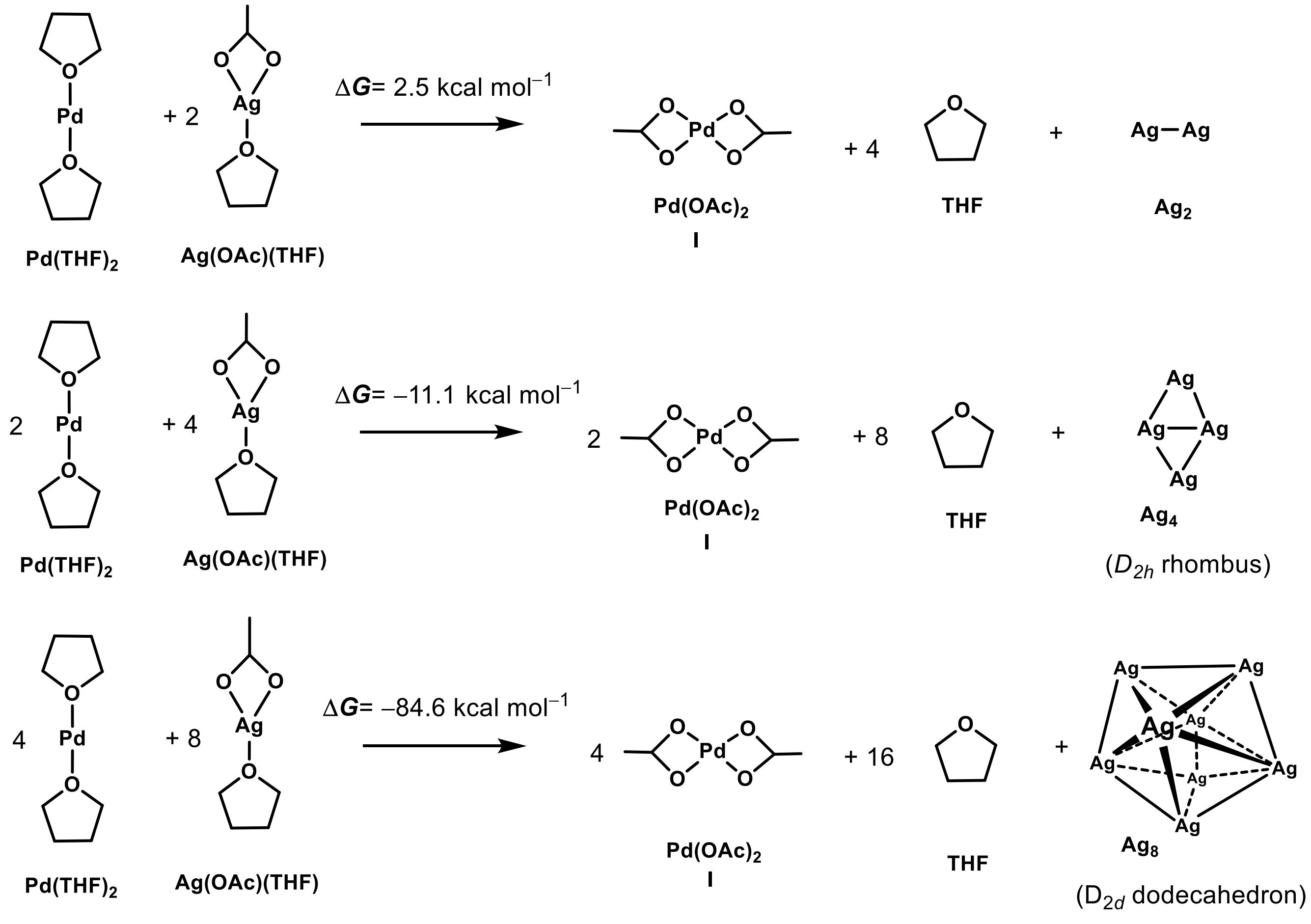

Figure S12. Reaction between $Pd(THF)_2$ and Ag(OAc)(THF) to give $Pd(OAc)_2$ and metallic Ag featuring three different oligomeric forms, and their associated Δ_rG.

12. Coordinates

```
   26
 Limonene
scf done:  -390.226531
 C    -0.828568     1.651394     0.156520
 C     0.353851     0.960481     0.829343
 C     0.808707    -0.255566     0.014475
 C    -0.339455    -1.274399    -0.049651
 C    -1.658287    -0.629998    -0.365433
 C    -1.894595     0.686555    -0.295481
 C    -3.218564     1.285598    -0.663118
 C     2.091134    -0.875190     0.525774
 C     3.118163    -1.101812    -0.299970
 C     2.170214    -1.226993     1.984529
 H    -2.464022    -1.293736    -0.681152
 H    -0.484478     2.235686    -0.710892
 H     1.181701     1.668143     0.955520
 H     0.057540     0.632846     1.834481
 H     0.995392     0.086750    -1.013128
 H    -0.415779    -1.821887     0.902145
 H    -0.109951    -2.035562    -0.806898
 H    -3.669810     1.804569     0.192850
 H    -3.102929     2.039016    -1.453811
 H    -3.925690     0.528919    -1.016158
 H     3.069186    -0.836619    -1.354173
 H     4.041366    -1.559324     0.049727
 H     3.102317    -1.750091     2.215693
 H     2.123496    -0.328654     2.612178
 H     1.335090    -1.866331     2.294852
 H    -1.273928     2.385904     0.841579

    8
 AcOH
scf done:  -228.847808
 H     1.830598     2.704495     2.158161
 H     2.391509     3.881692     0.970515
 C     2.878704     1.858744     0.503330
 H     0.991419     2.840215     0.585223
 C     1.957713     2.883765     1.086357
 O     2.614440     1.099729    -0.402189
 O     4.078725     1.865198     1.108487
 H     4.611292     1.179061     0.673517

   12
 Methyl acrylate
scf done:  -306.132140
 O     3.462241     0.403403     4.091372
 O     4.484397    -0.930326     5.590525
 H     2.443694    -1.212896     5.906893
 H     3.539846    -2.578130     6.278708
 H     3.147830    -2.170278     4.580696
```

```
 H     5.024742     2.384291     3.489767
 C     4.422165     0.141093     4.786502
 H     6.725492     2.637853     4.202663
 H     6.442393     0.570433     5.542270
 C     3.327591    -1.767768     5.581190
 C     5.817444     2.044683     4.151655
 C     5.668366     0.934245     4.872660

   13
 THF
scf done:  -232.194164
 C    -6.048477    -0.155575    -0.272602
 C    -6.677740    -0.152651     1.114167
 C    -8.142965    -0.415875     0.777086
 C    -8.301892     0.389143    -0.505758
 O    -7.038682     0.349154    -1.165800
 H    -5.767283    -1.176821    -0.573307
 H    -5.153257     0.473875    -0.341564
 H    -6.566864     0.832280     1.582274
 H    -6.236932    -0.900599     1.778874
 H    -8.838696    -0.102804     1.560352
 H    -8.301900    -1.482604     0.581261
 H    -8.568147     1.433957    -0.282206
 H    -9.067265    -0.016481    -1.178077

   27
 Pd(THF)2
scf done:  -592.266487
Pd    -0.902547     1.841994    -0.938832
 C     0.855569    -0.736462    -0.644457
 C     1.462354    -1.563963     0.472192
 C     0.335280    -1.566437     1.503798
 C    -0.225276    -0.156374     1.385831
 O     0.069348     0.261505     0.034280
 H     0.197723    -1.336959    -1.285532
 H     1.585113    -0.214789    -1.268562
 H     2.352914    -1.067836     0.873537
 H     1.742432    -2.565739     0.137084
 H     0.677157    -1.785131     2.518418
 H    -0.426084    -2.304647     1.229672
 H     0.268035     0.546479     2.066833
 H    -1.306217    -0.101822     1.538945
 C    -3.211871     3.383007    -2.412208
 C    -3.613191     4.835519    -2.622472
 C    -2.271600     5.513272    -2.896687
 C    -1.353454     4.779819    -1.937763
 O    -1.854686     3.429335    -1.919699
 H    -3.209457     2.812643    -3.348199
 H    -3.829478     2.859789    -1.677609
 H    -4.063723     5.243517    -1.711195
 H    -4.329565     4.946048    -3.440259
 H    -2.285952     6.589963    -2.710141
 H    -1.956990     5.342188    -3.932111
 H    -1.407670     5.194708    -0.923353
 H    -0.308164     4.741570    -2.253611
```

```
   21
 Ag(AcO)(THF)
scf done:  -607.431857
Ag    -0.311708    -0.257099     0.150181
 H    -2.337814     3.651810    -1.195779
 H    -0.764753     4.079785    -1.921462
 C    -0.864728     2.096608    -1.065641
 H    -1.880540     2.958921    -2.748727
 C    -1.489410     3.277105    -1.777219
 O    -1.643315     1.097226    -0.858525
 O     0.329862     2.126733    -0.716645
 C     2.174011    -1.722518     1.556365
 C     2.446866    -2.960096     2.389454
 C     1.538285    -3.994263     1.726146
 C     0.298100    -3.177560     1.407445
 O     0.781993    -1.832445     1.182963
 H     2.781199    -1.702388     0.643348
 H     2.303711    -0.780713     2.095881
 H     2.145919    -2.794589     3.429643
 H     3.502984    -3.239880     2.372754
 H     1.310282    -4.846439     2.370930
 H     1.996900    -4.368864     0.804661
 H    -0.404363    -3.149964     2.248449
 H    -0.229782    -3.507171     0.508677

   15
 Pd(OAc)2, I
scf done:  -584.399665
Pd     0.000045     0.000041     0.000036
 H     2.658200    -3.002780     1.812089
 H    -2.658665     3.003678    -1.809681
 H     1.392842    -4.035934     1.065290
 H    -1.390731     4.036358    -1.066607
 C     0.999812    -1.949333     1.005335
 C    -0.999679     1.949385    -1.005357
 H     1.111532    -3.267765     2.640664
 H    -1.113576     3.266215    -2.641826
 C     1.586067    -3.141756     1.660772
 C    -1.586072     3.141762    -1.660753
 O     1.475861    -0.777082     1.179061
 O    -1.475880     0.777153    -1.178838
 O    -0.031496    -2.025438     0.255911
 O     0.031738     2.025497    -0.256093

    2
 2
scf done:  -293.923882
Ag     0.000000     0.000000    -0.021262
Ag     0.000000     0.000000     2.561262

    4
 Ag4
scf done:  -587.881603
Ag    -1.650822    -0.411853    -0.386623
Ag     0.015392     1.574130    -0.775637
```

```
Ag    -0.081469    -0.450598    -2.659249
Ag    -1.553801     1.612721     1.497109


    8
 Ag8
scf done: -1175.869747
Ag     1.809839     0.039094     0.038110
Ag    -1.809983     0.038281     0.039447
Ag    -0.000623     2.149937    -0.427257
Ag     0.000385    -2.102810    -0.028884
Ag     0.000771    -0.426888     2.150097
Ag    -0.000845    -0.029437    -2.102691
Ag     1.459732     1.963323     1.962160
Ag    -1.459270     1.962680     1.963190

   36
 Product 2E,4E-3ab
scf done:  -695.187497
 C     2.366773     2.418699    -1.281241
 C     1.090834     1.889754    -0.632565
 C     1.343776     0.541617     0.037912
 C     1.775250    -0.481289    -1.030891
 C     2.789535     0.079060    -1.985126
 C     3.086331     1.379527    -2.100686
 C     4.142326     1.883937    -3.036854
 C     0.212283    -0.006309     0.877343
 C    -1.021136     0.552220     0.861029
 C     0.564224    -1.217213     1.690353
 H     3.317778    -0.641442    -2.610439
 H     3.051720     2.807202    -0.511949
 H     0.715268     2.618342     0.094655
 H     0.315272     1.775315    -1.401659
 H     2.202839     0.666227     0.717164
 H     0.887445    -0.825666    -1.584054
 H     2.189228    -1.376265    -0.550506
 H     3.716087     2.569449    -3.781085
 H     4.907383     2.455296    -2.494355
 H     4.641063     1.068003    -3.568400
 H    -1.190541     1.423793     0.232465
 H     0.633595    -2.107173     1.052302
 H     1.547857    -1.089493     2.156611
 H    -0.158442    -1.434428     2.478468
 H     2.129956     3.280707    -1.919543
 O    -4.467506    -0.809521     3.073281
 O    -5.625986     0.861066     2.101893
 H    -7.039127    -0.601408     2.559655
 H    -7.581464     1.103593     2.543034
 H    -6.601628     0.479252     3.904066
 C    -4.507484     0.144216     2.317540
 H    -2.071736    -0.766279     2.260855
 H    -3.577618     1.531108     0.895899
 C    -6.775215     0.425921     2.825708
 C    -2.169467     0.094706     1.602441
 C    -3.387176     0.670274     1.531178
```

36
Product 2Z,4E-3ab
scf done: -695.184138

C	2.851782	1.776816	-1.369343
C	1.585560	1.364671	-0.624059
C	1.696344	-0.073721	-0.124664
C	1.847014	-1.017982	-1.333297
C	2.830242	-0.503075	-2.344025
C	3.311433	0.746144	-2.367097
C	4.328813	1.197975	-3.370498
C	0.588819	-0.546789	0.788902
C	-0.532206	0.190743	0.979412
C	0.831592	-1.886220	1.420798
H	3.171865	-1.215232	-3.095914
H	3.666489	1.973462	-0.655486
H	1.406875	2.050875	0.211211
H	0.723618	1.451351	-1.298833
H	2.632154	-0.152740	0.452603
H	0.862830	-1.163950	-1.805435
H	2.164027	-2.012954	-0.997527
H	3.938122	2.017635	-3.987735
H	5.225683	1.588728	-2.871491
H	4.636742	0.385188	-4.035063
H	-0.631882	1.145023	0.474966
H	0.668351	-2.693733	0.696007
H	1.873614	-1.967806	1.750145
H	0.189718	-2.083446	2.280995
H	2.684922	2.730839	-1.887602
O	-2.475560	2.449799	0.653015
O	-4.361100	2.172426	1.846924
H	-4.132208	4.232089	1.622237
H	-5.795803	3.590080	1.771822
H	-4.885539	3.396741	0.243409
C	-3.143445	1.780515	1.421761
H	-1.565610	-1.146378	2.337104
H	-3.546850	0.064567	2.678215
C	-4.810787	3.424332	1.334122
C	-1.644895	-0.196752	1.811706
C	-2.795525	0.486480	2.017818

36
Product 2E,4Z-3ab
scf done: -695.184233

C	3.367786	1.549226	-1.045686
C	2.071536	1.381625	-0.259095
C	1.643799	-0.093585	-0.247952
C	1.374909	-0.574838	-1.682235
C	2.452255	-0.134089	-2.629901
C	3.365535	0.807682	-2.358083
C	4.445257	1.180664	-3.328695
C	0.524209	-0.412616	0.722454
C	-0.738429	0.077337	0.682944
C	0.905165	-1.362515	1.816527
H	2.482923	-0.633178	-3.598941
H	4.224419	1.206940	-0.444753
H	2.201859	1.746021	0.766107

```
 H      1.287638     1.995383    -0.716907
 H      2.520517    -0.657259     0.100860
 H      0.398255    -0.217617    -2.036054
 H      1.303024    -1.670665    -1.692182
 H      4.370286     2.237664    -3.616031
 H      5.438942     1.053460    -2.878678
 H      4.404318     0.574726    -4.238755
 H     -1.424010    -0.255502     1.462810
 H      1.262183    -2.311240     1.394305
 H      1.740629    -0.956701     2.402499
 H      0.075349    -1.574466     2.495968
 H      3.554037     2.615305    -1.234290
 O     -2.450734     2.838192    -2.159342
 O     -4.372920     2.681449    -0.994273
 H     -4.425891     4.547846    -1.921275
 H     -5.987024     3.738045    -1.590381
 H     -4.946368     3.181078    -2.935132
 C     -3.083207     2.375660    -1.227506
 H     -0.684616     1.378755    -1.082951
 H     -3.262685     1.108334     0.554053
 C     -4.958399     3.592774    -1.922051
 C     -1.301089     0.998998    -0.272263
 C     -2.576859     1.435007    -0.223116

   36
 Product 2Z,4Z-3ab
scf done:  -695.180515
 C      3.125019     1.735497    -1.098071
 C      1.776348     1.386179    -0.476645
 C      1.642214    -0.136510    -0.322281
 C      1.701712    -0.815201    -1.699878
 C      2.809424    -0.260347    -2.547290
 C      3.471185     0.874964    -2.286390
 C      4.599147     1.359760    -3.146494
 C      0.470069    -0.580249     0.531119
 C     -0.847051    -0.385980     0.272215
 C      0.862824    -1.300275     1.785119
 H      3.087785    -0.841431    -3.427074
 H      3.924759     1.643734    -0.347059
 H      1.670817     1.876070     0.497836
 H      0.972837     1.772288    -1.113936
 H      2.546593    -0.461381     0.211213
 H      0.743285    -0.715765    -2.227260
 H      1.847079    -1.895694    -1.567632
 H      4.370996     2.341690    -3.581480
 H      5.515605     1.490067    -2.555574
 H      4.817503     0.665595    -3.963505
 H     -1.566200    -0.769153     0.990464
 H      1.460102    -2.190309     1.546059
 H      1.504058    -0.662584     2.408399
 H     -0.001560    -1.609533     2.378390
 H      3.133448     2.790980    -1.402561
 O     -3.808116    -0.548715     0.741002
 O     -5.011966     0.430264    -0.886938
 H     -6.258234    -1.020921    -0.058704
 H     -7.026140     0.435696    -0.758985
```

```
 H    -6.197825     0.522514     0.824816
 C    -3.849395     0.057706    -0.315072
 H    -0.716617     0.686576    -1.609415
 H    -2.991385     1.019485    -2.053766
 C    -6.187750     0.064897    -0.168382
 C    -1.409169     0.287762    -0.873258
 C    -2.720500     0.490126    -1.145481

   41
 II(4E)
scf done:  -974.625127
Pd    -1.163467    -0.397960     1.068738
 H     2.748688    -1.908344     2.407124
 H    -4.922947     1.880509     1.328435
 H     2.580080    -2.700865     0.803242
 H    -4.081823     2.610222    -0.053632
 C     0.858049    -1.649755     1.472853
 C    -3.187947     0.847817     0.668220
 H     1.778257    -3.382070     2.232961
 H    -5.052874     1.146053    -0.305382
 C     2.076810    -2.450304     1.738733
 C    -4.396212     1.662103     0.397565
 O     0.368410    -0.852982     2.342773
 O    -2.703978     0.044443    -0.198476
 O     0.215157    -1.738434     0.373297
 O    -2.549639     0.929205     1.771055
 C     4.441688     0.810032     0.409042
 C     3.089746     1.517310     0.386555
 C     2.502007     1.529097    -1.022635
 C     3.447691     2.306686    -1.955745
 C     4.889873     1.943180    -1.750766
 C     5.356901     1.252015    -0.703008
 C     6.800843     0.873129    -0.567593
 C     1.083877     2.048075    -1.126065
 C     0.441311     2.638506    -0.109906
 C     0.417129     1.868969    -2.461425
 H     5.587827     2.263435    -2.525264
 H     4.301589    -0.280389     0.345086
 H     2.402479     1.028887     1.086758
 H     3.215459     2.551665     0.734352
 H     2.485875     0.487363    -1.383561
 H     3.306780     3.387638    -1.797178
 H     3.178352     2.125871    -3.003820
 H     7.241627     1.311765     0.337441
 H     6.913929    -0.214915    -0.468839
 H     7.391748     1.198948    -1.429004
 H     0.898555     2.788001     0.864078
 H    -0.575424     3.006704    -0.231563
 H    -0.630345     2.182465    -2.430706
 H     0.919250     2.449381    -3.245003
 H     0.453678     0.818991    -2.778982
 H     4.937992     0.981651     1.374151

   41
 TS(II4E-III4E)
scf done:  -974.612520
```

Pd	-0.703696	0.133748	0.671949
H	2.795740	-2.073296	2.200450
H	-4.913105	1.760576	1.258508
H	2.706273	-2.819452	0.571732
H	-4.184869	2.763671	-0.014224
C	1.060614	-1.563656	1.059563
C	-2.986742	1.133740	0.598172
H	1.670974	-3.412335	1.877692
H	-4.852507	1.178381	-0.436820
C	2.135602	-2.515738	1.452586
C	-4.324388	1.736019	0.339913
O	0.799730	-0.546834	1.807033
O	-2.329094	0.618327	-0.386470
O	0.360803	-1.723314	0.024524
O	-2.447158	1.130218	1.732253
C	4.458833	0.939313	0.578046
C	3.079386	1.591443	0.550739
C	2.397894	1.364423	-0.796879
C	3.241993	2.023552	-1.904244
C	4.708812	1.743010	-1.752525
C	5.276479	1.234429	-0.651507
C	6.741048	0.926751	-0.569894
C	0.957404	1.820059	-0.883088
C	0.292532	2.392720	0.152746
C	0.296692	1.681424	-2.225475
H	5.335988	1.965114	-2.616593
H	4.359897	-0.151790	0.690333
H	2.469826	1.191018	1.365758
H	3.183028	2.672181	0.719434
H	2.400751	0.280987	-1.001472
H	3.066215	3.111139	-1.901851
H	2.912124	1.673954	-2.889889
H	7.223863	1.510336	0.225067
H	6.906815	-0.129889	-0.320361
H	7.256420	1.138217	-1.511709
H	0.773844	2.601845	1.103755
H	-0.691993	2.828031	-0.002769
H	-0.782528	1.834950	-2.154710
H	0.700178	2.418025	-2.930807
H	0.482925	0.692296	-2.660060
H	5.010897	1.272508	1.467497

41

III(4E)

scf done: -974.639695

Pd	-0.150456	0.236347	0.318781
H	2.294362	-3.237586	1.598338
H	-4.548602	0.456745	-0.587618
H	0.722565	-4.040695	1.272251
H	-4.838730	1.574154	0.769952
C	0.684071	-1.916292	1.144270
C	-2.789121	1.002178	0.500109
H	0.991358	-3.183726	2.801411
H	-4.103893	2.156111	-0.753819
C	1.212088	-3.177836	1.727927
C	-4.158610	1.324597	-0.045631

O	1.415244	-0.865694	1.088126
O	-1.884137	0.852525	-0.433497
O	-0.506292	-1.824388	0.713250
O	-2.563085	0.883536	1.700965
C	4.646665	1.065667	0.506157
C	3.225030	1.613407	0.582876
C	2.516724	1.470791	-0.763548
C	3.269721	2.320910	-1.807270
C	4.756962	2.132249	-1.725790
C	5.398057	1.549642	-0.704958
C	6.882186	1.342598	-0.699428
C	1.040515	1.831078	-0.764450
C	0.389683	2.294284	0.378208
C	0.388382	1.965754	-2.110975
H	5.333859	2.492259	-2.578158
H	4.629514	-0.034916	0.505814
H	2.679817	1.093468	1.373767
H	3.259869	2.678526	0.850459
H	2.595728	0.422955	-1.090818
H	3.018467	3.384490	-1.669707
H	2.934598	2.064313	-2.818590
H	7.351162	1.870192	0.141527
H	7.129252	0.280046	-0.573080
H	7.347234	1.692157	-1.626091
H	0.931973	2.450439	1.306937
H	-0.557696	2.817750	0.279055
H	-0.696963	2.027226	-2.022421
H	0.746784	2.883989	-2.592501
H	0.651891	1.129617	-2.766962
H	5.201320	1.347230	1.411531

41
TS(III4E-IV4E)
scf done: -974.610053

Pd	-0.651475	-0.527527	0.744920
H	0.961121	-4.394394	2.171146
H	-4.147927	2.587698	0.593468
H	-0.796287	-4.753251	2.218666
H	-3.531070	3.255528	-0.926799
C	-0.307461	-2.724791	1.776918
C	-2.312143	1.738952	-0.032566
H	-0.023860	-3.853281	3.540047
H	-4.278089	1.623966	-0.889358
C	-0.024090	-4.019825	2.456903
C	-3.652100	2.336707	-0.350884
O	0.643019	-1.894579	1.553378
O	-2.263480	0.468830	0.065060
O	-1.481181	-2.394048	1.431798
O	-1.328280	2.501668	0.142849
C	5.217210	1.188181	-0.210006
C	3.757242	0.940289	0.158054
C	2.847438	1.344515	-1.001161
C	2.984142	2.861783	-1.240658
C	4.423700	3.288431	-1.271747
C	5.445281	2.542485	-0.831043
C	6.870276	2.997148	-0.925907

C	1.394109	0.972387	-0.850240
C	0.762514	0.908247	0.362858
C	0.662138	0.749265	-2.132081
H	4.629102	4.271893	-1.695314
H	5.573401	0.410773	-0.903180
H	3.604842	-0.112982	0.419130
H	3.503879	1.532866	1.046042
H	3.212256	0.845843	-1.911872
H	2.444356	3.404638	-0.449926
H	2.496765	3.141190	-2.183045
H	7.326905	3.074439	0.069580
H	7.475976	2.273734	-1.487898
H	6.957143	3.969731	-1.419475
H	1.372433	1.070078	1.254076
H	-0.246499	1.684468	0.289038
H	-0.420039	0.698513	-1.993401
H	0.906415	1.527500	-2.864847
H	0.991566	-0.202493	-2.569905
H	5.848250	1.091531	0.683784

41
IV(4E)
scf done: -974.626777

Pd	-0.610263	-0.668303	0.947079
H	-0.194826	-5.083976	1.106156
H	-2.546081	3.948867	0.955301
H	-1.875640	-4.950710	1.715498
H	-3.120872	3.378110	-0.612964
C	-0.867487	-3.084632	1.455390
C	-1.764995	2.073942	0.365506
H	-0.495304	-4.678369	2.801146
H	-3.751768	2.630487	0.887765
C	-0.865234	-4.543708	1.778595
C	-2.876190	3.061975	0.405043
O	0.239119	-2.521703	1.122877
O	-1.869082	0.965034	0.913889
O	-1.914600	-2.386752	1.525007
O	-0.696873	2.464674	-0.271146
C	5.113896	2.009717	0.171250
C	3.694656	1.522508	0.450336
C	3.007422	1.077668	-0.838353
C	2.906308	2.277347	-1.796665
C	4.181471	3.066696	-1.869178
C	5.196353	2.949269	-1.003309
C	6.469066	3.728154	-1.144903
C	1.663320	0.386608	-0.662567
C	1.020798	0.333582	0.521223
C	1.102207	-0.242609	-1.910379
H	4.271720	3.771278	-2.696737
H	5.780917	1.152538	-0.008829
H	3.720764	0.701240	1.175409
H	3.115187	2.335487	0.908878
H	3.665849	0.338689	-1.324192
H	2.080678	2.933791	-1.477850
H	2.638283	1.936683	-2.804273
H	6.638201	4.371144	-0.271044

```
 H     7.335771     3.056271    -1.205207
 H     6.465151     4.358888    -2.039038
 H     1.464399     0.801930     1.404850
 H     0.009744     1.757392    -0.199705
 H     0.182330    -0.794689    -1.696504
 H     0.878097     0.509310    -2.677052
 H     1.829425    -0.935308    -2.352363
 H     5.519080     2.509087     1.061961

   41
 TS(IV4E-V4E)
scf done:  -974.598092
Pd     0.023742    -1.504917    -0.094688
 H    -1.123638    -4.672808     2.804940
 H    -2.447076     2.707819     2.112804
 H    -2.617744    -4.677971     1.815829
 H    -1.886498     3.871378     0.911700
 C    -1.222997    -3.134807     1.325194
 C    -1.370283     1.842685     0.493008
 H    -2.369886    -3.451182     3.081339
 H    -3.271900     2.804561     0.531077
 C    -1.874385    -4.051258     2.310460
 C    -2.310373     2.871002     1.039047
 O    -0.145939    -2.524702     1.678432
 O    -1.658963     1.016795    -0.353269
 O    -1.702047    -2.928108     0.181328
 O    -0.156180     1.921768     1.042615
 C     5.355508     1.945965    -0.345430
 C     4.102182     1.152098     0.011646
 C     3.155028     1.062919    -1.181748
 C     2.708182     2.476616    -1.591203
 C     3.852934     3.446981    -1.630873
 C     5.057361     3.221422    -1.090057
 C     6.181298     4.207614    -1.193356
 C     1.969509     0.157010    -0.962677
 C     1.640713    -0.490170     0.161214
 C     1.027983    -0.056931    -2.131750
 H     3.673362     4.391028    -2.146287
 H     6.035360     1.328981    -0.952935
 H     4.380791     0.149902     0.356203
 H     3.580725     1.643081     0.844515
 H     3.717458     0.644130    -2.032952
 H     1.937157     2.834693    -0.890687
 H     2.222587     2.449249    -2.574865
 H     6.505386     4.542565    -0.199183
 H     7.061155     3.751812    -1.666946
 H     5.899418     5.089103    -1.777073
 H     2.189417    -0.511831     1.102450
 H     0.399787     1.216514     0.646523
 H     0.279647    -0.879484    -1.994684
 H     0.442172     0.835788    -2.369251
 H     1.581046    -0.366069    -3.026373
 H     5.918997     2.184369     0.566863

   41
 V(4E)
```

```
scf done:  -974.598114
Pd     0.064264    -1.539277    -0.104447
 H    -1.206231    -4.643502     2.808751
 H    -2.439791     2.687469     2.150038
 H    -2.680667    -4.631050     1.791206
 H    -1.840923     3.880404     0.997281
 C    -1.243102    -3.125281     1.307350
 C    -1.363714     1.857462     0.510075
 H    -2.430125    -3.391968     3.045430
 H    -3.244437     2.859163     0.563950
 C    -1.934153    -4.012950     2.291707
 C    -2.287610     2.885572     1.084346
 O    -0.177115    -2.509477     1.684375
 O    -1.655736     1.076559    -0.375751
 O    -1.676884    -2.947792     0.140739
 O    -0.157683     1.883586     1.084299
 C     5.356434     1.965560    -0.352527
 C     4.111691     1.162886     0.014836
 C     3.159719     1.059731    -1.173576
 C     2.698070     2.467289    -1.587078
 C     3.835018     3.446185    -1.639868
 C     5.044197     3.233330    -1.104517
 C     6.160244     4.226901    -1.221748
 C     1.985142     0.143400    -0.943189
 C     1.667345    -0.509900     0.179507
 C     1.034002    -0.080497    -2.103689
 H     3.645433     4.385134    -2.160944
 H     6.039346     1.350617    -0.958660
 H     4.400394     0.164851     0.363130
 H     3.589656     1.653578     0.847494
 H     3.721632     0.642148    -2.025741
 H     1.929155     2.822444    -0.882792
 H     2.205980     2.431015    -2.567212
 H     6.487844     4.571547    -0.232063
 H     7.040519     3.773964    -1.697307
 H     5.868482     5.101962    -1.810262
 H     2.212874    -0.528915     1.122370
 H     0.388739     1.182480     0.669309
 H     0.281220    -0.902386    -1.966685
 H     0.442504     0.808862    -2.338891
 H     1.579307    -0.394581    -3.001187
 H     5.921456     2.215084     0.555839

   45
 VI(4E)
scf done: -1051.875797
Pd    -0.720758    -0.901283     2.696027
 H    -2.510587    -4.048000     5.243758
 H    -1.779035    -3.085754     6.549484
 C    -1.283427    -2.456496     4.567351
 H    -0.795085    -4.255645     5.614427
 C    -1.604439    -3.523006     5.565379
 O    -0.766005    -2.809051     3.443744
 O    -1.522973    -1.241291     4.784648
 C     1.258278    -0.427141    -3.300370
 C     0.900009    -0.833657    -1.874009
```

C	0.701494	0.394192	-0.988628
C	2.011892	1.196733	-0.926005
C	2.644058	1.361481	-2.278034
C	2.309124	0.651058	-3.363118
C	2.943957	0.881321	-4.701301
C	0.181756	0.071213	0.389032
C	0.032616	-1.130806	0.944358
C	-0.249221	1.234916	1.266130
H	3.422263	2.120613	-2.363717
H	0.359722	-0.080217	-3.833675
H	-0.004660	-1.452591	-1.876164
H	1.706930	-1.449161	-1.453968
H	-0.049819	1.041883	-1.471019
H	2.713862	0.694412	-0.241747
H	1.830068	2.186882	-0.489289
H	3.461659	-0.020781	-5.053216
H	2.185948	1.117263	-5.460232
H	3.666271	1.702803	-4.675260
H	0.263167	-2.111023	0.530821
H	-0.640927	0.985120	2.293577
H	0.581165	1.915730	1.480646
H	-1.064674	1.805562	0.808003
H	1.607385	-1.303867	-3.862784
O	-4.110785	-2.286774	1.314086
O	-3.656219	-0.809693	-0.321256
H	-2.594815	-2.527225	-0.832722
H	-3.163360	-1.472805	-2.163556
H	-4.316191	-2.530724	-1.295102
H	-3.904410	1.559047	0.373171
C	-3.982131	-1.139173	0.935080
H	-4.288143	2.120725	2.101303
H	-4.411671	-0.221666	2.845575
C	-3.420453	-1.911224	-1.198972
C	-4.117653	1.302593	1.406868
C	-4.182198	0.033304	1.814579

45
TS(VI4E-VII4E)
scf done: -1051.875411

Pd	-0.899778	-0.771324	2.635017
H	-2.490328	-3.991222	5.227034
H	-1.775174	-2.998107	6.515385
C	-1.348161	-2.350191	4.523735
H	-0.758985	-4.126674	5.562993
C	-1.599223	-3.429429	5.528655
O	-0.758152	-2.664824	3.425541
O	-1.719379	-1.163118	4.712203
C	1.346500	-0.445245	-3.282921
C	0.927042	-0.793259	-1.857621
C	0.723668	0.469434	-1.023796
C	2.052279	1.239637	-0.937758
C	2.738374	1.342953	-2.269458
C	2.424231	0.606137	-3.343044
C	3.111288	0.778132	-4.664102
C	0.139506	0.215836	0.345006
C	-0.049504	-0.970807	0.925186

C	-0.300940	1.425500	1.146679
H	3.537598	2.080450	-2.350878
H	0.476392	-0.097518	-3.860972
H	0.010496	-1.394067	-1.872500
H	1.704686	-1.409865	-1.386965
H	0.010711	1.117516	-1.560835
H	2.714045	0.742883	-0.210685
H	1.882421	2.248822	-0.542120
H	3.620275	-0.146073	-4.968143
H	2.386448	1.005856	-5.457176
H	3.851039	1.583982	-4.638080
H	0.203458	-1.956805	0.537568
H	-0.642897	1.207111	2.189060
H	0.520872	2.134946	1.293715
H	-1.131473	1.953644	0.664520
H	1.695350	-1.348738	-3.801508
O	-4.037877	-2.542055	1.206573
O	-3.817065	-0.879396	-0.292871
H	-2.957829	-2.534627	-1.221797
H	-3.742528	-1.310077	-2.264199
H	-4.736337	-2.451672	-1.308200
H	-3.866375	1.390560	0.716301
C	-3.929893	-1.358735	0.952446
H	-3.923443	1.734496	2.541081
H	-3.979383	-0.680871	3.006334
C	-3.814873	-1.863873	-1.327921
C	-3.900879	1.008862	1.732513
C	-3.925718	-0.302387	1.989409

45
VII(4E)
scf done: -1051.916247

Pd	-1.936516	-1.071761	2.397878
H	-0.017544	-3.603565	5.532492
H	-1.016844	-2.583522	6.597736
C	-0.989536	-2.002515	4.535795
H	0.567372	-2.003828	5.994768
C	-0.330957	-2.576742	5.749610
O	-0.268374	-1.739714	3.507707
O	-2.233854	-1.790354	4.499482
C	0.917524	-0.064597	-3.219086
C	0.198401	-0.436221	-1.925141
C	0.501303	0.576282	-0.823271
C	2.011648	0.561715	-0.531263
C	2.841496	0.556965	-1.782674
C	2.364861	0.296259	-3.006846
C	3.224827	0.348370	-4.233453
C	-0.305872	0.419364	0.457271
C	-1.041802	-0.669203	0.703368
C	-0.194309	1.569180	1.423189
H	3.899740	0.794600	-1.667711
H	0.406374	0.778125	-3.709928
H	-0.881200	-0.497831	-2.103573
H	0.526698	-1.433496	-1.602014
H	0.262736	1.576534	-1.221509
H	2.252914	-0.320480	0.083059

H	2.288354	1.430048	0.079515
H	3.258022	-0.627845	-4.735106
H	2.819989	1.058132	-4.967435
H	4.250888	0.648898	-4.000611
H	-1.126622	-1.496387	-0.003775
H	-0.876609	1.453355	2.270702
H	0.822536	1.656995	1.825166
H	-0.419301	2.520460	0.923943
H	0.854640	-0.896668	-3.933845
O	-4.111688	-2.895678	0.051304
O	-3.885897	-0.806763	-0.754456
H	-3.112292	-2.061914	-2.231577
H	-3.808284	-0.494192	-2.749618
H	-4.880396	-1.841928	-2.263410
H	-3.251977	0.889003	1.047390
C	-3.970714	-1.703610	0.228219
H	-3.819980	0.682593	2.799100
H	-4.442441	-1.677148	2.332933
C	-3.923653	-1.346053	-2.079838
C	-3.544612	0.222001	1.852404
C	-3.908568	-1.091062	1.586512

45
TS(VII4E-VIII4E)
scf done: -1051.905280

Pd	-1.953711	-1.075199	1.872767
H	-0.378994	-4.436750	4.322538
H	-0.801537	-3.291906	5.605393
C	-0.876543	-2.503219	3.618096
H	0.787261	-3.181946	4.780740
C	-0.275273	-3.397471	4.654497
O	-0.165840	-2.019376	2.685680
O	-2.124168	-2.243801	3.644877
C	1.474352	0.588311	-3.243029
C	0.522995	0.145097	-2.134946
C	0.703076	1.007528	-0.887761
C	2.138020	0.833599	-0.358887
C	3.164558	0.889698	-1.452989
C	2.885559	0.799979	-2.759825
C	3.941796	0.908164	-3.817763
C	-0.316448	0.792529	0.215804
C	-1.173062	-0.242860	0.199716
C	-0.290891	1.806042	1.323323
H	4.201295	1.023107	-1.141921
H	1.110799	1.518470	-3.706528
H	-0.511833	0.195371	-2.492698
H	0.724136	-0.904894	-1.882434
H	0.605112	2.061382	-1.198269
H	2.213052	-0.124998	0.178817
H	2.363130	1.607438	0.385366
H	3.992711	-0.008078	-4.420735
H	3.715442	1.724684	-4.516616
H	4.931822	1.092175	-3.389673
H	-1.205449	-0.948388	-0.628640
H	-1.124461	1.670704	2.018674
H	0.633806	1.724745	1.908228

H	-0.321432	2.827703	0.923319
H	1.477786	-0.155486	-4.051520
O	-5.576097	-1.998103	2.024369
O	-4.523744	-2.116987	0.037787
H	-5.143623	-4.069007	0.422157
H	-5.036231	-3.551481	-1.288085
H	-6.391423	-2.993942	-0.259940
H	-3.313685	-0.024023	-0.510152
C	-4.744849	-1.578669	1.246078
H	-2.956210	1.329528	0.676202
H	-4.158077	0.123898	2.436103
C	-5.329902	-3.253400	-0.281593
C	-3.153262	0.274001	0.521258
C	-3.872982	-0.410368	1.531113

45
VIII(4E)
scf done: -1051.955423

Pd	-1.476342	-0.808011	1.007529
H	0.067990	-4.498303	3.035801
H	1.097713	-3.242474	3.732324
C	-0.054152	-2.591570	2.075852
H	1.564186	-3.990382	2.189108
C	0.718072	-3.654990	2.790904
O	0.454440	-1.943167	1.118088
O	-1.242617	-2.317082	2.461149
C	1.792329	0.074768	-2.657868
C	0.498617	-0.121554	-1.870676
C	0.273507	1.028725	-0.889709
C	1.442775	1.105513	0.105626
C	2.779336	0.919446	-0.549301
C	2.961931	0.470928	-1.797467
C	4.320120	0.343601	-2.418301
C	-1.077299	1.059336	-0.186181
C	-2.164717	0.334254	-0.666600
C	-1.246759	2.216557	0.767086
H	3.651315	1.183015	0.050331
H	1.648025	0.837737	-3.438575
H	-0.331495	-0.201593	-2.579956
H	0.543364	-1.064467	-1.311061
H	0.290790	1.964702	-1.474572
H	1.304633	0.344953	0.888183
H	1.434618	2.074326	0.619661
H	4.532015	-0.697588	-2.695169
H	4.384557	0.928407	-3.345785
H	5.111149	0.686743	-1.744624
H	-2.031969	-0.312929	-1.530299
H	-2.272348	2.334329	1.118418
H	-0.598973	2.119005	1.643222
H	-0.954468	3.142406	0.254209
H	2.036996	-0.849257	-3.198957
O	-4.598631	-1.533884	2.772912
O	-4.514845	-2.104131	0.599751
H	-4.770565	-3.901802	1.621900
H	-5.458654	-3.775993	-0.025460
H	-6.259390	-2.962037	1.352378

H	-4.272748	-0.076737	-0.727434
C	-4.217291	-1.304924	1.641666
H	-3.906945	1.530463	-0.075105
H	-3.344611	0.570297	2.086929
C	-5.299125	-3.253093	0.918022
C	-3.568104	0.490173	-0.117035
C	-3.379441	-0.139916	1.257986

45
TS(VIII4E-IX2E4E)
scf done: -1051.915739

Pd	-1.774505	-2.547500	0.849847
H	0.933990	-5.727664	2.625718
H	-0.576799	-5.849172	3.553643
C	-0.513240	-4.213009	2.210186
H	0.607355	-4.609595	3.988875
C	0.168007	-5.154250	3.150158
O	-0.497226	-4.384799	0.962982
O	-1.157826	-3.214376	2.699190
C	2.188480	0.865076	-2.889414
C	0.905572	0.374668	-2.223390
C	0.409372	1.383071	-1.190188
C	1.473427	1.538428	-0.088406
C	2.863627	1.664190	-0.640710
C	3.204393	1.383735	-1.905138
C	4.598786	1.568072	-2.423136
C	-0.951051	1.101523	-0.588678
C	-1.614624	-0.047022	-0.806182
C	-1.500636	2.197377	0.281158
H	3.631909	2.019096	0.047250
H	1.958930	1.657655	-3.618346
H	0.139293	0.193875	-2.985912
H	1.099090	-0.587602	-1.729523
H	0.334489	2.360644	-1.694899
H	1.417259	0.675070	0.594294
H	1.250506	2.416652	0.530363
H	5.019524	0.616101	-2.773060
H	4.610401	2.245990	-3.287212
H	5.267493	1.978480	-1.660435
H	-1.161007	-0.796599	-1.453115
H	-2.576954	2.101109	0.446953
H	-1.019504	2.202804	1.267491
H	-1.310143	3.180240	-0.166021
H	2.637965	0.053071	-3.477209
O	-4.248620	-1.243614	3.127018
O	-5.004030	-1.825130	1.089483
H	-6.002632	-3.093294	2.408260
H	-6.849803	-2.623823	0.903279
H	-6.694057	-1.459054	2.253472
H	-3.410253	-1.220568	-0.822653
C	-4.076352	-1.322285	1.926400
H	-3.671863	0.411470	-0.263608
H	-2.247030	-0.249018	1.863895
C	-6.207531	-2.274979	1.712378
C	-2.953840	-0.422408	-0.215597
C	-2.833352	-0.908833	1.219440

```
   45
 IX(2E4E)
scf done: -1051.931500
Pd    -1.988489    -3.130922     1.190562
 H    -1.714131    -7.626817     0.920149
 H    -2.982435    -7.349385     2.131024
 C    -1.963848    -5.593043     1.531446
 H    -1.285737    -7.297033     2.630026
 C    -1.973720    -7.059861     1.815674
 O    -2.116949    -5.147619     0.356840
 O    -1.826517    -4.762871     2.493340
 C     3.100086     1.063628    -1.722953
 C     1.885212     0.530468    -0.968223
 C     0.591257     1.064123    -1.577558
 C     0.572426     2.599089    -1.458803
 C     1.883826     3.221995    -1.840574
 C     3.030948     2.545840    -1.983386
 C     4.306339     3.206418    -2.411847
 C    -0.691594     0.470379    -1.036096
 C    -0.701689    -0.338748     0.035698
 C    -1.937491     0.876508    -1.774026
 H     1.878502     4.297763    -2.019530
 H     3.212428     0.536198    -2.682748
 H     1.895376    -0.565499    -0.980427
 H     1.947167     0.840106     0.083830
 H     0.613125     0.830963    -2.654698
 H     0.308470     2.880272    -0.426943
 H    -0.221947     3.016544    -2.090026
 H     5.083305     3.102094    -1.643026
 H     4.706403     2.735737    -3.319949
 H     4.166301     4.272394    -2.615219
 H     0.234361    -0.610979     0.517431
 H    -2.778681     0.202241    -1.597057
 H    -2.256491     1.886707    -1.488467
 H    -1.749234     0.899066    -2.853279
 H     4.016440     0.838552    -1.160445
 O    -2.798674    -1.221959     4.244180
 O    -4.094072    -0.853293     2.440140
 H    -5.395786    -1.627891     3.871105
 H    -6.061885    -0.468337     2.681221
 H    -5.037285     0.110643     4.029837
 H    -2.161321    -1.932182     0.001589
 C    -2.923547    -1.129673     3.039307
 H    -2.852751    -0.400715     0.446199
 H    -0.837904    -1.236209     2.559960
 C    -5.209893    -0.702187     3.319804
 C    -1.919303    -0.918718     0.673374
 C    -1.812109    -1.333984     2.080443

   45
 TS(IX2E4E-X2E4E)
scf done: -1051.925955
Pd    -2.057056    -2.851868     1.125085
 H    -1.343249    -7.226032     0.432041
 H    -2.434390    -7.227294     1.831305
```

C	-1.699671	-5.309519	1.318225
H	-0.695277	-7.028918	2.092787
C	-1.519037	-6.789925	1.416919
O	-2.102032	-4.782706	0.229809
O	-1.483848	-4.562815	2.321465
C	3.080999	0.894274	-1.615902
C	1.849076	0.480775	-0.815276
C	0.573640	0.976586	-1.491410
C	0.593487	2.515977	-1.545843
C	1.922454	3.056077	-1.988407
C	3.051875	2.338536	-2.043601
C	4.346610	2.912412	-2.534327
C	-0.726069	0.488196	-0.897118
C	-0.767291	-0.191524	0.266744
C	-1.960641	0.832558	-1.681321
H	1.946220	4.104082	-2.288894
H	3.184341	0.258573	-2.508693
H	1.829854	-0.609537	-0.705003
H	1.914112	0.904898	0.195796
H	0.591473	0.623576	-2.535450
H	0.336034	2.917627	-0.553120
H	-0.189393	2.879156	-2.223259
H	5.116090	2.876909	-1.751868
H	4.738838	2.330073	-3.378841
H	4.236226	3.951196	-2.859826
H	0.156358	-0.438140	0.784148
H	-2.776848	0.123957	-1.514904
H	-2.331832	1.831343	-1.419113
H	-1.741441	0.846395	-2.754004
H	3.988169	0.711464	-1.024108
O	-3.047289	-1.324614	4.369434
O	-4.263430	-0.758465	2.561294
H	-5.613939	-1.732578	3.814044
H	-6.250432	-0.447345	2.743162
H	-5.310811	-0.020097	4.205676
H	-2.588046	-1.932879	0.018137
C	-3.119570	-1.084574	3.181464
H	-2.928551	-0.176458	0.614934
H	-0.997060	-1.148075	2.793714
C	-5.425998	-0.741388	3.392737
C	-1.993129	-0.625455	0.940085
C	-1.951041	-1.124633	2.269183

45
X(2E4E)
scf done: -1051.931906

Pd	-2.360121	-2.666534	1.289047
H	-0.255333	-6.044463	-0.718766
H	-0.536403	-6.717114	0.887358
C	-0.888322	-4.624221	0.733022
H	0.931645	-5.743960	0.582717
C	-0.128422	-5.854535	0.351613
O	-2.158856	-4.603120	0.537986
O	-0.316829	-3.619112	1.233789
C	2.930847	0.404507	-1.386315
C	1.654238	0.168347	-0.584127

C	0.437375	0.697374	-1.339196
C	0.582696	2.219461	-1.535114
C	1.962457	2.609427	-1.979620
C	3.030184	1.802155	-1.939363
C	4.378254	2.226392	-2.438349
C	-0.910246	0.367252	-0.743550
C	-1.023392	-0.178740	0.491692
C	-2.087551	0.720119	-1.603017
H	2.078590	3.621613	-2.368418
H	2.997004	-0.315717	-2.216175
H	1.543232	-0.901201	-0.372156
H	1.735367	0.681803	0.383319
H	0.448612	0.248241	-2.345592
H	0.330882	2.733032	-0.593959
H	-0.148028	2.575950	-2.271428
H	5.123682	2.199616	-1.632625
H	4.741578	1.542636	-3.217180
H	4.360072	3.237885	-2.855173
H	-0.122348	-0.407992	1.055058
H	-3.002935	0.198847	-1.316715
H	-2.290489	1.797594	-1.559721
H	-1.875166	0.482516	-2.651114
H	3.807849	0.202295	-0.756696
O	-3.551535	-1.210437	4.503591
O	-4.598094	-0.328614	2.716880
H	-6.116423	-1.284119	3.777441
H	-6.549441	0.176852	2.838395
H	-5.661275	0.329412	4.384997
H	-3.846793	-2.470676	1.180060
C	-3.537809	-0.849947	3.344630
H	-3.199266	-0.148907	0.704431
H	-1.405803	-1.017532	3.066925
C	-5.801278	-0.278077	3.487436
C	-2.271530	-0.480043	1.161628
C	-2.325389	-0.926963	2.489757

45
TS(X2E4E-XI2E4E)
scf done: -1051.918793

Pd	-1.652520	-2.429225	1.767248
H	-1.585225	-6.665182	-0.928324
H	-1.597642	-6.925285	0.836895
C	-1.635999	-4.860585	0.249673
H	-0.120034	-6.375329	0.051167
C	-1.211287	-6.296756	0.027986
O	-2.354030	-4.268488	-0.551885
O	-1.162038	-4.352118	1.352643
C	3.122669	0.817917	-1.181428
C	1.849035	0.559175	-0.381737
C	0.616464	0.707617	-1.270361
C	0.540396	2.155221	-1.795756
C	1.870814	2.657760	-2.276498
C	3.043630	2.060602	-2.029182
C	4.340589	2.577881	-2.573721
C	-0.699756	0.297020	-0.655953
C	-0.812034	0.043050	0.672530

```
 C    -1.853720     0.230806    -1.609448
 H     1.856201     3.567509    -2.877504
 H     3.347763    -0.044490    -1.827362
 H     1.887754    -0.442664     0.061366
 H     1.791313     1.277135     0.447291
 H     0.760524     0.060838    -2.150561
 H     0.153823     2.811779    -1.000610
 H    -0.186863     2.220730    -2.614155
 H     5.030329     2.846065    -1.762618
 H     4.849964     1.809109    -3.170036
 H     4.196087     3.458700    -3.206470
 H     0.071930     0.121223     1.301997
 H    -2.710317    -0.319313    -1.216612
 H    -2.195192     1.240457    -1.870253
 H    -1.542754    -0.246158    -2.545466
 H     3.979314     0.900536    -0.499020
 O    -3.307855    -0.696256     4.755539
 O    -4.420347    -0.522597     2.806278
 H    -5.738055    -1.547715     4.053633
 H    -6.439351    -0.486325     2.794818
 H    -5.708027     0.222589     4.266918
 H    -3.070254    -2.813698     1.984652
 C    -3.307595    -0.609625     3.544945
 H    -2.977614    -0.278216     0.826809
 H    -1.172733    -0.389056     3.338167
 C    -5.647515    -0.588392     3.536868
 C    -2.031473    -0.341403     1.357252
 C    -2.062882    -0.573536     2.737692

   45
 XI(2E4E)
scf done: -1051.947721
Pd    -1.521149    -2.480261     2.129905
 H    -1.651172    -6.312377    -0.988774
 H    -2.730651    -6.914001     0.270707
 C    -1.925700    -4.987129     0.639743
 H    -0.964403    -6.880416     0.561300
 C    -1.801639    -6.364017     0.094048
 O    -2.937208    -4.291796     0.160894
 O    -1.147447    -4.512180     1.472739
 C     3.223052     1.097722    -1.138373
 C     1.969893     0.736385    -0.345226
 C     0.743081     0.703145    -1.253446
 C     0.523262     2.105022    -1.852907
 C     1.796652     2.725167    -2.350427
 C     3.023458     2.276310    -2.055433
 C     4.265549     2.902906    -2.613375
 C    -0.532269     0.181368    -0.630281
 C    -0.638320    -0.056757     0.696190
 C    -1.667607    -0.025899    -1.593355
 H     1.694446     3.593321    -3.002662
 H     3.558320     0.234023    -1.733159
 H     2.111759    -0.231997     0.148364
 H     1.816433     1.480597     0.447935
 H     0.978056     0.034766    -2.098659
 H     0.059562     2.754856    -1.093848
```

H	-0.199388	2.053367	-2.676884
H	4.909006	3.287545	-1.811048
H	4.865568	2.164468	-3.162005
H	4.036771	3.728259	-3.294383
H	0.230165	0.111293	1.330164
H	-2.427908	-0.712050	-1.212388
H	-2.168747	0.921273	-1.831617
H	-1.295906	-0.430842	-2.541815
H	4.051255	1.313135	-0.449446
O	-3.223179	-0.559002	4.784080
O	-4.268202	-0.590216	2.791840
H	-5.574429	-1.572354	4.085935
H	-6.284949	-0.638268	2.735755
H	-5.619247	0.205836	4.166104
H	-2.902100	-3.406446	0.611663
C	-3.166425	-0.570000	3.567640
H	-2.772782	-0.507322	0.845289
H	-1.053071	-0.223181	3.400176
C	-5.505229	-0.652236	3.498233
C	-1.834332	-0.515401	1.395075
C	-1.904342	-0.554309	2.805137

45
TS(VIII4E-IX2Z4E)
scf done: -1051.910849

Pd	-1.227309	-1.793522	2.952321
H	-1.735358	-6.024719	4.223622
H	-1.255495	-5.236398	5.733324
C	-0.975580	-4.035327	4.012194
H	0.001070	-5.831581	4.627971
C	-0.987453	-5.372390	4.679600
O	0.084005	-3.374411	3.855607
O	-2.092916	-3.537996	3.613957
C	1.689159	-0.250218	-2.616026
C	0.791834	-0.139376	-1.386043
C	0.093169	1.217609	-1.340783
C	1.157592	2.325026	-1.240778
C	2.307274	2.111925	-2.182459
C	2.554634	0.964612	-2.827647
C	3.690677	0.812248	-3.793512
C	-0.968211	1.386188	-0.270610
C	-1.154782	0.477961	0.699577
C	-1.763716	2.659290	-0.378753
H	2.968884	2.963241	-2.347403
H	1.079438	-0.417188	-3.517459
H	0.056954	-0.952489	-1.391282
H	1.402940	-0.265843	-0.481645
H	-0.412669	1.355204	-2.311096
H	1.526815	2.380030	-0.204021
H	0.704080	3.303728	-1.442247
H	4.392025	0.036159	-3.459459
H	3.327715	0.494955	-4.780373
H	4.247531	1.745626	-3.920248
H	-0.518939	-0.407711	0.696501
H	-2.707943	2.620273	0.170664
H	-1.200863	3.518509	0.007836

H	-1.995469	2.881153	-1.427349
H	2.329752	-1.138868	-2.532013
O	-4.681834	-1.993906	1.616589
O	-2.983698	-1.723697	0.173372
H	-3.887010	-3.518994	-0.379413
H	-3.099781	-2.552490	-1.663844
H	-4.680842	-2.047810	-0.994085
H	-2.904748	1.313797	1.662196
C	-3.588843	-1.529737	1.356532
H	-1.579671	0.966537	2.732385
H	-3.400502	-0.543021	3.196960
C	-3.716773	-2.510240	-0.765812
C	-2.117117	0.570519	1.851531
C	-2.776538	-0.723858	2.315522

45
IX(2Z4E)
scf done: -1051.926464

Pd	-1.878335	-1.735753	3.104481
H	-1.887539	-5.580869	5.377836
H	-2.062371	-4.373011	6.657874
C	-1.568612	-3.557871	4.767000
H	-0.436114	-4.802754	6.086966
C	-1.472032	-4.653558	5.778397
O	-0.664613	-2.679168	4.658321
O	-2.596830	-3.483579	4.011488
C	0.956163	0.438327	-3.428670
C	0.244230	0.172914	-2.104969
C	0.170537	1.444952	-1.263897
C	1.599614	1.913362	-0.933089
C	2.512380	1.861745	-2.123878
C	2.234559	1.218476	-3.265177
C	3.159940	1.234858	-4.444122
C	-0.673648	1.363605	-0.008180
C	-1.170635	0.190768	0.421341
C	-0.873726	2.677879	0.693243
H	3.457119	2.399598	-2.036934
H	0.289991	0.981788	-4.116368
H	-0.759872	-0.222127	-2.297127
H	0.791509	-0.600505	-1.549046
H	-0.280991	2.229679	-1.892760
H	2.006780	1.291406	-0.119976
H	1.580052	2.937076	-0.539181
H	3.491269	0.219932	-4.700655
H	2.653134	1.627941	-5.335690
H	4.046749	1.848512	-4.259108
H	-0.960304	-0.714483	-0.135017
H	-1.713629	2.673926	1.391245
H	0.021811	2.958499	1.261546
H	-1.047607	3.477553	-0.035641
H	1.176307	-0.513509	-3.931018
O	-4.606280	-2.356377	0.484558
O	-2.417888	-2.456571	-0.014022
H	-3.166310	-4.333530	-0.524288
H	-1.781529	-3.704580	-1.468384
H	-3.427913	-3.054583	-1.737977

```
 H    -2.313531     0.946180     2.102533
 C    -3.467972    -1.974894     0.669720
 H    -1.168178    -0.325842     2.486303
 H    -4.022423    -0.612294     2.194380
 C    -2.729786    -3.449001    -0.995333
 C    -2.011792     0.005432     1.639752
 C    -3.115697    -0.977748     1.712424

   45
 TS(IX2Z4E-X2Z4E)
scf done: -1051.920588
Pd    -1.937869    -1.370541     2.912801
 H    -1.097343    -5.295961     4.960393
 H    -1.351705    -4.171580     6.306659
 C    -1.242768    -3.233928     4.412581
 H     0.247573    -4.265963     5.549933
 C    -0.825643    -4.315558     5.356069
 O    -0.610444    -2.127236     4.389194
 O    -2.244570    -3.388189     3.649458
 C     0.934640     0.570018    -3.411723
 C     0.056140     0.286338    -2.196250
 C     0.133979     1.436712    -1.196306
 C     1.583106     1.568279    -0.690183
 C     2.585427     1.492109    -1.805344
 C     2.310047     1.063120    -3.043776
 C     3.335803     1.047966    -4.136507
 C    -0.827306     1.373889    -0.032967
 C    -1.544499     0.260454     0.228696
 C    -0.865738     2.604749     0.827357
 H     3.598499     1.821786    -1.572564
 H     0.450815     1.311837    -4.065625
 H    -0.979087     0.120879    -2.515617
 H     0.395027    -0.640212    -1.713382
 H    -0.095672     2.367324    -1.740442
 H     1.782338     0.776645     0.049492
 H     1.710816     2.515299    -0.151254
 H     3.501013     0.028386    -4.509009
 H     3.000096     1.640274    -4.998292
 H     4.296444     1.450248    -3.801056
 H    -1.429536    -0.612491    -0.401956
 H    -1.845620     2.783070     1.277523
 H    -0.142096     2.524250     1.649726
 H    -0.597131     3.493694     0.248481
 H     1.028934    -0.337415    -4.023531
 O    -4.769871    -2.639180     0.622865
 O    -2.639004    -2.482187    -0.072761
 H    -3.138858    -4.490773    -0.312978
 H    -1.930541    -3.793650    -1.435035
 H    -3.669208    -3.411856    -1.629970
 H    -2.796768     1.073575     1.799750
 C    -3.690802    -2.084515     0.655948
 H    -1.452457     0.065718     2.720511
 H    -4.316471    -0.665014     2.116393
 C    -2.870781    -3.617411    -0.912699
 C    -2.494076     0.137176     1.335213
 C    -3.428844    -0.918331     1.539088
```

```
   45
 X(2Z4E)
scf done: -1051.927499
Pd    -2.375714    -1.047714     2.841810
 H     1.053397    -4.069538     2.992759
 H     0.451075    -3.985112     4.676506
 C    -0.428396    -2.564024     3.351018
 H     1.558640    -2.748583     4.078543
 C     0.728619    -3.403439     3.793026
 O    -1.025605    -1.835587     4.226045
 O    -0.816115    -2.545466     2.153686
 C     1.072152     0.178458    -3.018918
 C     0.018741     0.046239    -1.922573
 C     0.090679     1.230501    -0.961917
 C     1.469351     1.239314    -0.271874
 C     2.592088     0.998460    -1.238755
 C     2.434406     0.540185    -2.487081
 C     3.585846     0.361208    -3.429629
 C    -1.013764     1.325046     0.063364
 C    -1.883980     0.300842     0.251994
 C    -1.028911     2.598776     0.854221
 H     3.596521     1.229689    -0.882676
 H     0.761487     0.935155    -3.755670
 H    -0.977822    -0.027013    -2.372495
 H     0.189929    -0.885576    -1.367024
 H     0.035169     2.152178    -1.563654
 H     1.483748     0.474569     0.520907
 H     1.630580     2.197323     0.237255
 H     3.684721    -0.688103    -3.737441
 H     3.432516     0.939433    -4.350659
 H     4.532998     0.678180    -2.982851
 H    -1.780019    -0.596576    -0.344470
 H    -1.990350     2.807963     1.326666
 H    -0.274529     2.562153     1.650655
 H    -0.771421     3.449571     0.214481
 H     1.143041    -0.762077    -3.581829
 O    -5.191945    -2.609738     0.939590
 O    -3.303728    -2.237218    -0.219522
 H    -3.524246    -4.309107    -0.216534
 H    -2.706362    -3.560936    -1.622287
 H    -4.484329    -3.442404    -1.444394
 H    -3.149909     1.256520     1.722754
 C    -4.213123    -1.928420     0.709579
 H    -3.208971    -0.175790     3.739275
 H    -4.812232    -0.364661     2.023359
 C    -3.529334    -3.467626    -0.913901
 C    -2.953866     0.308904     1.226693
 C    -3.938545    -0.671659     1.455372

   45
 X'(2Z4E)
scf done: -1051.915597
Pd    -2.250008    -1.621041     2.329130
 H     1.397074    -2.449901     5.664605
 H     1.299822    -3.668657     4.362503
```

C	-0.191486	-2.121467	4.244681
H	0.119878	-3.695838	5.670284
C	0.719987	-3.032854	5.038602
O	-0.160799	-0.900331	4.381543
O	-0.979632	-2.764647	3.430954
C	0.932468	0.577670	-3.244439
C	-0.097787	0.286805	-2.156618
C	0.120180	1.198289	-0.951897
C	1.512976	0.921435	-0.350393
C	2.577293	0.813406	-1.403128
C	2.338878	0.676878	-2.713724
C	3.439217	0.618648	-3.729602
C	-0.933166	1.145779	0.126879
C	-1.905503	0.196675	0.115842
C	-0.785662	2.179591	1.202540
H	3.609344	0.861401	-1.054492
H	0.678985	1.512354	-3.767780
H	-1.108607	0.413571	-2.560562
H	-0.005285	-0.761667	-1.842414
H	0.136998	2.238202	-1.316546
H	1.474686	-0.005637	0.243422
H	1.782532	1.715028	0.356913
H	3.421706	-0.333178	-4.276664
H	3.320413	1.409203	-4.482629
H	4.425824	0.732141	-3.270424
H	-1.923989	-0.528495	-0.689363
H	-1.719511	2.398964	1.724002
H	-0.062551	1.845375	1.957224
H	-0.400340	3.114739	0.783688
H	0.891540	-0.205413	-4.013531
O	-5.423995	-2.419669	0.162455
O	-3.299463	-2.335782	-0.569293
H	-3.954069	-4.262110	-1.023017
H	-2.707870	-3.535069	-2.083922
H	-4.414009	-3.003211	-2.199883
H	-3.029343	0.881573	1.841986
C	-4.307487	-1.951121	0.227798
H	-3.076688	-1.314531	3.525100
H	-4.760529	-0.703685	1.896817
C	-3.626027	-3.349682	-1.526935
C	-2.920325	0.056477	1.141387
C	-3.934685	-0.914410	1.223810

45
TS(X'2Z4E-XI2Z4E)
scf done: -1051.915590

Pd	-2.232191	-1.621185	2.324992
H	1.293062	-2.488124	5.799766
H	1.355638	-3.574369	4.383555
C	-0.234804	-2.126452	4.322253
H	0.111345	-3.811678	5.607634
C	0.694996	-3.049846	5.080794
O	-0.289162	-0.923794	4.566244
O	-0.938601	-2.742600	3.414317
C	0.921663	0.588583	-3.264411
C	-0.103562	0.295105	-2.172549

C	0.122968	1.200394	-0.964740
C	1.518246	0.917342	-0.371944
C	2.576786	0.811528	-1.430720
C	2.331102	0.681664	-2.740656
C	3.425940	0.625175	-3.762559
C	-0.924836	1.145259	0.119326
C	-1.898552	0.197650	0.109945
C	-0.769879	2.174267	1.198637
H	3.610778	0.855252	-1.087319
H	0.667862	1.526426	-3.781913
H	-1.116225	0.426150	-2.570460
H	-0.011894	-0.755070	-1.863834
H	0.140413	2.241950	-1.324640
H	1.480757	-0.012422	0.217739
H	1.793471	1.706982	0.337618
H	3.403186	-0.324222	-4.313629
H	3.305149	1.419314	-4.511479
H	4.415224	0.734243	-3.308095
H	-1.922038	-0.524808	-0.697587
H	-1.700991	2.394023	1.724873
H	-0.044494	1.835261	1.949074
H	-0.383814	3.110144	0.782107
H	0.874659	-0.190660	-4.037045
O	-5.414569	-2.417525	0.162767
O	-3.289244	-2.338837	-0.567379
H	-3.949227	-4.262653	-1.024002
H	-2.700001	-3.538045	-2.082988
H	-4.404428	-3.000934	-2.199680
H	-3.015264	0.879769	1.841747
C	-4.296942	-1.951849	0.229256
H	-3.046785	-1.307331	3.526685
H	-4.745405	-0.707279	1.901808
C	-3.618042	-3.350576	-1.526497
C	-2.908260	0.055061	1.140374
C	-3.921548	-0.916978	1.226023

45
XI(2Z4E)
scf done: -1051.943436

Pd	-2.218163	-1.930525	2.264942
H	1.101817	-2.111657	5.940689
H	1.069003	-3.706790	5.127565
C	-0.533615	-2.405504	4.622906
H	-0.058199	-3.333574	6.464529
C	0.464255	-2.929197	5.591865
O	-1.278299	-1.418376	5.076645
O	-0.668903	-2.850018	3.478081
C	0.893871	0.628710	-3.366708
C	-0.063022	0.266801	-2.233907
C	0.166384	1.166858	-1.022265
C	1.600043	0.950035	-0.501238
C	2.616172	0.921854	-1.605954
C	2.318433	0.796720	-2.905734
C	3.366282	0.819932	-3.977271
C	-0.836685	1.039387	0.102399
C	-1.750387	0.040822	0.128091

C	-0.692427	2.067120	1.189194
H	3.661318	1.021251	-1.310384
H	0.565202	1.556310	-3.860385
H	-1.098717	0.345784	-2.583524
H	0.098192	-0.780794	-1.945400
H	0.116219	2.211080	-1.373312
H	1.639082	0.008391	0.069754
H	1.864956	1.739064	0.213620
H	3.371323	-0.119469	-4.545785
H	3.167059	1.618779	-4.704219
H	4.368113	0.976952	-3.566163
H	-1.771364	-0.669119	-0.690446
H	-1.611679	2.221048	1.759217
H	0.088579	1.778161	1.905216
H	-0.392638	3.033723	0.769100
H	0.856628	-0.143131	-4.147636
O	-5.312333	-2.641144	0.228872
O	-3.388083	-2.224653	-0.850667
H	-3.930450	-4.156271	-1.418684
H	-3.013113	-3.167773	-2.594843
H	-4.749268	-2.845312	-2.303764
H	-2.816171	0.624266	1.919152
C	-4.250952	-2.047781	0.168012
H	-1.899448	-1.177498	4.336260
H	-4.617465	-0.886253	1.902721
C	-3.806595	-3.157772	-1.846828
C	-2.724345	-0.180506	1.190653
C	-3.806625	-1.097125	1.207541

41
II(4Z)
scf done: -974.625276

Pd	1.513912	5.802878	0.391576
H	-1.442949	7.642238	-2.301464
H	3.679083	3.370047	3.251755
H	-1.089285	6.089346	-3.076949
H	4.655756	4.848377	3.327983
C	0.188136	6.566884	-1.472550
C	2.834875	5.013963	2.247256
H	-0.019659	7.476927	-3.382998
H	3.167853	4.692919	4.321804
C	-0.637287	6.982660	-2.631468
C	3.636150	4.459525	3.363952
O	-0.327007	6.321660	-0.330016
O	3.354993	5.290515	1.114022
O	1.451941	6.408450	-1.560103
O	1.578161	5.214126	2.347961
C	4.170365	-0.039724	-1.174400
C	3.046058	0.794229	-0.566842
C	2.219593	1.475691	-1.654813
C	3.126324	2.428991	-2.453551
C	4.450782	1.812686	-2.799490
C	4.932576	0.690794	-2.249077
C	6.242253	0.089702	-2.661732
C	0.971532	2.179943	-1.167317
C	0.683779	2.352498	0.129341

C	0.043229	2.683973	-2.237217
H	5.041479	2.323185	-3.561112
H	3.766002	-0.973886	-1.594130
H	2.408501	0.159342	0.058762
H	3.476690	1.561218	0.091820
H	1.887761	0.693484	-2.357631
H	3.283697	3.352992	-1.874341
H	2.625242	2.743204	-3.377595
H	6.940445	0.044033	-1.815450
H	6.108522	-0.944440	-3.006770
H	6.717353	0.657651	-3.467360
H	-0.231882	2.853631	0.436079
H	1.331803	1.996847	0.925303
H	-0.866384	3.113843	-1.807698
H	0.522192	3.453154	-2.855219
H	-0.246196	1.873731	-2.918681
H	4.869313	-0.355485	-0.387741

41
TS(II4Z-III4Z)
scf done: -974.613272

Pd	1.448734	4.988584	0.058251
H	-1.346854	7.510635	-2.240189
H	3.979807	2.972004	3.087400
H	-0.836800	6.315004	-3.441803
H	4.860862	4.486306	2.829747
C	0.318711	6.313986	-1.673654
C	2.867608	4.450604	2.061749
H	0.140318	7.782560	-3.205349
H	3.571875	4.392890	4.072910
C	-0.476568	7.037195	-2.700840
C	3.885225	4.063750	3.080664
O	-0.272719	5.675101	-0.728008
O	3.234281	4.544235	0.825631
O	1.582290	6.273622	-1.694405
O	1.664887	4.659582	2.343167
C	4.157525	0.262152	-0.955865
C	3.003783	1.094611	-0.403680
C	2.155512	1.668751	-1.536375
C	3.025058	2.593261	-2.407826
C	4.369150	1.999657	-2.713005
C	4.892545	0.940479	-2.082222
C	6.222354	0.357965	-2.454866
C	0.875899	2.349256	-1.105650
C	0.568944	2.597885	0.190335
C	-0.093620	2.678207	-2.203429
H	4.938480	2.473276	-3.513578
H	3.787492	-0.713447	-1.307505
H	2.388984	0.476562	0.260519
H	3.401771	1.920458	0.199936
H	1.847652	0.828829	-2.180883
H	3.148849	3.559810	-1.894875
H	2.510327	2.820405	-3.349545
H	6.922781	0.400088	-1.610348
H	6.125163	-0.702951	-2.721979
H	6.675382	0.881545	-3.302127

```
 H    -0.413254     2.979799     0.462283
 H     1.204443     2.265484     1.004885
 H    -1.000284     3.146072    -1.813835
 H     0.348276     3.350013    -2.948223
 H    -0.374035     1.762339    -2.739351
 H     4.867362     0.028738    -0.150661

   41
 III(4Z)
scf done:  -974.639843
Pd     1.157913     4.459310    -0.487222
 H    -1.227727     7.306410    -2.907081
 H     4.785804     4.867454     2.097678
 H     0.192341     8.329542    -2.509424
 H     3.821170     4.804427     3.600963
 C     0.159097     6.436980    -1.538557
 C     2.683498     4.527506     1.803420
 H    -1.108003     8.109175    -1.326957
 H     4.205828     3.314114     2.699897
 C    -0.536538     7.615174    -2.120944
 C     3.952174     4.375074     2.606756
 O    -0.370742     5.270874    -1.596626
 O     2.804692     4.115134     0.567687
 O     1.273950     6.545634    -0.942429
 O     1.647966     4.985803     2.278327
 C     4.160624     0.179507    -0.850721
 C     3.006954     1.051978    -0.362395
 C     2.247216     1.641245    -1.549457
 C     3.180335     2.530445    -2.388169
 C     4.515481     1.885171    -2.619123
 C     4.971787     0.820636    -1.946546
 C     6.300042     0.192626    -2.243213
 C     0.923858     2.310927    -1.228276
 C     0.467153     2.540851     0.069055
 C    -0.041900     2.370456    -2.377309
 H     5.139400     2.328292    -3.395840
 H     3.779711    -0.788641    -1.211441
 H     2.333557     0.452282     0.260785
 H     3.389227     1.867775     0.259964
 H     1.960812     0.798873    -2.200252
 H     3.316879     3.498251    -1.883563
 H     2.713864     2.758890    -3.354729
 H     6.958265     0.227798    -1.365143
 H     6.183835    -0.869104    -2.499054
 H     6.810321     0.688904    -3.074167
 H    -0.596196     2.700970     0.243801
 H     1.054085     2.229787     0.927738
 H    -0.944592     2.931611    -2.133410
 H     0.413658     2.799438    -3.274608
 H    -0.329466     1.339497    -2.623283
 H     4.821868    -0.065777    -0.008730

   41
 TS(III4Z-IV4Z)
scf done:  -974.607845
Pd     1.489523     4.804842    -0.122825
```

H	1.815912	7.236954	-3.767015
H	3.459562	4.867982	3.927643
H	1.516076	8.538128	-2.592872
H	2.729406	3.348598	4.542522
C	1.311868	6.556084	-1.829775
C	2.382057	3.722093	2.462132
H	0.153059	7.599111	-3.283218
H	4.170055	3.295968	3.513622
C	1.185474	7.554082	-2.928800
C	3.229314	3.826661	3.698588
O	0.669751	5.448829	-1.891055
O	2.502470	4.662810	1.611344
O	2.065678	6.753545	-0.829888
O	1.638498	2.719773	2.316607
C	4.460528	1.131559	-1.052865
C	3.210275	1.800097	-0.488813
C	2.013901	1.542347	-1.402588
C	2.283349	2.188562	-2.779134
C	3.664577	1.880139	-3.279941
C	4.655134	1.388195	-2.524404
C	6.004420	1.055497	-3.085555
C	0.668794	1.991067	-0.895709
C	0.459718	3.026845	-0.019791
C	-0.484341	1.193535	-1.415360
H	3.848098	2.069095	-4.338067
H	4.423086	0.044813	-0.880319
H	3.010577	1.432498	0.522414
H	3.378762	2.882493	-0.411167
H	1.946317	0.457399	-1.572454
H	2.140375	3.276724	-2.700990
H	1.543497	1.839621	-3.510507
H	6.791205	1.646614	-2.598875
H	6.254525	0.001329	-2.905704
H	6.055011	1.238068	-4.163174
H	-0.592293	3.239241	0.196171
H	1.082885	2.843474	1.055756
H	-1.448849	1.626012	-1.141449
H	-0.426921	1.103775	-2.507581
H	-0.429329	0.169148	-1.023770
H	5.346196	1.481693	-0.505734

41
IV(4Z)
scf done: -974.625138

Pd	1.481608	4.804792	-0.135072
H	2.547751	7.047894	-3.794995
H	2.837478	4.830727	4.224590
H	2.278622	8.443373	-2.721141
H	1.803237	3.462634	4.747503
C	1.764152	6.542139	-1.889719
C	2.010265	3.668427	2.644379
H	0.900433	7.620968	-3.516512
H	3.424477	3.146831	4.124037
C	1.877228	7.482408	-3.045661
C	2.550737	3.799369	4.024268
O	1.107792	5.447353	-2.045578

O	2.052963	4.615263	1.843293
O	2.306341	6.778913	-0.777380
O	1.510234	2.499041	2.355171
C	4.562955	1.414146	-1.353669
C	3.327931	2.000856	-0.675098
C	2.053648	1.575265	-1.407336
C	2.091567	2.120856	-2.844465
C	3.418818	1.887640	-3.506478
C	4.541453	1.557110	-2.853900
C	5.836648	1.296538	-3.562522
C	0.744911	1.944307	-0.731881
C	0.449534	3.143433	-0.189110
C	-0.294361	0.851966	-0.757614
H	3.451370	1.993327	-4.591554
H	4.665305	0.347609	-1.099231
H	3.295132	1.682553	0.373479
H	3.393052	3.098524	-0.675951
H	2.079350	0.477949	-1.478499
H	1.860460	3.195963	-2.825630
H	1.296057	1.653749	-3.440791
H	6.616089	1.996380	-3.233438
H	6.211972	0.289148	-3.337184
H	5.733036	1.385230	-4.648235
H	-0.561400	3.286772	0.213344
H	1.187010	2.503441	1.406137
H	-1.245900	1.174857	-0.325848
H	-0.477271	0.527222	-1.790314
H	0.056120	-0.031866	-0.209260
H	5.468895	1.893695	-0.958234

41
TS(IV4Z-V4Z)
scf done: -974.604742

Pd	1.910612	4.566574	-0.909574
H	0.479467	8.453476	-2.599558
H	3.228799	4.687542	3.987811
H	2.203112	8.870835	-2.335113
H	1.486116	4.462444	4.316324
C	1.619879	6.964014	-1.565523
C	2.120964	3.803926	2.396357
H	1.048490	8.921568	-0.989256
H	2.563104	3.065683	4.344950
C	1.321731	8.389447	-1.907470
C	2.375335	4.024395	3.852355
O	0.651804	6.124544	-1.512722
O	2.774934	4.321784	1.500798
O	2.791033	6.578926	-1.300088
O	1.104779	2.983239	2.166563
C	4.691312	1.705110	-1.429134
C	3.318009	2.254528	-1.052639
C	2.177763	1.323866	-1.455235
C	2.221817	1.081933	-2.974965
C	3.610763	0.828820	-3.485209
C	4.733208	1.094447	-2.804601
C	6.096568	0.792862	-3.349282
C	0.822053	1.846300	-1.043657

```
 C      0.593347      3.152107    -0.805937
 C     -0.292269      0.836784    -0.970607
 H      3.690675      0.398824    -4.484028
 H      4.996526      0.949135    -0.689608
 H      3.292850      2.462911     0.020085
 H      3.193458      3.216352    -1.625670
 H      2.346997      0.355217    -0.961328
 H      1.790728      1.955040    -3.489195
 H      1.575555      0.235128    -3.237015
 H      6.697360      1.707475    -3.438080
 H      6.649560      0.124204    -2.676063
 H      6.045952      0.318620    -4.333894
 H     -0.418817      3.513945    -0.594648
 H      0.987998      2.893843     1.191457
 H     -1.243976      1.301900    -0.699553
 H     -0.422845      0.328767    -1.934259
 H     -0.062781      0.058791    -0.231586
 H      5.443728      2.501495    -1.357302

   41
 V(4Z)
scf done:  -974.611429
Pd      1.909731      4.581143    -1.526670
 H      0.545331      8.757725    -2.172209
 H      3.088549      3.597978     4.416745
 H      2.308568      9.067056    -2.101054
 H      1.709476      4.700682     4.149695
 C      1.727973      7.061857    -1.641597
 C      2.126091      3.386150     2.526146
 H      1.357358      8.875782    -0.607924
 H      1.403946      2.999737     4.499994
 C      1.474734      8.534317    -1.642063
 C      2.088610      3.685786     3.992995
 O      0.753081      6.268973    -1.367385
 O      3.135930      3.156741     1.889831
 O      2.864793      6.575509    -1.880582
 O      0.907018      3.402403     1.979877
 C      4.635952      2.206304    -1.788629
 C      3.196379      2.603826    -1.474848
 C      2.224486      1.429763    -1.385882
 C      2.262200      0.619410    -2.692082
 C      3.654417      0.426866    -3.220771
 C      4.730192      1.122267    -2.829353
 C      6.100892      0.862338    -3.376967
 C      0.835471      1.925471    -1.077729
 C      0.563825      3.243813    -1.122035
 C     -0.217551      0.903424    -0.752965
 H      3.778051     -0.340146    -3.985633
 H      5.128071      1.865856    -0.865755
 H      3.194223      3.147877    -0.512056
 H      2.865577      3.212234    -2.379990
 H      2.563480      0.770549    -0.572380
 H      1.646291      1.126706    -3.451262
 H      1.791604     -0.358887    -2.535885
 H      6.502196      1.754206    -3.875726
 H      6.804879      0.615253    -2.571212
```

```
 H     6.102061     0.037183    -4.095183
 H    -0.441545     3.634814    -0.940810
 H     0.990254     3.217322     1.019927
 H    -1.178038     1.374436    -0.526792
 H    -0.366333     0.211559    -1.591034
 H     0.083266     0.296646     0.110345
 H     5.203710     3.086372    -2.116693

   45
 VI(4Z)
scf done: -1051.888706
Pd     1.124629    -1.492478     0.895232
 H    -0.047319    -4.775409     3.810874
 H     0.913652    -5.618465     2.553689
 C     0.509593    -3.557254     2.145375
 H    -0.786744    -5.226812     2.259969
 C     0.134070    -4.876257     2.739574
 O     1.136527    -3.542548     1.023225
 O     0.207091    -2.466388     2.696894
 C     0.582557     2.072953     1.379748
 C     0.990287     0.844092     0.573050
 C     1.734483     1.173764    -0.717302
 C     0.816461     2.021119    -1.609351
 C     0.100704     3.094363    -0.839531
 C    -0.006109     3.143595     0.495596
 C    -0.709405     4.259912     1.207075
 C     2.226690    -0.084935    -1.383485
 C     2.034776    -1.280425    -0.802131
 C     2.943126     0.055204    -2.698216
 H    -0.359374     3.888231    -1.428365
 H     1.447072     2.483279     1.921188
 H     1.694929     0.263628     1.248035
 H     0.061569     0.301644     0.305420
 H     2.607958     1.794707    -0.461251
 H     0.082068     1.366651    -2.104740
 H     1.398695     2.479046    -2.418341
 H    -1.562419     3.882301     1.786138
 H    -0.040417     4.751565     1.925693
 H    -1.076896     5.018179     0.509420
 H     2.384772    -2.212667    -1.253380
 H     3.324680    -0.907445    -3.049979
 H     2.280990     0.460451    -3.473425
 H     3.789273     0.748892    -2.609792
 H    -0.147365     1.789911     2.148934
 O    -2.490176    -2.678577    -0.170192
 O    -2.228281    -0.730147    -1.267244
 H    -0.857253    -1.973955    -2.221333
 H    -1.688752    -0.739444    -3.213830
 H    -2.561312    -2.213209    -2.694077
 H    -2.814745     1.267414     0.086275
 C    -2.538683    -1.464418    -0.189965
 H    -3.377832     1.217220     1.855153
 H    -3.204855    -1.237303     1.852674
 C    -1.809167    -1.471161    -2.414667
 C    -3.052772     0.680007     0.968312
 C    -2.960079    -0.651230     0.971149
```

```
   45
 TS(VI4Z-VII4Z)
scf done: -1051.881954
Pd     0.285042    -1.426001     1.016614
 H    -0.546431    -4.457740     4.310548
 H     1.219194    -4.481348     4.145195
 C     0.229397    -3.326348     2.667728
 H     0.234055    -5.439368     3.029155
 C     0.273685    -4.504137     3.591602
 O     0.767149    -3.428253     1.510879
 O    -0.299342    -2.235616     3.022723
 C     1.105900     2.379997     1.549309
 C     1.199236     1.026268     0.851870
 C     1.454672     1.137988    -0.648246
 C     0.345625     1.977529    -1.308091
 C    -0.044826     3.180935    -0.500061
 C     0.290931     3.386185     0.780448
 C    -0.100561     4.625101     1.527784
 C     1.575086    -0.215824    -1.304166
 C     1.101793    -1.332360    -0.732405
 C     2.210708    -0.252564    -2.669749
 H    -0.645626     3.934100    -1.011080
 H     2.120127     2.778151     1.705361
 H     1.977208     0.421107     1.330627
 H     0.184881     0.549393     0.987697
 H     2.403236     1.680994    -0.780621
 H    -0.535641     1.340306    -1.477375
 H     0.670450     2.299791    -2.305576
 H    -0.720197     4.380991     2.400661
 H     0.785045     5.147144     1.914148
 H    -0.657861     5.322885     0.895741
 H     1.180690    -2.311955    -1.213030
 H     2.239727    -1.268127    -3.074802
 H     1.663692     0.380478    -3.379995
 H     3.238511     0.131204    -2.631553
 H     0.684693     2.254806     2.555425
 O    -2.081854    -2.965105    -1.862425
 O    -2.156630    -0.723540    -2.048753
 H    -0.983841    -1.329842    -3.659337
 H    -1.977334     0.145068    -3.862922
 H    -2.744375    -1.471416    -3.903009
 H    -2.255610     0.466917     0.127889
 C    -2.166578    -1.866091    -1.352788
 H    -2.489029    -0.364912     1.774056
 H    -2.436937    -2.573067     0.676712
 C    -1.952422    -0.865603    -3.455168
 C    -2.337205    -0.451369     0.701714
 C    -2.308235    -1.656650     0.107365

   45
 VII(4Z)
scf done: -1051.912607
Pd    -0.599943    -1.756531     0.570979
 H     1.200241    -4.559540     3.546717
 H     0.118089    -3.663421     4.642100
```

C	0.259855	-2.880928	2.652672
H	1.724353	-3.005534	4.199146
C	0.854143	-3.563547	3.843258
O	1.032945	-2.508805	1.700582
O	-0.985182	-2.681602	2.562953
C	1.360580	2.200206	1.846943
C	0.961967	0.947606	1.069726
C	1.193043	1.143134	-0.427094
C	0.305305	2.296344	-0.928173
C	0.344432	3.488026	-0.015374
C	0.831883	3.470280	1.232369
C	0.891471	4.698370	2.089918
C	1.047656	-0.085763	-1.303373
C	0.339032	-1.193891	-1.049949
C	1.805043	0.001337	-2.609531
H	-0.045342	4.424457	-0.416330
H	2.457287	2.267208	1.919795
H	1.526810	0.083695	1.436588
H	-0.102583	0.737853	1.252827
H	2.235280	1.480535	-0.544204
H	-0.731271	1.941070	-1.034887
H	0.615206	2.602115	-1.935655
H	0.298527	4.570944	3.005236
H	1.920876	4.900037	2.415415
H	0.522415	5.583425	1.562807
H	0.317324	-1.998700	-1.791374
H	1.644326	-0.880432	-3.236293
H	1.502214	0.885050	-3.185244
H	2.882551	0.099221	-2.424382
H	1.005711	2.126106	2.884079
O	-2.367284	-3.665271	-2.036984
O	-2.385738	-1.508715	-2.680201
H	-1.314960	-2.477501	-4.184816
H	-2.267582	-1.031721	-4.639722
H	-3.090062	-2.589180	-4.317201
H	-2.096559	0.110347	-0.742954
C	-2.402781	-2.482298	-1.770174
H	-2.725421	-0.292036	0.952728
H	-2.990244	-2.676414	0.298189
C	-2.255814	-1.940635	-4.038731
C	-2.335510	-0.643930	-0.000103
C	-2.502085	-1.970872	-0.372176

45
TS(VII4Z-VIII'4Z)
scf done: -1051.900815

Pd	-0.328612	-1.713979	-0.008057
H	1.276029	-5.297566	2.089173
H	0.451210	-4.491268	3.435825
C	0.623961	-3.333220	1.642964
H	2.127733	-4.034501	2.991933
C	1.151577	-4.342410	2.611888
O	1.412778	-2.666129	0.907668
O	-0.633046	-3.156681	1.524652
C	0.815496	2.319752	2.115013
C	0.585744	1.218518	1.082646

C	1.286753	1.565620	-0.230581
C	0.674665	2.858835	-0.803865
C	0.497974	3.918998	0.244585
C	0.578360	3.702027	1.563955
C	0.445208	4.805245	2.569894
C	1.370449	0.482753	-1.287230
C	0.592472	-0.606919	-1.428516
C	2.477951	0.693178	-2.290744
H	0.305258	4.930849	-0.113795
H	1.841807	2.261554	2.509483
H	0.950197	0.260458	1.470863
H	-0.492635	1.104959	0.915122
H	2.329581	1.811471	0.026625
H	-0.293677	2.638200	-1.279047
H	1.309922	3.251316	-1.607840
H	-0.405971	4.628167	3.240486
H	1.335585	4.860958	3.210481
H	0.308662	5.780182	2.092239
H	0.756933	-1.258862	-2.288607
H	2.495157	-0.089228	-3.054309
H	2.375573	1.660528	-2.799206
H	3.453734	0.704958	-1.788339
H	0.161287	2.160264	2.982990
O	-3.866525	-2.884820	-0.420670
O	-2.533036	-2.773608	-2.230969
H	-3.050278	-4.786832	-2.084051
H	-2.740706	-4.124457	-3.717930
H	-4.266915	-3.742710	-2.863629
H	-1.442459	-0.552683	-2.447037
C	-2.966075	-2.350871	-1.034297
H	-1.396415	0.725269	-1.133224
H	-2.684775	-0.725925	0.355577
C	-3.196431	-3.929905	-2.747028
C	-1.456782	-0.326984	-1.385039
C	-2.237517	-1.154398	-0.539954

45
VIII'(4Z)
scf done: -1051.952312

Pd	0.098329	-1.636149	-0.141525
H	1.706488	-5.520041	1.316786
H	1.115198	-4.840423	2.839848
C	1.160206	-3.477171	1.190399
H	2.757818	-4.434299	2.246130
C	1.723777	-4.631078	1.957498
O	1.915408	-2.686559	0.551694
O	-0.104338	-3.302660	1.152405
C	0.186169	2.545082	2.093025
C	-0.106366	1.411955	1.112551
C	0.940354	1.411162	-0.004947
C	0.832319	2.724999	-0.801302
C	0.736381	3.920887	0.101300
C	0.463417	3.860346	1.411272
C	0.422467	5.080606	2.280556
C	1.007278	0.238153	-0.976934
C	-0.027388	-0.245038	-1.773657

C	2.420146	-0.103622	-1.377394
H	0.915077	4.893360	-0.358410
H	1.044963	2.283885	2.730544
H	-0.103629	0.449890	1.637691
H	-1.110587	1.550671	0.697156
H	1.917031	1.427206	0.499201
H	-0.041916	2.685067	-1.468255
H	1.702963	2.833784	-1.461092
H	-0.570948	5.212733	2.729053
H	1.128799	4.990337	3.116626
H	0.670358	5.988449	1.722379
H	0.251520	-0.853107	-2.634861
H	2.456609	-0.886410	-2.139244
H	2.910580	0.793494	-1.777485
H	3.002666	-0.431104	-0.510510
H	-0.660979	2.666625	2.781397
O	-3.342352	-2.754258	-0.074071
O	-2.443138	-2.563203	-2.126231
H	-2.871481	-4.591531	-1.912235
H	-2.948306	-3.893845	-3.558221
H	-4.252725	-3.568018	-2.377344
H	-2.049054	-0.146731	-2.519520
C	-2.608542	-2.169665	-0.847806
H	-1.700398	1.068463	-1.276383
H	-2.145788	-0.554721	0.444133
C	-3.178419	-3.725691	-2.505635
C	-1.499733	0.043457	-1.595267
C	-1.801034	-0.973683	-0.502717

45
VIII(4Z)
scf done: -1051.958409

Pd	0.171201	-1.409469	-0.325632
H	0.894045	-5.399544	1.508932
H	0.486477	-4.463208	2.949951
C	0.818338	-3.305282	1.185489
H	2.173607	-4.490947	2.346976
C	1.121032	-4.480440	2.060319
O	1.743158	-2.650679	0.622588
O	-0.399545	-2.971758	0.984991
C	0.883726	4.083147	-0.126397
C	1.507490	2.860332	-0.792727
C	0.867455	1.573622	-0.243434
C	1.171424	1.462737	1.254158
C	0.916997	2.756059	1.972999
C	0.766402	3.942883	1.369728
C	0.471409	5.199597	2.131550
C	1.296709	0.365487	-1.055240
C	0.488631	-0.188875	-2.048325
C	2.764179	0.027242	-1.059934
H	0.844612	2.703467	3.059665
H	-0.113876	4.279172	-0.548328
H	1.383264	2.917700	-1.880365
H	2.585640	2.844996	-0.590227
H	-0.217159	1.692721	-0.343771
H	2.213718	1.150378	1.414436

H	0.554655	0.667213	1.694592
H	1.266679	5.943411	1.990407
H	-0.455449	5.667383	1.773447
H	0.364677	5.011180	3.204047
H	0.955910	-0.851302	-2.777623
H	2.963223	-0.867711	-1.654229
H	3.333879	0.857864	-1.494701
H	3.150415	-0.138329	-0.050602
H	1.478720	4.976743	-0.359717
O	-3.440436	-1.880997	-0.399668
O	-2.343935	-2.345634	-2.307082
H	-3.173534	-4.160230	-1.706647
H	-2.966674	-3.880257	-3.462028
H	-4.283700	-3.064618	-2.566360
H	-1.349916	-0.286115	-3.157506
C	-2.541692	-1.634055	-1.180423
H	-1.176812	1.225732	-2.248671
H	-1.878865	0.123609	-0.204851
C	-3.253181	-3.425893	-2.513000
C	-0.975204	0.150296	-2.230313
C	-1.546799	-0.547828	-0.999878

45
TS(VIII4Z-IX2E4Z)
scf done: -1051.915354

Pd	0.265730	-2.866032	-0.681180
H	0.074951	-6.415144	1.964667
H	1.029392	-5.305159	2.956269
C	0.764323	-4.705612	0.921057
H	1.838779	-6.355150	1.749225
C	0.951562	-5.757978	1.965167
O	1.311443	-4.781165	-0.211213
O	-0.001408	-3.707522	1.183119
C	0.533080	4.333632	-0.375385
C	1.494564	3.285407	-0.927658
C	1.090937	1.880365	-0.461063
C	1.185654	1.819448	1.072946
C	0.562775	3.017216	1.729972
C	0.244931	4.151983	1.092825
C	-0.415560	5.304712	1.787160
C	1.891566	0.781402	-1.121343
C	1.318938	-0.267913	-1.738676
C	3.389604	0.903745	-1.085469
H	0.353465	2.935082	2.797171
H	-0.416558	4.310776	-0.932051
H	1.516681	3.334232	-2.022739
H	2.510957	3.506329	-0.577027
H	0.032775	1.743859	-0.719696
H	2.237534	1.733146	1.384798
H	0.697661	0.905678	1.436953
H	0.211892	6.204839	1.744014
H	-1.363851	5.566449	1.298761
H	-0.624597	5.083075	2.838125
H	1.984073	-1.008209	-2.187946
H	3.870556	0.013113	-1.500221
H	3.731684	1.770091	-1.665014

H	3.762937	1.046564	-0.064307
H	0.941271	5.340301	-0.540494
O	-2.953205	-1.573014	0.263788
O	-2.629463	-1.867969	-1.942891
H	-4.180139	-3.172324	-1.454187
H	-4.169154	-2.472917	-3.101652
H	-4.661937	-1.475240	-1.699503
H	-0.306749	-1.224992	-2.748751
C	-2.216699	-1.541407	-0.702880
H	-0.720173	0.365367	-2.164014
H	-0.560926	-0.638201	0.269140
C	-3.995700	-2.270383	-2.044442
C	-0.158774	-0.543228	-1.896341
C	-0.774925	-1.174953	-0.657916

45
IX(2E4Z)
scf done: -1051.931585

Pd	0.277847	-2.917217	-0.374587
H	-0.820444	-6.739656	-2.328157
H	0.737895	-7.177995	-1.618950
C	0.278168	-5.093115	-1.575214
H	0.672963	-6.391927	-3.228698
C	0.227182	-6.433164	-2.233839
O	0.467746	-4.034793	-2.242435
O	0.101352	-5.002919	-0.312593
C	0.642486	3.654345	-2.052390
C	1.715715	2.817452	-1.362954
C	1.113184	2.026914	-0.195178
C	0.562607	3.010538	0.850529
C	-0.230888	4.121227	0.225190
C	-0.223562	4.415605	-1.081723
C	-1.069897	5.507399	-1.663605
C	2.082635	1.046828	0.426677
C	1.752925	-0.216600	0.745123
C	3.472312	1.541733	0.710024
H	-0.858647	4.709753	0.895201
H	0.002084	3.013086	-2.677564
H	2.185247	2.138734	-2.084670
H	2.504801	3.480444	-0.985980
H	0.256278	1.472941	-0.598463
H	1.388164	3.431448	1.444464
H	-0.066213	2.466846	1.568071
H	-0.448939	6.278869	-2.137831
H	-1.729220	5.116179	-2.450048
H	-1.694439	5.988654	-0.904909
H	2.512691	-0.862382	1.185124
H	4.054035	0.800667	1.264940
H	4.009413	1.766543	-0.219372
H	3.456303	2.470176	1.293390
H	1.111291	4.362636	-2.749117
O	-1.492519	-3.023730	2.863036
O	-2.193085	-1.498919	1.361816
H	-3.836262	-2.748731	1.645183
H	-4.154629	-1.033019	1.246404
H	-3.611702	-1.492249	2.889051

```
 H     0.409417    -1.241874    -0.610405
 C    -1.249218    -2.238459     1.968635
 H    -0.419752    -0.161127     0.464396
 H     0.886737    -2.361004     2.098516
 C    -3.528403    -1.714682     1.822183
 C     0.416640    -0.854774     0.568791
 C     0.108597    -2.015883     1.417537

  45
 TS(IX2E4Z-X2E4Z)
scf done: -1051.925998
Pd     0.177079    -2.670899    -0.338157
 H    -0.500611    -6.478449    -2.528259
 H     1.054899    -6.848973    -1.767045
 C     0.406475    -4.821315    -1.574500
 H     0.995480    -5.931757    -3.307369
 C     0.510639    -6.098515    -2.343761
 O     0.351858    -3.705746    -2.188674
 O     0.340208    -4.830983    -0.307433
 C     0.607550     3.502311    -2.094685
 C     1.681918     2.658145    -1.416043
 C     1.103939     1.941626    -0.189753
 C     0.636737     2.989639     0.835016
 C    -0.145073     4.100135     0.195117
 C    -0.187354     4.336120    -1.122850
 C    -1.021951     5.430771    -1.716040
 C     2.061408     0.957522     0.439155
 C     1.681530    -0.258751     0.884698
 C     3.491911     1.382349     0.582917
 H    -0.721176     4.739194     0.865149
 H    -0.081570     2.857919    -2.662017
 H     2.094483     1.932479    -2.126599
 H     2.509127     3.307759    -1.103281
 H     0.211365     1.401476    -0.530183
 H     1.504189     3.403807     1.371378
 H     0.023054     2.500535     1.602940
 H    -0.397449     6.158241    -2.251185
 H    -1.728662     5.029063    -2.454523
 H    -1.595138     5.966460    -0.953326
 H     2.433620    -0.916599     1.318923
 H     4.069879     0.654577     1.158433
 H     3.968604     1.496144    -0.398352
 H     3.569434     2.356337     1.081006
 H     1.069009     4.162049    -2.842059
 O    -1.641447    -3.128168     2.880171
 O    -2.311156    -1.447233     1.540188
 H    -3.959318    -2.717349     1.653651
 H    -4.269908    -0.968665     1.429805
 H    -3.764211    -1.597902     3.027448
 H     0.012463    -1.210220    -0.767582
 C    -1.383523    -2.251194     2.081213
 H    -0.493498    -0.090630     0.684649
 H     0.763352    -2.447108     2.196551
 C    -3.656973    -1.707980     1.945378
 C     0.317624    -0.789726     0.872338
 C    -0.009916    -1.961693     1.603148
```

```
45
X(2E4Z)
scf done: -1051.932082
Pd    -0.359622    -2.579251    -0.241007
 H     1.129321    -5.983380    -2.692152
 H     2.705439    -5.242065    -2.313507
 C     0.992451    -4.114261    -1.696889
 H     1.692765    -4.612258    -3.649721
 C     1.685574    -5.041192    -2.643947
 O    -0.154645    -3.639846    -2.029981
 O     1.497128    -3.804436    -0.584326
 C     0.515807     3.150564    -2.190226
 C     1.523354     2.192949    -1.562215
 C     0.997437     1.670340    -0.219282
 C     0.830598     2.851265     0.753497
 C     0.154303     4.024842     0.105867
 C    -0.014510     4.169973    -1.215051
 C    -0.738358     5.340919    -1.808010
 C     1.864118     0.592007     0.382165
 C     1.369409    -0.532349     0.957658
 C     3.346090     0.805628     0.352877
 H    -0.229609     4.794912     0.775752
 H    -0.329194     2.588662    -2.616994
 H     1.726936     1.359888    -2.245054
 H     2.473176     2.718682    -1.403137
 H    -0.003276     1.263856    -0.406998
 H     1.810606     3.156982     1.150085
 H     0.249057     2.524733     1.625508
 H    -0.080147     5.918045    -2.470787
 H    -1.582994     5.008015    -2.425963
 H    -1.125408     6.014857    -1.037804
 H     2.074559    -1.252507     1.371437
 H     3.873481     0.043002     0.931323
 H     3.722501     0.773022    -0.677155
 H     3.614921     1.792007     0.749312
 H     0.977392     3.670861    -3.040464
 O    -2.193032    -3.154962     2.952246
 O    -2.654739    -1.150604     2.038650
 H    -4.483456    -2.149529     2.006660
 H    -4.510383    -0.370670     2.202380
 H    -4.039739    -1.421678     3.572987
 H    -1.724492    -1.973774    -0.418300
 C    -1.846927    -2.180181     2.317119
 H    -0.775291    -0.122134     0.840788
 H     0.258035    -2.648490     2.289085
 C    -4.004385    -1.292758     2.488379
 C    -0.031651    -0.872205     1.093351
 C    -0.468701    -2.007677     1.790939

45
X'(2E4Z)
scf done: -1051.919219
Pd     0.331551    -2.608186     0.466419
 H     2.019530    -4.641895    -3.257885
 H     0.953301    -3.745399    -4.374666
```

C	0.659729	-3.285841	-2.289368
H	0.323343	-5.097292	-3.394098
C	1.010386	-4.245169	-3.406637
O	0.334683	-2.122367	-2.515547
O	0.745226	-3.823495	-1.105655
C	0.580334	2.662693	-2.326482
C	1.630072	1.963517	-1.468735
C	1.047069	1.613057	-0.094066
C	0.655933	2.909808	0.637893
C	-0.088579	3.858983	-0.255725
C	-0.150417	3.755482	-1.589778
C	-0.946836	4.705281	-2.432477
C	1.973055	0.778693	0.754402
C	1.553725	-0.273845	1.503182
C	3.417839	1.168656	0.785148
H	-0.618507	4.674854	0.236868
H	-0.151430	1.931602	-2.702924
H	1.987113	1.059319	-1.975012
H	2.494981	2.626478	-1.341105
H	0.122611	1.052263	-0.274359
H	1.553415	3.402472	1.041729
H	0.037789	2.662193	1.510728
H	-0.301457	5.240659	-3.141271
H	-1.687877	4.165680	-3.037229
H	-1.477001	5.445094	-1.825126
H	2.293367	-0.806464	2.101627
H	3.974120	0.597529	1.532642
H	3.885470	0.999521	-0.192716
H	3.535898	2.236772	1.003327
H	1.054626	3.086401	-3.222072
O	-1.790618	-3.072370	3.610257
O	-2.454332	-1.416082	2.237892
H	-4.064453	-2.738967	2.257250
H	-4.419773	-0.998876	2.035293
H	-3.973501	-1.629188	3.650199
H	-1.121880	-2.690884	0.172958
C	-1.529787	-2.191130	2.817460
H	-0.612311	-0.183400	1.163298
H	0.610626	-2.308525	3.051816
C	-3.809582	-1.723889	2.573857
C	0.194661	-0.771548	1.592029
C	-0.143220	-1.859898	2.405749

45
TS(X'2E4Z-XI2E4Z)
scf done: -1051.919161

Pd	0.374744	-2.588324	0.455933
H	1.988669	-4.473726	-3.374889
H	0.656137	-3.878361	-4.403430
C	0.500458	-3.329237	-2.324686
H	0.409060	-5.248372	-3.286103
C	0.911598	-4.285732	-3.424224
O	-0.065744	-2.268186	-2.570920
O	0.815391	-3.757754	-1.133732
C	0.624284	2.659873	-2.316171
C	1.668912	1.981924	-1.435539

C	1.068005	1.635382	-0.067446
C	0.649903	2.933276	0.646896
C	-0.092315	3.865223	-0.266623
C	-0.132014	3.749337	-1.600528
C	-0.927183	4.681454	-2.463901
C	1.990388	0.816916	0.800345
C	1.574302	-0.244835	1.537254
C	3.427626	1.231301	0.860569
H	-0.640291	4.678426	0.210364
H	-0.092020	1.916190	-2.697704
H	2.046534	1.078356	-1.928130
H	2.522951	2.657213	-1.299499
H	0.153269	1.062390	-0.258650
H	1.534913	3.440607	1.059935
H	0.021364	2.685849	1.512330
H	-0.277679	5.218976	-3.167289
H	-1.651742	4.127137	-3.075279
H	-1.476184	5.419569	-1.871419
H	2.311324	-0.769125	2.146150
H	3.978203	0.668282	1.618396
H	3.918180	1.071943	-0.107672
H	3.523381	2.300756	1.082982
H	1.107325	3.082099	-3.207782
O	-1.764972	-3.108236	3.567672
O	-2.428532	-1.433729	2.217550
H	-4.027808	-2.769576	2.196054
H	-4.395202	-1.028617	2.002850
H	-3.959865	-1.685339	3.610226
H	-1.072070	-2.643734	0.131577
C	-1.503349	-2.211638	2.792489
H	-0.587402	-0.178078	1.170201
H	0.635602	-2.312840	3.047237
C	-3.784422	-1.758581	2.534024
C	0.221025	-0.762948	1.600667
C	-0.115861	-1.863219	2.399005

45
XI(2E4Z)
scf done: -1051.948046

Pd	0.388971	-2.794805	0.768460
H	1.268829	-4.597182	-3.487697
H	1.225407	-2.923966	-4.123583
C	0.460513	-3.148921	-2.158591
H	-0.269658	-3.854715	-4.015552
C	0.694108	-3.673090	-3.529429
O	-0.176043	-1.995507	-2.111379
O	0.824471	-3.732804	-1.133735
C	0.664244	2.409562	-2.355235
C	1.704410	1.840924	-1.394836
C	1.072353	1.546799	-0.028509
C	0.559003	2.861090	0.583006
C	-0.193060	3.696991	-0.411946
C	-0.174371	3.498820	-1.736794
C	-0.980270	4.334525	-2.685129
C	2.000336	0.818379	0.915230
C	1.618725	-0.254977	1.645123

C	3.404781	1.335057	1.032439
H	-0.799929	4.508578	-0.008687
H	0.001621	1.607273	-2.715730
H	2.146746	0.930145	-1.816123
H	2.519474	2.565628	-1.271940
H	0.193755	0.917260	-0.216764
H	1.399356	3.440171	0.995476
H	-0.089388	2.635536	1.439972
H	-0.331465	4.862989	-3.396153
H	-1.651483	3.707939	-3.287777
H	-1.588897	5.076803	-2.159848
H	2.362416	-0.716049	2.296664
H	3.952149	0.823680	1.829289
H	3.962221	1.193801	0.097542
H	3.423342	2.411730	1.242800
H	1.159191	2.802329	-3.254088
O	-1.688225	-3.084064	3.811341
O	-2.348326	-1.539651	2.314049
H	-3.903859	-2.926677	2.357423
H	-4.320274	-1.217997	2.030523
H	-3.902795	-1.744139	3.688911
H	-0.279436	-1.773496	-1.149729
C	-1.408419	-2.243971	2.975186
H	-0.524412	-0.330877	1.213224
H	0.711961	-2.225664	3.320983
C	-3.696023	-1.886381	2.624749
C	0.299005	-0.876884	1.668668
C	-0.028162	-1.894899	2.592286

45
TS(VIII4Z-IX2Z4Z)
scf done: -1051.913282

Pd	-0.765711	-1.086137	0.861363
H	1.130476	-3.036580	4.517151
H	1.205905	-4.268276	3.235653
C	0.110362	-2.531166	2.708177
H	-0.293451	-4.060185	4.147742
C	0.563179	-3.528936	3.725968
O	0.613440	-1.381389	2.629001
O	-0.805927	-2.880641	1.875937
C	1.043983	3.855063	0.197828
C	0.790669	3.155554	-1.134398
C	0.568189	1.652351	-0.922682
C	1.842812	1.037181	-0.324001
C	2.391036	1.857384	0.807535
C	2.031175	3.121732	1.069919
C	2.582743	3.888443	2.234079
C	0.118565	0.930806	-2.173193
C	-1.013179	0.210602	-2.259609
C	1.006843	1.053679	-3.382627
H	3.127499	1.374277	1.450855
H	0.099197	3.975609	0.750285
H	-0.073873	3.603990	-1.637944
H	1.656718	3.304550	-1.792361
H	-0.212050	1.550596	-0.155167
H	2.613901	0.918967	-1.099791

H	1.623742	0.018644	0.030393
H	3.127857	4.780032	1.896869
H	1.775188	4.248963	2.885173
H	3.261466	3.280123	2.839462
H	-1.215790	-0.283425	-3.209000
H	0.635810	0.440794	-4.209168
H	1.059618	2.091540	-3.734654
H	2.037622	0.745420	-3.170921
H	1.408527	4.876675	0.022667
O	-2.842423	-3.516712	-1.105634
O	-0.965121	-2.666413	-1.994316
H	-0.671647	-4.729757	-1.969277
H	0.125191	-3.791238	-3.268476
H	-1.633684	-4.115950	-3.336739
H	-3.063375	0.226946	-1.696650
C	-2.059413	-2.594060	-1.219856
H	-2.016914	0.813243	-0.445283
H	-3.131494	-1.346050	0.079131
C	-0.785273	-3.906415	-2.679802
C	-2.085544	0.035472	-1.224704
C	-2.196645	-1.298802	-0.489837

45
IX(2Z4Z)
scf done: -1051.926198

Pd	-0.693133	-2.325521	1.187455
H	0.365511	-5.343862	4.374603
H	-0.170070	-6.336353	3.000724
C	-0.483026	-4.258205	2.738056
H	-1.378619	-5.647153	4.091669
C	-0.411376	-5.463862	3.618015
O	0.481090	-3.442912	2.655438
O	-1.527698	-4.054339	2.030581
C	0.862032	4.478904	-0.559010
C	0.872445	3.323142	-1.555012
C	0.960319	1.980811	-0.819848
C	2.283488	1.918622	-0.037443
C	2.560032	3.188582	0.714134
C	1.918791	4.346586	0.507533
C	2.201784	5.576254	1.316732
C	0.813102	0.780228	-1.726775
C	0.087456	-0.312229	-1.424965
C	1.562504	0.819472	-3.028678
H	3.331226	3.141906	1.483818
H	-0.124807	4.558691	-0.077452
H	-0.025431	3.359469	-2.182957
H	1.735885	3.430309	-2.223738
H	0.154118	1.973069	-0.075982
H	3.117239	1.702189	-0.722776
H	2.254611	1.074542	0.664562
H	2.560035	6.395253	0.679113
H	1.289367	5.943078	1.805763
H	2.951994	5.391955	2.091618
H	0.087631	-1.145294	-2.121020
H	1.502747	-0.138636	-3.551961
H	1.161572	1.593808	-3.693723

H	2.621465	1.059650	-2.873619
H	1.002980	5.430573	-1.089459
O	-3.532064	-2.595896	-1.373685
O	-1.370720	-2.865471	-1.925280
H	-2.294164	-4.647987	-2.484216
H	-0.873729	-4.113399	-3.433252
H	-2.458494	-3.308614	-3.649004
H	-0.941795	0.427360	0.334184
C	-2.361609	-2.321139	-1.200869
H	0.110032	-0.948386	0.610048
H	-2.764113	-0.989553	0.396790
C	-1.787104	-3.790332	-2.933663
C	-0.727046	-0.505821	-0.189622
C	-1.902756	-1.401487	-0.128727

45
TS(IX2Z4Z-X2Z4Z)
scf done: -1051.920460

Pd	-0.720292	-1.986986	1.113058
H	0.581550	-4.948439	4.221496
H	0.670586	-5.885227	2.719845
C	-0.282097	-4.006443	2.505373
H	-0.878804	-5.761400	3.571078
C	0.035089	-5.221180	3.316639
O	0.488079	-2.991620	2.545686
O	-1.299225	-3.979551	1.747407
C	1.056786	4.452603	-0.651828
C	1.090597	3.265745	-1.609981
C	0.886650	1.954648	-0.843583
C	2.044018	1.764724	0.153311
C	2.341815	3.020725	0.919717
C	1.892095	4.237348	0.584280
C	2.181778	5.455397	1.408446
C	0.751808	0.738611	-1.728663
C	-0.094218	-0.284951	-1.478308
C	1.661859	0.658098	-2.917568
H	2.959398	2.912756	1.811976
H	0.020476	4.669559	-0.350208
H	0.321694	3.384358	-2.382221
H	2.060140	3.242130	-2.123209
H	-0.027258	2.073304	-0.249143
H	2.946781	1.427599	-0.378846
H	1.794952	0.955004	0.852763
H	2.742704	6.200430	0.828851
H	1.251346	5.946670	1.723265
H	2.759060	5.213620	2.305908
H	-0.074109	-1.147376	-2.136638
H	1.585789	-0.312125	-3.415200
H	1.421888	1.437588	-3.650888
H	2.707830	0.817195	-2.627443
H	1.403817	5.358295	-1.167627
O	-3.694284	-2.722099	-1.234162
O	-1.557587	-2.837024	-1.919632
H	-2.339462	-4.737481	-2.266137
H	-1.035615	-4.163372	-3.350315
H	-2.699677	-3.525385	-3.523550

H	-1.235530	0.610173	0.134529
C	-2.547160	-2.330412	-1.171759
H	-0.045551	-0.621533	0.989944
H	-2.975838	-0.919195	0.364370
C	-1.944287	-3.881336	-2.818578
C	-1.057080	-0.333762	-0.376635
C	-2.128815	-1.261677	-0.227722

45
X(2Z4Z)
scf done: -1051.927685

Pd	-1.307059	-1.477373	1.113076
H	0.852823	-4.718958	3.298062
H	2.154907	-3.563721	3.014091
C	0.379225	-3.175626	1.908725
H	1.781486	-4.790814	1.771678
C	1.354456	-4.128417	2.525581
O	-0.249331	-2.361193	2.680189
O	0.167167	-3.150766	0.668767
C	1.455540	4.157127	-0.716601
C	1.474882	2.887212	-1.562119
C	0.841301	1.724481	-0.790525
C	1.672982	1.440047	0.474420
C	2.039261	2.697934	1.206411
C	1.929789	3.931836	0.696307
C	2.266141	5.159993	1.486918
C	0.682342	0.460970	-1.598671
C	-0.375001	-0.384159	-1.482950
C	1.796044	0.105678	-2.533867
H	2.407339	2.577296	2.225841
H	0.440599	4.582590	-0.690937
H	0.945201	3.056774	-2.506616
H	2.512648	2.637043	-1.815381
H	-0.144028	2.066392	-0.452121
H	2.583998	0.881959	0.208462
H	1.102897	0.776047	1.138522
H	3.081645	5.723038	1.014111
H	1.407455	5.842919	1.535277
H	2.565556	4.917132	2.510888
H	-0.364519	-1.303097	-2.059328
H	1.659272	-0.890475	-2.961788
H	1.859100	0.827256	-3.357888
H	2.766015	0.135756	-2.022537
H	2.081170	4.927311	-1.187810
O	-4.152959	-2.708183	-1.025192
O	-2.100652	-2.739334	-1.939043
H	-2.685256	-4.737968	-1.862732
H	-1.573410	-4.245361	-3.176463
H	-3.309613	-3.813991	-3.254504
H	-1.610352	0.830816	-0.188824
C	-3.050731	-2.215784	-1.158662
H	-2.139849	-0.487923	1.878769
H	-3.524111	-0.499156	0.005279
C	-2.450612	-3.961657	-2.595291
C	-1.516269	-0.162712	-0.621442
C	-2.657291	-0.969435	-0.449983

```
   45
 X'(2Z4Z)
scf done: -1051.923907
Pd    -1.261969    -1.754083     1.243583
 H     1.849632    -4.387161     3.082363
 H     1.562748    -5.331758     1.589099
 C     0.235531    -3.675740     1.883158
 H     0.464410    -5.475654     2.986966
 C     1.088488    -4.787798     2.406854
 O    -0.284199    -2.854426     2.724650
 O     0.008345    -3.535933     0.652834
 C     1.672408     2.812682     1.542098
 C     1.162192     1.514163     0.924607
 C     1.075590     1.653775    -0.603028
 C     0.082213     2.765076    -0.982072
 C     0.277218     3.994812    -0.143763
 C     0.998584     4.040488     0.984538
 C     1.193381     5.309075     1.759150
 C     0.842464     0.353053    -1.343168
 C    -0.226109    -0.476514    -1.222920
 C     1.934702    -0.016901    -2.297847
 H    -0.201505     4.907169    -0.500811
 H     2.759113     2.903875     1.391515
 H     1.826265     0.683528     1.189982
 H     0.176757     1.277167     1.341798
 H     2.062727     2.012657    -0.925698
 H    -0.954950     2.413737    -0.892253
 H     0.209122     3.019644    -2.042772
 H     0.789255     5.216990     2.775827
 H     2.261193     5.539868     1.871536
 H     0.710152     6.162469     1.274025
 H    -0.231456    -1.380598    -1.822458
 H     1.731784    -0.951143    -2.827554
 H     2.082635     0.782361    -3.036026
 H     2.888070    -0.116779    -1.762002
 H     1.531315     2.786755     2.631147
 O    -4.114502    -2.654199    -0.993180
 O    -2.086157    -2.646042    -1.959131
 H    -2.728864    -4.624668    -2.079839
 H    -1.637725    -4.023968    -3.365615
 H    -3.361336    -3.539068    -3.345720
 H    -1.426362     0.684740     0.154937
 C    -3.000309    -2.183018    -1.101835
 H    -1.986511    -0.742456     2.087354
 H    -3.400459    -0.560188     0.215132
 C    -2.490177    -3.780652    -2.731765
 C    -1.370042    -0.269359    -0.359910
 C    -2.552973    -1.029874    -0.276184

   45
 TS(X'2Z4Z-XI2Z4Z)
scf done: -1051.911208
Pd    -1.553556    -2.163181     0.731657
 H    -0.659890    -4.727803     4.439539
 H     1.039548    -4.184464     4.464061
```

C	-0.147672	-3.367249	2.861244
H	0.478279	-5.384126	3.266023
C	0.209166	-4.480394	3.821421
O	-1.094269	-3.679167	2.027218
O	0.424606	-2.277223	2.892526
C	1.486367	2.548915	1.731469
C	1.014084	1.280066	1.028382
C	0.998750	1.493499	-0.492823
C	0.017455	2.618095	-0.863413
C	0.167319	3.807154	0.040296
C	0.834046	3.800387	1.202532
C	0.985605	5.030354	2.045957
C	0.807169	0.231640	-1.307690
C	-0.290774	-0.569503	-1.334041
C	1.967820	-0.114335	-2.187308
H	-0.298060	4.734328	-0.295581
H	2.578593	2.650578	1.638746
H	1.669630	0.441091	1.285188
H	0.011201	1.020120	1.385792
H	1.998334	1.870111	-0.750140
H	-1.020471	2.258940	-0.836600
H	0.190370	2.923383	-1.904055
H	0.532542	4.888202	3.035870
H	2.045691	5.257037	2.221790
H	0.523255	5.905516	1.579606
H	-0.273897	-1.414006	-2.016753
H	1.799035	-1.023411	-2.770070
H	2.183123	0.712584	-2.876802
H	2.873253	-0.248287	-1.580695
H	1.292914	2.468547	2.809697
O	-4.334586	-2.308966	-1.907806
O	-2.180789	-2.526269	-2.510895
H	-3.076813	-4.321325	-3.077655
H	-1.686658	-3.741989	-4.046561
H	-3.291978	-2.967592	-4.218877
H	-1.538862	0.449530	0.123216
C	-3.165167	-2.023993	-1.753650
H	-2.555249	-1.644032	1.696211
H	-3.576687	-0.651254	-0.183459
C	-2.597263	-3.447284	-3.525314
C	-1.496976	-0.403703	-0.547593
C	-2.719436	-1.082215	-0.692459

45
XI(2Z4Z)
scf done: -1051.939967

Pd	-1.473683	-2.229567	0.502804
H	0.708195	-4.527647	3.980685
H	-0.631257	-3.981285	5.032327
C	-0.509098	-2.988028	3.166069
H	0.793090	-2.958380	4.837951
C	0.137332	-3.664787	4.320479
O	-0.417226	-3.393032	2.002056
O	-1.197824	-1.908358	3.473638
C	1.627339	2.152204	1.665411
C	1.089013	1.001578	0.819744

C	0.987594	1.421482	-0.653463
C	0.014975	2.602952	-0.801998
C	0.236351	3.650711	0.249949
C	0.968060	3.472699	1.358338
C	1.189611	4.569389	2.356352
C	0.712518	0.287022	-1.622351
C	-0.367142	-0.531668	-1.630250
C	1.780369	0.091680	-2.658676
H	-0.231401	4.620946	0.078518
H	2.713838	2.255036	1.519415
H	1.741175	0.125608	0.917008
H	0.103088	0.705042	1.196270
H	1.979339	1.815519	-0.918200
H	-1.027150	2.256039	-0.767775
H	0.136385	3.051240	-1.797355
H	0.794132	4.292583	3.342584
H	2.261796	4.761456	2.497312
H	0.714599	5.505380	2.047188
H	-0.401844	-1.297440	-2.399667
H	1.543607	-0.718744	-3.353883
H	1.932291	1.012204	-3.238427
H	2.745607	-0.131818	-2.183893
H	1.498823	1.924356	2.732583
O	-4.335191	-2.538157	-1.942703
O	-2.232422	-2.559003	-2.729519
H	-3.043596	-4.402435	-3.268860
H	-1.793731	-3.696858	-4.337501
H	-3.460421	-3.044819	-4.343883
H	-1.552469	0.353794	-0.045389
C	-3.183483	-2.150706	-1.868510
H	-1.575949	-1.566795	2.617123
H	-3.571898	-0.818757	-0.266845
C	-2.671158	-3.481993	-3.726677
C	-1.502707	-0.501906	-0.715045
C	-2.723375	-1.214930	-0.821731

13. Author Contributions

Giovanni Poli: conceptualization: (lead); data curation (supporting); formal analysis (supporting); funding acquisition (lead); methodology (lead); project administration (lead); resources (lead); supervision (equal); visualization: (lead); writing – original draft (lead); writing – review & editing (lead). Alexandre Pradal: conceptualization (supporting); supervision (lead); data curation: (supporting); investigation (supporting); validation (supporting). Marco Di Matteo: data curation (supporting); investigation (lead); validation (lead). Luis F. Veiros: formal analysis (lead). Anna Gagliardi: investigation (supporting). Fabrice Gallou: conceptualization: (supporting); supervision (supporting).

14. References

(1) (a) Chen, M. S.; White, M. C. A sulfoxide-promoted, Catalytic Method for the Regioselective Synthesis of Allylic Acetates from Monosubstituted Olefins via C-H Oxidation *J. Am. Chem. Soc.* **2004**, *126*, 1346–1347; (b) Chen, M. S.; Narayanasamy, P.; Labenz, N. A.; White, M. C. Serial Ligand Catalysis: A Highly Selective Allylic C-H Oxidation *J. Am. Chem. Soc.* **2005**, *127*, 6970–6971.

(2) Chen, H.; Farizyan, M.; Ghiringhelli, F.; van Gemmeren, M. Sterically Controlled C-H Olefination of Heteroarenes *Angew. Chem. Int. Ed.* **2020**, *59*, 12213–12220.

(3) Paul, J.; Xavier, T.; Presset, M., Le Gall, E.; Léonel, E.; Pichon, C.; Condon, S. Cobalt-Zinc-Diimine Multicatalysis: Enhanced syn Diastereoselectivity in the Reductive Multicomponent Coupling of Arylbromides, Acrylates and Aldehydes *ChemistrySelect* **2018**, *3*, 13480–13486.

(4) Worzakowska, M. TG/DSC/FTIR/QMS Studies on the Oxidative Decomposition of Terpene Acrylate Homopolymers *J. Term. Anal. Calorim.* **2017**, *127*, 2025–2035.

(5) Commare, B.; Rigault, D.; Lemasson, I. A.; Deschamps, P.; Tomas, A.; Roussel, P.; Brabet, I.; Goudet, C.; Pin, J.-P.; Leroux, F. R.; Colobert, F.; Acher, F. C. Determination of the Absolute Configuration of Phosphinic Analogues of Glutamate *Org. Biomol. Chem.* **2015**, *13*, 1106–1112.

(6) Khong, S. N.; Kwon, O. Chiral Aminophosphines as Catalysts for Enantioselective Double-Michael Indoline Syntheses *Molecules* **2012**, *17*, 5626–5650.

(7) Arora, A.; Teegardin, K. A.; Weaver, J. D. Reductive Alkylation of 2-Bromoazoles via Photoinduced Electron Transfer: A Versatile Strategy to Csp^2-Csp^3 Coupled Products *Org. Lett.* **2015**, *17*, 3722–3725.

(8) Geoghegan, K.; Evans, P. Synthesis of (+)-Perillyl Alcohol from (+)-Limonene *Tetrahedron Lett.* **2014**, *55*, 1431–1433.

(9) For this compound it was not possible to obtain an HRMS, either using ESI or APCI ionization method. Analysis was still performed via GC/MS with electron impact as the ionization method, which provided the correct mass peak.

(10) Kim, I. S.; Dong, G. R.; Jung, Y. H. Palladium(II)-Catalyzed Isomerization of Olefins with Tributyltin Hydride *J. Org. Chem.* **2007**, *72*, 5424–5426.

(11) Mingoia, F.; Vitale, M.; Madec, D.; Prestat, G.; Poli, G. Pseudo-Domino Palladium-Catalyzed Allylic Alkylation/Mizoroki-Heck Coupling Reaction: A Key Sequence Towards (±)-Podophyllotoxin *Tetrahedron Lett.* **2008**, *49*, 760–763.

(12) (a) Bollenbach, M.; Wagner, P.; Aquino, P. G. V.; Bourguignon, J.-J.; Bihel, F.; Salomé, C.; Schmitt, M. D-Glucose: An Efficient Reducing Agent for a Copper(II)-Mediated Arylation of Primary Amines in Water *ChemSusChem* **2016**, *9*, 3244–3249; (b) Camp, J. E.; Dunsford, J. J.; Cannons, E. P.; Restorick, W. J.; Gadzhieva, A.; Fay, M. W.; Smith, R. J. Glucode-Derived Palladium(0) Nanoparticles as In Situ-Formed Catalysts for Suzuki-Miyaura Cross-Coupling Reactions in Isopropanol *ACS Sustainable Chem. Eng.* **2014**, *2*, 500–505.

(13) Jacobi, M.; Gallou, F.; Sparr, C.; Parmentier, M. A General Protocol for Robust Sonogashira Reactions in Micellar Medium *Helv. Chim. Acta* **2019**, *102*, e1900024.

(14) GAUSSIAN 09, Revision A.01, Frisch, M. J.; Trucks, G. W.; Schlegel, H. B.; Scuseria, G. E.; Robb, M. A.; Cheeseman, J. R.; Scalmani, G.; Barone, V.; Mennucci, B.; Petersson, G. A.; Nakatsuji, H.; Caricato, M.; Li, X.; Hratchian, H. P.; Izmaylov, A. F.; Bloino, J.; Zheng, G.; Sonnenberg, J. L.; Hada, M.; Ehara, M.; Toyota, K.; Fukuda, R.; Hasegawa, J.; Ishida, M.; Nakajima, T.; Honda, Y.; Kitao, O.; Nakai, H.; Vreven, T.; Montgomery, Jr., J. A.; Peralta, J. E.; Ogliaro, F.; Bearpark, M.; Heyd, J. J.; Brothers, E.; Kudin, K. N.; Staroverov, V. N.; Kobayashi, R.; Normand, J.; Raghavachari, K.; Rendell, A.; Burant, J. C.; Iyengar, S. S.; Tomasi, J.; Cossi, M.; Rega, N.; Millam, J. M.; Klene, M.; Knox, J. E.; Cross, J. B.; Bakken, V.; Adamo, C.; Jaramillo, J.; Gomperts, R.; Stratmann, R. E.; Yazyev, O.; Austin, A. J.; Cammi, R.; Pomelli, C.; Ochterski, J. W.; Martin, R. L.; Morokuma, K.; Zakrzewski, V. G.; Voth, G. A.; Salvador, P.; Dannenberg, J. J.; Dapprich, S.; Daniels, A. D.; Farkas, Ö.; Foresman, J. B.; Ortiz, J. V.; Cioslowski, J.; Fox, D. J. Gaussian, Inc., Wallingford CT, 2009.

(15) Hehre, W. J.; Radom, L.; Schleyer, P. v.R.; Pople, J. A. *Ab Initio Molecular Orbital Theory*, John Wiley & Sons, NY, 1986.

(16) Parr, R. G.; Yang, W. *Density Functional Theory of Atoms and Molecules*; Oxford University Press: New York, 1989.

(17) (a) Perdew, J. P.; Burke, K.; Ernzerhof, M. Generalized Gradient Approximation Made Simple *Phys. Rev. Lett.* **1996**, *77*, 3865-3868; (b) Perdew, J. P.; Burke, K.; Ernzerhof, M. Generalized Gradient Approximation Made Simple *Phys. Rev. Lett.* **1997**, *78*, 1396-1396. (c) Perdew, J. P. Density-functional approximation for the correlation energy of the inhomogeneous electron gas *Phys. Rev. B* **1986**, *33*, 8822-8824.

(18) (a) Haeusermann, U.; Dolg, M.; Stoll, H.; Preuss, H.; Schwerdtfeger, P.; Pitzer, R. M. Accuracy of energy-adjusted quasirelativistic ab initio pseudopotentials *Mol. Phys.* **1993**, *78*, 1211-1224. (b) Kuechle, W.; Dolg, M.; Stoll, H.; Preuss, H. Energy-adjusted pseudopotentials for the actinides. Parameter sets and test calculations for thorium and thorium monoxide *J. Chem. Phys.* **1994**, *100*, 7535-7542. (c) Leininger, T.; Nicklass, A.; Stoll, H.; Dolg, M.; Schwerdtfeger, P. The accuracy of the pseudopotential approximation. II. A comparison of various core sizes for indium pseudopotentials in calculations for spectroscopic constants of InH, InF, and InCl *J. Chem. Phys.* **1996**, *105*, 1052-1059.

(19) Ehlers, A. W.; Böhme, M.; Dapprich, S.; Gobbi, A.; Höllwarth, A.; Jonas, V.; Köhler, K. F.; Stegmann, R.; Veldkamp, A.; Frenking, G. A set of f-polarization functions for pseudo-potential basis sets of the transition metals Sc−Cu, Y−Ag and La−Au *Chem. Phys. Lett.* **1993**, *208*, 111-114.

(20) (a) Ditchfield, R.; Hehre, W. J.; Pople, J. A. Self-Consistent Molecular-Orbital Methods. IX. An Extended Gaussian-Type Basis for Molecular-Orbital Studies of Organic Molecules *J. Chem. Phys.* **1971**, *54*, 724-728. (b) Hehre, W. J.; Ditchfield, R.; Pople, J. A. Self-Consistent Molecular Orbital Methods. 12. Further extensions of Gaussian-type basis sets for use in molecular-orbital studies of organic-molecules *J. Chem. Phys.* **1972**, *56*, 2257-2261. (c) Hariharan, P. C.; Pople, J. A. Accuracy of AH equilibrium geometries by single determinant molecular-orbital theory *Mol. Phys.* **1974**, *27*, 209-214. (d) Gordon, M. S. The isomers of silacyclopropane *Chem. Phys. Lett.* **1980**, *76*, 163-168. (e) Hariharan, P. C.; Pople, J. A. Influence of polarization functions on molecular-orbital hydrogenation energies *Theor. Chim. Acta* **1973**, *28*, 213-222.

(21) (a) Peng, C.; Ayala, P. Y.; Schlegel, H. B.; Frisch, M. J. Using redundant internal coordinates to optimize equilibrium geometries and transition states *J. Comp. Chem.* **1996**, *17*, 49-56. (b) Peng, C.; Schlegel, H. B. Combining Synchronous Transit and Quasi-Newton Methods for Finding Transition States *Israel J. Chem.* **1993**, *33*, 449-454.

(22) (a) McClean, A. D.; Chandler, G. S. Contracted Gaussian basis sets for molecular calculations. I. Second row atoms, *Z*=11-18 *J. Chem. Phys.* **1980**, *72*, 5639-5648. (b) Krishnan, R.; Binkley, J. S.; Seeger, R.; Pople, J. A. Self-consistent molecular orbital methods. XX. A basis set for correlated wave functions *J. Chem. Phys.* **1980**, *72*, 650-654. (c) Wachters, A. J. H. Gaussian Basis Set for Molecular Wavefunctions Containing Third-Row Atoms *J. Chem. Phys.* **1970**, *52*, 1033-1036. (d) Hay, P. J. Gaussian basis sets for molecular calculations - representation of 3D orbitals in transition-metal atoms *J. Chem. Phys.* **1977**, *66*, 4377-4384. (e) Raghavachari, K.; Trucks, G. W. Highly correlated systems: Excitation energies of first row transition metals Sc-Cu *J. Chem. Phys.* **1989**, *91*, 1062-1065. (f) Binning Jr., R. C.; Curtiss, L. A. Compact contracted basis-sets for 3rd-row atoms - Ga-Kr *J. Comp. Chem.* **1990**, *11*, 1206-1216. (g) McGrath, M. P.; Radom, L. Extension of Gaussian-1 (G1) theory to bromine-containing molecules *J. Chem. Phys.* **1991**, *94*, 511-516. (h) Curtiss, L. A.; McGrath, M. P.; Blaudeau, J.-P.; Davis, N. E.; Binning Jr., R. C.; Radom, L. Extension of Gaussian-2 theory to molecules containing third-row atoms Ga-Kr *J. Chem. Phys.*, **1995**, *103*, 6104-6113. (i) Clark, T.; Chandrasekhar, J.; Spitznagel, G. W.; Schleyer, P. v. R. Efficient diffuse function-augmented basis-sets for anion calculations. 3. The 3-21+G basis set for 1st-row elements, Li-F *J. Comp. Chem.* **1983**, *4*, 294-301. (j) Frisch, M. J.; Pople, J. A.; Binkley, J. S. Self-Consistent Molecular Orbital Methods. 25. Supplementary Functions for Gaussian Basis Sets *J. Chem. Phys.* **1984**, *80*, 3265-3269.

(23) (a) Cancès, M. T.; Mennucci, B.; Tomasi, J. A new integral equation formalism for the polarizable continuum model: Theoretical background and applications to isotropic and anistropic dielectrics *J. Chem. Phys.* **1997**, *107*, 3032-3041. (b) Cossi, M.; Barone, V.; Mennucci, B.; Tomasi, J. Ab initio study of ionic solutions by a polarizable continuum dielectric model *Chem. Phys. Lett.* **1998**, *286*, 253-260. (c) Mennucci, B.; Tomasi, J. Continuum solvation models: A new approach to the problem of solute's charge distribution and cavity boundaries *J. Chem. Phys.* **1997**, *106*, 5151-5158. (d) Tomasi, J.; Mennucci, B.; Cammi, R. Quantum mechanical continuum solvation models *Chem. Rev.* **2005**, *105*, 2999-3094.

(24) Marenich, A. V.; Cramer, C. J.; Truhlar, D. G. Universal solvation model based on solute electron density and a continuum model of the solvent defined by the bulk dielectric constant and atomic surface tensions *J. Phys. Chem. B*, **2009**, *113*, 6378-6396.

(25) Grimme, S.; Antony, J.; Ehrlich, S.; Krieg, H. A consistent and accurate ab initio parameterization of density functional dispersion correction (DFT-D) for the 94 elements H-Pu *J. Chem. Phys.* **2010**, *132*, 154104.

(26) (a) Becke, A. D.; Johnson, E. R. A density-functional model of the dispersion interaction *J. Chem. Phys.* **2005**, *122*, 154101. (b) Johnson, E. R.; Becke, A. D. A post-Hartree-Fock model of intermolecular interactions *J. Chem. Phys.* **2005**, *123*, 24101. (c) Johnson, E. R.; Becke, A. D. A post-Hartree-Fock model of intermolecular interactions: Inclusion of higher-order corrections *J. Chem. Phys.* **2006**, *124*, 174104.
